# Multi-species integrative biclustering

**DOI:** 10.1186/gb-2010-11-9-r96

**Published:** 2010-09-29

**Authors:** Peter Waltman, Thadeous Kacmarczyk, Ashley R Bate, Daniel B Kearns, David J Reiss, Patrick Eichenberger, Richard Bonneau

**Affiliations:** 1 Computer Science Department, Warren Weaver Hall (Room 305), 251 Mercer Street, New York, NY 10012, USA; 2 Computational Biology Program, New York University, Warren Weaver Hall (Room 1105), 251 Mercer Street, New York, NY 10012, USA; 3 Center for Genomics and Systems Biology, Department of Biology, New York University, Silver Building (Room 1009), 100 Washington Square East, New York, NY 10003, USA; 4 Department of Biology, Indiana University, 1001 East 3rd Street, Jordan Hall 142, Bloomington, IN 47405, USA; 5 Institute for Systems Biology, 1441 North 34th Street, Seattle, WA 98103, USA

## Abstract

We describe an algorithm, multi-species cMonkey, for the simultaneous biclustering of heterogeneous multiple-species data collections and apply the algorithm to a group of bacteria containing *Bacillus subtilis*, *Bacillus anthracis*, and *Listeria monocytogenes*. The algorithm reveals evolutionary insights into the surprisingly high degree of conservation of regulatory modules across these three species and allows data and insights from well-studied organisms to complement the analysis of related but less well studied organisms.

## Background

The rapidly increasing volume of genome scale data has enabled global regulatory network inference and genome-wide prediction of gene function within single organisms. In this work, we exploit another advantage of the growing quantity of genomics data: by comparing genome-wide datasets for closely related organisms, we can add a critical evolutionary component to systems biology data analysis. Whereas several well-developed tools exist for identifying orthologous genes on the basis of sequence similarity, the identification of conserved co-regulated gene groups (modules) is a relatively recent problem requiring development of new methods. Here, we present an algorithm that performs integrative biclustering for multiple-species datasets in order to identify conserved modules and the conditions under which these modules are active. The advantages of this method are that conserved modules are more likely to be biologically significant than co-regulated gene groups lacking detectable conservation, and the identification of these conserved modules can provide a basis for investigating the evolution of gene regulatory networks.

Clustering has long been a popular tool in analyzing systems biology data types (for example, the clustering of microarray data to generate putative co-regulated gene groups). Most genomics studies employ clustering methods that require genes to participate in mutually exclusive clusters, such as hierarchical agglomerative clustering [1], k-means clustering [2] and singular value decomposition derived methods [3-5]. Because most genes are unlikely to be co-regulated under every possible condition (for instance, bacterial genes can have more than one transcription start site and, in that case, each site will be regulated by a different set of transcription factors depending on the cell's state), defining mutually exclusive gene clusters cannot capture the complexity of transcriptional regulatory networks. Clearly, sophisticated integrative methods are needed to arrive at the identification of more mechanistically meaningful condition-dependent conserved modules.

Biclustering refers to the simultaneous clustering of both genes and conditions [6,7]. Early work [8] introduced the idea of biclustering as 'direct clustering' [9], node deletion problems on graphs [10], and biclustering [11]. More recently, biclustering has been used in several studies to address the biologically relevant condition dependence of co-expression patterns [6,12-19]. Additional genome-wide data (such as association networks and transcription factor binding sites) greatly improves the performance of these approaches [19-22]. Examples include the most recent version of SAMBA, which incorporates experimentally validated protein-protein and protein-DNA associations into a Bayesian framework [19], and cMonkey [20], an algorithm we recently introduced.

cMonkey integrates expression and sequence data, metabolic and signaling pathways [23], protein-protein interactions, and comparative genomics networks [24-26] to estimate condition dependent co-regulated modules. We have previously shown that cMonkey can be used to 'pre-cluster' genes prior to learning global regulatory networks [27]. Biclusters are iteratively optimized, starting with a random or semi-random seed, via a Monte Carlo Markov chain process. At each iteration, each bicluster's state is updated based upon conditional probability distributions computed using the bicluster's previous state. This enables cMonkey to determine the probability that a given gene or condition belongs in the bicluster, dependent upon the current state of the bicluster. The components of this conditional probability (one for each of the different data types) are modeled independently as *P*-values based upon individual data likelihoods, which are combined to determine the full conditional probability of a given gene or condition belonging to a given bicluster.

Previous multi-species clustering methods generally fall into two classes (for reviews see [17,28]). The first class attempts to match conditions between species in order to identify similarities and differences for a given cell process [29-32]. By requiring matched conditions, this approach is not well suited to large sets of public experiments, as it is limited to only the conditions that have direct analogs for both species. The second class of multi-species clustering methods employs a strategy where the datasets for each organism are reduced to a unit-less measure of co-expression (for example Pearson's correlation) and are then used to compare co-expression patterns in multiple species [33-38]. This second class includes methods analyzing the conservation of individual orthologous pairs [37,38] and those seeking to identify larger conserved modules [33,34,36]. The common objective is to gain insight into the evolution of related species, including the role of duplication in regulatory network evolution and the occurrence of convergent evolution versus conserved co-expression [35,38]. However, none of these studies can be considered a true multi-species biclustering algorithm; for example, both Bergmann *et al*. [34] and Tanay *et al*. [36] performed the analyses of the different species sequentially. Furthermore, with the exception of Tanay *et al*. [36], the methods were limited to considering only expression data.

Below, we present multi-species cMonkey, a biclustering framework that enables us to integrate data across multiple species and multiple data-types simultaneously. Our approach maintains the independence of the organism-specific data while still allowing for true biclustering. Specifically, gene membership in multiple clusters is possible and integration of a variety of data types remains an integral part of the approach. Once the conserved modules have been identified, our method further allows the discovery of species-specific modifications (which we term 'elaborations', that is, the addition of species-specific genes that fit well with the conserved core of the bicluster according to the multi-data score). The ability to find species specific elaborations of conserved co-regulated core sets of genes is a unique strength of the method and is critical to understanding the evolution and function of conserved modules.

Our multi-species biclustering method was applied to all pairings that are possible for three closely related species of Firmicutes: *Bacillus subtilis*, *Bacillus anthracis *and *Listeria monocytogenes*. As one of the best-studied bacterial model organisms, *B. subtilis *was selected due to the wealth of publicly available genomic data and the large amount of knowledge accumulated on this organism over the years. Additionally, *B. subtilis *and *B. anthracis *have similar life cycles, alternating between vegetative cell and dormant spore states [39-43]. The third member of the triplet, *L. monocytogenes*, was selected as it shares similar morphology and physiology with *B. subtilis *and *B. anthracis*, but lacks the ability to form spores. In addition, *B. anthracis *and *L. monocytogenes *are pathogenic species, while *B. subtilis *is non-pathogenic. Evolutionarily, the *Bacillus *and *Listeria *genera are estimated to have separated more than 1 billion years ago [44]. Analysis of the biclusters obtained as a result of the procedure revealed several gene groups of interest and led us to formulate new hypotheses about the biology of these organisms. Specifically, we were able to detect a temporal difference between the two *Bacillus *species in the expression of a group of metabolic genes involved in spore formation. Furthermore, the unexpected identification of a bicluster for genes required for flagellum formation in *B. anthracis *prompted us to re-examine the capacity for flagellar-based motility in that species.

## Results

In this section we provide a description and genome-wide benchmarking of the multispecies integrative biclustering method (or FD-MSCM for full-data multi-species cMonkey). We compare our method to the original single-species cMonkey algorithm, a simple k-means clustering method that has been adapted to multi-species analysis and to several other single- and multi-species biclustering algorithms. We will refer only to analysis of pairs of organisms here and focus primarily on the *B. subtilis-B. anthracis *pair. We note that the method scales linearly with the number of species being analyzed and can be extended to larger numbers of organisms. The difficulties in validating biclustering performance and the need to compare the algorithm to primarily single species methods required that we initially limit the scope of this work to the simpler pairwise case. Lastly, we include examples of biologically significant biclusters retrieved by the method.

**Figure 1:**
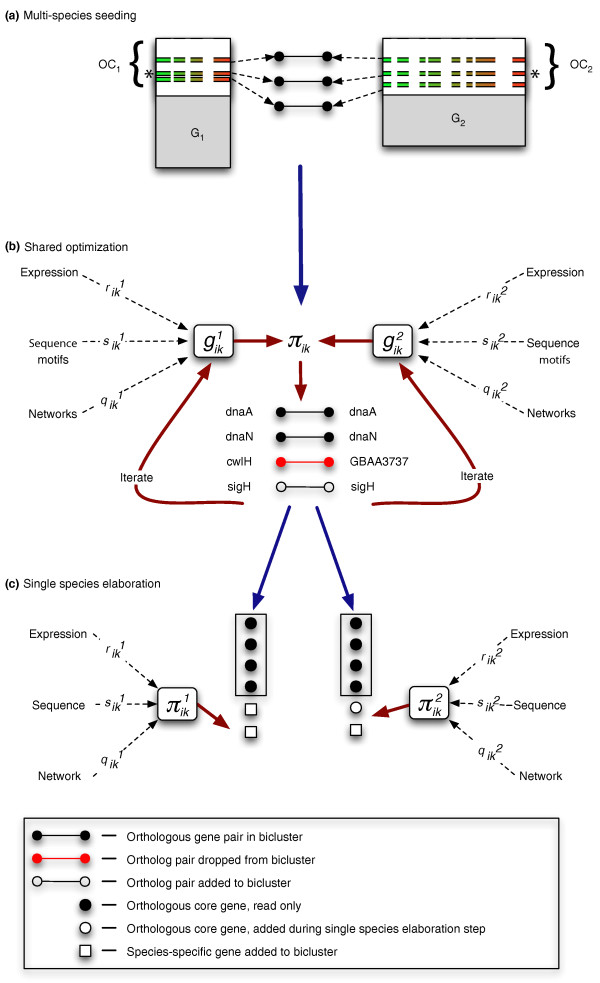
**Schematic overview of the multiple-species method**. **(a) **Shared-space bicluster seeds are generated by calculating the pairwise correlation of the gene pairs to a randomly selected gene pair. **(b) **The shared-space multi-species optimization, where orthologous gene pairs are iteratively added or dropped from the bicluster according to the multi-species multi-data score. **(c) **When completed, shared-space biclusters are separated into their respective species, and further optimized during the elaboration step. During this step the genes from the original shared-space bicluster are prevented from being dropped, as indicated by the boxes surrounding these genes (represented as black circles). OC, orthologous core (the set of actively expressed orthologous genes shared between a group of organisms on which we run our multi-species biclustering).

Our method is composed of two sequential phases (Figure 1): an initial step where conserved cores are learned in a integrated multiple-species fashion and a later step where species-specific features are added to the conserved core (called the elaboration step). The algorithm takes as input a matrix of normalized expression data for each organism (where each organism's data matrix may be normalized separately), upstream sequences for all genes, and one or more networks for each organism (in this case we used metabolic and signaling pathways from the Kyoto Encyclopedia of Genes and Genomes (KEGG), predicted co-membership in an operon and phylogenetic profile networks). The experimental datasets collected for each organism are described fully in Additional file 1 (Tables S1 and S2 in Additional file 1).

The method begins by randomly selecting a single orthologous pair (for example, *dnaA*) around which to build a seed bicluster. For the randomly selected orthologous pair, conditions are chosen in each organism's expression matrix where the orthologous gene from that organism is most significantly differentially expressed. The semi-random seed is completed by adding the five to ten most correlated orthologous pairs (for example, *dnaN*) to the randomly selected seed pair (over the conditions defined in each species). This heuristic seeding is required as most of the MSCM score terms demand that a bicluster have three or more genes in each organism to compute the scores required for further iterations. Once seeded, orthologous gene pairs are then iteratively added to (for example, *sigH*) or dropped from (for example, *cwlH*) the growing bicluster using the multi-data/multi-species score until no improvements can be made (convergence). After a bicluster converges, new biclusters are seeded and built from additional random seeds until no significant biclusters can be found or a maximum number of biclusters is reached.

Biclusters are generated sequentially and the number of biclusters to be optimized is chosen by the user. Considering that initially optimized biclusters will be unaffected by later biclusters, the number of biclusters is set higher than the expected number of true co-regulated modules. For each of the three possible species pairs, we generated 150 biclusters in the shared (multi-species) data-space that were then elaborated in the single-species data-space. Thus, each bicluster contains a conserved core (orthologous pairs that were added based on the entire integrated dataset), and 0 or more genes that were added during the elaboration step (performed separately for each organism, based on each single species dataset). A complete specification of the method is given in the Materials and methods section.

### Genome-wide assessment of multi-species biclustering performance

To validate MSCM, we compared it to several multi-species and single-species methods (Table 1; Table S3 in Additional file 1). Among the single-species methods, we included the single-species version of cMonkey (SSCM; which was previously shown to be competitive with other biclustering methods [20]) as well as two recent single-species biclustering methods, QUBIC (QUalitative BIClustering algorithm) [45] and Coalesce [22] (COAL). In addition, we compared our method to a multi-species version of the biclustering Iterative Signature Algorithm (MSISA) [13], and two multi-species clustering methods, a simple multi-species k-means algorithm (MSKM) [46] and a balanced multi-species k-means clustering method (BMSKM). We constructed the BMSKM version to balance the disproportionate size of expression datasets between the two species and thereby perform a more meaningful comparison to MSCM. We refer to the results as 'shared' (SH) if we restrict our analysis to orthologous pairs between the two species and 'elaborated' (EL) if a second step is used to add species-specific genes, that is, MSCM-EL. When possible, we evaluate both SH and EL results. In order to remain consistent with the MSISA nomenclature [13], we also use the terms 'purified' (MSISA-P) and 'refined' (MSISA-R), as these terms were used in the original work describing these methods. Descriptions of the multi-species methods can be found in the Materials and methods section. When evaluating integrative methods that take into account more than just expression data (FD: full data) we also compare to expression-only (EO) runs of each method. Our evaluation of the various methods is based on two criteria: the ability to detect statistically significant modules; and more importantly to this work, the ability to identify conserved modules. We show that MSCM produces biclusters that are a good balance of coverage, functional significance, and conservation, suggesting that the biclusters obtained by this procedure are of greater biological significance.

**Table 1:** Key to abbreviations used for methods tested

	Expression only		Full data	
		
	Shared space	Full genome (elaboration)	Shared space	Full genome (elaboration)
Multi-species				
cMonkey	EO-MSCM-SH	EO-MSCM-EL	FD-MSCM-SH	FD-MSCM-EL
ISA	MSISA-P	MSISA-R	NA	NA
k-means	MSKM-SH	MSKM-EL	NA	NA
(Balanced) k-means	BMSKM-SH	BMSKM-EL	NA	NA
				
Single-species				
cMonkey	EO-SSCM		FD-SSCM	
Coalesce	EO-COAL		FD-COAL	
Qubic	QUBIC		NA	

Tested methods are shown organized by main method (multi-species or single-species), data types used, and whether the analysis was performed over the full genome or restricted to only genes with orthologs across the species analyzed. ISA, Iterative Signature Algorithm; EO, expression only; MSCM, multi-species cMonkey; SH, shared biclusters; MSISA, multi-species ISA; P, purified biclusters; MSKM, multi-species k-means; BMSKM, balanced multi-species k-means; EL, elaborated biclusters; R, refined biclusters, applies only to the ISA algorithm (MSISA-R); FD, full data; SSCM, single-species cMonkey; COAL, Coalesce biclustering method; QUBIC, QUalitative BIClustering algorithm.

#### Using multiple metrics for validating multi-species biclustering

Validation and comparison of clustering methods remains a difficult problem [20,47]. There is, as of yet, no 'solved' organism (that is, an organism whose full regulatory network is known and experimentally validated) that can be used as a benchmark. Artificial datasets are also of limited value due to the complexity of generating reasonable synthetic datasets (one would have to generate sequences, expression data and networks, and make assumptions about the evolution of these data-types). In the face of these challenges, several criteria for judging the biological significance of gene clusters have been implemented. We will focus on five metric classes: 1) bicluster coherence; 2) functional enrichment; 3) coverage; 4) overlap between biclusters; and 5) conservation. We evaluate bicluster coherence with five metrics that gauge the support of the three data types cMonkey integrates, described further below and in Additional file 1. We also assess the number of biclusters that have a significant enrichment, considering that enrichment metrics imply that co-functional and interacting genes (by protein-protein or regulatory interaction) should have a higher probability of clustering. Expression matrix coverage and overlap between biclusters were calculated as the percentage of data-matrix elements that can be in one or more biclusters (as opposed to just genes). Gene-wise comparisons can be found in Additional file 1.

The last metric we consider, unique to multi-species datasets, is the conservation of (bi)clustered genes between the two species. Although we cannot know *a priori *what percentage of co-regulated genes will be preserved, we can state for two closely related organisms that: if two biclustering methods are equivalent (according to all other metrics), then the more conserved method is likely to be of higher biological significance; and the conserved score between biclustering methods should be well separated from a random background, but still lower than 1. In addition, more distantly related organisms should have less conserved co-regulation. By strictly enforcing a perfect conservation between the species, the two k-means variants (BMSKM and MSKM) are good examples of methods that over-estimate the degree of conservation between two species.

**Figure 2:**
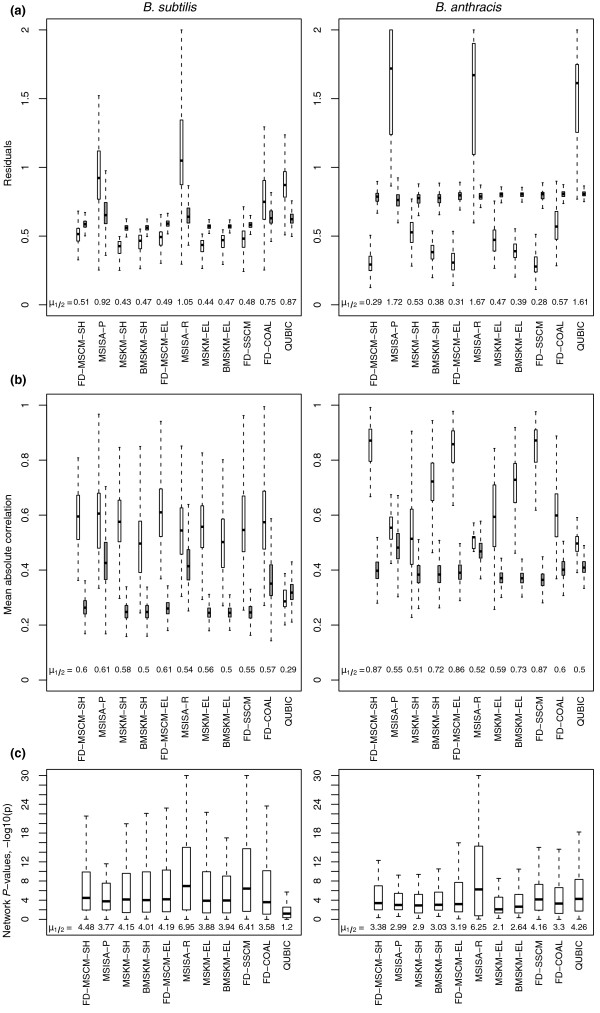
**Comparing the distribution of expression and network coherence for single- and multi-species methods for the *B. subtilis*-*B. anthracis *pairing**. A comparison of the expression and network coherence for the different MS and SS methods. For brevity, we present here only the results from full data methods (FD) from the *B. subtilis*-*B. anthracis *pairing (the results for the other pairings and expression only (EO) methods can be found in Additional file 1). Abbreviations are given for each method; a key to these abbreviations can be found in Table 1. Across the three comparisons, no method outperformed all other methods as judged by all three metrics, with the MSCM results performing competitively with the others. **(a) **The distributions of the residual values from each method for the pairing of *B. subtilis *and *B. anthracis*. We also show, next to each distribution (in gray), the residuals from randomly shuffled (bi)clusters that match the size distribution for each method with *n *= 1000 for the number of copies of the original set of (bi)clusters (same number of genes, conditions and (bi)clusters). Most methods tested were significantly better than random for both organisms; the exceptions being MSISA, QUBIC, and Coalesce (COAL). In addition, this plot illustrates the tendency of MSKM to allow an organism with a considerably larger expression dataset to dominate the analysis. **(b) **The distributions of the average absolute correlation from each method for the pairing of *B. subtilis *and *B. anthracis *are displayed to allow comparison between methods that identify inversely correlated biclusters (MSISA, QUBIC) and those that do not. As in (a), we also display the results from a randomly shuffled distribution next to each method in gray (*n *= 1000). In all cases, with the exception of QUBIC for *B. subtilis*, the method was significantly higher than random. **(c) **The distributions of the association *P*-values (-log10) from each method compared.

**Figure 3:**
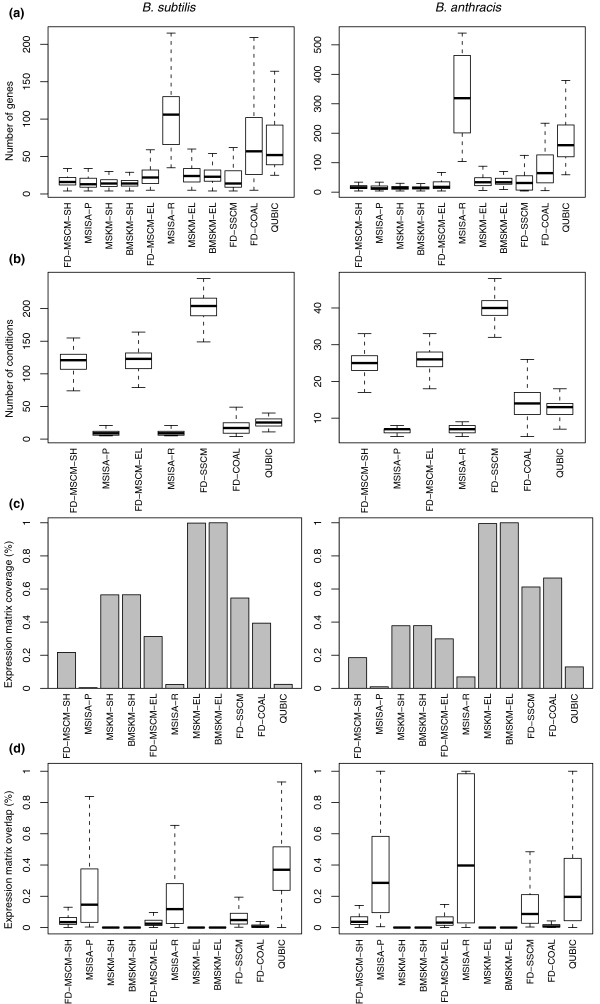
**Comparison of the size, coverage and overlap for single and multi-species methods for the *B. subtilis*-*B. anthracis *pairing (full data results only, where applicable)**. For brevity, we present here only the results from full data methods (FD) from the *B. subtilis-B. anthracis *pairing (results for the other pairings and EO methods can be found in Additional file 1). **(a) **The distribution of the number of genes in the (bi)clusters from the different methods. There is a consistent increase in the median size between the shared and elaboration steps (this is most extreme in the case of the MSISA method). For both organisms, Coalesce (COAL) and QUBIC produced the next largest biclusters, in terms of the number of genes. **(b) **The distribution of the number of conditions in the biclusters from the different biclustering methods only. We do not show this for the MSKM and BMSKM results as these methods use all conditions. For both organisms, the MS/SS cMonkey methods produced the biclusters with the most conditions. The MSISA method produced the biclusters with the least number of conditions. **(c) **The coverage of the total expression data matrix by the (bi)clusters from the different methods is displayed. The elaborated results of the MSKM and BMSKM methods achieve perfect coverage, by definition. The MSISA and QUBIC biclusters had the smallest coverage of any of the methods, while the Coalesce biclusters achieved coverages comparable with the SSCM biclusters. **(d) **The distribution of all pairwise, non-zero overlaps between the (bi)clusters from the different methods; overlap in terms of the overlap of expression matrix elements, rather than genes. By definition, the MSKM and BMSKM clusters have no overlap, while the MSISA and QUBIC biclusters had the greatest. Of the biclustering methods, Coalesce had the least overlap. Coalesce identifies more distinct biclusters with greater numbers of genes, but fewer conditions; and the SS/MS cMonkey methods identify biclusters that are slightly more overlapped than does Coalesce, with fewer genes, but covering more conditions.

Figures 2 and 3 and Table 2 present this multiple-metric comparison; Additional file 1 contains additional details and associated methods supporting these comparisons as well as this multi-metric comparison performed for the other two organism pairings. Given the above metrics and evolutionary considerations, our assessment of methods attempts to balance the five metric classes above:

**Table 2:** Summary of evaluation criteria for the single and multi-species methods for the *B. subtilis*-*B. anthracis *pairing

									GO		KEGG	
										
	Conservation score	Mean correlation: absolute value	Mean net *P*-value (-log10)	Mean number of genes	Mean number of conditions	Number of biclusters	Coverage element-wise	Mean overlap element-wise	Percent (bi)clusters enriched (*P *< 0.01)	Number of unique enriched terms	Percent (bi)clusters enriched (*P *< 0.01)	Number of unique enriched pathways
EO MSCM-SH	1	0.52 (0.69)	8.21 (6.45)	16.78 (16.78)	125.74 (25.86)	148 (148)	18.69% (15.73%)	4.76% (5.20%)	33.78% (37.16%)	378 (338)	4.05% (6.76%)	10 (16)
**FD MSCM-SH**	**1**	**0.59 (0.85)**	**9.10 (8.57)**	**21.82 (21.82)**	**116.97 (24.87)**	**150 (150)**	**21.71% (18.53%)**	**5.33% (5.93%)**	**51.33% (51.33%)**	**575 (500)**	**12.67% (12.67%)**	**24 (28)**
ISA-P	1	0.60 (0.56)	5.92 (5.63)	16.90 (16.90)	10.22 (6.85)	41 (41)	0.41% (0.95%)	22.24% (34.64%)	53.66% (75.61%)	160 (164)	19.51% (19.51%)	12 (15)
MSKM-SH	1	0.58 (0.52)	11.49 (11.62)	14.99 (14.99)	314 (51)	148 (148)	56.49% (37.83%)	0% (0%)	50.68% (39.19%)	617 (559)	14.19% (14.86%)	22 (25)
**BMSKM-SH**	**1**	**0.49 (0.72)**	**9.89 (12.19)**	**15.00 (15.00)**	**314 (51)**	**148 (148)**	**56.52% (37.85%)**	**0% (0%)**	**50.00% (48.65%)**	**658 (578)**	**16.89% (15.54%)**	**29 (34)**
EO MSCM-EL	0.907	0.54 (0.69)	7.41 (6.35)	22.74 (23.60)	129.69 (27.07)	148 (148)	25.03% (21.68%)	4.38% (5.06%)	40.54% (60.81%)	449 (485)	11.49% (10.81%)	18 (18)
**FD MSCM-EL**	**0.852**	**0.61 (0.84)**	**7.64 (8.65)**	**33.75 (34.63)**	**119.87 (26.26)**	**150 (150)**	**31.29% (29.90%)**	**4.00% (5.72%)**	**56.00% (72.67%)**	**649 (664)**	**15.33% (21.33%)**	**30 (37)**
ISA-R	0.093	0.55 (0.51)	3.54 (8.87)	106.05 (335.71)	10.22 (6.93)	41 (41)	2.36% (6.90%)	18.34% (46.28%)	95.12% (100.00%)	287 (235)	24.39% (58.54%)	10 (20)
MSKM-EL	0.956	0.56 (0.58)	10.27 (6.65)	26.49 (39.44)	314 (51)	148 (148)	99.80% (99.52%)	0% (0%)	63.51% (75.68%)	732 (675)	14.86% (12.16%)	31 (30)
**BMSKM-EL**	**0.959**	**0.50 (0.71)**	**8.58 (7.93)**	**26.54 (39.63)**	**314 (51)**	**148 (148)**	**100% (100%)**	**0% (0%)**	**52.70% (81.76%)**	**743 (710)**	**15.54% (11.49%)**	**35 (25)**
EO SSCM	0.098	0.70 (0.91)	8.58 (7.43)	26.19 (34.11)	193.40 (38.66)	161 (210)	39.48% (46.81%)	9.44% (14.10%)	42.24% (66.19%)	499 (629)	10.56% (17.62%)	19 (29)
**FD SSCM**	**0.124**	**0.56 (0.82)**	**10.14 (7.31)**	**23.06 (40.65)**	**200.76 (39.81)**	**295 (315)**	**54.55% (61.24%)**	**7.53% (15.46%)**	**50.51% (61.59%)**	**746 (712)**	**11.53% (9.52%)**	**32 (31)**
EO COAL	0.107	0.58 (0.64)	5.21 (5.06)	86.65 (115.71)	20.09 (13.13)	300 (158)	40.21% (66.40%)	1.94% (2.12%)	63.67% (76.58%)	744 (659)	17.67% (9.49%)	32 (24)
**FD COAL**	**0.101**	**0.59 (0.62)**	**5.27 (5.69)**	**88.16 (131.12)**	**20.24 (14.24)**	**287 (136)**	**39.39% (66.63%)**	**2.06% (2.16%)**	**64.81% (80.88%)**	**776 (686)**	**16.03% (14.71%)**	**24 (24)**
QUBIC	0.054	0.36 (0.49)	1.38 (5.90)	71.59 (188.25)	25.45 (12.63)	150 (150)	2.43% (12.95%)	38.34% (26.49%)	43.33% (88.67%)	227 (331)	3.33% (14.67%)	5 (13)

We compare several metrics of bicluster conservation, coverage, and functional enrichment. In all cases metrics are averaged over all biclusters produced by that method for each species. In each column, the results for *B. subtilis *are listed first, with those for *B. anthracis *listed in parentheses. 'Conservation score' provides an estimate of the conservation identified between biclusters of the different organisms as defined in the methods. 'Mean correlation' measures the coherence of the biclusters given the expression. 'Mean net *P*-value' measures the enrichment of network edges within biclusters. 'Mean number of genes', 'Mean number of conditions' and 'Number of biclusters' summarize the size distributions of the (bi)clusters identified. 'Coverage element-wise' is the percentage of the total expression data that is found in one or more (bi)cluster. 'Mean overlap element-wise' estimates the redundancy of the (bi)clusters; overlap is calculated as the mean of the maximum percentage of overlap for each bicluster in the full set of biclusters for a given method. 'Percent (bi)clusters enriched (*P *< 0.01)' for GO and KEGG provides an estimate of the functional significance of the (bi)clusters identified. 'Number of unique enriched terms' for GO and 'Number of unique enriched pathways' for KEGG are the number of unique terms/pathways across all biclusters for that method; this number of enriched terms/pathwaus provides an estimate of the redundancy of the biological functions enriched in one or more biclusters across the full set of biclusters for any given method. Further explanations of these metrics can be found within the text and Additional file 1. GO, Gene Ontology; KEGG, Kyoto Encyclopedia of Genes and Genomes; ISA, Iterative Signature Algorithm; EO, expression only; MSCM, multi-species cMonkey; SH, shared biclusters; MSISA, multi-species ISA; P, purified biclusters; MSKM, multi-species k-means; BMSKM, balanced multi-species k-means; EL, elaborated biclusters; R, refined biclusters (applies only to the ISA algorithm (MSISA-R)); FD, full data; SSCM, single-species cMonkey; COAL, Coalesce biclustering method; QUBIC, QUalitative BIClustering algorithm.

$$\begin{array}{l} \text{bicluster}-\text{quality }=\\ \;\left[\text{data support}:\;\left(\text{1}\right)\text{ coherence},\;\left(\text{2}\right)\text{ functional enrichment}\right]\times \\ \;\left[\text{completeness}:\;\left(\text{3}\right)\text{ coverage},\;\left(\text{4}\right)\text{ overlap}\right]\times \\ \;\left[\text{conservation}:\;\left(\text{5}\right)\text{ conservation score}\right] \end{array}$$

#### Comparing the degree of conserved co-regulation detected by each method

A bicluster is considered to be perfectly conserved when all of the orthologous genes from that bicluster are found in a single bicluster in the related species. We evaluated the ability of all the tested methods to identify conserved biclusters using a metric similar to the F-statistic [48], which gauges the degree of recovery between a bicluster in one species with that of the closest bicluster in the other species. For the multi-species methods, we calculated the metric using the shared bicluster for one organism with its bicluster counterpart in the other. Details of the procedure can be found in the Materials and methods section.

Using this simple measure of conservation, we evaluated the results from all the multi-species (MS) methods with those from several single-species (SS) methods (Table 2 displays the results for the *B. subtilis-B. anthracis *pairing; see Tables S2 and S3 in Additional file 1 for the others). With the exception of MSISA-R, the MS methods displayed a far greater degree of conservation than any of the SS methods, with the shared (SH) steps (and the equivalent MSISA-P step) having perfect conservation, and the elaboration (EL) steps having conservation scores >0.85. As they overestimate the conservation between the two species by assuming perfect conservation for all orthologous pairs during their shared steps, both B/MSKM-EL results display a greater degree of conservation than the MSCM-EL results. In contrast, none of the SS methods possessed a conservation score >0.125 (although it is likely that this score underestimates the degree of conserved co-regulation they detect as the conservation scores for many of them were still significantly greater than random (PHW, unpublished results)).

The low conservation score for closely related organisms obtained when running SS methods on individual datasets was surprising. We expected that the truly conserved co-regulated gene groups would be detected individually by the SS methods and thus contribute to higher conservation scores. We attribute the low conservation scores in part to biologically relevant differences in co-regulation, but also to the fact that SS biclusters are supported by smaller datasets that contain systematic errors that likely differ between species (and thus, correctly cancel out in the multi-species analysis). Importantly, the greater conservation scores for MSCM had little or no negative impact on the other commonly used evaluation metrics we employed.

#### Coherence of biclusters, coverage and bicluster overlap

In this section we evaluate the ability of each method to simultaneously find coherent biclusters (Figure 2), cover the input dataset, and minimize the overlap between biclusters (Figure 3). We assess bicluster expression coherence by: 1) residual, the mean error when the average expression value over the bicluster is used to predict gene expression levels (Figures S1, S2, and S3 in Additional file 1); and 2) mean correlation, the average pairwise correlation between all (bi)cluster members, taking the absolute value of the correlation to allow unbiased comparison between methods that identify inversely correlated patterns (QUBIC and MSISA) and those that do not (Figures S4, S5, and S6 in Additional file 1). These two measures are dependent on the number of conditions and rows in the bicluster and overall coverage of the data matrix. Therefore, in all cases we compare co-expression values to a randomized background generated specifically for that biclustering (see Materials and methods). We assess bicluster network coherence by: 3) association network *P*-values, a measure of the significance of the subnetworks within biclusters compared to the full network (Figures S7, S8, and S9 in Additional file 1). We assess bicluster sequence coherence by 4) upstream motif E-values, a measure of the quality/significance of the upstream binding site motifs detected for each bicluster (Figures S10, S11, and S12 in Additional file 1); and 5) sequence *P*-values, representing the preferential partitioning of the discovered motifs to genes in the bicluster over the remainder of the genome (Figures S13, S14, and S15 in Additional file 1). We direct the reader to Additional file 1 and prior work [20] for detailed descriptions of these metrics, along with the individual comparisons. Note, in the case of the non-integrative methods, sequence and network based metrics or scores were calculated *post hoc *for the (bi)clusters they produced.

We found that for all five coherence metrics, FD-MSCM performed as well or better than the other methods (Tables S4, S5, S6, S7, and S8 in Additional file 1); specifically in 71 of the 92 individual comparisons of the expression residual distributions, in all 92 of the mean correlation comparisons, in 77 of the 92 comparisons for the network association *P*-values, in 69 of the 92 comparisons for the motif E-values, and in 72 of the 92 comparisons for the sequence *P*-values. Note, the large number of comparisons (92) results from the fact that we have three organism pairings and that for each run we must separate the multi-species run into a set of biclusters for each species to calculate these validation metrics (thus, each species pair results in twice the number). Similar comparisons with EO-MSCM (Tables S9, S10, S11, S12, and S13 in Additional file 1) indicated that for four of the five metrics, it did as well or better than the other methods tested - the sole exception being motif E-values.

In the comparisons with the random permutation results for the expression metrics (Tables S14 and 15 in Additional file 1), expression residuals for the MSCM and SSCM were all significantly better than random distributions generated for each method (differing cluster and bicluster sizes required a separate calculation of the random background for these expression coherence metrics for each method and for each data-set), for all organisms and pairing combinations, as were those for the two MS k-means variants (B/MSKM). In contrast, the residuals from both QUBIC and the two MSISA steps were all significantly worse than random; while the residuals from COAL were significantly better for *B. anthracis*, but somewhat worse for *B. subtilis *and *L. monocytogenes*. However, when considering the mean correlation results, nearly all methods were better than random, the sole exception to this being the MSISA results for *L. monocytogenes *in the pairing with *B. subtilis*.

Regardless of the pairing, both QUBIC and MSISA produced biclusters with the most genes (Figures S16, S17, and S18 in Additional file 1) and fewest conditions (Figures S19, S20, and S21 in Additional file 1), while also simultaneously having the least coverage (Figures S22, S23, S24, S25, S26, and S27 in Additional file 1) and most redundant set of biclusters (Figures S28, S29, S30, S31, S32, and S33 in Additional file 1). We exclude QUBIC and MSISA from further consideration for this reason. By contrast, the two B/MSKM variants display complete coverage of the data space. Although it is not possible to say what the optimal value for coverage should be, it is clear that numbers approaching 100% include several false positives (with respect to conserved co-regulation), as one cannot reasonably expect every gene to be a member of a conserved regulatory module, and that methods that cover 2% or less of the data space are likely missing the majority of conserved co-regulation. We note that the coverage of both the genome and expression dataset for MSCM is considerably smaller in comparison to SSCM and COAL. This is not unexpected because the search spaces are constrained by the orthologous core, with the search space of the elaboration step indirectly constrained by results of the shared step. The SS methods typically had better coverage, reflecting that a significant fraction of co-expressed gene groups are not conserved across the species investigated.

#### Estimating functional coherence via enrichment of function annotations

**Figure 4:**
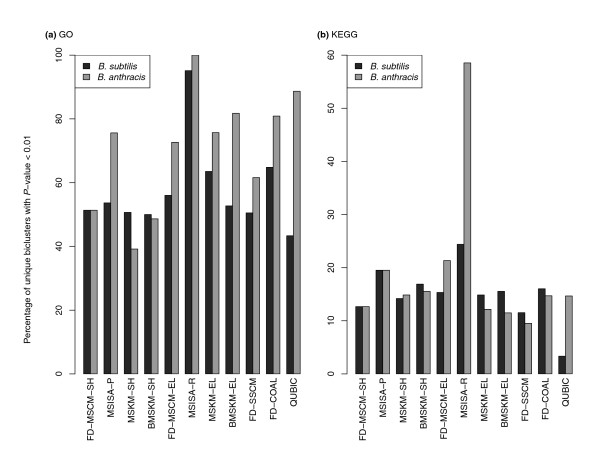
**Comparison of the fraction of biclusters with significant GO and KEGG annotation enrichments for the single and multi-species methods for the *B. subtilis*-*B. anthracis *pairing**. **(a) **GO terms. For all multi-species methods there is a consistent increase from the shared to elaboration step, with the percentage of elaborated biclusters with significant GO term enrichments consistently greater than those from the single species optimization. **(b) **KEGG pathways. For both of the multi-species biclustering methods (MSCM and MSISA), there is a consistent increase in percentage from the shared to elaborated optimizations, similar to the GO term enrichments, with a similarly large increase for the refined MSISA biclusters for *B. anthracis*. The two k-means clustering variants showed either negligible increase or even a decrease between the shared and elaboration steps.

We compared the percentages of biclusters that were significantly enriched (*P*-value <0.01) for both Gene Ontology (GO) terms and co-presence in KEGG pathways. Again, we limit the discussion of these below to the pairing of *B. subtilis *with *B. anthracis *(Figure 4; Figure S34 in Additional file 1), though similar patterns were observed with the other pairings as well (Figure S35 and S36 in Additional file 1). For all of the multi-species methods, there was a consistent increase between the shared and elaboration optimizations, indicating the importance of adding species-specific genes to conserved co-regulated cores. For example, for FD-MSCM, the percentage of biclusters with GO term enrichments increases from 51.3% to 56.0% for *B. subtilis *(from 51.3% to 72.7% for *B. anthracis*) between the shared and elaboration optimizations (similarly, for MSKM, the increase is from 50.7% to 63.5% for *B. subtilis*; 39.2% to 75.7% for *B. anthracis*). The large increase observed for the MSISA results (53.7% to 95.1% for *B. subtilis*; 75.7% to 100% for *B. anthracis*) is a reflection of the small number of large and highly redundant biclusters it identifies. When a filter is applied that allows a GO term to be enriched for only a single bicluster, these percentages drop considerably (70.1% for *B. subtilis*, 39% for *B. anthracis*, MSISA-R biclusters).

The percentage of biclusters with enriched KEGG pathways is much higher for the MS methods than for SSCM. For example, the percentage of the FD-MSCM-EL for *B. subtilis *was enriched 15.3%, while the percentage of the FD-SSCM results was 11.5% (21.3% versus 9.4% for *B. anthracis*). We observed a pattern similar to what was observed with the GO terms, in the sense that there was also a consistent increase between the shared and elaboration runs. For example, with FD-MSCM, the percentages increase from 12.7% to 15.3% for *B. subtilis *(12.7% to 21.3% for *B. anthracis*).

We also compared the performance of different species-species pairings (see Additional file 1 for data). We observed that for both of the pairings involving *B. subtilis*, the residuals of the clusters generated by MSKM were significantly better for the *B. subtilis *clusters, but significantly worse for the other organisms. As the *B. subtilis *expression dataset contained nearly six times more conditions than the other organisms, a key limitation of this and other similarly constructed methods is the dominance of a single species in the results. This effect was muted by the 'balancing' procedure (that is, the BMSKM method). However, while the performance for the organism with the smaller dataset improved, the performance for the organism with the larger dataset decreased significantly. A similar effect was observed with MSISA.

Finally, we noticed that there was a consistent increase in the quality of the motifs associated with the biclusters returned by the elaboration step of both MS methods. One possible explanation for this behavior is simply algorithmic, namely, that MEME [49], the motif inference tool we use, is able to infer more significant motifs from the larger pool of sequences accessible to the elaborated biclusters. Another reason may be that this behavior indicates a significant species-specific change at the level of binding sites, even when the gene membership in a module is conserved (an example of this is provided below). Our methodology for modeling and detecting binding sites as part of the multi-species procedure can likely be improved substantially and should prove a promising area for future work.

### Examples of conserved modules detected by the multi-species analysis: application to *B. anthracis *and *B. subtilis*

To illustrate the strength of our method's ability to identify conserved modules and also to highlight species specific elaboration of these modules, we focus on two processes - endospore formation (sporulation) and flagellum synthesis. In the case of sporulation, both *B. anthracis *and *B. subtilis *can sporulate, while *L. monocytogenes *cannot [50]. Similarly, both *B. subtilis *[51] and *L. monocytogenes *[52] possess flagella and are motile, while *B. anthracis *is a non-motile species [53].

#### Biclusters involved in sporulation shared between *B. subtilis *and *B. anthracis*

Sporulation is a cellular differentiation process that *B. subtilis *and *B. anthracis *undergo as a response to resource depletion [39-42]. Sporulating cells divide asymmetrically near one cell pole to produce a smaller cell, the forespore and a larger cell, the mother cell. The forespore will differentiate into a highly resistant dormant cell type called an endospore (hereafter: spore). The mature spore is surrounded by two membranes and a thick proteinaceous layer (the coat). A modified peptidoglycan layer (the cortex) is synthesized in the intermembrane space.

As expected, the multi-species method identified several sporulation modules from the *B. subtilis-B. anthracis *pairing and no sporulation modules from the pairings involving *L. monocytogenes*. Here, we focus on three biclusters (32, 82 and 84), whose orthologous cores contained largely non-overlapping sets of genes. Analysis of the gene content indicated that each bicluster was involved in distinct biological functions during sporulation. Bicluster 84 primarily contains genes involved in metabolic functions (Figures S43 and S44 in Additional file 1). Bicluster 32 contains genes involved in activation of late sporulation σ factors (σ^G ^and σ^K^) and cortex synthesis (Figures S39 and S40 in Additional file 1). Bicluster 82 contained a majority of spore coat genes (Figures S41 and S42 in Additional file 1).

Most of the genes found in those three biclusters had been previously identified as members of the mother cell transcriptome in *B. subtilis *[54-56]. Specifically, 16 of the 26 core genes from the metabolism bicluster, 36 of the 38 core genes from the cortex bicluster and the 24 core genes from the coat bicluster are expressed under the control of the early mother-cell σ factor, σ^E^. Nevertheless, the metabolism bicluster contained five previously unrecognized sporulation genes (*ykwC*, *ctaC*, *ctaD*, *ctaE *and *ctaF*). The *ykwC *gene encodes a protein from the 3-hydroxyisobutyrate dehydrogenase family, which is consistent with the function of several other genes found in that bicluster (for example, the *mmg *and *yngJ *operons [57]). The *cta *operon encodes the four subunits of cytochrome C oxidase. These genes are subject to catabolite repression by glucose; therefore, their expression is prevented during exponential growth in glucose-containing medium [58]. During sporulation initiation, the *cta *operon is activated by Spo0A~P (the master regulator of sporulation) [59]. The neighboring *ctaA *gene, which is transcribed in the divergent direction, has been previously reported to be controlled by RNA polymerase containing σ^E ^[60]. Examination of the *ctaC *upstream region reveals a possible σ^E ^binding site with a reasonable match to the consensus (Figure S37 in Additional file 1). Protracted expression of these genes (first under the control of Spo0A~P in the pre-divisional cell and then of σ^E ^after asymmetric division) is consistent with the conclusions of previous studies indicating that tricarboxylic acid cycle function (and by extension the electron transport chain) is required during sporulation [61-63].

**Figure 5:**
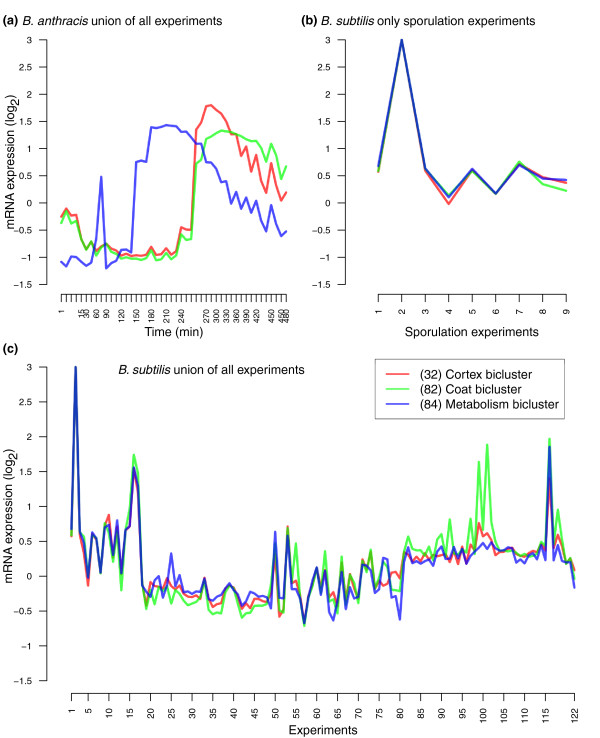
**Expression profiles of three partially conserved sporulation biclusters, identified by the multi-species analysis of *B. subtilis *and *B. anthracis***. Bicluster 84 (blue line) is composed primarily of genes involved in metabolic functions during sporulation, bicluster 82 (green line) includes primarily genes encoding spore coat proteins, and bicluster 32 (red line) contains genes involved in spore cortex formation and activation of the σ factors required for the latest stages of sporulation. **(a) **The *B. anthracis *biclusters display distinct profiles, revealing a temporal aspect not present in the *B. subtilis *dataset. The *B. subtilis *biclusters all follow the same expression profile (that is, similar expression over nearly every experimental condition included in the dataset), as shown for **(b) **only sporulation experimental conditions (with abscissa corresponding to: 1) hour 2 *sigF*; 2) hour 2.5 *sigE*; 3) hour 3.5 *gerR*; 4) hour 3.5 *spoIIID*; 5) hour 4 *sigG*; 6) hour 4.5 *sigK*; 7) hour 5 *spoVT*; 8) hour 5.5 *gerE*; 9) hour 6.5 *gerE*) and **(c) **all experimental conditions within the three biclusters.

Unexpectedly, we uncovered a key species-specific difference in the timing of expression of one conserved sporulation module (the metabolism bicluster). The expression data we used for *B. anthracis *is a time series transcriptional profile of the entire life-cycle, from germination through sporulation [64]. Expression of genes from the metabolism bicluster reaches its maximal level at t = 180 minutes (Figure 5a), 2 hours before the expression peak of genes from the cortex and coat biclusters at t = 270 minutes. No such temporal difference exists between the metabolism bicluster and the other two biclusters during *B. subtilis *sporulation (Figure 5b) because most of these genes are directly controlled by σ^E ^in *B. subtilis*. We propose that the observed timing difference between the two species is caused by transcriptional re-wiring. In support of this interpretation, examination of the regulatory sequence upstream of the genes from the metabolism bicluster did not reveal obvious σ^E ^binding sites in *B. anthracis*, while putative σ^E ^promoters were present upstream of genes from the cortex and coat biclusters in both species. Thus, in *B. anthracis*, the metabolism bicluster may be under the control of a transcription factor active prior to σ^E ^activation. This is further supported by the fact that in *B. anthracis sigE *itself is expressed after the expression peak of the metabolism bicluster [64].

#### Flagellar assembly biclusters shared between *B. subtilis*, *B. anthracis *and *L. monocytogenes*

**Figure 6:**
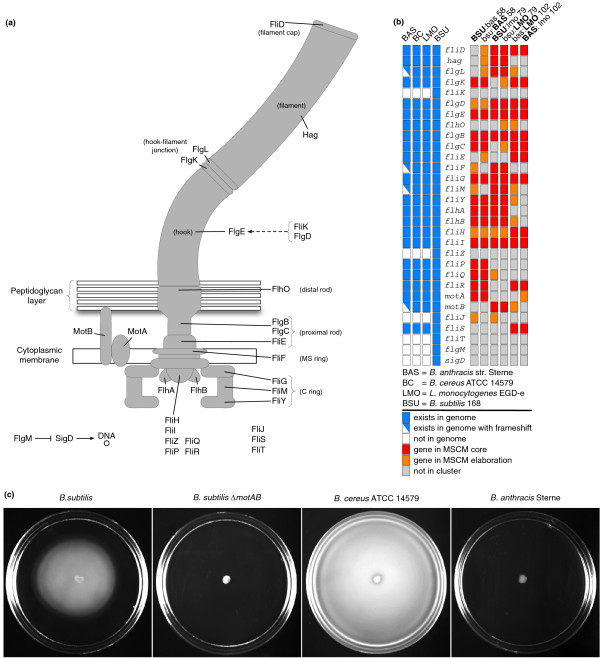
**Conserved motility modules active in all three organisms and motility assays**. **(a) **We show a schematic of the flagellar apparatus for *B. subtilis *showing the location of 26 flagellar proteins, two motor proteins (MotA and MotB) and two transcriptional regulators (FlgM and SigD) (using gene names from *B. subtilis*). **(b) **The left panel shows the presence (blue)/absence (white) of the corresponding genes in the genomes of *B. anthracis *Sterne (BAS), *B. cereus *ATCC 14579 (BC), *L. monocytogenes *EGD-e (LMO) and *B. subtilis *168 (BSU). In *B. anthracis *Sterne, *motB*, *fliM*, *fliF*, and *flgL *are represented by two colors indicating a gene coding for a truncated protein due to a frameshift mutation that introduces a premature stop codon. The right panel shows the gene presence in the main flagellar bicluster resulting from each of the three pairwise multi-species biclusterings. Indicated are genes of the flagellar apparatus - included in the bicluster core (red), in the elaboration of the bicluster (orange), and not included in the bicluster (gray). *B. subtilis *and *L. monocytogenes *are both known to be flagellated and motile. *B. anthracis *Sterne is non-motile, but our results indicate a bicluster enriched for genes involved in flagellar biosynthesis. **(c) **Swimming motility was assayed on 0.3% agar plates for *B. cereus *ATCC 14579, *B. anthracis *Sterne, *B. subtilis *PY79, and *B. subtilis *PY79 Δ*motAB*::*tet *(strain DS219). *B. cereus *and *B. subtilis *are motile [51,68]. A deletion of *motAB *in *B. subtilis *impairs motility [69,91]. The assay shows that *B. anthracis *Sterne is not motile under the conditions tested.

Assembly of the bacterial flagellum is a well-known pathway (Figure 6a) that has been studied over a wide range of prokaryotes [65-67]. It contains approximately 25 proteins conserved across numerous species, though not all these species are motile [66]. Here we use the expression of flagellar genes as another benchmark of the multi-species method. We expected that multi-species integrative biclustering with any pairing including *B. anthracis *would be unable to recover modules enriched with flagellar genes. Nonetheless, we discovered that flagellar modules were retrieved with all possible pairings (Figures 6b; Figures S45, S46, S47, S48, S49, and S50 in Additional file 1). Furthermore, recovery was well supported by the *B. anthracis *portion of the multi-data score. This result was unexpected as it was assumed that the loss of motility would be rapidly followed by loss of coordinated expression of flagellar genes.

One simple explanation of the conservation of the *B. anthracis *motility bicluster would be that the strain is, in fact, still motile or able to recover motility through a common reversion mutation. To explore and partially rule out this possibility, we confirmed experimentally that *B. anthracis *Sterne was non-motile at 37°C by performing swimming motility assays on 0.3% agar plates (Figure 6c). We used *B. cereus *ATCC 14579 and *B. subtilis *PY79 as positive controls for swimming [51,68] and *B. subtilis *PY79 Δ*motAB*::*tet *as a negative control [69]. Even after prolonged incubation of those plates at 37°C for several days, we were unable to observe motile *B. anthracis *cells.

*B. anthracis *Sterne lacks six flagellar genes present in *B. subtilis *(*fliK*, *fliO*, *fliJ*, *fliT*, *flgM*, and *sigD*) [70]. Although most of these genes are likely to be essential for flagellum function in *B. subtilis *(Table S16 in Additional file 1), they are absent in several motile species, including *L. monocytogenes *and *B. cereus*. These genes may in fact be dispensable for motility if a different gene provides a corresponding function. For example, while σ^D ^and FlgM (the anti- σ^D ^factor) regulate flagellar gene expression in *B. subtilis*, they are not found in *L. monocytogenes*, where flagellar gene expression is regulated by the transcription factor MogR, which is absent in *B. subtilis *[52] (Table S17 in Additional file 1). We performed a BLAST search-based analysis of the presence or absence of flagellar assembly and chemotaxis genes for *L. monocytogenes *and various *Bacillus *species (Table S18 in Additional file 1). Since *B. cereus *is the closest motile relative to *B. anthracis *[71], we focused on cases where a flagellar gene was present in *B. cereus *and absent in *B. anthracis*. Specifically, BLAST searches were performed against the genomes of various *B. anthracis *strains using *B. cereus *ATCC 14579 protein sequences as a reference. In *B. anthracis *Sterne two strong hits were retrieved for MotB; each of which covered a different half of the *B. cereus *MotB sequence. Upon closer inspection, it was found that both these coding sequences derived from the same gene, which had undergone a frameshift mutation via a one base-pair deletion. The frameshift resulted in an in-frame stop codon shortly following the deletion (Figure S38 in Additional file 1). In *B. subtilis*, *motB *has been shown to be essential for motility [69] (Figure 6c).

We then examined the protein sequences of all the flagellar proteins in *B. anthracis *Sterne by performing multiple alignments with other related *Bacilli *and discovered that three additional proteins appeared truncated: FliM, FliF and FlgL. Investigation of the gene sequences for these proteins in *B. anthracis *Sterne revealed that they all contained a frameshift mutation, which resulted in the introduction of an in-frame stop codon. In *B. subtilis*, *fliM *mutations result in a non-flagellated phenotype [72], while *fliF *and *flgL *are essential for flagellar assembly in *L. monocytogenes *[73,74]. In addition, we found a similar frameshift in *cheV*, a gene required for chemotaxis in *B. subtilis*.

The presence of the frameshift mutations for these key motility genes most likely explains why *B. anthracis *Sterne is non-motile and does not readily revert back to a motile phenotype. Importantly, this observation indicates that a conserved module can persist for some time even after the loss of the associated phenotype.

## Discussion

Any attempt to detect conserved modules across multiple species data collections needs to simultaneously address the following non-trivial challenges: 1) modules may be active or coherent in subsets of the conditions for each species; 2) in most cases there is little or no correspondence between the experimental conditions and experimental designs across different species datasets; 3) the amount and quality of data available often varies dramatically across species of interest; 4) modules may not be conserved in their regulation or function; 5) conserved modules may have extensive species specific elaborations that complicate their detection; 6) in many cases, the sequence-based orthology is not a one-to-one mapping; and 7) integration of additional data types needs to be robust to the differences in the available data and annotation completeness of the species considered. In this investigation, we have introduced a new algorithm, multi-species cMonkey (MSCM), that allows us to address all of these challenges in a unified analysis. We tested six other biclustering and clustering methods in various combinations (13 clustering and biclustering formulations were tested) and found no other method capable of balancing all of these challenges. We have shown that MSCM provides better or comparable coverage, functional enrichment scores, bicluster coherence, and conservation than other tested methods, with all other methods failing in one of the main categories of assessment. Furthermore, our method effectively balances the influence of each organism, preventing organisms with more complete datasets from dominating the analysis, while also integrating other supporting data types, enabling the method to identify more biologically relevant modules and delimit the conditions over which the modules are active. The fact that the MSCM biclusters have many-fold higher conservation scores than several of the tested methods suggests that they have a higher level of biological significance than equally co-expressed (and/or equally functionally enriched) non-conserved alternative biclusters. An analysis that takes into account several validation metrics supports the idea that MSCM is the top performing method for comparative biclustering.

In the single-species setting, SSCM and other biclustering methods, particularly Coalesce, are comparable in performance (when one considers score, enrichment and coverage but not conservation). Our analysis suggests that multi-species extensions of other top performing algorithms (particularly Coalesce) will also perform well at detecting conserved modules (assuming that such extensions are possible). For all the organism pairings, there was a consistent increase in the percentage of GO and KEGG enrichments from the shared to elaboration steps of the MSCM method. This results from shared biclusters that contain enrichments that are insignificant until genes from outside of the orthologous core are added during the elaboration step. We argue that this improved functional coherence illustrates the necessity of a species-specific elaboration step in any type of multi-species analysis similar to the one described here. Future work will include development of methods for adding non-obvious homologs, and perhaps phenologs [75], to the comparative phase of our analysis.

## Conclusions

A careful examination of several of the conserved biclusters generated as part of the MSCM analysis indicates that our method can reveal important new biology. For instance, we found two cases where conserved biclusters function differently in the species analyzed. The recovery of a flagellar module in the non-motile *B. anthracis *species shows that it is possible to identify conserved modules, even in cases where phenotypic divergence suggests none should exist. In addition, a key temporal difference in the sporulation programs for *B. subtilis *and *B. anthracis *emerged that led us to propose that a rewiring event took place during the evolution of the expression of a group of metabolic genes involved in sporulation. Our biclustering approach also appears useful in generating functional hypotheses for genes that are grouped with other genes of previously established functions, considering that many of the unannotated genes contained in biclusters with GO or KEGG enrichments are well supported across six or more datasets (2 organisms × 3 or more data types). Our method also reveals new links between functions that were previously considered to be separate, such as the association of the *cta *operon and the *ykwC *gene with several other *B. subtilis *metabolism genes.

## Materials and methods

Here, we describe the main steps in the MSCM algorithm, which is implemented in the R programming language and freely available [76]. We emphasize the novel modifications to the algorithm that allow for identifying biclusters in a multi-species context; for a more detailed description of the individual cMonkey scoring function components see [20]. Methods used for global assessment and comparison of our methods to other biclustering and clustering methods, experimental validation of results, and code release as well as two simple multi-species clustering methods of our own construction (multi-species k-means and balanced multi-species k-means) are also described. A complete description of the data used for each organism is provided in Additional file 1.

### Multi-species cMonkey method overview

Briefly, the MSCM algorithm is composed of three steps (optionally four): 1) the identification of orthologous genes between closely related species; 2) an iterative, Monte Carlo optimization within the space of shared orthologs (involving pairs of orthologous genes); 3) an iterative, Monte Carlo optimization in the space of each organism's genome that elaborates the biclusters found in step 2 by adding non-orthologous genes; and 4 (optional) an application of the original, single-species method to the remainder of each organism's genome (that was not added to the conserved biclusters found in steps 2 and 3) to identify completely species-specific biclusters.

#### Algorithm overview

1) Identification of orthologous genes, 2) Identification of shared biclusters by optimizing MSCM score (orthologous gene space), 3)	Single-species elaboration of shared biclusters from step 2 (single-species full genome space), 4) Identification of non-shared biclusters (single species full genome space) (optional).

### Determining putative orthologs spanning relevant genomes (step 1)

Our analysis requires the identification of putative orthologs between each pair of organisms as input. As identification of ortholog sets between species is not a primary focus of this investigation, we rely on publicly available tools and resources to define our starting set of putative orthologs between two or more species. Dependent upon the organisms used, there may be databases that can provide these ortholog sets, such as the well-annotated list of orthologs from the Mouse Genomics Informatics database [77]. In cases where a pre-existing curated list of orthologs is unavailable, we use the InParanoid algorithm [78] as two recent benchmarks [79,80] determined it to be among the most accurate when identifying pairwise orthology. InParanoid allows for the identification of families of orthologous and paralogous genes that are shared by two genomes, rather just single pair matches (for example, as the *cotZ *gene in *B. subtilis *has two possible orthologs in *B. anthracis*, *cotZ1 *and *cotZ2*, both the *cotZ-cotZ1 *and *cotZ-cotZ2 *pairs will be considered by our algorithm). This feature of the InParanoid algorithm is useful in the context of this work as it allows for more permissive supersets of putative orthology from which we can sample using cMonkey (thus letting the data select amongst orthologous super-sets).

#### Defining the multi-species data-space

In the first phase of our algorithm, biclustering is performed on groups of orthologous genes (in this study we limit the algorithm to pairs, but the algorithm is easily extendable to larger groups). For any two genomes, *G*_*U *_and *G*_*V*_, we use *OC*_*U *_and *OC*_*V *_to refer to the portions of these genomes with one or more orthologs in the other genome, which we term the 'orthologous cores' of these genomes. Furthermore, we will use *OC*_*UV *_to refer to the list of all possible pairings of orthologs between the species, which for convenience we will refer to as 'orthologous pairs'. In the case of gene families, where genes from one genome have several putative orthologs in the other, we allow the algorithm to separately consider gene pairs for each of the possible pairwise relationships. Thus, if we have a family, *f*, that has four members in genome *U*, $OC_{U}^{f}=\left\{g_{U}^{1},\ldots g_{U}^{4}\right\}$, and three in genome *V*, $OC_{V}^{f}=\left\{g_{V}^{1},\ldots g_{V}^{3}\right\}$, this will result in 12 possible pairs for this family, that is, $OC_{UV}^{f}=\left\{g_{U}^{1}g_{V}^{1},g_{U}^{1}g_{V}^{2},\ldots ,g_{U}^{4}g_{V}^{2},g_{U}^{4}g_{V}^{3}\right\}$.

#### Seeding the biclustering

The first step in building multiple-species biclusters out of ortholog pairs, as defined above, consists of seeding a bicluster (selecting a starting subset of orthologous pairs to define as the starting bicluster). For example, this can be done via selection of a random subset of orthologous pairs as a 'seed', which is then optimized. For this study we choose a semi-random seeding. We choose a random orthologous gene pair and then 1) define the bicluster seed to be the 70% of conditions in each organism's dataset where the ortholog pair has the highest variance, and 2) add the most correlated five to ten ortholog pairs (where the correlation is calculated as the average for each gene in the ortholog pair over only the conditions in the bicluster). We refer to this simple procedure for seeding the bicluster optimization as semi-random seeding. The main motivation behind this scheme (described in Additional file 1 and [20]) is to improve the convergence rate by jump-starting the optimization, though MSCM can also be used to refine randomly generated seeds.

### Finding biclusters in the multi-species data-space (step 2)

Given a bicluster seed (semi-random, random or a seed generated via a different method) we begin the multi-species optimization by iteratively adding and dropping genes and conditions as part of a simulated annealing optimization of the multi-species integrative score. Letting *X*_*U *_and *X*_*V *_represent the expression datasets for the two genomes considered, a single-species bicluster is defined as a set of genes and a set of conditions in *X*_*U *_and *X*_*V*_. In the single-species biclustering case, we calculate a combined score for every gene in the genome (given the supporting data) to determine the likelihood of it being added or dropped from the bicluster. Extending this idea to the multi-species space requires that, for every orthologous pair, we can determine the likelihood of that ortholog pair being added or dropped from a given shared-space bicluster. We do this by combining the single-species gene scores (calculated separately for each organism within its independent data space) for the genes in an orthologous pair to compute the multi-species score *π*_*ik*_:

$$\pi_{ik}=p\left(y_{ik}=1|g_{ik}^{U},g_{ik}^{V}\right)\propto \exp\left(\beta_{0}+\beta_{1}\left(g_{ik}^{U}+g_{ik}^{V}\right)\right)$$

where $g_{ik}^{U}$ and $g_{ik}^{V}$ are the species-specific likelihoods for the members of pair *i *for bicluster *k*, and *β*_*0 *_and *β*_*1 *_are the parameters of the logistic regression. Note, this framework can easily be extended to more than two organisms, where the likelihood of the orthologous N-tuples for the *N *organisms would be defined as:

$$\pi_{ik}=p\left(y_{ik}^{1}=1,\ldots ,y_{ik}^{\left|N\right|}=1|g_{ik}^{1},\ldots ,g_{ik}^{\left|N\right|}\right)\propto \exp\left(\beta_{0}+\beta_{1}\sum_{n\in N}g_{ik}^{n}\right)$$

The parameters in this regression determine a decision boundary between genes in and out of the bicluster (fitted to the combined single-species scores for the pairs in *OC*_*UV *_at the previous iteration). It is important to note that individual data types from each species are not concatenated or combined through any other lossy or unbalanced transformation. The multiple species integration occurs solely via the computation of this decision boundary at this final step in computing the score. We believe that this imparts significant flexibility to the algorithm that will allow extension to other data types and larger collections of species in the future within this framework. For each organism *j *(*j *∈ {*U*, *V*}), $g_{ik}^{j}$, is defined as in the SSCM algorithm, as:

$$g_{ik}^{j}=r_{0}\log\left({\tilde{r}}_{ik}^{j}\right)+s_{0}\log\left({\tilde{s}}_{ik}^{j}\right)+\sum_{n\;\in \;N}q_{0}^{n}\log\left({\tilde{q}}_{nik}^{j}\right)$$

where ${\tilde{r}}_{ik}^{j}$, ${\tilde{s}}_{ik}^{j}$, and ${\tilde{q}}_{nik}^{j}$ are the individual likelihoods for the expression, sequence and networks, as defined by our earlier work and *r*_*0*_, *s*_*0 *_and *q*_*0 *_are mixing parameters set to roughly equalize the influence of each data type in this work (these mixing parameters can also be used to increase the influence of single data types if desired). For this work these mixing parameters were set such that each data type would have equal aggregate effect on the biclustering. Each of these individual score components, ${\tilde{r}}_{ik}^{j}$, ${\tilde{s}}_{ik}^{j}$, and ${\tilde{q}}_{nik}^{j}$, are described in [20]. The probability that any gene pair in *OC*_*UV *_is added to the growing bicluster is a well balanced function of the evidence derived from the integrated dataset for each species, formulated as the two multi-data scores, $g_{ik}^{1}$ and $g_{ik}^{2}$, that represent the individual species support values for each gene in an orthologous pair ($g_{ik}^{1}$ and $g_{ik}^{2}$ represent the multi-data-type integration for each organism separately and *π*_*ik *_affects the multi-species integration). Once this coupled version of the cMonkey score is obtained, the algorithm progresses in a manner similar to SSCM, but adding and removing pairs from the bicluster during each iteration instead, and stopping when convergence criteria are met [20,27]. At this stage, the formation of ortholog-pair biclusters, we limit any given bicluster to including only a single pair from any one particular ortholog family. Multiple members of an orthologous core can be included in different biclusters, and additional members of any given family of orthologs can be added in the following species-specific elaboration stage.

### Identification of species-specific elaborations of conserved-core biclusters (step 3)

In this step, we identify species-specific modifications to the biclusters that are discovered during the orthologous-pair biclustering described above (Figure 1c). To do this, we decouple the orthologous pairs from the shared-space modules to generate two biclusters, one for each organism, which represent the conserved cores of a putative, conserved, co-regulated module. These effectively serve as 'super-seeds' for this step, which are each separately optimized in a manner similar to the original SSCM method, but now considering the full genomes of each respective organism (genes without clear orthologs in the other organism can now be added if supported by the integrative score). Unlike the original method, though, in this step, we anchor these searches by preventing the genes from the original shared-space orthologous cores from being dropped. In so doing, we maintain the original putative, conserved module, while allowing the addition of species-specific or non-conserved orthologous core genes that fit well to the bicluster in a species-specific manner. During this stage, we also remove the constraint that only one gene from a given orthologous group can be selected by a given shared bicluster to permit detection of *bona fide *co-regulation of multiple members of paralogous gene families (for example, enabling the potential identification of dosage selection of paralogous genes). Finally, unlike either the shared-space or single-species optimizations previously described, where the mixing parameters, *r*_*0*_, *p*_*0 *_and *q*_*0*_, follow a structured annealing schedule during the optimization, in this optimization step we hold these mixing parameters constant, using the final values from the shared optimization for these.

### Identification of species-specific biclusters (optional step 4)

Once the multi-species analysis has been completed, as an optional final step, any species-specific modules that are completely unique to each organism can be identified by running SSCM on the remaining un-biclustered genes. We direct the reader to Additional file 1 for a more detailed description and discussion of this step as it is not a main focus of this first demonstration of our method. These last two species-specific steps provide our method with the strength and flexibility to simultaneously identify both conserved, partially conserved and species-specific modules, giving a correct limiting behavior across a wide range of possible species pairings.

### Gauging conservation between biclusters: the conservation score

We provide a measure for how well the single species version of the algorithm would do at detecting conserved co-regulation if the algorithm was run independently on each species dataset and then aligned and compared after the uncoupled single species biclusterings. To make this comparison, we introduce a clustering conservation metric, based on the F-statistic [48], to gauge the degree of conservation between a biclustering from one organism to that from another. Thus, for two organisms, *U *and *V*, we measure the degree of conservation between biclusters, $b_{k}^{U}$ and $b_{l}^{V}$, for each as:

$$Cons\left(b_{k}^{U},b_{l}^{V}\right)=\frac{2\cdot \left|OC_{b_{k}^{U},b_{l}^{V}}\right|}{\left|OC_{b_{k}^{U}}\right|+\left|OC_{b_{l}^{V}}\right|}$$

where $OC_{b_{k}^{U}}$ is the set of genes from bicluster $b_{k}^{U}$ that belong to the orthologous core for genome *U*, *OC*_*U*_, (where $OC_{b_{k}^{U}}\subseteq b_{k}^{U}$ and $OC_{b_{k}^{U}}\subset OC_{U}\subset G_{U}$). Likewise $OC_{b_{l}^{V}}$ is defined similarly for genome *V *and *OC*_*V*_. $OC_{b_{k}^{U},b_{l}^{V}}=OC_{b_{k}^{U}}\cap OC_{b_{l}^{V}}$ is the set of genes in bicluster $b_{k}^{U}$ with direct matching orthologs in $b_{l}^{V}$, and vice versa. This measure is similar to an F-statistic that gauges the ability of the biclustering for one organism to recall the biclustering of another (after orthology-based alignment/matching of the biclusters). To make this metric more general, in the case of multi-member orthologous families, we consider all possible pairs, including those with putative paralogs, such as those returned by InParanoid.

### Multi-species k-means and balanced multi-species k-means

For comparison we also re-implemented a simple MSKM method similar to the method used in Herschkowitz *et al*. [46] to compare human and mouse microarray data. In this simple method, only the reciprocal best Blast matches are selected as orthologous pairs. These one-to-one pairwise relationships are first used to form a concatenated expression matrix, so that a row in this matrix corresponds to the concatenation of the expression data for two orthologous genes. This concatenated expression matrix is next clustered using k-means, using the Euclidean distance metric and with k = 150 (as this was the same size used for the test of the MSCM method) to generate what we will call shared k-means clusters. Next, as a modification to Herschkowitz *et al*.'s shared k-means algorithm, we added a subsequent step, similar to the elaboration step of the MSCM algorithm. In this step, the components of the shared k-means centroids are separated by organism (into the components that correspond to the organism-specific conditions of the concatenated expression dataset). For each organism, then, the organism-specific shared k-means (sub-)centroids are used to perform a Voronoi partitioning of that organism's non-orthologous core expression data. Thus, in this step, the orthologous genes that belonged to the original shared k-means clusters remain in their original cluster.

As our comparisons indicated that MSKM is prone to allowing an organism to dominate the analysis if its expression data have far more conditions than those of the other organism, we also implemented a balanced version of the multi-species k-means algorithm (BMSKM). There are a number of ways this balancing could be implemented. One would be to use individual weights for the different conditions from the different species. Another, even simpler implementation, which we used, is to concatenate the smaller dataset to itself so that it has roughly an equivalent number of conditions as the larger dataset, and use this in the MSKM analysis instead. For example, when *B. anthracis*, with 51 conditions in its expression data, was paired with *B. subtilis*, which has >300 conditions, a new dataset for *B. anthracis *was generated that contained the original *B. anthracis *dataset concatenated it to itself 5 times, so that there were 6 copies of each condition. This analysis was not applied to this pairing of *B. anthracis *and *L. monocytogenes *as their expression datasets are roughly equivalent in size.

### Multi-species Iterative Signature Algorithm

We re-implemented a multi-species version of the ISA described by Bergmann *et al*. [13], using the isa2 package for R [13,81], available from the Comprehensive R Archive Network. A more thorough discussion of the MSISA method can be found in Additional file 1, but as a quick review of the method, MSISA contains five main steps. First, a well-characterized organism is used as a 'reference' organism, with a less characterized organism as the 'target' organism (note, we use the terminology of a later paper from the same group [35], which employs a similar strategy for multi-species comparisons). Second, using a pre-generated set of biclusters from the reference organism, biclusters containing genes that have putative orthologs in the target organism are selected and used to generate 'homologous' biclusters for the target organism that contain these putative orthologs such that there is a direct one-to-one mapping between the biclusters for both organisms. Third, standard, single-species ISA is performed on the target organism, using only these homologous biclusters as seeds. Fourth, the intersection of the homologous bicluster seeds input into step 3 and resulting biclusters produced by step 3 are selected to generate a set of 'purified' biclusters in order to select only the conserved genes in the reference organism. In the final step, single-species ISA is run again on each organism, but using the purified biclusters to generate a set of 'refined' biclusters for each organism. As such, this step is similar to the elaboration step of MSCM as it allows species-specific modifications to be added to the purified bicluster.

For combinatoric reasons, MSISA was only applied to the pairings involving *B. subtilis*, using *B. subtilis *as the reference organism as it is the best studied organism of the three we consider in this study. Hence there are no MSISA results to report for the pairing of *B. anthracis *with *L. monocytogenes*.

### Visualization and exploration of multi-species biclusters

The Comparative Microbial Module Resource [82] is an integrated collection of diverse functional genomics datasets and software tools that facilitate the visualization and analysis of conserved cMonkey biclusters, or putatively co-regulated gene modules, across species. The interface allows the visualization and exploration of a bicluster's properties (such as coupled multi-species biclusters, conserved orthologous core gene members, species-specific gene members, experimental conditions, gene coexpression pattern, sequence motif logos, and significant functional annotations). Integration with the Gaggle allows access to additional biological information from online databases and further analysis (for example, integrated tools include, but are not limited to, the FireGoose plugin, cytoscape, the Data Matrix Viewer, and an R goose for using the R language and environment for statistical computing and graphics).

### Multi-species cMonkey code release, maintenance and documentation

Both the MSCM and a re-factored SSCM are freely available for download and use [76]. This website includes functionality for bug tracking, tutorials on use, example datasets and runs of the algorithm, links to required packages, and python code developed to aid in data import. MSCM is written in R [83] with a data import module written in Python and has three main modules: a reader; the main code; and validation and visualization codes. With regards to the reader, cMonkey is given gene expression matrices and ortholog pairs, along with optional protein association networks and upstream sequences. The user may request cMonkey to automatically find the required and optional data types for each organism. The main code is written in R and contains bicluster seeding, bicluster overall optimization, scoring functions, and methods for output and visualization of results. The validation and visualization codes implement the bicluster and biclustering assessment described, code to facilitate connection to network and cluster visualization tools, such as the Gaggle. All code (cMonkey, the reader, and validation code) are freely available.

We have attempted to make several of the steps required for assembling and integrated dataset automatic in this code release, in the hope that this will extend the usefulness of the algorithm to a greater number of biologists. The biologist needs only to prepare simple gene expression matrices and pairs of orthologs. The rest of the data types will be queried from biological databases (networks, sequences, annotations for validation scripts, and so on). All input and output will also be stored in a portable, standard relational database that will readily permit use of the integrated dataset and cMonkey results by other tools. These key changes to how data are imported and stored in cMonkey's database and the core data-object for cMonkey allow for multi-species integration. The biologist may use the reader in two modes: automatic or manual. In automatic mode, the biologist need prepare only gene expression matrices and pairs of orthologs, while protein association networks and upstream sequences are queried from biological databases such as BioNetBuilder [84], MicrobesOnline [85], Prolinks [24], STRING [86,87] and RSAT [88,89].

### Swimming motility assays

Individual colonies of *B. subtilis *PY79 [90] and DS219 [91], *B. cereus *ATCC 14579 (obtained from Daniel Ziegler, *Bacillus* Genetic Stock Center, Ohio State University) and *B. anthracis *Sterne (a gift from Adam Driks, Loyola University Chicago) were picked with a wooden stick and inoculated into Luria-Bertaini (LB) 10 g tryptone, 5 g yeast extract, 5 g NaCL per liter of broth. Cultures were grown to log phase and 3 μl of the broth culture was centrally inoculated on LB agar plates containing 0.3% agar. Motility was scored after approximately 20 hours incubation at 30°C. Plates were photographed against a dark background such that areas of bacterial colonization appear light.

## Abbreviations

BMSKM: balanced multi-species k-means; COAL: Coalesce biclustering method; EL: elaborated biclusters (multi-species biclusters that have additional genes unique to each organism added (MSCM, MSKM, BMSKM)); EO: expression only; FD: full data (EO and FD are used to distinguish between expression only tests and full data runs of integrative methods); GO: Gene Ontology; ISA: Iterative Signature Algorithm; KEGG: Kyoto Encyclopedia of Genes and Genomes; LB: Luria-Bertaini; MS: multiple-species; MSCM: multi-species cMonkey; MSISA: multi-species ISA; MSKM: multi-species k-means; OC: orthologous core (the set of actively expressed orthologous genes shared between a group of organisms on which we run our multi-species biclustering); P: purified biclusters (applies only to the ISA algorithm (MSISA-P)); QUBIC: QUalitative BIClustering algorithm; R: refined biclusters (applies only to the ISA algorithm (MSISA-R)); SH: shared biclusters (biclusters generated only from orthologous pairs (MSCM, MSKM, BMSKM)); SS: single species; SSCM: single-species cMonkey.

## Authors' contributions

PHW implemented and tested the method, and prepared the manuscript. TJK collated the datasets analyzed, generated the visualizations of the results, and aided in validation of results. ARB did extensive studies of the sporulation and flagellar biclusters, performed the motility assays and helped in the writing of the manuscript. DJR helped conceive of the project, and provided initial guidance. DBK provided guidance on the biological interpretation and analysis of results and provided strains for motility assays. PE oversaw all biological aspects of the project, contributed to the validation and visualization of the results, and aided in the writing of the manuscript. RB conceived and oversaw all aspects of the project, and aided in the writing of the manuscript. All authors read and approved the final manuscript.

## Supplementary Material

Additional file 1 **Additional results**. This document contains detailed descriptions of the dataset and any external tools used in our analysis; additional method steps not described in the main text; detailed definitions of the global bicluster quality metrics, figures and descriptions of the statistical tests comparing the results from the different methods compared; gene lists and bicluster images of the sporulation and flagellar biclusters described above; and further information regarding the *B. anthracis *flagellar pathway genes. Click here for file
