# Supplementary material for: Multi-species integrative biclustering
Source: Genome Biol. 2010 Sep 29;11(9):R96. doi: 10.1186/gb-2010-11-9-r96 (PMC2965388; doi:10.1186/gb-2010-11-9-r96)
Supplement: Additional file 1 — Additional results. This document contains detailed descriptions of the dataset and any external tools used in our analysis; additional method steps not described in the main text; detailed definitions of the global bicluster quality metrics, figures and descriptions of the statistical tests comparing the results from the different methods compared; gene lists and bicluster images of the sporulation and flagellar biclusters described above; and further information regarding the B. anthracis flagellar pathway genes. [file gb-2010-11-9-r96-S1.DOC]

Table of Contents

[Additional Results 3](#__RefHeading___Toc268265912)

[Overview of the (bi)cluster comparison metrics 3](#__RefHeading___Toc268265913)

[Quick-glance tables for pairings involving L. monocytogenes 3](#__RefHeading___Toc268265914)

[Additional (bi)cluster coherence metric figures 6](#__RefHeading___Toc268265915)

[Residuals 6](#__RefHeading___Toc268265916)

[Average pairwise correlations 7](#__RefHeading___Toc268265917)

[Network Association p-values 9](#__RefHeading___Toc268265918)

[Motif E-values 10](#__RefHeading___Toc268265919)

[Sequence p-values 12](#__RefHeading___Toc268265920)

[Additional size distribution, overlap and coverage figures 13](#__RefHeading___Toc268265921)

[Number of genes 13](#__RefHeading___Toc268265922)

[Number of conditions 15](#__RefHeading___Toc268265923)

[Coverage (matrix element-wise) 16](#__RefHeading___Toc268265924)

[Coverage (matrix gene-wise) 18](#__RefHeading___Toc268265925)

[Overlap (matrix element-wise) 19](#__RefHeading___Toc268265926)

[Overlap (matrix gene-wise) 21](#__RefHeading___Toc268265927)

[Comparison of the (bi)cluster coherence metrics 22](#__RefHeading___Toc268265928)

[Comparisons with FD-MSCM 22](#__RefHeading___Toc268265929)

[Residuals 22](#__RefHeading___Toc268265930)

[Mean correlations 23](#__RefHeading___Toc268265931)

[Network Association p-values 24](#__RefHeading___Toc268265932)

[Motif E-values 26](#__RefHeading___Toc268265933)

[Sequence p-values 27](#__RefHeading___Toc268265934)

[Comparisons with EO-MSCM 28](#__RefHeading___Toc268265935)

[Residuals 28](#__RefHeading___Toc268265936)

[Mean correlations 29](#__RefHeading___Toc268265937)

[Network Association p-values 30](#__RefHeading___Toc268265938)

[Motif E-values 32](#__RefHeading___Toc268265939)

[Sequence p-values 33](#__RefHeading___Toc268265940)

[Comparisons with Randomized tests 34](#__RefHeading___Toc268265941)

[Residuals 34](#__RefHeading___Toc268265942)

[Mean correlations 35](#__RefHeading___Toc268265943)

[Additional GO term and KEGG pathway enrichment figures 36](#__RefHeading___Toc268265944)

[Putative σ^E^ binding site in the ctaC operon upstream sequence 38](#__RefHeading___Toc268265945)

[Flagellar motility regulators and missing genes 39](#__RefHeading___Toc268265946)

[Full descriptions of highlighted biclusters 41](#__RefHeading___Toc268265947)

[Gene lists for B. subtilis, B. anthracis Sterne sporulation clusters 32, 82, and 84. 41](#__RefHeading___Toc268265948)

[B. subtilis - B. anthracis cluster 32 41](#__RefHeading___Toc268265949)

[B. subtilis - B. anthracis cluster 82 45](#__RefHeading___Toc268265950)

[B. subtilis - B. anthracis cluster 84 48](#__RefHeading___Toc268265951)

[Gene lists for flagellar clusters 50](#__RefHeading___Toc268265952)

[B. subtilis - B. anthracis cluster 58: 51](#__RefHeading___Toc268265953)

[B. subtilis - L. monocytogenes cluster 79 54](#__RefHeading___Toc268265954)

[B. anthracis - L. monocytogenes cluster 102 57](#__RefHeading___Toc268265955)

[Materials 59](#__RefHeading___Toc268265956)

[Data set analyzed 59](#__RefHeading___Toc268265957)

[External tools used 61](#__RefHeading___Toc268265958)

[Methods 61](#__RefHeading___Toc268265959)

[Additional multi-species cMonkey biclustering steps: 61](#__RefHeading___Toc268265960)

[Seeding the initial biclusters: 61](#__RefHeading___Toc268265961)

[Extend to include species-specific modules: 62](#__RefHeading___Toc268265962)

[Multi-Species Iterative Signature Algorithm 62](#__RefHeading___Toc268265963)

[Explanation of the (bi)cluster coherence metrics 64](#__RefHeading___Toc268265964)

[Residuals 64](#__RefHeading___Toc268265965)

[Mean correlations 64](#__RefHeading___Toc268265966)

[Network Association p-values 65](#__RefHeading___Toc268265967)

[Motif E-values 65](#__RefHeading___Toc268265968)

[Sequence p-values 66](#__RefHeading___Toc268265969)

[References 66](#__RefHeading___Toc268265970)

# Additional Results

We provide here again Table 1 from the main text here for quick translation of the method acronyms (Table S1).

Table S1: Quick lookup table for methods considered by this study.

|  |  | **Expression Only** | |  | **Full Data** | |
| --- | --- | --- | --- | --- | --- | --- |
| **Multi-Species** | | **shared space** | **full genome (elaboration)** |  | **shared space** | **full genome (elaboration)** |
|  | **cMonkey** | EO-MSCM-SH | EO-MSCM-EL |  | FD-MSCM-SH | FD-MSCM-EL |
|  | **ISA*** | MSISA-P | MSISA-R |  | NA | NA |
|  | **K-Means*** | MSKM-SH | MSKM-EL |  | NA | NA |
|  | **(Balanced) K-Means*** | BMSKM-SH | BMSKM-EL |  | NA | NA |
|  |  |  |  |  |  |  |
|  |  |  |  |  |  |  |
| **Single-Species** | | **Expression Only** | |  | **Full Data** | |
|  | **cMonkey** | EO-SSCM | |  | FD-SSCM | |
|  | **Coalesce** | EO-COAL | |  | FD-COAL | |
|  | **Qubic*** | QUBIC | |  | NA | |
|  |  |  |  |  |  |  |
|  |  |  |  |  |  |  |
|  | *** Expression only method by method definition - no distinction between "expression only" or "full data" is necessary.** | | | | | |
|  |
|  |

## Overview of the (bi)cluster comparison metrics

We compared the relative performances of the four multi-species methods (MSCM, MSISA, MSKM and BMSKM), and the three single species methods (SSCM, Coalesce and Qubic) compared in this study using 5 metric classes: 1) bicluster coherence; 2) functional enrichment; 3) coverage; 4) overlap between biclusters; and 5) conservation, described in the main text (Table 2, and Tables S2-3). We gauge bicluster coherence with five commonly used metrics that gauge the degree of support that is provided to each bicluster by the three data types that cMonkey integrates (expression, sequence and association networks). For comparison of SSCM to other biclustering algorithms, and comparison between single species biclustering and clustering algorithms, see [20, 47]. Our coherence metrics are: 1) expression residuals, a measure of the coherence of expression across the two species datasets for conditions within the bicluster; 2) mean correlation, the average pairwise correlation between members of a (bi)cluster (taking the absolute value to allow fair comparison between methods that identify inversely correlated patterns (QUBIC and MSISA) and those that do not; 3) network p-values, a measure of the significance of the sub-networks within biclusters compared to the full network; 4) motif E-values, a measure of the quality/significance of the upstream binding site motifs detected for each bicluster; and 5) sequence p-values, an estimate of a sequence’s match to the motifs associated with a (bi)cluster. Each of the coherence metrics will be described in greater detail below as we discuss the relative performance of MSCM to the other methods.

## Quick-glance tables for pairings involving L. monocytogenes

Table S2: Summary of evaluation criteria for the single and multi-species methods for the B. subtilis – L. monocytogenes pairing. We compare several metrics of bicluster conservation, coverage, and functional enrichment. In all cases metrics are averaged over all biclusters produced by that method. Abbreviations are given for each method; translations can be found in Table S3. In each column, the results for B. subtilis are listed first, with those for L. monocytogenes listed in parentheses.

|  | **Conservation  Score** | **Mean Correlation (absolute value)** | **Net p-value  (-log10)** | **Number of Genes** | **Number of Conditions** | **Number of Biclusters** |
| --- | --- | --- | --- | --- | --- | --- |
| EO MSCM-SH | 1 | 0.52 (0.64) | 15.18 (8.20) | 14.51 (14.51) | 127.45 (27.31) | 150 (150) |
| **FD MSCM-SH** | **1** | **0.59 (0.80)** | **10.73 (8.79)** | **16.09 (16.09)** | **121.36 (25.96)** | **147 (147)** |
| MSISA-P | 1 | 0.60 (0.47) | 6.82 (0.00) | 5.88 (5.88) | 10.85 (4.97) | 33 (33) |
| MSKM-SH | 1 | 0.59 (0.51) | 12.14 (12.66) | 9.83 (9.83) | 314 (56) | 145 (145) |
| **BMSKM-SH** | **1** | **0.52 (0.63)** | **11.96 (12.39)** | **9.78 (9.78)** | **314 (56)** | **146 (146)** |
| EO MSCM-EL | 0.951 | 0.54 (0.64) | 13.59 (8.49) | 20.05 (18.92) | 132.92 (30.29) | 150 (150) |
| **FD MSCM-EL** | **0.884** | **0.61 (0.81)** | **9.13 (7.41)** | **26.44 (25.73)** | **123.17 (28.84)** | **147 (147)** |
| MSISA-R | 0.060 | 0.55 (0.50) | 3.15 (3.12) | 106.39 (113.05) | 10.37 (6.42) | 38 (38) |
| MSKM-EL | 0.963 | 0.56 (0.55) | 7.52 (8.37) | 26.85 (18.66) | 314 (56) | 145 (145) |
| **BMSKM-EL** | **0.949** | **0.53 (0.64)** | **7.55 (9.43)** | **26.90 (19.14)** | **314 (56)** | **146 (146)** |
| EO SSCM | 0.096 | 0.70 (0.86) | 8.58 (4.98) | 26.19 (30.95) | 193.40 (40.99) | 161 (83) |
| **FD SSCM** | **0.147** | **0.56 (0.71)** | **10.14 (6.70)** | **23.06 (19.79)** | **200.76 (42.32)** | **295 (300)** |
| EO COAL | 0.088 | 0.58 (0.81) | 5.21 (5.60) | 86.65 (78.81) | 20.09 (12.04) | 300 (81) |
| **FD COAL** | **0.095** | **0.59 (0.80)** | **5.27 (5.46)** | **88.16 (84.15)** | **20.24 (12.73)** | **287 (78)** |
| QUBIC | 0.048 | 0.36 (0.45) | 1.38 (5.26) | 71.59 (182.92) | 25.45 (19.91) | 150 (150) |
|  |  |  |  |  |  |  |
|  |  |  | **GO** |  | **KEGG** |  |
|  | **Coverage  (element-wise)** | **Overlap  (element-wise)** | **Percent Significant (bi)clusters** | **Number of Significant Terms** | **Percent Significant (bi)clusters** | **Number of Significant Terms** |
| EO MSCM-SH | 15.65% (26.16%) | 6.46% (6.17%) | 22.67% (20.67%) | 339 (303) | 4.67% (3.33%) | 11 (13) |
| **FD MSCM-SH** | **15.85% (26.08%)** | **5.95% (5.87%)** | **37.41% (35.37%)** | **427 (371)** | **10.20% (6.12%)** | **19 (19)** |
| MSISA-P | 0.14% (0.42%) | 29.03% (45.95%) | 48.15% (37.04%) | 109 (86) | 18.18% (21.21%) | 8 (9) |
| MSKM-SH | 36.28% (50.98%) | 0% (0%) | 37.93% (34.48%) | 500 (398) | 10.34% (8.97%) | 24 (27) |
| **BMSKM-SH** | **36.35% (51.09%)** | **0% (0%)** | **30.82% (31.51%)** | **479 (411)** | **11.64% (11.64%)** | **18 (24)** |
| EO MSCM-EL | 21.98% (32.52%) | 5.36% (6.47%) | 37.33% (30.67%) | 449 (386) | 8.67% (8.00%) | 16 (15) |
| **FD MSCM-EL** | **25.95% (40.29%)** | **4.65% (6.12%)** | **56.46% (53.74%)** | **542 (468)** | **16.33% (10.20%)** | **23 (19)** |
| MSISA-R | 2.27% (5.44%) | 17.80% (57.90%) | 97.37% (92.11%) | 285 (179) | 31.58% (57.89%) | 13 (14) |
| MSKM-EL | 99.11% (96.78%) | 0.00% (0.00%) | 59.31% (44.14%) | 640 (476) | 15.17% (11.03%) | 28 (20) |
| **BMSKM-EL** | **100% (100%)** | **0.00% (0.00%)** | **51.37% (46.58%)** | **669 (480)** | **12.33% (11.64%)** | **25 (24)** |
| EO SSCM | 39.48% (37.34%) | 9.44% (15.76%) | 42.24% (55.42%) | 499 (298) | 10.56% (19.28%) | 19 (15) |
| **FD SSCM** | **54.55% (61.27%)** | **7.53% (13.19%)** | **50.51% (36.91%)** | **746 (451)** | **11.53% (5.67%)** | **32 (17)** |
| EO COAL | 40.21% (41.73%) | 1.94% (8.65%) | 63.67% (53.09%) | 744 (319) | 17.67% (11.11%) | 32 (12) |
| **FD COAL** | **39.39% (43.07%)** | **2.06% (9.69%)** | **64.81% (56.41%)** | **776 (294)** | **16.03% (11.54%)** | **24 (11)** |
| QUBIC | 2.43% (9.14%) | 38.34% (62.22%) | 43.33% (100.00%) | 227 (175) | 3.33% (62.00%) | 5 (4) |

Table S3: Summary of evaluation criteria for the single and multi-species methods for the B. anthracis – L. monocytogenes pairing. We compare several metrics of bicluster conservation, coverage, and functional enrichment. In all cases metrics are averaged over all biclusters produced by that method. Abbreviations are given for each method; translations can be found in Table S3. In each column, the results for B. anthracis are listed first, with those for L. monocytogenes listed in parentheses. Note, results for MSISA and BMSKM are not reported as these methods were not performed for this pairing.

|  | **Conservation  Score** | **Mean Correlation (absolute value)** | **Net p-value  (-log10)** | **Number of Genes** | **Number of Conditions** | **Number of Biclusters** |
| --- | --- | --- | --- | --- | --- | --- |
| EO MSCM-SH | 1 | 0.63 (0.63) | 5.90 (5.92) | 15.78 (15.78) | 25.60 (27.51) | 141 (141) |
| **FD MSCM-SH** | **1** | **0.82 (0.77)** | **8.82 (6.28)** | **16.81 (16.81)** | **24.82 (26.05)** | **148 (148)** |
| *MSISA-P* | *NA* | *NA* | *NA* | *NA* | *NA* | *NA* |
| MSKM-SH | 1 | 0.69 (0.60) | 9.95 (13.62) | 10.20 (10.20) | 51.00 (56.00) | 145 (145) |
| *BMSKM-SH* | *NA* | *NA* | *NA* | *NA* | *NA* | *NA* |
| EO MSCM-EL | 0.963 | 0.63 (0.63) | 6.79 (6.51) | 20.69 (19.79) | 26.96 (30.59) | 141 (141) |
| **FD MSCM-EL** | **0.906** | **0.80 (0.78)** | **8.15 (5.59)** | **25.26 (23.90)** | **26.43 (28.97)** | **148 (148)** |
| *MSISA-R* | *NA* | *NA* | *NA* | *NA* | *NA* | *NA* |
| MSKM-EL | 0.943 | 0.70 (0.63) | 5.95 (9.19) | 39.63 (19.11) | 51.00 (56.00) | 145 (145) |
| *BMSKM-EL* | *NA* | *NA* | *NA* | *NA* | *NA* | *NA* |
| EO SSCM | 0.090 | 0.91 (0.86) | 7.43 (4.98) | 34.11 (30.95) | 38.66 (40.99) | 210 ( 83) |
| **FD SSCM** | **0.126** | **0.82 (0.71)** | **7.31 (6.70)** | **42.02 (19.79)** | **39.87 (42.32)** | **300 (300)** |
| EO COAL | 0.102 | 0.64 (0.81) | 5.06 (5.60) | 115.71 (78.81) | 13.13 (12.04) | 158 (81) |
| **FD COAL** | **0.101** | **0.62 (0.80)** | **5.69 (5.46)** | **131.12 (84.15)** | **14.24 (12.73)** | **136 (78)** |
| QUBIC | 0.045 | 0.49 (0.45) | 5.90 (5.26) | 188.25 (182.92) | 12.63 (19.91) | 150 (150) |
|  |  |  |  |  |  |  |
|  |  |  | **GO** |  | **KEGG** |  |
|  | **Coverage  (element-wise** | **Overlap  (element-wise)** | **Percent Significant (bi)clusters** | **Number of Significant Terms** | **Percent Significant (bi)clusters** | **Number of Significant Terms** |
| EO MSCM-SH | 12.99% (26.51%) | 6.03% (5.51%) | 20.57% (21.28%) | 281 (286) | 7.09% (6.38%) | 10 (11) |
| **FD MSCM-SH** | **13.97% (27.33%)** | **6.71% (5.87%)** | **40.54% (43.24%)** | **432 (423)** | **10.14% (10.81%)** | **18 (17)** |
| *MSISA-P* | *NA* | *NA* | *NA* | *NA* | *NA* | *NA* |
| MSKM-SH | 25.22% (52.92%) | 0.00% (0.00%) | 38.62% (37.24%) | 454 (443) | 9.66% (9.66%) | 20 (21) |
| *BMSKM-SH* | *NA* | *NA* | *NA* | *NA* | *NA* | *NA* |
| EO MSCM-EL | 16.89% (32.56%) | 5.60% (6.10%) | 52.48% (33.33%) | 466 (359) | 9.22% (7.09%) | 21 (13) |
| **FD MSCM-EL** | **20.80% (38.82%)** | **5.99% (5.61%)** | **79.73% (56.76%)** | **590 (479)** | **16.22% (14.86%)** | **30 (23)** |
| *MSISA-R* | *NA* | *NA* | *NA* | *NA* | *NA* | *NA* |
| MSKM-EL | 97.97% (99.14%) | 0.00% (0.00%) | 79.31% (53.79%) | 742 (525) | 11.72% (11.03%) | 24 ( 24) |
| *BMSKM-EL* | *NA* | *NA* | *NA* | *NA* | *NA* | *NA* |
| EO SSCM | 46.81% (37.34%) | 14.10% (15.76%) | 66.19% (55.42%) | 629 (298) | 17.62% (19.28%) | 29 (15) |
| **FD SSCM** | **60.29% (61.27%)** | **15.72% (13.19%)** | **62.33% (36.91%)** | **707 (451)** | **10.00% (5.67%)** | **32 (17)** |
| EO COAL | 66.40% (41.73%) | 2.12% (8.65%) | 76.58% (53.09%) | 659 (319) | 9.49% (11.11%) | 24 (12) |
| **FD COAL** | **66.63% (43.07%)** | **2.16% (9.69%)** | **80.88% (56.41%)** | **686 (294)** | **14.71% (11.54%)** | **24 (11)** |
| QUBIC | 12.95% (9.14%) | 26.49% (62.22%) | 88.67% (100.00%) | 331 (175) | 14.67% (62.00%) | 13 (4) |

## Additional (bi)cluster coherence metric figures

### Residuals

Figure S1: Residuals from the B. subtilis – B. anthracis pairing. The distributions of the residuals from all methods considered by this study for the B. subtilis- B. anthracis pairing. Next to each distribution, in gray, are residuals from randomly shuffled (bi)clusters that match the size distribution for each method. Explanations of the method name abbreviations can be found in Table S1.


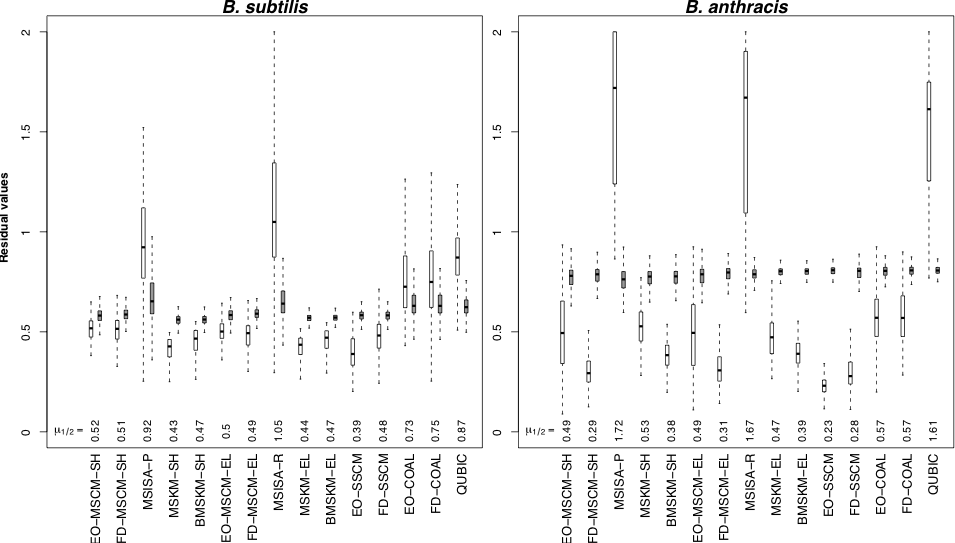


Figure S2: Residuals from the B. subtilis – L. monocytogenes pairing. The distributions of the residuals from all methods considered by this study for the B. subtilis- L. monocytogenes pairing. Next to each distribution, in gray, are residuals from randomly shuffled (bi)clusters that match the size distribution for each method. Explanations of the method name abbreviations can be found in Table S1.


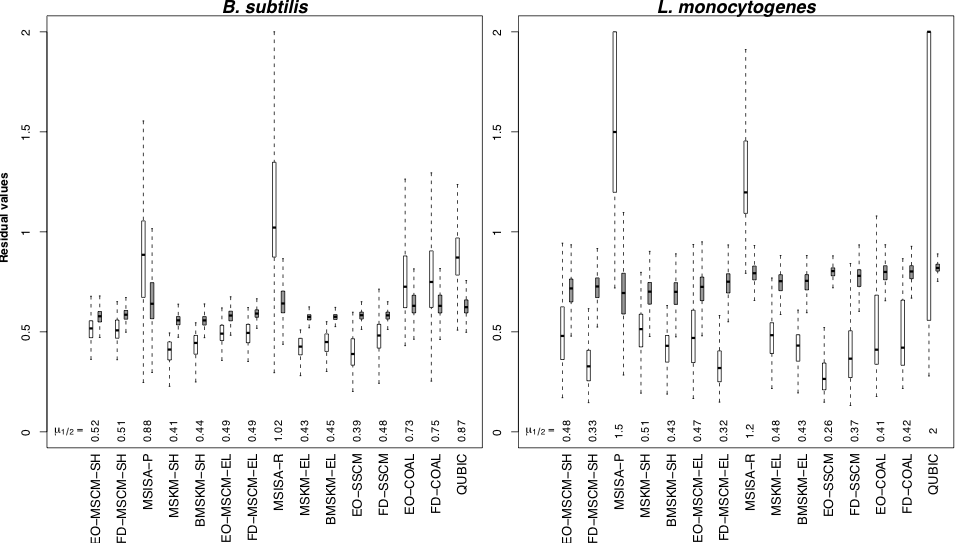


Figure S3: Residuals from the B. anthracis – L. monocytogenes pairing. The distributions of the residuals from all methods considered by this study for the B. anthracis – L. monocytogenes pairing. Next to each distribution, in gray, are residuals from randomly shuffled (bi)clusters that match the size distribution for each method. Explanations of the method name abbreviations can be found in Table S1.


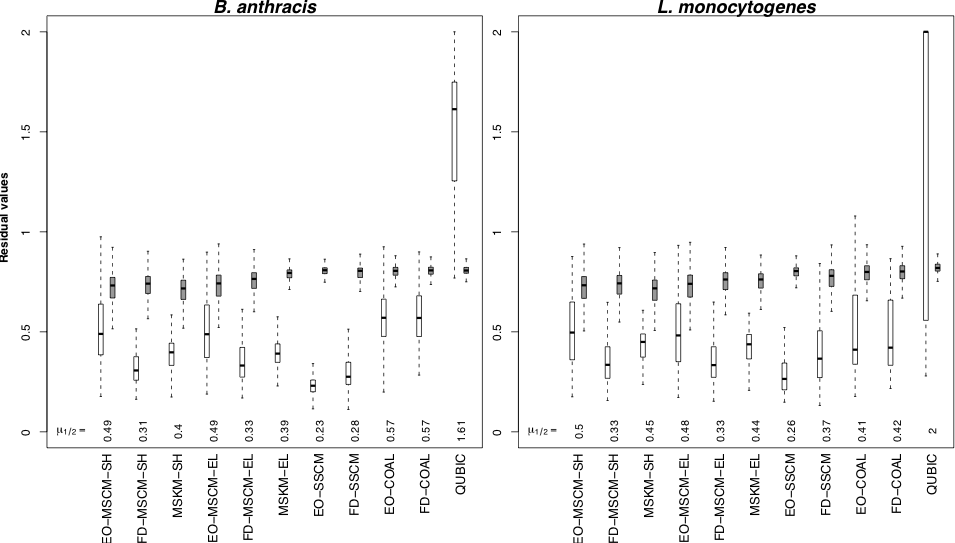


### Average pairwise correlations

Figure S4: Mean correlations from the B. subtilis – B. anthracis pairing. The distributions of the mean correlations from all methods considered by this study for the B. subtilis- B. anthracis pairing. Next to each distribution, in gray, are residuals from randomly shuffled (bi)clusters that match the size distribution for each method. Explanations of the method name abbreviations can be found in Table S1.


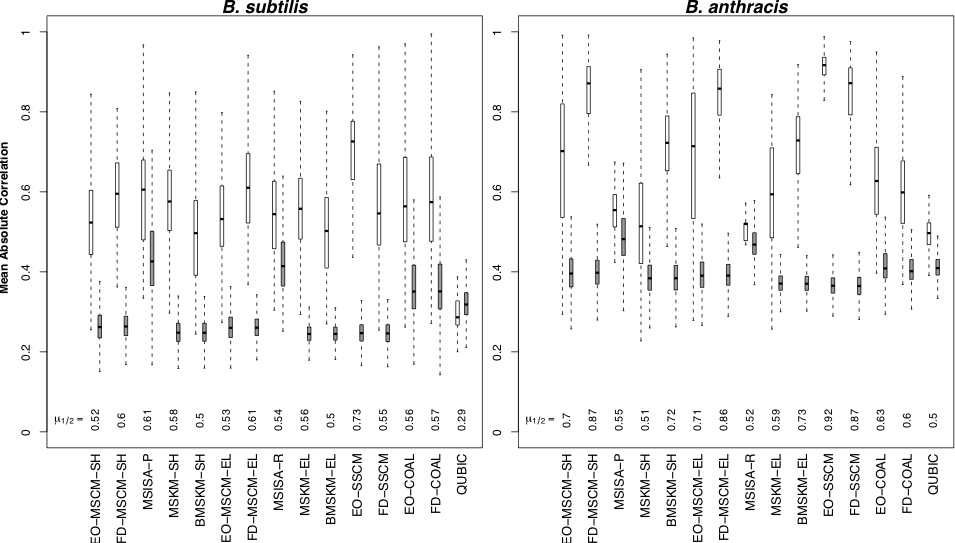


Figure S5: Mean correlations from the B. subtilis – L. monocytogenes pairing. The distributions of the mean correlations from all methods considered by this study for the B. subtilis- L. monocytogenes pairing. Next to each distribution, in gray, are residuals from randomly shuffled (bi)clusters that match the size distribution for each method. Explanations of the method name abbreviations can be found in Table S1.


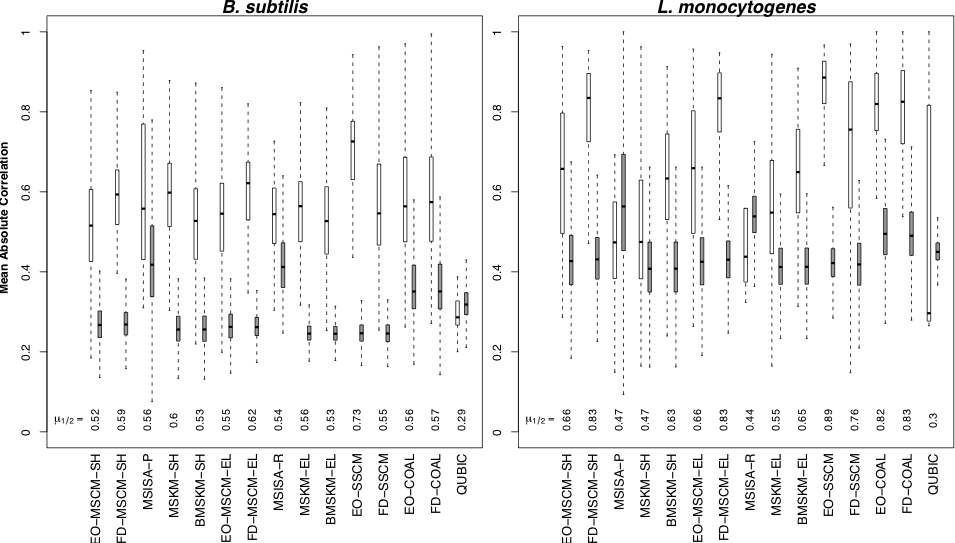


Figure S6: Mean correlations from the B. anthracis – L. monocytogenes pairing. The distributions of the mean correlations from all methods considered by this study for the B. anthracis – L. monocytogenes pairing. Next to each distribution, in gray, are residuals from randomly shuffled (bi)clusters that match the size distribution for each method. Explanations of the method name abbreviations can be found in Table S1.


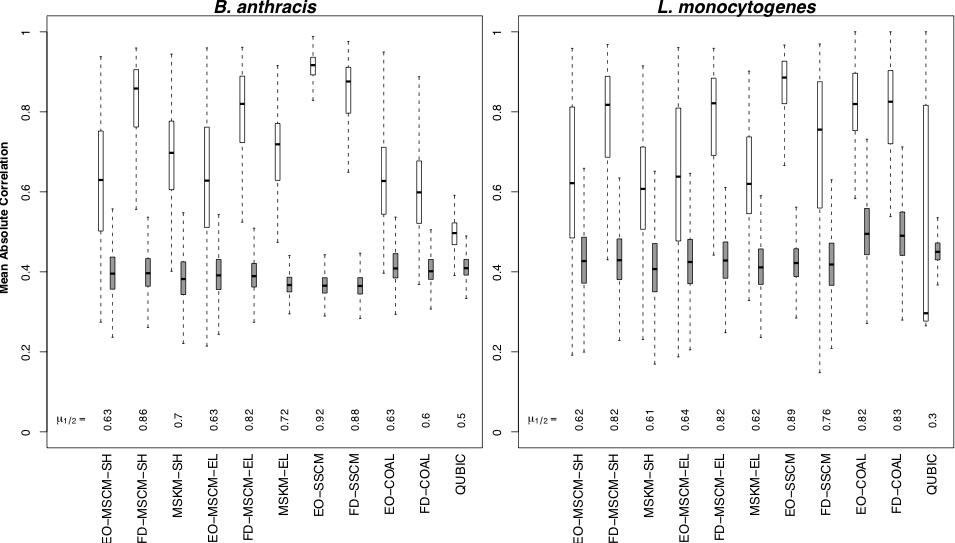


### Network Association p-values

Figure S7: Network Association p-values from the B. subtilis – B. anthracis pairing. The distributions of the network association p-values (-log_10_) from all methods considered by this study for the B. subtilis- B. anthracis pairing. Explanations of the method name abbreviations can be found in Table S1.

**
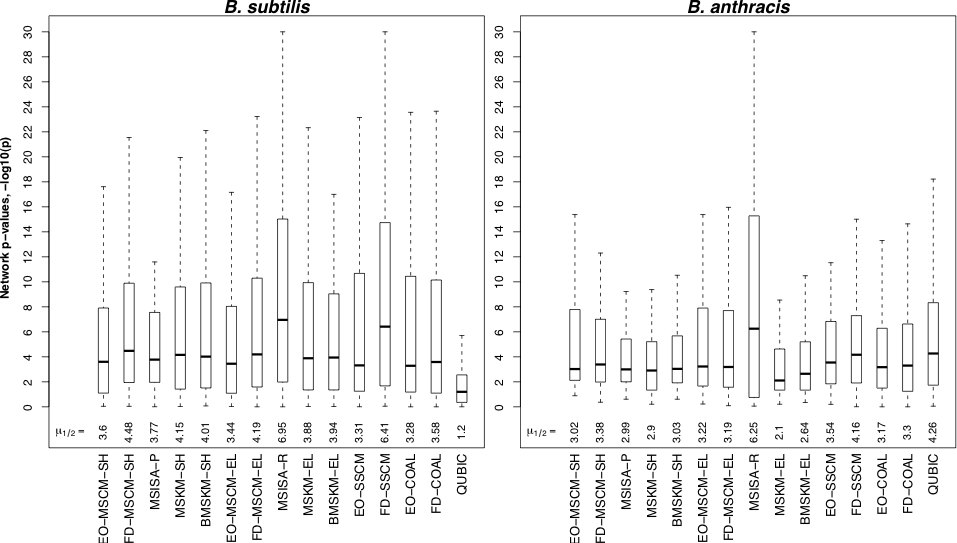
**

Figure S8: Network Association p-values from the B. subtilis – L. monocytogenes pairing. The distributions of the network association p-values (-log_10_) from all methods considered by this study for the B. subtilis- L. monocytogenes pairing. Explanations of the method name abbreviations can be found in Table S1.

**
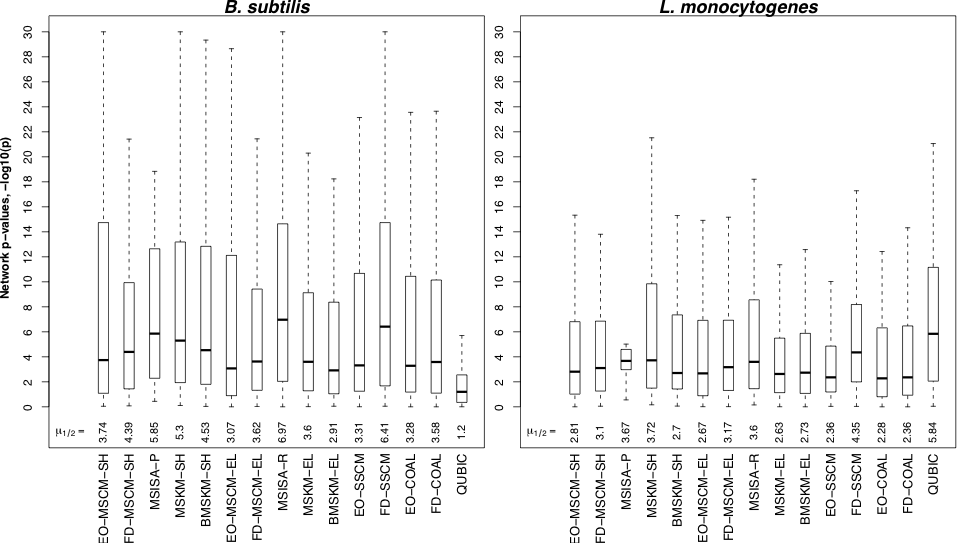
**

Figure S9: Network Association p-values from the B. anthracis – L. monocytogenes pairing. The distributions of the network association p-values (-log_10_) from all methods considered by this study for the B. anthracis- L. monocytogenes pairing. Explanations of the method name abbreviations can be found in Table S1.

**
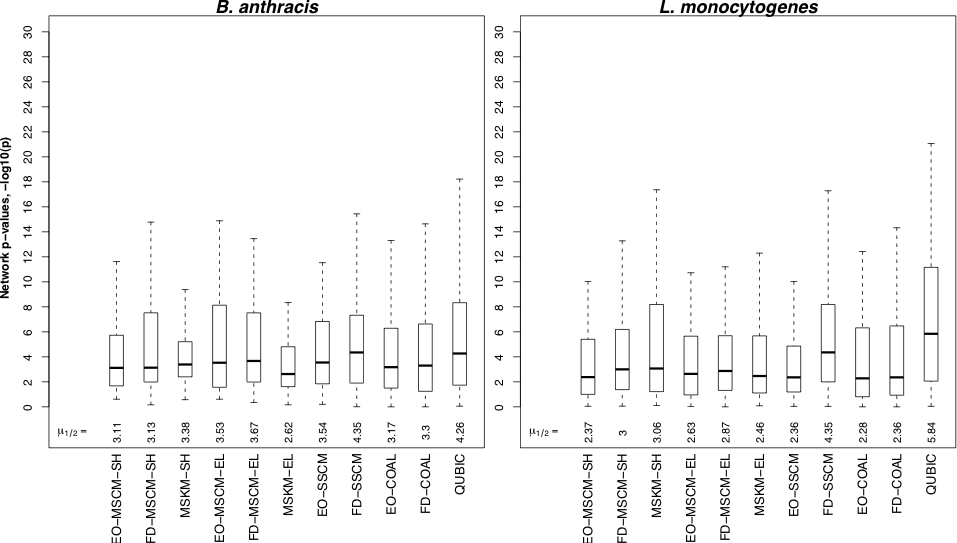
**

### Motif E-values

Figure S10: Motif E-values from the B. subtilis-B. anthracis pairing. The distributions of the motif E-values (-log_10_) from all methods considered by this study for the B. subtilis-B. anthracis pairing. Explanations of the method name abbreviations can be found in Table S1.

*
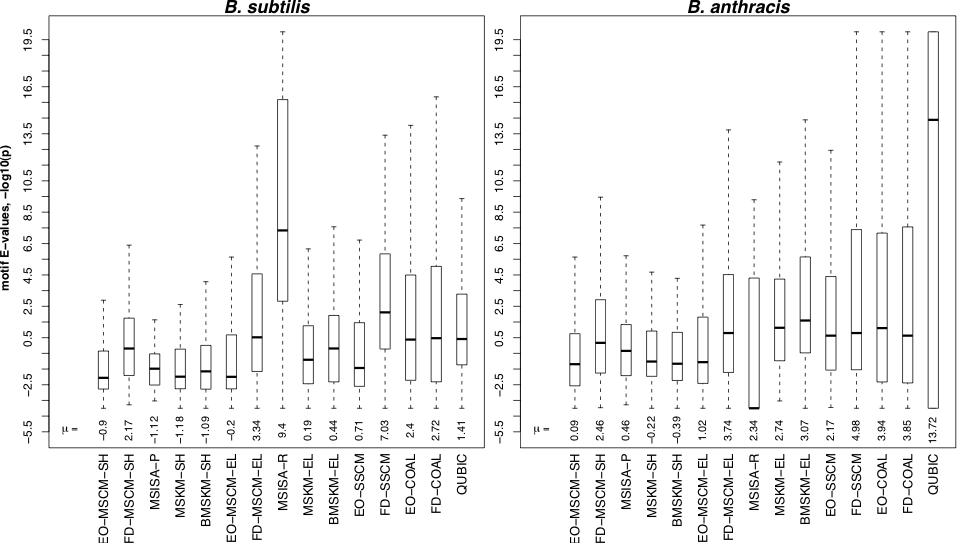
*

Figure S11: Motif E-values from the B. subtilis-L. monocytogenes pairing. The distributions of the motif E-values (-log_10_) from all methods considered by this study for the B. subtilis- L. monocytogenes pairing. Explanations of the method name abbreviations can be found in Table S1.

*
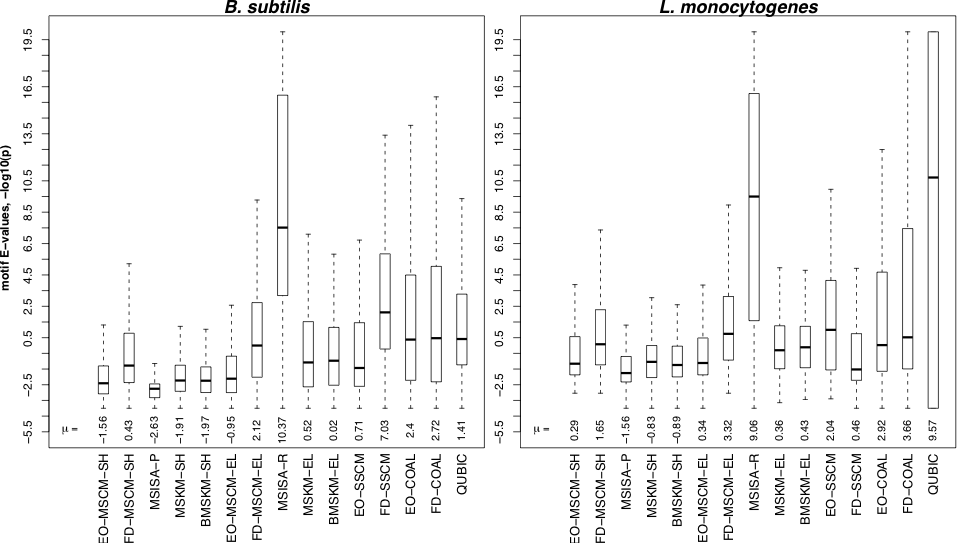
*

Figure S12: Motif E-values from the B. anthracis-L. monocytogenes pairing. The distributions of the motif E-values (-log_10_) from all methods considered by this study for the B. anthracis-L. monocytogenes pairing. Explanations of the method name abbreviations can be found in Table S1.


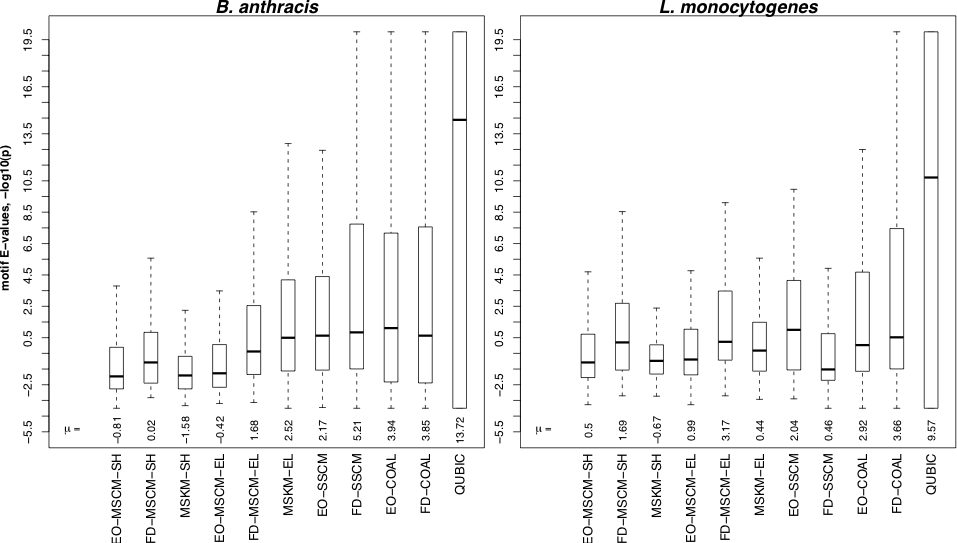


### Sequence p-values

Figure S13: Sequence p-values from the B. subtilis-B. anthracis pairing. The distributions of the sequence p-values (-log_10_) from all methods considered by this study for the B. subtilis-B. anthracis pairing. Explanations of the method name abbreviations can be found in Table S1.

*
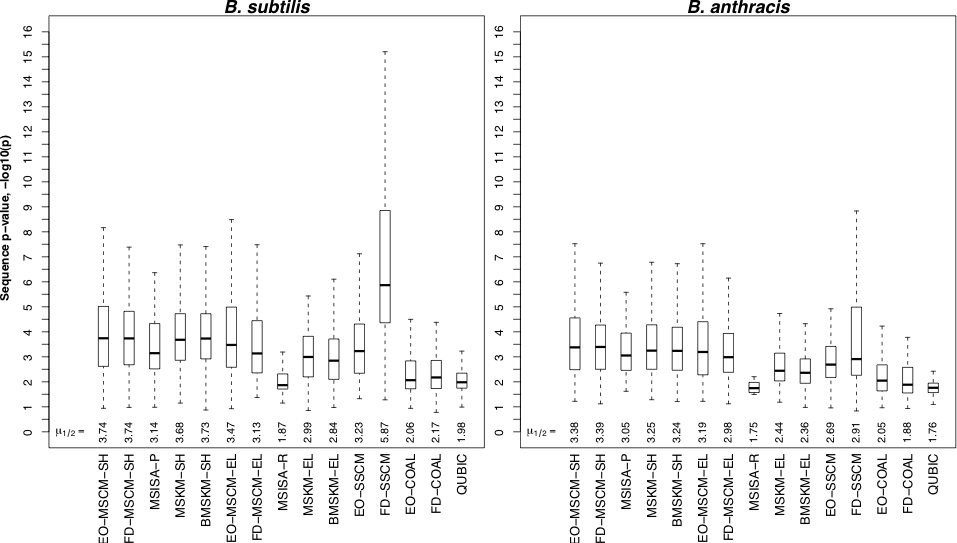
*

Figure S14: Sequence p-values from the B. subtilis-L. monocytogenes pairing. The distributions of the sequence p-values (-log_10_) from the multi-species cMonkey and k-means optimizations for the B. subtilis- L. monocytogenes pairing. Explanations of the method name abbreviations can be found in Table S1.

*
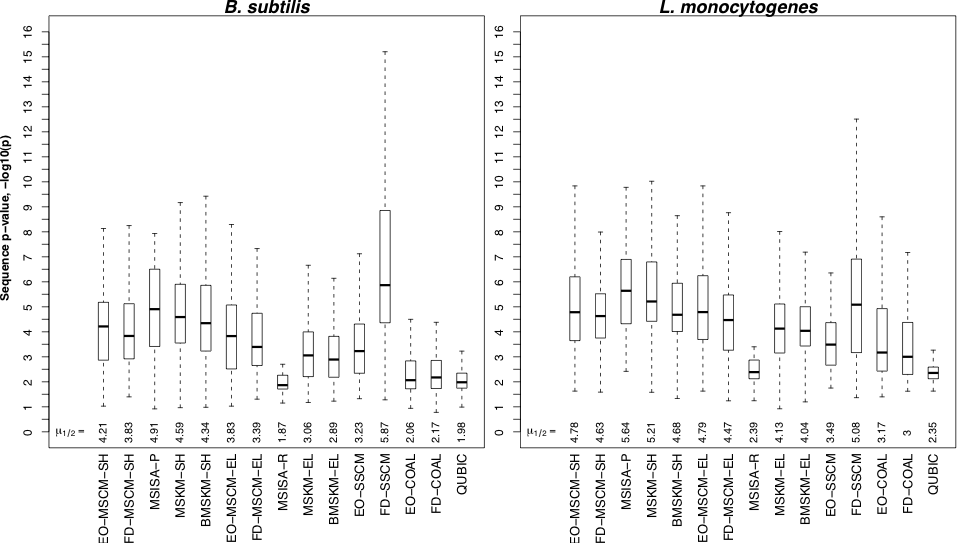
*

Figure S15: Sequence p-values from the B. anthracis-L. monocytogenes pairing. The distributions of the sequence p-values (-log_10_) from the multi-species cMonkey and k-means optimizations for the B. anthracis-L. monocytogenes pairing. Explanations of the method name abbreviations can be found in Table S1.


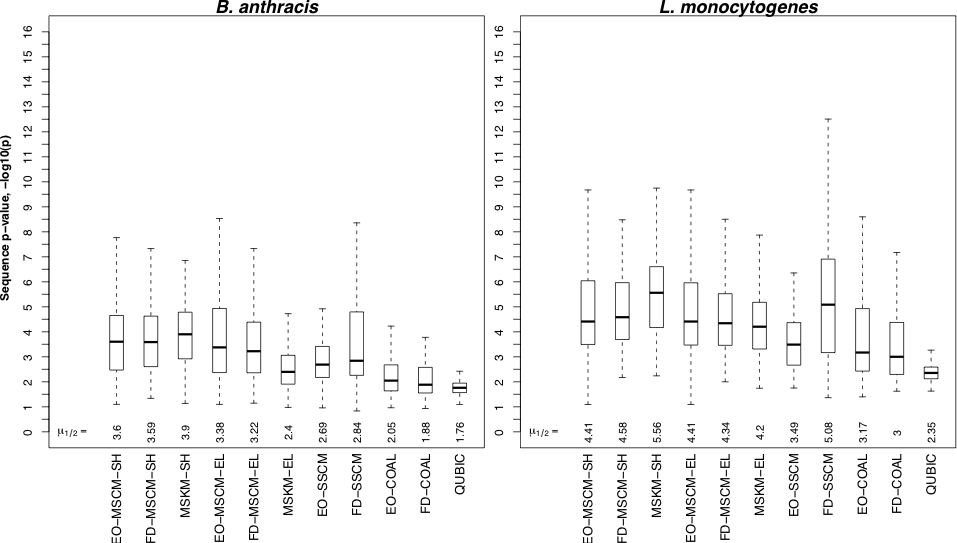


## Additional size distribution, overlap and coverage figures

### Number of genes

Figure S16: Number of genes from the B. subtilis – B. anthracis pairing. The distributions of the number of genes from all methods considered by this study for the B. subtilis- B. anthracis pairing. Explanations of the method name abbreviations can be found in Table S1.


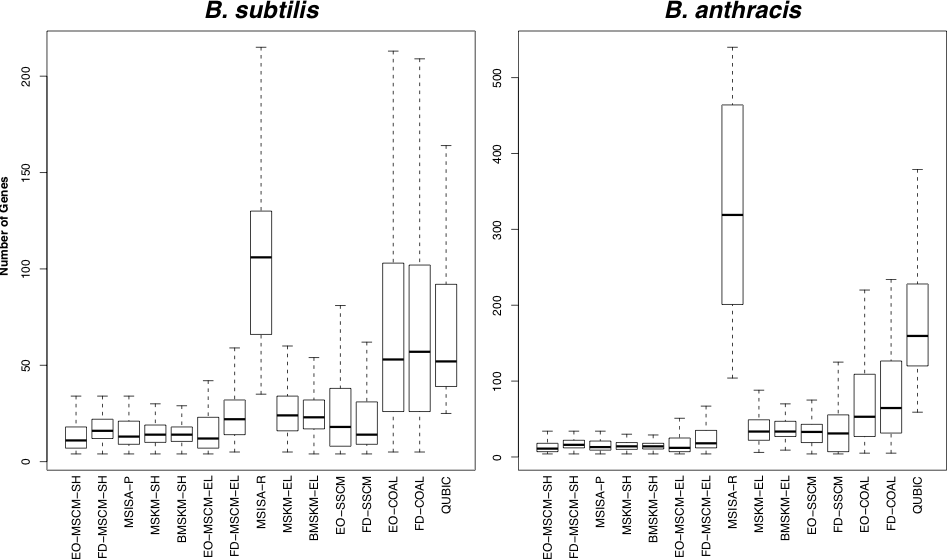


Figure S17: Number of genes from the B. subtilis – L. monocytogenes pairing. The distributions of the number of genes from all methods considered by this study for the B. subtilis- L. monocytogenes pairing. Explanations of the method name abbreviations can be found in Table S1.


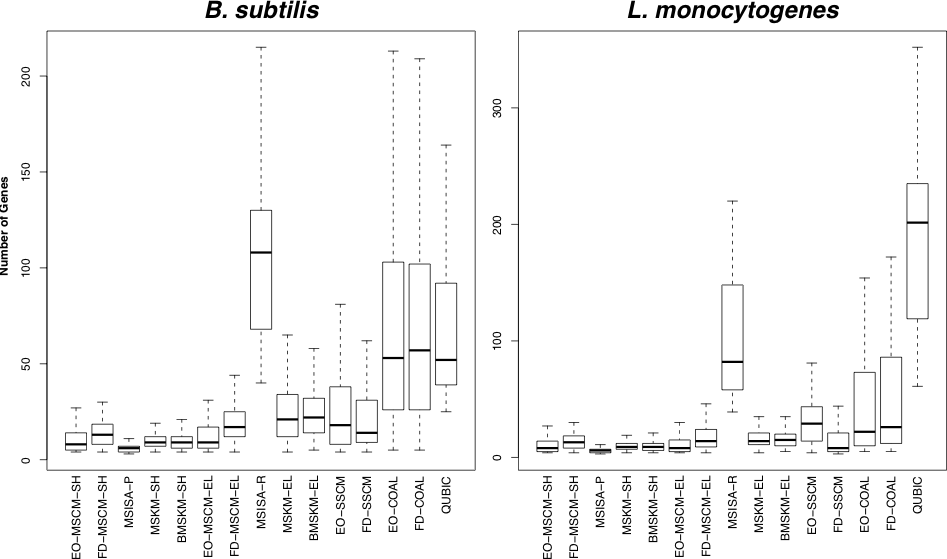


Figure S18: Number of genes from the B. anthracis – L. monocytogenes pairing. The distributions of the number of genes from all methods considered by this study for the B. anthracis – L. monocytogenes pairing. Explanations of the method name abbreviations can be found in Table S1.


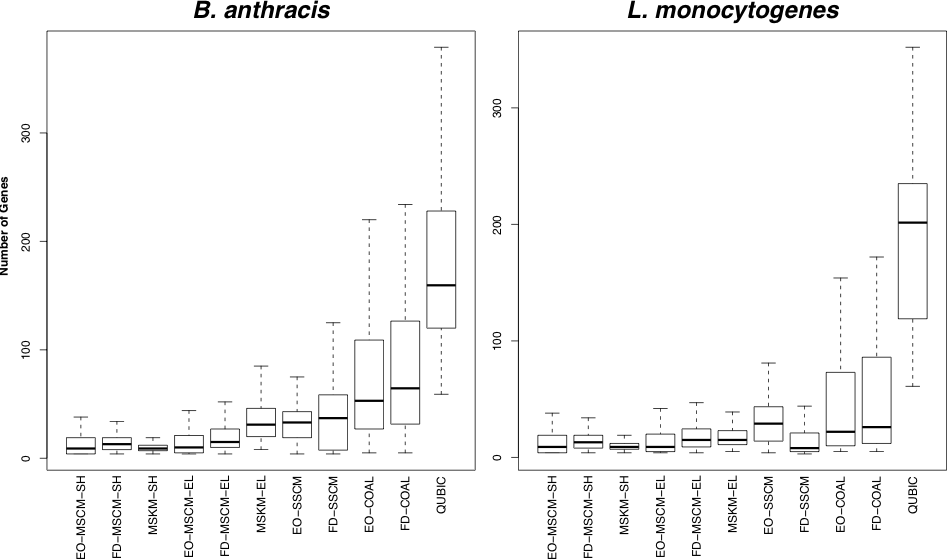


### Number of conditions

Figure S19: Number of conditions from the B. subtilis – B. anthracis pairing. The distributions of the number of conditions from all methods considered by this study for the B. subtilis- B. anthracis pairing. Explanations of the method name abbreviations can be found in Table S1.


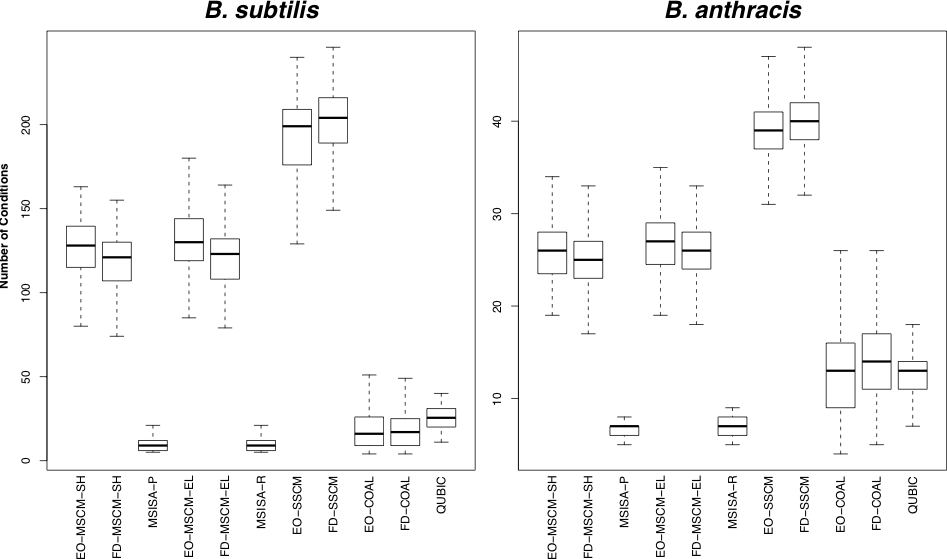


Figure S20: Number of conditions from the B. subtilis – L. monocytogenes pairing. The distributions of the number of conditions from all methods considered by this study for the B. subtilis- L. monocytogenes pairing. Explanations of the method name abbreviations can be found in Table S1.


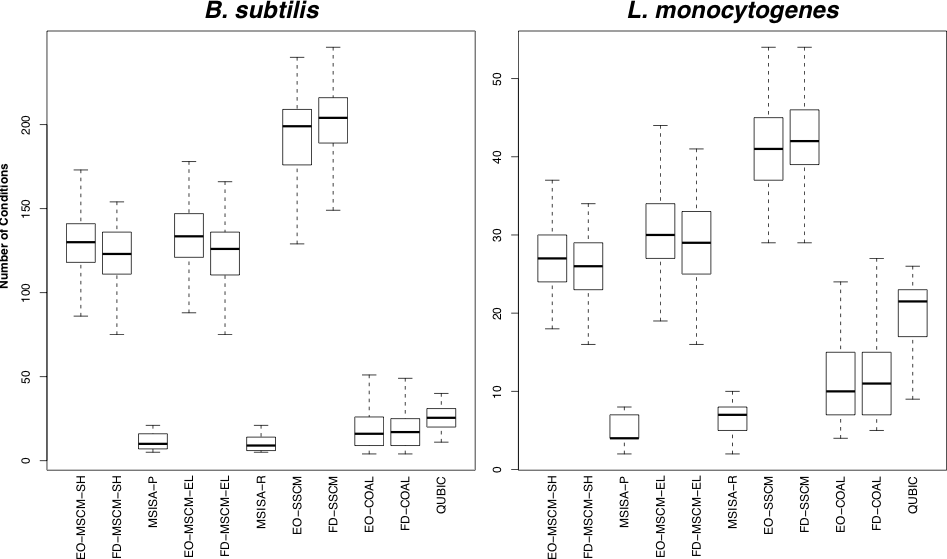


Figure S21: Number of conditions from the B. anthracis – L. monocytogenes pairing. The distributions of the number of conditions from all methods considered by this study for the B. anthracis – L. monocytogenes pairing. Explanations of the method name abbreviations can be found in Table S1.


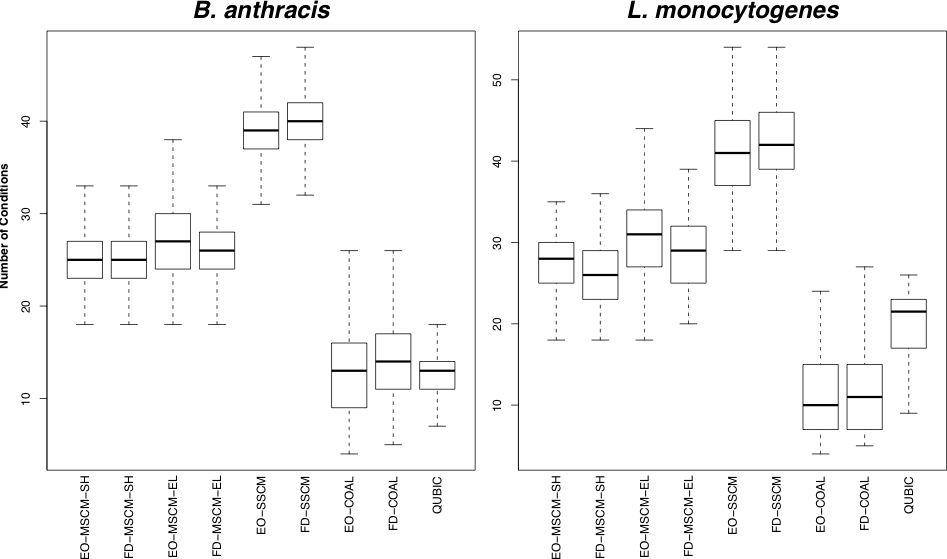


### Coverage (matrix element-wise)

Figure S22: Coverages (matrix element-wise) from the B. subtilis – B. anthracis pairing. The distributions of the Coverages (matrix element-wise) from all methods considered by this study for the B. subtilis- B. anthracis pairing. Explanations of the method name abbreviations can be found in Table S1.


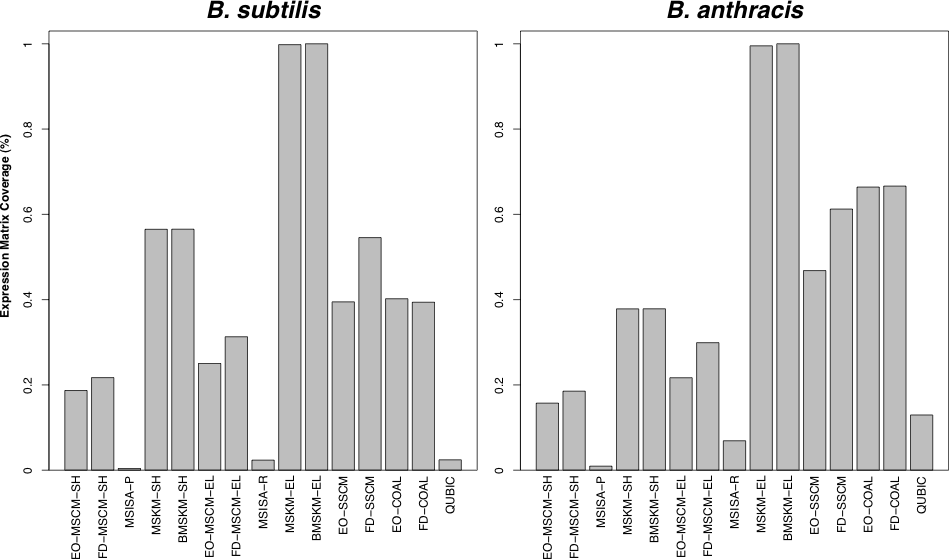


Figure S23: Coverages (matrix element-wise) from the B. subtilis – L. monocytogenes pairing. The distributions of the Coverages (matrix element-wise) from all methods considered by this study for the B. subtilis- L. monocytogenes pairing. Explanations of the method name abbreviations can be found in Table S1.


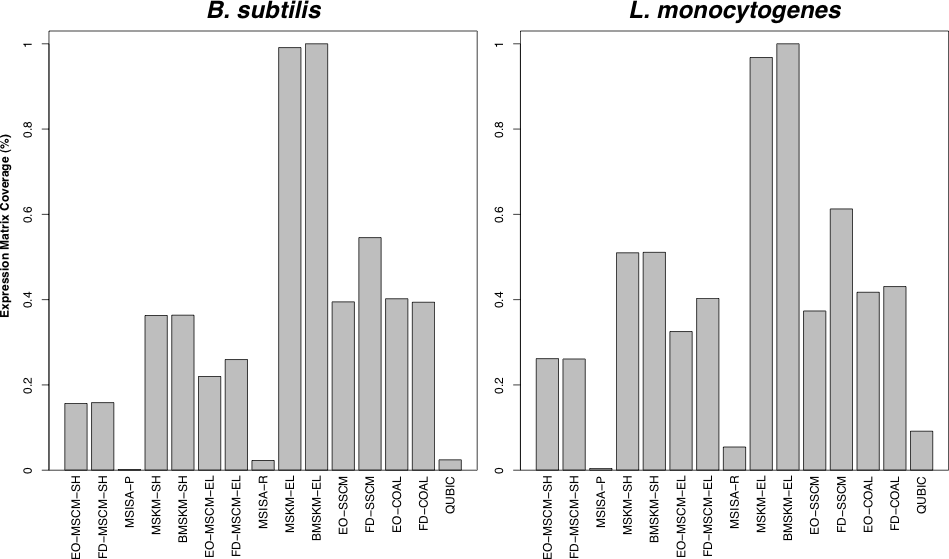


Figure S24: Coverages (matrix element-wise) from the B. anthracis – L. monocytogenes pairing. The distributions of the Coverages (matrix element-wise) from all methods considered by this study for the B. anthracis – L. monocytogenes pairing. Explanations of the method name abbreviations can be found in Table S1.


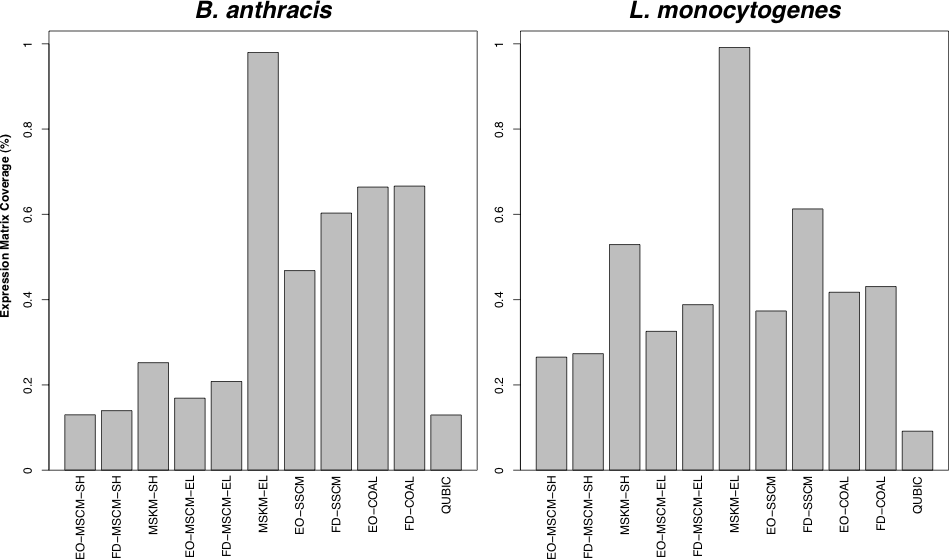


### Coverage (matrix gene-wise)

Figure S25: Coverages (gene-wise) from the B. subtilis – B. anthracis pairing. The distributions of the Coverages (gene-wise) from all methods considered by this study for the B. subtilis- B. anthracis pairing. Explanations of the method name abbreviations can be found in Table S1.


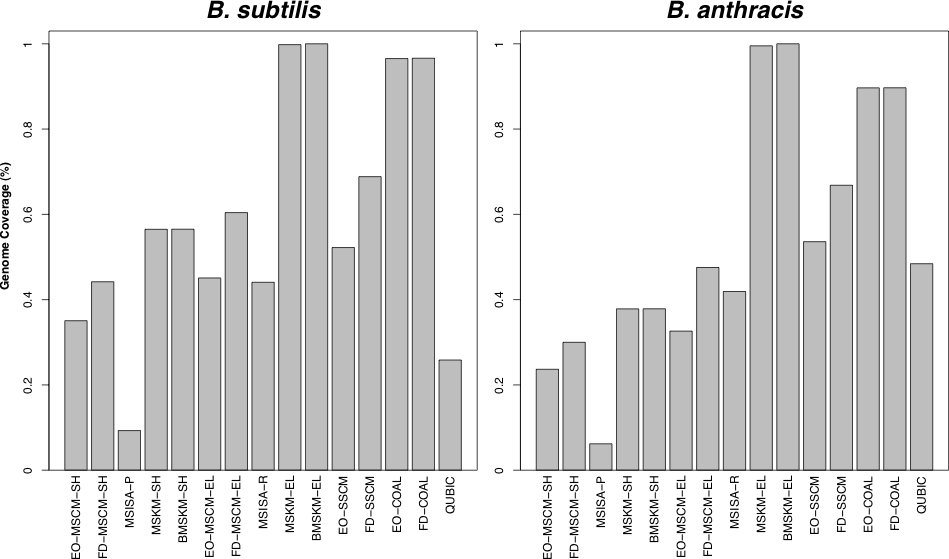


Figure S26: Coverages (gene-wise) from the B. subtilis – L. monocytogenes pairing. The distributions of the Coverages (gene-wise) from all methods considered by this study for the B. subtilis- L. monocytogenes pairing. Explanations of the method name abbreviations can be found in Table S1.


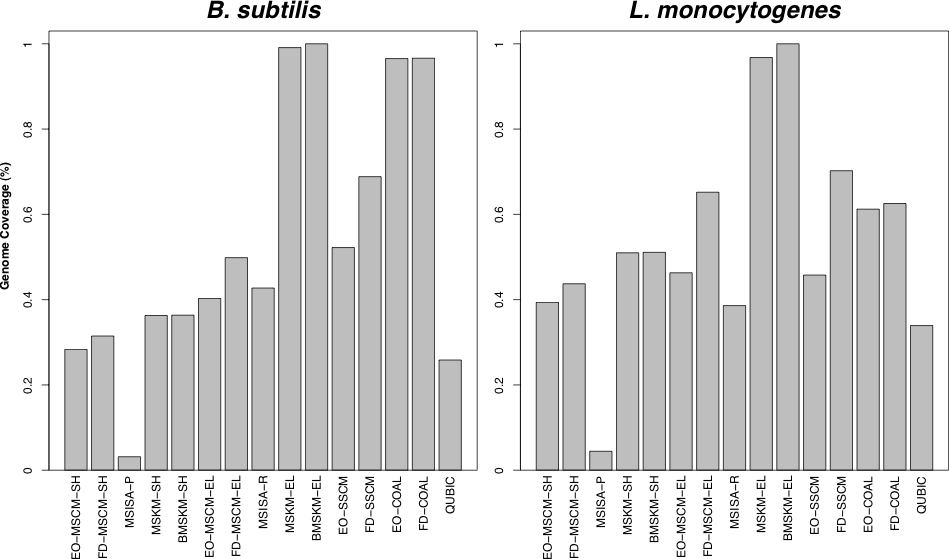


Figure S27: Coverages (gene-wise) from the B. anthracis – L. monocytogenes pairing. The distributions of the Coverages (gene-wise) from all methods considered by this study for the B. anthracis – L. monocytogenes pairing. Explanations of the method name abbreviations can be found in Table S1.


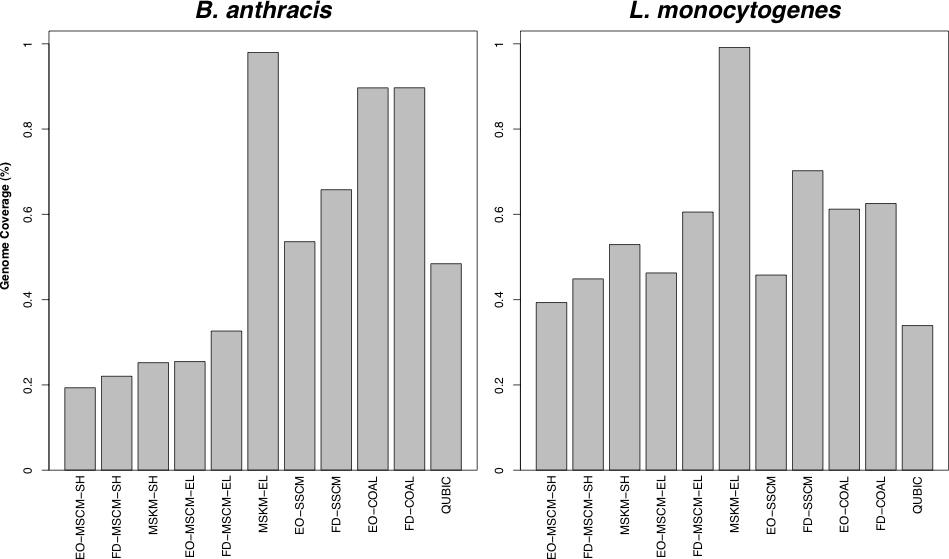


### Overlap (matrix element-wise)

Figure S28: Overlaps (matrix element-wise) from the B. subtilis – B. anthracis pairing. The distributions of the Overlaps (matrix element-wise) from all methods considered by this study for the B. subtilis- B. anthracis pairing. Explanations of the method name abbreviations can be found in Table S1.


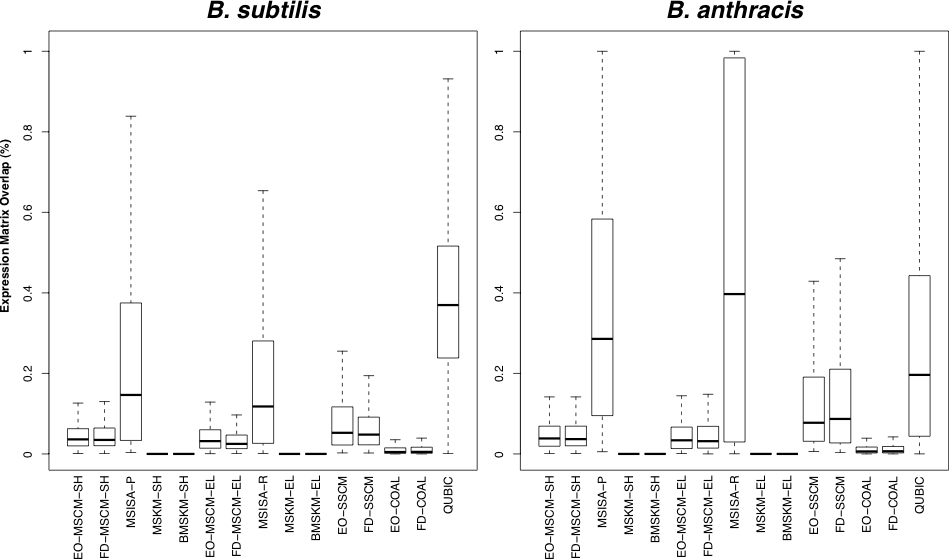


Figure S29: Overlaps (matrix element-wise) from the B. subtilis – L. monocytogenes pairing. The distributions of the Overlaps (matrix element-wise) from all methods considered by this study for the B. subtilis- L. monocytogenes pairing. Explanations of the method name abbreviations can be found in Table S1.


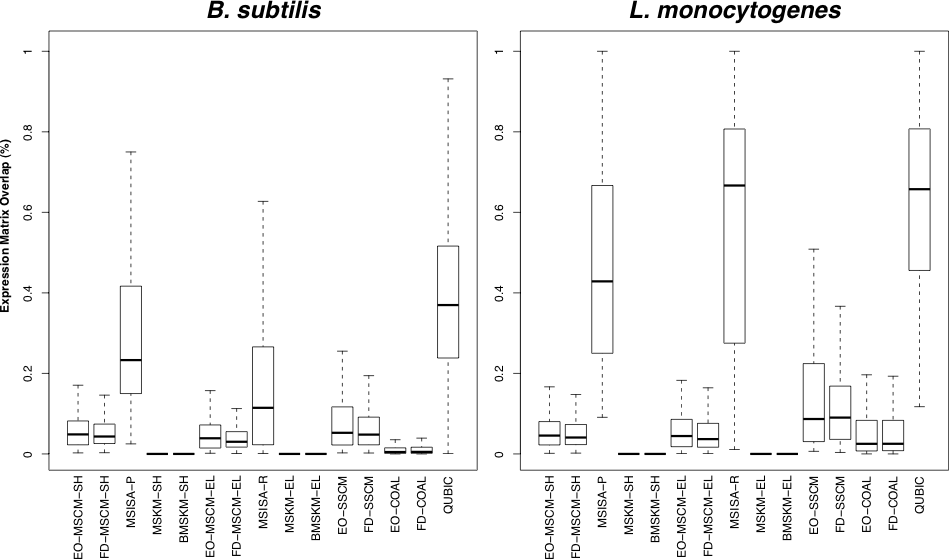


Figure S30: Overlaps (matrix element-wise) from the B. anthracis – L. monocytogenes pairing. The distributions of the Overlaps (matrix element-wise) from all methods considered by this study for the B. anthracis – L. monocytogenes pairing. Explanations of the method name abbreviations can be found in Table S1.


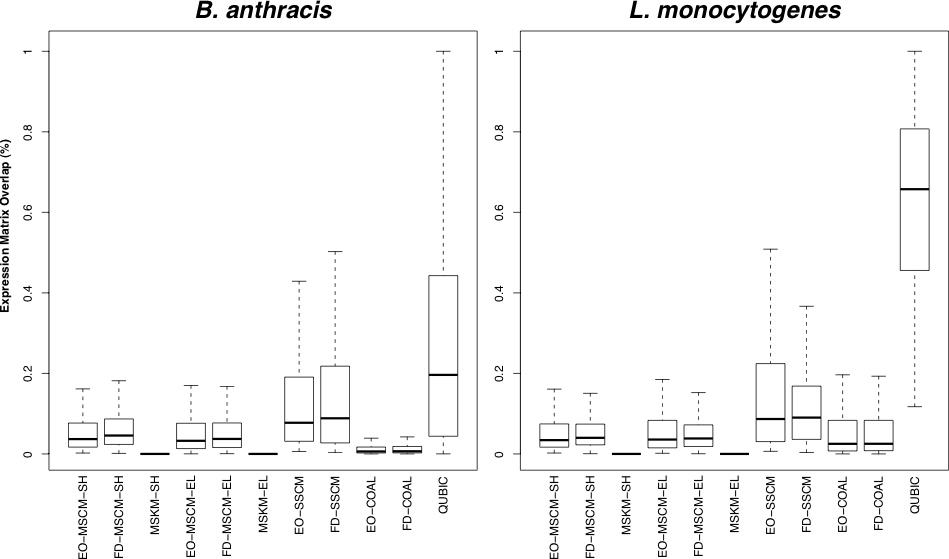


### Overlap (matrix gene-wise)

Figure S31: Overlaps (gene-wise) from the B. subtilis – B. anthracis pairing. The distributions of the Overlaps (gene-wise) from all methods considered by this study for the B. subtilis- B. anthracis pairing. Explanations of the method name abbreviations can be found in Table S1.


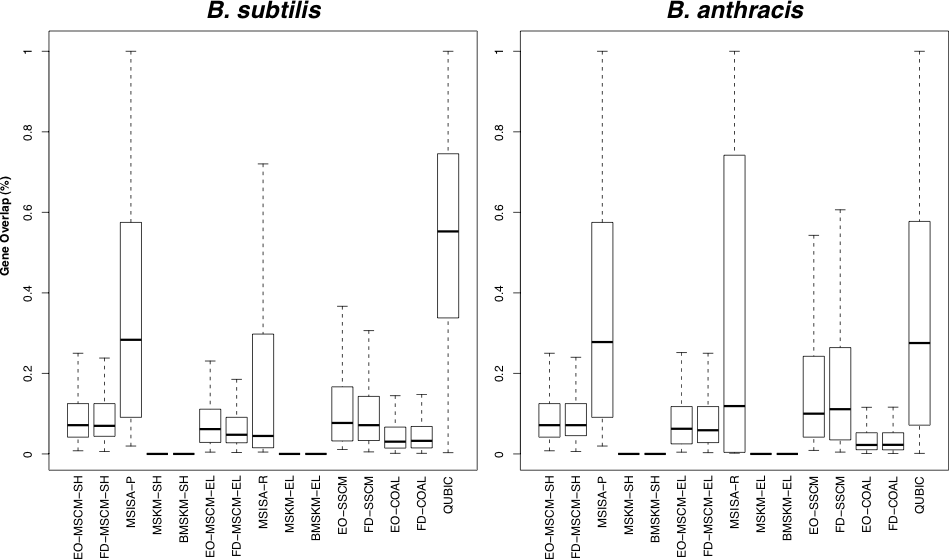


Figure S32: Overlaps (gene-wise) from the B. subtilis – L. monocytogenes pairing. The distributions of the Overlaps (gene-wise) from all methods considered by this study for the B. subtilis- L. monocytogenes pairing. Explanations of the method name abbreviations can be found in Table S1.


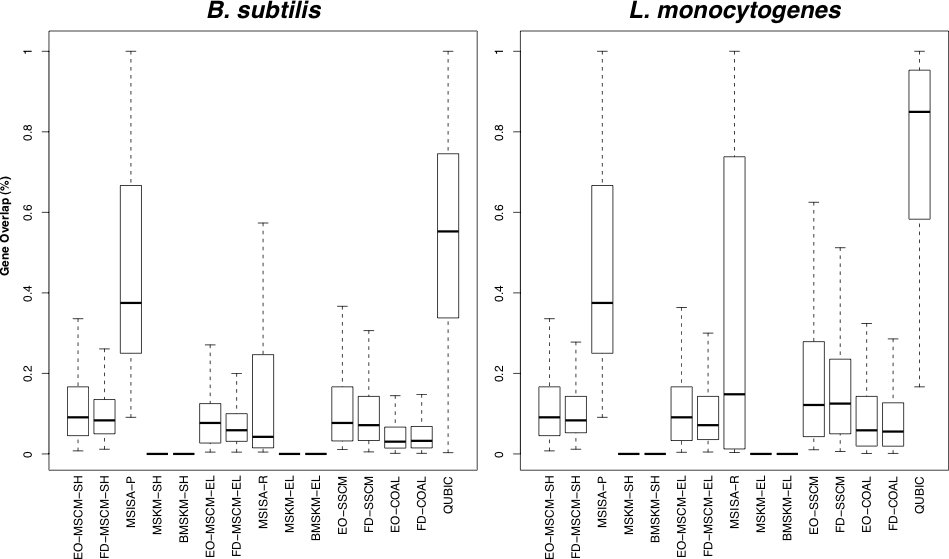


Figure S33: Overlaps (gene-wise) from the B. anthracis – L. monocytogenes pairing. The distributions of the Overlaps (gene-wise) from all methods considered by this study for the B. anthracis – L. monocytogenes pairing. Explanations of the method name abbreviations can be found in Table S1.


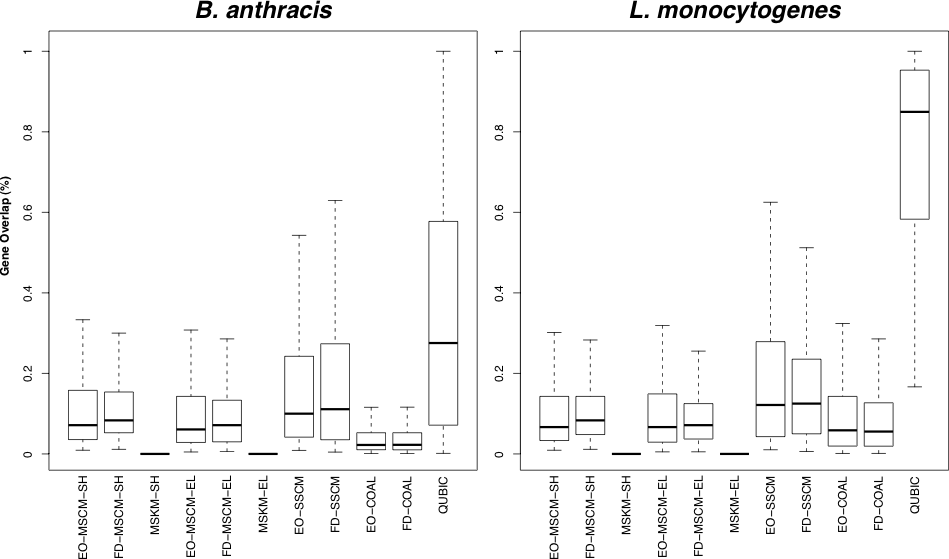


## Comparison of the (bi)cluster coherence metrics

### Comparisons with FD-MSCM

#### Residuals

Table S4: Comparison of bicluster residuals from the full data methods considered by this study for all pairings of B. subtilis, B. anthracis and L. monocytogenes. A comparison of the residuals of the results from MSCM (full data) with all other relevant methods for all 3 pairings of the three organisms examined. In the comparisons, we compare both MSCM steps to the other methods. Displayed are the means for each method and/or step compared, as well as the Wilcoxon’s non-parametric rank test (2-sided) comparing their distributions. We use ‘dist’ as an abbreviation for distribution, and the “dist1 vs. dist2” column to represent both the distributions being compared and their order in the table. Therefore, for example, the FD-MSCM-SH vs. FD-SSCM row displays the comparison of the distributions of residuals from the shared MS cMonkey results with those from the SS cMonkey, for the appropriate organism, with the FD-MSCM-SH as dist1 (and FD-SSCM as dist2). In addition, we color-code the Wilcoxon’s 2-sided column for a given organism to indicate whether the test indicated the distributions were the same or different, and if different, the distribution with the better overall score, as determined by the metric (Residuals). In this scheme, we use green to indicate dist1 had a statistically better score, red if dist2, and yellow to indicate a tie. Therefore, as the MSCM results are always the dist1 in these comparisons, this color scheme allows one to quickly and easily determine the overall frequency with which MSCM did as well or better than the other methods. In this case, these results illustrate that in 71 of the 92 comparisons (77.2%) MSCM step did as well or better than its competitors.

| ***B. subtilis - B. anthracis pairing*** |  | ***B. subtilis*** |  |  | ***B. anthracis*** | | |
| --- | --- | --- | --- | --- | --- | --- | --- |
| **dist1 vs. dist2** | **dist1 mean (green)** | **Wilcoxon's  2-sided** | **dist2 mean (red)** |  | **dist1 mean (green)** | **Wilcoxon's  2-sided** | **dist2 mean (red)** |
| **FD-MSCM-SH vs. FD-SSCM** | 0.51 ± 0.08 | **0.001** | 0.49 ± 0.13 |  | 0.30 ± 0.09 | **0.456** | 0.31 ± 0.12 |
| **FD-MSCM-SH vs. QUBIC** | 0.51 ± 0.08 | **3.69E-37** | 0.87 ± 0.21 |  | 0.30 ± 0.09 | **1.08E-50** | 1.51 ± 0.29 |
| **FD-MSCM-SH vs. FD-COAL** | 0.51 ± 0.08 | **9.94E-44** | 0.80 ± 0.25 |  | 0.30 ± 0.09 | **4.77E-38** | 0.58 ± 0.17 |
| **FD-MSCM-EL vs. FD-SSCM** | 0.49 ± 0.09 | **0.273** | 0.49 ± 0.13 |  | 0.32 ± 0.09 | **0.036** | 0.31 ± 0.12 |
| **FD-MSCM-EL vs. QUBIC** | 0.49 ± 0.09 | **5.60E-38** | 0.87 ± 0.21 |  | 0.32 ± 0.09 | **1.08E-50** | 1.51 ± 0.29 |
| **FD-MSCM-EL vs. FD-COAL** | 0.49 ± 0.09 | **6.10E-47** | 0.80 ± 0.25 |  | 0.32 ± 0.09 | **8.43E-36** | 0.58 ± 0.17 |
| **FD-MSCM-SH vs. MSISA-P** | 0.51 ± 0.08 | **2.62E-17** | 0.98 ± 0.39 |  | 0.30 ± 0.09 | **1.11E-22** | 1.97 ± 0.94 |
| **FD-MSCM-SH vs. MSISA-R** | 0.51 ± 0.08 | **5.99E-20** | 1.11 ± 0.41 |  | 0.30 ± 0.09 | **1.11E-22** | 1.58 ± 0.38 |
| **FD-MSCM-SH vs. MSKM-SH** | 0.51 ± 0.08 | **1.52E-25** | 0.41 ± 0.07 |  | 0.30 ± 0.09 | **7.05E-38** | 0.53 ± 0.12 |
| **FD-MSCM-SH vs. MSKM-EL** | 0.51 ± 0.08 | **4.31E-23** | 0.42 ± 0.06 |  | 0.30 ± 0.09 | **2.63E-33** | 0.48 ± 0.11 |
| **FD-MSCM-SH vs. BMSKM-SH** | 0.51 ± 0.08 | **2.68E-11** | 0.45 ± 0.07 |  | 0.30 ± 0.09 | **5.36E-16** | 0.38 ± 0.07 |
| **FD-MSCM-SH vs. BMSKM-EL** | 0.51 ± 0.08 | **1.84E-11** | 0.45 ± 0.06 |  | 0.30 ± 0.09 | **4.79E-18** | 0.39 ± 0.07 |
| **FD-MSCM-EL vs. MSISA-P** | 0.49 ± 0.09 | **9.21E-18** | 0.98 ± 0.39 |  | 0.32 ± 0.09 | **1.11E-22** | 1.97 ± 0.94 |
| **FD-MSCM-EL vs. MSISA-R** | 0.49 ± 0.09 | **5.02E-20** | 1.11 ± 0.41 |  | 0.32 ± 0.09 | **1.14E-22** | 1.58 ± 0.38 |
| **FD-MSCM-EL vs. MSKM-SH** | 0.49 ± 0.09 | **1.33E-17** | 0.41 ± 0.07 |  | 0.32 ± 0.09 | **2.40E-35** | 0.53 ± 0.12 |
| **FD-MSCM-EL vs. MSKM-EL** | 0.49 ± 0.09 | **4.38E-15** | 0.42 ± 0.06 |  | 0.32 ± 0.09 | **2.41E-29** | 0.48 ± 0.11 |
| **FD-MSCM-EL vs. BMSKM-SH** | 0.49 ± 0.09 | **6.91E-05** | 0.45 ± 0.07 |  | 0.32 ± 0.09 | **3.02E-11** | 0.38 ± 0.07 |
| **FD-MSCM-EL vs. BMSKM-EL** | 0.49 ± 0.09 | **1.30E-04** | 0.45 ± 0.06 |  | 0.32 ± 0.09 | **3.34E-13** | 0.39 ± 0.07 |
|  |  |  |  |  |  |  |  |
|  |  |  |  |  |  |  |  |
| ***B. subtilis - L. monocytogenes pairing*** |  | ***B. subtilis*** |  |  | ***L. monocytogenes*** | | |
| **dist1 vs. dist2** | **dist1 mean (green)** | **Wilcoxon's  2-sided** | **dist2 mean (red)** |  | **dist1 mean (green)** | **Wilcoxon's 2-sided** | **dist2 mean (red)** |
| **FD-MSCM-SH vs. FD-SSCM** | 0.52 ± 0.08 | **2.82E-04** | 0.49 ± 0.13 |  | 0.34 ± 0.12 | **0.006** | 0.40 ± 0.18 |
| **FD-MSCM-SH vs. QUBIC** | 0.52 ± 0.08 | **1.31E-36** | 0.87 ± 0.21 |  | 0.34 ± 0.12 | **5.94E-37** | 1.81 ± 0.85 |
| **FD-MSCM-SH vs. FD-COAL** | 0.52 ± 0.08 | **3.16E-42** | 0.80 ± 0.25 |  | 0.34 ± 0.12 | **1.91E-08** | 1.70 ± 3.24 |
| **FD-MSCM-EL vs. FD-SSCM** | 0.50 ± 0.10 | **0.088** | 0.49 ± 0.13 |  | 0.34 ± 0.12 | **0.004** | 0.40 ± 0.18 |
| **FD-MSCM-EL vs. QUBIC** | 0.50 ± 0.10 | **2.05E-36** | 0.87 ± 0.21 |  | 0.34 ± 0.12 | **3.99E-37** | 1.81 ± 0.85 |
| **FD-MSCM-EL vs. FD-COAL** | 0.50 ± 0.10 | **1.35E-44** | 0.80 ± 0.25 |  | 0.34 ± 0.12 | **1.11E-08** | 1.70 ± 3.24 |
| **FD-MSCM-SH vs. MSISA-P** | 0.52 ± 0.08 | **1.16E-09** | 0.87 ± 0.34 |  | 0.34 ± 0.12 | **3.21E-19** | 1.59 ± 0.52 |
| **FD-MSCM-SH vs. MSISA-R** | 0.52 ± 0.08 | **1.90E-18** | 1.11 ± 0.42 |  | 0.34 ± 0.12 | **2.30E-21** | 1.31 ± 0.34 |
| **FD-MSCM-SH vs. MSKM-SH** | 0.52 ± 0.08 | **1.07E-31** | 0.40 ± 0.07 |  | 0.34 ± 0.12 | **3.63E-22** | 0.50 ± 0.12 |
| **FD-MSCM-SH vs. MSKM-EL** | 0.52 ± 0.08 | **1.07E-25** | 0.42 ± 0.06 |  | 0.34 ± 0.12 | **4.44E-19** | 0.48 ± 0.11 |
| **FD-MSCM-SH vs. BMSKM-SH** | 0.52 ± 0.08 | **2.52E-19** | 0.43 ± 0.07 |  | 0.34 ± 0.12 | **1.37E-10** | 0.42 ± 0.09 |
| **FD-MSCM-SH vs. BMSKM-EL** | 0.52 ± 0.08 | **3.47E-17** | 0.44 ± 0.06 |  | 0.34 ± 0.12 | **2.35E-11** | 0.42 ± 0.09 |
| **FD-MSCM-EL vs. MSISA-P** | 0.50 ± 0.10 | **8.82E-10** | 0.87 ± 0.34 |  | 0.34 ± 0.12 | **3.21E-19** | 1.59 ± 0.52 |
| **FD-MSCM-EL vs. MSISA-R** | 0.50 ± 0.10 | **2.99E-18** | 1.11 ± 0.42 |  | 0.34 ± 0.12 | **2.30E-21** | 1.31 ± 0.34 |
| **FD-MSCM-EL vs. MSKM-SH** | 0.50 ± 0.10 | **2.23E-25** | 0.40 ± 0.07 |  | 0.34 ± 0.12 | **1.17E-22** | 0.50 ± 0.12 |
| **FD-MSCM-EL vs. MSKM-EL** | 0.50 ± 0.10 | **1.13E-18** | 0.42 ± 0.06 |  | 0.34 ± 0.12 | **1.32E-19** | 0.48 ± 0.11 |
| **FD-MSCM-EL vs. BMSKM-SH** | 0.50 ± 0.10 | **6.56E-13** | 0.43 ± 0.07 |  | 0.34 ± 0.12 | **7.95E-11** | 0.42 ± 0.09 |
| **FD-MSCM-EL vs. BMSKM-EL** | 0.50 ± 0.10 | **8.10E-11** | 0.44 ± 0.06 |  | 0.34 ± 0.12 | **8.42E-12** | 0.42 ± 0.09 |
|  |  |  |  |  |  |  |  |
|  |  |  |  |  |  |  |  |
| ***B. anthracis - L. monocytogenes pairing*** | ***B. anthracis*** | | |  | ***L. monocytogenes*** | | |
| **dist1 vs. dist2** | **dist1 mean (green)** | **Wilcoxon's  2-sided** | **dist2 mean (red)** |  | **dist1 mean (green)** | **Wilcoxon's  2-sided** | **dist2 mean (red)** |
| **FD-MSCM-SH vs. FD-SSCM** | 0.33 ± 0.10 | **0.004** | 0.31 ± 0.12 |  | 0.36 ± 0.14 | **0.097** | 0.40 ± 0.18 |
| **FD-MSCM-SH vs. QUBIC** | 0.33 ± 0.10 | **2.30E-50** | 1.51 ± 0.29 |  | 0.36 ± 0.14 | **4.38E-35** | 1.81 ± 0.85 |
| **FD-MSCM-SH vs. FD-COAL** | 0.33 ± 0.10 | **1.34E-33** | 0.58 ± 0.17 |  | 0.36 ± 0.14 | **1.83E-06** | 1.70 ± 3.24 |
| **FD-MSCM-EL vs. FD-SSCM** | 0.36 ± 0.11 | **2.95E-07** | 0.31 ± 0.12 |  | 0.36 ± 0.13 | **0.049** | 0.40 ± 0.18 |
| **FD-MSCM-EL vs. QUBIC** | 0.36 ± 0.11 | **2.30E-50** | 1.51 ± 0.29 |  | 0.36 ± 0.13 | **8.30E-36** | 1.81 ± 0.85 |
| **FD-MSCM-EL vs. FD-COAL** | 0.36 ± 0.11 | **8.77E-28** | 0.58 ± 0.17 |  | 0.36 ± 0.13 | **4.48E-07** | 1.70 ± 3.24 |
| **FD-MSCM-SH vs. MSKM-SH** | 0.33 ± 0.10 | **2.89E-11** | 0.40 ± 0.08 |  | 0.36 ± 0.14 | **2.64E-10** | 0.43 ± 0.08 |
| **FD-MSCM-SH vs. MSKM-EL** | 0.33 ± 0.10 | **3.35E-11** | 0.39 ± 0.07 |  | 0.36 ± 0.14 | **3.47E-09** | 0.43 ± 0.08 |
| **FD-MSCM-EL vs. MSKM-SH** | 0.36 ± 0.11 | **5.90E-05** | 0.40 ± 0.08 |  | 0.36 ± 0.13 | **8.73E-12** | 0.43 ± 0.08 |
| **FD-MSCM-EL vs. MSKM-EL** | 0.36 ± 0.11 | **9.70E-05** | 0.39 ± 0.07 |  | 0.36 ± 0.13 | **1.27E-10** | 0.43 ± 0.08 |

#### Mean correlations

Table S5: Comparison of bicluster mean correlations from the full data methods considered by this study for all pairings of B. subtilis, B. anthracis and L. monocytogenes. A comparison of the mean correlations of the results from MSCM (full data) with all other relevant methods for all 3 pairings of the three organisms examined. In the comparisons, we compare both MSCM steps to the other methods. Displayed are the means for each method and/or step compared, as well as the Wilcoxon’s non-parametric rank test (2-sided) comparing their distributions. We direct the reader to 4 for instructions on how to interpret the table. In this case, these results illustrate that in 92 of the 92 comparisons (100%) MSCM step did as well or better than its competitors.

| ***B. subtilis - B. anthracis pairing*** |  | ***B. subtilis*** |  |  | ***B. anthracis*** | | |
| --- | --- | --- | --- | --- | --- | --- | --- |
| **dist1 vs. dist2** | **dist1 mean (green)** | **Wilcoxon's  2-sided** | **dist2 mean (red)** |  | **dist1 mean (green)** | **Wilcoxon's 2-sided** | **dist2 mean (red)** |
| **FD-MSCM-SH vs. FD-SSCM** | 0.59 ± 0.11 | **0.013** | 0.56 ± 0.14 |  | 0.85 ± 0.09 | **0.351** | 0.82 ± 0.15 |
| **FD-MSCM-SH vs. QUBIC** | 0.59 ± 0.11 | **7.33E-28** | 0.36 ± 0.19 |  | 0.85 ± 0.09 | **1.85E-50** | 0.49 ± 0.05 |
| **FD-MSCM-SH vs. FD-COAL** | 0.59 ± 0.11 | **0.446** | 0.59 ± 0.15 |  | 0.85 ± 0.09 | **4.87E-35** | 0.62 ± 0.13 |
| **FD-MSCM-EL vs. FD-SSCM** | 0.61 ± 0.11 | **3.11E-04** | 0.56 ± 0.14 |  | 0.84 ± 0.09 | **0.760** | 0.82 ± 0.15 |
| **FD-MSCM-EL vs. QUBIC** | 0.61 ± 0.11 | **5.14E-29** | 0.36 ± 0.19 |  | 0.84 ± 0.09 | **1.37E-50** | 0.49 ± 0.05 |
| **FD-MSCM-EL vs. FD-COAL** | 0.61 ± 0.11 | **0.067** | 0.59 ± 0.15 |  | 0.84 ± 0.09 | **1.89E-33** | 0.62 ± 0.13 |
| **FD-MSCM-SH vs. MSISA-P** | 0.59 ± 0.11 | **0.963** | 0.60 ± 0.14 |  | 0.85 ± 0.09 | **9.59E-22** | 0.56 ± 0.07 |
| **FD-MSCM-SH vs. MSISA-R** | 0.59 ± 0.11 | **0.041** | 0.55 ± 0.13 |  | 0.85 ± 0.09 | **1.38E-22** | 0.51 ± 0.03 |
| **FD-MSCM-SH vs. MSKM-SH** | 0.59 ± 0.11 | **0.340** | 0.58 ± 0.11 |  | 0.85 ± 0.09 | **1.49E-43** | 0.52 ± 0.14 |
| **FD-MSCM-SH vs. MSKM-EL** | 0.59 ± 0.11 | **0.020** | 0.56 ± 0.11 |  | 0.85 ± 0.09 | **1.33E-40** | 0.58 ± 0.15 |
| **FD-MSCM-SH vs. BMSKM-SH** | 0.59 ± 0.11 | **1.11E-11** | 0.49 ± 0.13 |  | 0.85 ± 0.09 | **5.18E-25** | 0.72 ± 0.10 |
| **FD-MSCM-SH vs. BMSKM-EL** | 0.59 ± 0.11 | **1.17E-10** | 0.50 ± 0.12 |  | 0.85 ± 0.09 | **9.12E-26** | 0.71 ± 0.10 |
| **FD-MSCM-EL vs. MSISA-P** | 0.61 ± 0.11 | **0.500** | 0.60 ± 0.14 |  | 0.84 ± 0.09 | **7.49E-22** | 0.56 ± 0.07 |
| **FD-MSCM-EL vs. MSISA-R** | 0.61 ± 0.11 | **0.009** | 0.55 ± 0.13 |  | 0.84 ± 0.09 | **1.18E-22** | 0.51 ± 0.03 |
| **FD-MSCM-EL vs. MSKM-SH** | 0.61 ± 0.11 | **0.035** | 0.58 ± 0.11 |  | 0.84 ± 0.09 | **4.07E-43** | 0.52 ± 0.14 |
| **FD-MSCM-EL vs. MSKM-EL** | 0.61 ± 0.11 | **0.001** | 0.56 ± 0.11 |  | 0.84 ± 0.09 | **1.01E-39** | 0.58 ± 0.15 |
| **FD-MSCM-EL vs. BMSKM-SH** | 0.61 ± 0.11 | **2.10E-14** | 0.49 ± 0.13 |  | 0.84 ± 0.09 | **7.87E-23** | 0.72 ± 0.10 |
| **FD-MSCM-EL vs. BMSKM-EL** | 0.61 ± 0.11 | **3.51E-13** | 0.50 ± 0.12 |  | 0.84 ± 0.09 | **1.31E-23** | 0.71 ± 0.10 |
|  |  |  |  |  |  |  |  |
|  |  |  |  |  |  |  |  |
| ***B. subtilis - L. monocytogenes pairing*** |  | ***B. subtilis*** |  |  | ***L. monocytogenes*** | | |
| **dist1 vs. dist2** | **dist1 mean (green)** | **Wilcoxon's  2-sided** | **dist2 mean (red)** |  | **dist1 mean (green)** | **Wilcoxon's 2-sided** | **dist2 mean (red)** |
| **FD-MSCM-SH vs. FD-SSCM** | 0.59 ± 0.11 | **0.011** | 0.56 ± 0.14 |  | 0.80 ± 0.13 | **2.31E-04** | 0.71 ± 0.20 |
| **FD-MSCM-SH vs. QUBIC** | 0.59 ± 0.11 | **5.45E-27** | 0.36 ± 0.19 |  | 0.80 ± 0.13 | **2.92E-18** | 0.45 ± 0.27 |
| **FD-MSCM-SH vs. FD-COAL** | 0.59 ± 0.11 | **0.481** | 0.59 ± 0.15 |  | 0.80 ± 0.13 | **0.999** | 0.80 ± 0.12 |
| **FD-MSCM-EL vs. FD-SSCM** | 0.61 ± 0.10 | **2.58E-04** | 0.56 ± 0.14 |  | 0.81 ± 0.11 | **3.00E-05** | 0.71 ± 0.20 |
| **FD-MSCM-EL vs. QUBIC** | 0.61 ± 0.10 | **1.36E-28** | 0.36 ± 0.19 |  | 0.81 ± 0.11 | **3.29E-18** | 0.45 ± 0.27 |
| **FD-MSCM-EL vs. FD-COAL** | 0.61 ± 0.10 | **0.064** | 0.59 ± 0.15 |  | 0.81 ± 0.11 | **0.674** | 0.80 ± 0.12 |
| **FD-MSCM-SH vs. MSISA-P** | 0.59 ± 0.11 | **0.639** | 0.60 ± 0.20 |  | 0.80 ± 0.13 | **5.34E-13** | 0.47 ± 0.23 |
| **FD-MSCM-SH vs. MSISA-R** | 0.59 ± 0.11 | **0.036** | 0.55 ± 0.12 |  | 0.80 ± 0.13 | **4.18E-08** | 0.50 ± 0.27 |
| **FD-MSCM-SH vs. MSKM-SH** | 0.59 ± 0.11 | **0.716** | 0.59 ± 0.11 |  | 0.80 ± 0.13 | **8.05E-33** | 0.51 ± 0.17 |
| **FD-MSCM-SH vs. MSKM-EL** | 0.59 ± 0.11 | **0.021** | 0.56 ± 0.11 |  | 0.80 ± 0.13 | **4.99E-29** | 0.55 ± 0.16 |
| **FD-MSCM-SH vs. BMSKM-SH** | 0.59 ± 0.11 | **6.43E-06** | 0.52 ± 0.14 |  | 0.80 ± 0.13 | **5.58E-20** | 0.63 ± 0.15 |
| **FD-MSCM-SH vs. BMSKM-EL** | 0.59 ± 0.11 | **1.28E-05** | 0.53 ± 0.12 |  | 0.80 ± 0.13 | **9.89E-19** | 0.64 ± 0.14 |
| **FD-MSCM-EL vs. MSISA-P** | 0.61 ± 0.10 | **0.440** | 0.60 ± 0.20 |  | 0.81 ± 0.11 | **1.28E-13** | 0.47 ± 0.23 |
| **FD-MSCM-EL vs. MSISA-R** | 0.61 ± 0.10 | **0.004** | 0.55 ± 0.12 |  | 0.81 ± 0.11 | **1.11E-08** | 0.50 ± 0.27 |
| **FD-MSCM-EL vs. MSKM-SH** | 0.61 ± 0.10 | **0.347** | 0.59 ± 0.11 |  | 0.81 ± 0.11 | **1.47E-34** | 0.51 ± 0.17 |
| **FD-MSCM-EL vs. MSKM-EL** | 0.61 ± 0.10 | **2.30E-04** | 0.56 ± 0.11 |  | 0.81 ± 0.11 | **3.83E-31** | 0.55 ± 0.16 |
| **FD-MSCM-EL vs. BMSKM-SH** | 0.61 ± 0.10 | **3.53E-08** | 0.52 ± 0.14 |  | 0.81 ± 0.11 | **3.86E-22** | 0.63 ± 0.15 |
| **FD-MSCM-EL vs. BMSKM-EL** | 0.61 ± 0.10 | **4.23E-08** | 0.53 ± 0.12 |  | 0.81 ± 0.11 | **9.29E-21** | 0.64 ± 0.14 |
|  |  |  |  |  |  |  |  |
|  |  |  |  |  |  |  |  |
| ***B. anthracis - L. monocytogenes pairing*** | ***B. anthracis*** | | |  | ***L. monocytogenes*** | | |
| **dist1 vs. dist2** | **dist1 mean (green)** | **Wilcoxon's  2-sided** | **dist2 mean (red)** |  | **dist1 mean (green)** | **Wilcoxon's 2-sided** | **dist2 mean (red)** |
| **FD-MSCM-SH vs. FD-SSCM** | 0.82 ± 0.11 | **0.227** | 0.82 ± 0.15 |  | 0.77 ± 0.14 | **0.011** | 0.71 ± 0.20 |
| **FD-MSCM-SH vs. QUBIC** | 0.82 ± 0.11 | **5.78E-49** | 0.49 ± 0.05 |  | 0.77 ± 0.14 | **1.03E-17** | 0.45 ± 0.27 |
| **FD-MSCM-SH vs. FD-COAL** | 0.82 ± 0.11 | **4.30E-29** | 0.62 ± 0.13 |  | 0.77 ± 0.14 | **0.344** | 0.80 ± 0.12 |
| **FD-MSCM-EL vs. FD-SSCM** | 0.80 ± 0.11 | **3.92E-04** | 0.82 ± 0.15 |  | 0.78 ± 0.13 | **0.003** | 0.71 ± 0.20 |
| **FD-MSCM-EL vs. QUBIC** | 0.80 ± 0.11 | **2.76E-48** | 0.49 ± 0.05 |  | 0.78 ± 0.13 | **1.22E-17** | 0.45 ± 0.27 |
| **FD-MSCM-EL vs. FD-COAL** | 0.80 ± 0.11 | **1.34E-24** | 0.62 ± 0.13 |  | 0.78 ± 0.13 | **0.532** | 0.80 ± 0.12 |
| **FD-MSCM-SH vs. MSKM-SH** | 0.82 ± 0.11 | **7.41E-21** | 0.69 ± 0.12 |  | 0.77 ± 0.14 | **6.55E-19** | 0.60 ± 0.14 |
| **FD-MSCM-SH vs. MSKM-EL** | 0.82 ± 0.11 | **6.40E-20** | 0.70 ± 0.10 |  | 0.77 ± 0.14 | **1.47E-16** | 0.63 ± 0.13 |
| **FD-MSCM-EL vs. MSKM-SH** | 0.80 ± 0.11 | **1.09E-14** | 0.69 ± 0.12 |  | 0.78 ± 0.13 | **9.32E-22** | 0.60 ± 0.14 |
| **FD-MSCM-EL vs. MSKM-EL** | 0.80 ± 0.11 | **8.90E-14** | 0.70 ± 0.10 |  | 0.78 ± 0.13 | **4.40E-19** | 0.63 ± 0.13 |

#### Network Association p-values

Table S6: Comparison of bicluster network association p-values from the full data methods considered by this study for all pairings of B. subtilis, B. anthracis and L. monocytogenes. A comparison of the association p-values (-log10) from MSCM (full data) with all other relevant methods for all 3 pairings of the three organisms examined. In the comparisons, we compare both MSCM steps to the other methods. Displayed are the means for each method and/or step compared, as well as the Wilcoxon’s non-parametric rank test (2-sided) comparing their distributions. We direct the reader to 4 for instructions on how to interpret the table. As the table indicates, in 77 of the 92 comparisons (83.7%) MSCM does as well or better than its competitors.

| ***B. subtilis - B. anthracis pairing*** |  | ***B. subtilis*** |  |  | ***B. anthracis*** | | |
| --- | --- | --- | --- | --- | --- | --- | --- |
| **dist1 vs. dist2** | **dist1 mean (green)** | **Wilcoxon's  2-sided** | **dist2 mean (red)** |  | **dist1 mean (green)** | **Wilcoxon's  2-sided** | **dist2 mean (red)** |
| **FD-MSCM-SH vs. FD-SSCM** | 7.82 ± 8.76 | **0.009** | 9.78 ± 9.76 |  | 6.16 ± 7.23 | **0.935** | 5.44 ± 5.38 |
| **FD-MSCM-SH vs. QUBIC** | 7.82 ± 8.76 | **5.42E-36** | 2.52 ± 4.78 |  | 6.16 ± 7.23 | **0.658** | 6.73 ± 7.52 |
| **FD-MSCM-SH vs. FD-COAL** | 7.82 ± 8.76 | **0.042** | 7.57 ± 9.16 |  | 6.16 ± 7.23 | **0.132** | 6.50 ± 8.74 |
| **FD-MSCM-EL vs. FD-SSCM** | 7.79 ± 9.05 | **0.001** | 9.78 ± 9.76 |  | 6.38 ± 7.19 | **0.753** | 5.44 ± 5.38 |
| **FD-MSCM-EL vs. QUBIC** | 7.79 ± 9.05 | **1.09E-35** | 2.52 ± 4.78 |  | 6.38 ± 7.19 | **0.853** | 6.73 ± 7.52 |
| **FD-MSCM-EL vs. FD-COAL** | 7.79 ± 9.05 | **0.114** | 7.57 ± 9.16 |  | 6.38 ± 7.19 | **0.098** | 6.50 ± 8.74 |
| **FD-MSCM-SH vs. MSISA-P** | 7.82 ± 8.76 | **0.300** | 5.56 ± 5.86 |  | 6.16 ± 7.23 | **0.834** | 5.61 ± 7.17 |
| **FD-MSCM-SH vs. MSISA-R** | 7.82 ± 8.76 | **0.041** | 9.69 ± 9.37 |  | 6.16 ± 7.23 | **0.186** | 9.66 ± 9.95 |
| **FD-MSCM-SH vs. MSKM-SH** | 7.82 ± 8.76 | **0.416** | 7.87 ± 9.35 |  | 6.16 ± 7.23 | **0.084** | 4.38 ± 5.10 |
| **FD-MSCM-SH vs. MSKM-EL** | 7.82 ± 8.76 | **0.505** | 8.15 ± 9.65 |  | 6.16 ± 7.23 | **0.003** | 4.06 ± 5.32 |
| **FD-MSCM-SH vs. BMSKM-SH** | 7.82 ± 8.76 | **0.527** | 7.27 ± 8.25 |  | 6.16 ± 7.23 | **0.591** | 5.54 ± 6.48 |
| **FD-MSCM-SH vs. BMSKM-EL** | 7.82 ± 8.76 | **0.128** | 6.93 ± 8.19 |  | 6.16 ± 7.23 | **0.021** | 4.56 ± 5.86 |
| **FD-MSCM-EL vs. MSISA-P** | 7.79 ± 9.05 | **0.539** | 5.56 ± 5.86 |  | 6.38 ± 7.19 | **0.987** | 5.61 ± 7.17 |
| **FD-MSCM-EL vs. MSISA-R** | 7.79 ± 9.05 | **0.017** | 9.69 ± 9.37 |  | 6.38 ± 7.19 | **0.241** | 9.66 ± 9.95 |
| **FD-MSCM-EL vs. MSKM-SH** | 7.79 ± 9.05 | **0.742** | 7.87 ± 9.35 |  | 6.38 ± 7.19 | **0.093** | 4.38 ± 5.10 |
| **FD-MSCM-EL vs. MSKM-EL** | 7.79 ± 9.05 | **0.867** | 8.15 ± 9.65 |  | 6.38 ± 7.19 | **0.005** | 4.06 ± 5.32 |
| **FD-MSCM-EL vs. BMSKM-SH** | 7.79 ± 9.05 | **0.871** | 7.27 ± 8.25 |  | 6.38 ± 7.19 | **0.700** | 5.54 ± 6.48 |
| **FD-MSCM-EL vs. BMSKM-EL** | 7.79 ± 9.05 | **0.296** | 6.93 ± 8.19 |  | 6.38 ± 7.19 | **0.018** | 4.56 ± 5.86 |
|  |  |  |  |  |  |  |  |
|  |  |  |  |  |  |  |  |
| ***B. subtilis - L. monocytogenes pairing*** |  | ***B. subtilis*** |  |  | ***L. monocytogenes*** | | |
| **dist1 vs. dist2** | **dist1 mean (green)** | **Wilcoxon's  2-sided** | **dist2 mean (red)** |  | **dist1 mean (green)** | **Wilcoxon's  2-sided** | **dist2 mean (red)** |
| **FD-MSCM-SH vs. FD-SSCM** | 7.70 ± 8.79 | **0.004** | 9.78 ± 9.76 |  | 5.84 ± 7.26 | **0.012** | 6.90 ± 7.75 |
| **FD-MSCM-SH vs. QUBIC** | 7.70 ± 8.79 | **8.94E-30** | 2.52 ± 4.78 |  | 5.84 ± 7.26 | **1.73E-05** | 9.95 ± 10.82 |
| **FD-MSCM-SH vs. FD-COAL** | 7.70 ± 8.79 | **0.165** | 7.57 ± 9.16 |  | 5.84 ± 7.26 | **0.146** | 5.93 ± 8.27 |
| **FD-MSCM-EL vs. FD-SSCM** | 7.68 ± 9.27 | **1.72E-04** | 9.78 ± 9.76 |  | 5.69 ± 6.92 | **0.007** | 6.90 ± 7.75 |
| **FD-MSCM-EL vs. QUBIC** | 7.68 ± 9.27 | **3.99E-27** | 2.52 ± 4.78 |  | 5.69 ± 6.92 | **3.27E-06** | 9.95 ± 10.82 |
| **FD-MSCM-EL vs. FD-COAL** | 7.68 ± 9.27 | **0.636** | 7.57 ± 9.16 |  | 5.69 ± 6.92 | **0.138** | 5.93 ± 8.27 |
| **FD-MSCM-SH vs. MSISA-P** | 7.70 ± 8.79 | **0.185** | 9.05 ± 8.89 |  | 5.84 ± 7.26 | **0.948** | 3.70 ± 1.79 |
| **FD-MSCM-SH vs. MSISA-R** | 7.70 ± 8.79 | **0.025** | 9.61 ± 9.29 |  | 5.84 ± 7.26 | **0.257** | 6.20 ± 6.65 |
| **FD-MSCM-SH vs. MSKM-SH** | 7.70 ± 8.79 | **0.088** | 9.76 ± 10.54 |  | 5.84 ± 7.26 | **0.165** | 7.88 ± 9.56 |
| **FD-MSCM-SH vs. MSKM-EL** | 7.70 ± 8.79 | **0.364** | 7.68 ± 9.47 |  | 5.84 ± 7.26 | **0.152** | 4.91 ± 6.44 |
| **FD-MSCM-SH vs. BMSKM-SH** | 7.70 ± 8.79 | **0.392** | 9.23 ± 10.39 |  | 5.84 ± 7.26 | **0.874** | 7.10 ± 9.45 |
| **FD-MSCM-SH vs. BMSKM-EL** | 7.70 ± 8.79 | **0.025** | 6.79 ± 8.75 |  | 5.84 ± 7.26 | **0.179** | 4.86 ± 6.39 |
| **FD-MSCM-EL vs. MSISA-P** | 7.68 ± 9.27 | **0.137** | 9.05 ± 8.89 |  | 5.69 ± 6.92 | **0.941** | 3.70 ± 1.79 |
| **FD-MSCM-EL vs. MSISA-R** | 7.68 ± 9.27 | **0.005** | 9.61 ± 9.29 |  | 5.69 ± 6.92 | **0.218** | 6.20 ± 6.65 |
| **FD-MSCM-EL vs. MSKM-SH** | 7.68 ± 9.27 | **0.018** | 9.76 ± 10.54 |  | 5.69 ± 6.92 | **0.152** | 7.88 ± 9.56 |
| **FD-MSCM-EL vs. MSKM-EL** | 7.68 ± 9.27 | **0.878** | 7.68 ± 9.47 |  | 5.69 ± 6.92 | **0.138** | 4.91 ± 6.44 |
| **FD-MSCM-EL vs. BMSKM-SH** | 7.68 ± 9.27 | **0.127** | 9.23 ± 10.39 |  | 5.69 ± 6.92 | **0.854** | 7.10 ± 9.45 |
| **FD-MSCM-EL vs. BMSKM-EL** | 7.68 ± 9.27 | **0.146** | 6.79 ± 8.75 |  | 5.69 ± 6.92 | **0.147** | 4.86 ± 6.39 |
|  |  |  |  |  |  |  |  |
|  |  |  |  |  |  |  |  |
| ***B. anthracis - L. monocytogenes pairing*** |  | ***B. anthracis*** |  |  | ***L. monocytogenes*** | | |
| **dist1 vs. dist2** | **dist1 mean (green)** | **Wilcoxon's  2-sided** | **dist2 mean (red)** |  | **dist1 mean (green)** | **Wilcoxon's  2-sided** | **dist2 mean (red)** |
| **FD-MSCM-SH vs. FD-SSCM** | 6.80 ± 8.19 | **0.944** | 5.47 ± 5.39 |  | 5.35 ± 6.97 | **6.99E-04** | 6.90 ± 7.75 |
| **FD-MSCM-SH vs. QUBIC** | 6.80 ± 8.19 | **0.679** | 6.73 ± 7.52 |  | 5.35 ± 6.97 | **6.82E-08** | 9.95 ± 10.82 |
| **FD-MSCM-SH vs. FD-COAL** | 6.80 ± 8.19 | **0.226** | 6.50 ± 8.74 |  | 5.35 ± 6.97 | **0.281** | 5.93 ± 8.27 |
| **FD-MSCM-EL vs. FD-SSCM** | 6.70 ± 7.99 | **0.869** | 5.47 ± 5.39 |  | 5.03 ± 6.91 | **8.08E-06** | 6.90 ± 7.75 |
| **FD-MSCM-EL vs. QUBIC** | 6.70 ± 7.99 | **0.838** | 6.73 ± 7.52 |  | 5.03 ± 6.91 | **2.94E-11** | 9.95 ± 10.82 |
| **FD-MSCM-EL vs. FD-COAL** | 6.70 ± 7.99 | **0.095** | 6.50 ± 8.74 |  | 5.03 ± 6.91 | **0.567** | 5.93 ± 8.27 |
| **FD-MSCM-SH vs. MSKM-SH** | 6.80 ± 8.19 | **0.930** | 5.67 ± 7.00 |  | 5.35 ± 6.97 | **0.418** | 6.83 ± 8.86 |
| **FD-MSCM-SH vs. MSKM-EL** | 6.80 ± 8.19 | **0.049** | 3.86 ± 4.13 |  | 5.35 ± 6.97 | **0.334** | 4.94 ± 6.73 |
| **FD-MSCM-EL vs. MSKM-SH** | 6.70 ± 7.99 | **0.778** | 5.67 ± 7.00 |  | 5.03 ± 6.91 | **0.186** | 6.83 ± 8.86 |
| **FD-MSCM-EL vs. MSKM-EL** | 6.70 ± 7.99 | **0.011** | 3.86 ± 4.13 |  | 5.03 ± 6.91 | **0.687** | 4.94 ± 6.73 |

#### Motif E-values

Table S7: Comparison of bicluster motif E-values(-log10) from the full data methods considered by this study for all pairings of B. subtilis, B. anthracis and L. monocytogenes. A comparison of the motif E-values (-log10) from MSCM (full data) with all other relevant methods for all 3 pairings of the three organisms examined. In the comparisons, we compare both MSCM steps to the other methods. Displayed are the means for each method and/or step compared, as well as the Wilcoxon’s non-parametric rank test (2-sided) comparing their distributions. We direct the reader to 4 for instructions on how to interpret the table. As the table indicates, in 69 of the 92 of the comparisons (75%) MSCM does as well or better than its competitors.

| ***B. subtilis - B. anthracis pairing*** | ***B. subtilis*** | | |  | ***B. anthracis*** | | |
| --- | --- | --- | --- | --- | --- | --- | --- |
| **dist1 vs. dist2** | **dist1 mean (green)** | **Wilcoxon's  2-sided** | **dist2 mean (red)** |  | **dist1 mean (green)** | **Wilcoxon's  2-sided** | **dist2 mean (red)** |
| **FD-MSCM-SH vs. FD-SSCM** | 2.17 ± 10.07 | **2.91E-10** | 7.03 ± 18.81 |  | 2.46 ± 8.08 | **0.043** | 4.98 ± 10.63 |
| **FD-MSCM-SH vs. QUBIC** | 2.17 ± 10.07 | **0.033** | 1.41 ± 3.94 |  | 2.46 ± 8.08 | **1.43E-07** | 13.72 ± 14.49 |
| **FD-MSCM-SH vs. FD-COAL** | 2.17 ± 10.07 | **0.172** | 2.72 ± 7.30 |  | 2.46 ± 8.08 | **0.618** | 3.85 ± 8.83 |
| **FD-MSCM-EL vs. FD-SSCM** | 3.34 ± 8.22 | **4.28E-04** | 7.03 ± 18.81 |  | 3.74 ± 10.70 | **0.427** | 4.98 ± 10.63 |
| **FD-MSCM-EL vs. QUBIC** | 3.34 ± 8.22 | **0.802** | 1.41 ± 3.94 |  | 3.74 ± 10.70 | **5.09E-07** | 13.72 ± 14.49 |
| **FD-MSCM-EL vs. FD-COAL** | 3.34 ± 8.22 | **0.464** | 2.72 ± 7.30 |  | 3.74 ± 10.70 | **0.685** | 3.85 ± 8.83 |
| **FD-MSCM-SH vs. MSISA-P** | 2.17 ± 10.07 | **0.002** | -1.12 ± 2.03 |  | 2.46 ± 8.08 | **0.226** | 0.46 ± 3.43 |
| **FD-MSCM-SH vs. MSISA-R** | 2.17 ± 10.07 | **1.12E-08** | 9.40 ± 9.19 |  | 2.46 ± 8.08 | **4.99E-06** | 2.34 ± 11.56 |
| **FD-MSCM-SH vs. MSKM-SH** | 2.17 ± 10.07 | **1.75E-07** | -1.18 ± 2.62 |  | 2.46 ± 8.08 | **0.001** | -0.22 ± 2.96 |
| **FD-MSCM-SH vs. MSKM-EL** | 2.17 ± 10.07 | **0.045** | 0.19 ± 4.26 |  | 2.46 ± 8.08 | **0.037** | 2.74 ± 5.66 |
| **FD-MSCM-SH vs. BMSKM-SH** | 2.17 ± 10.07 | **1.02E-06** | -1.09 ± 2.68 |  | 2.46 ± 8.08 | **1.87E-04** | -0.39 ± 2.87 |
| **FD-MSCM-SH vs. BMSKM-EL** | 2.17 ± 10.07 | **0.378** | 0.44 ± 4.06 |  | 2.46 ± 8.08 | **0.002** | 3.07 ± 5.44 |
| **FD-MSCM-EL vs. MSISA-P** | 3.34 ± 8.22 | **5.26E-05** | -1.12 ± 2.03 |  | 3.74 ± 10.70 | **0.059** | 0.46 ± 3.43 |
| **FD-MSCM-EL vs. MSISA-R** | 3.34 ± 8.22 | **7.22E-06** | 9.40 ± 9.19 |  | 3.74 ± 10.70 | **5.97E-06** | 2.34 ± 11.56 |
| **FD-MSCM-EL vs. MSKM-SH** | 3.34 ± 8.22 | **2.91E-11** | -1.18 ± 2.62 |  | 3.74 ± 10.70 | **2.12E-05** | -0.22 ± 2.96 |
| **FD-MSCM-EL vs. MSKM-EL** | 3.34 ± 8.22 | **1.88E-04** | 0.19 ± 4.26 |  | 3.74 ± 10.70 | **0.495** | 2.74 ± 5.66 |
| **FD-MSCM-EL vs. BMSKM-SH** | 3.34 ± 8.22 | **2.18E-10** | -1.09 ± 2.68 |  | 3.74 ± 10.70 | **3.64E-06** | -0.39 ± 2.87 |
| **FD-MSCM-EL vs. BMSKM-EL** | 3.34 ± 8.22 | **0.004** | 0.44 ± 4.06 |  | 3.74 ± 10.70 | **0.101** | 3.07 ± 5.44 |
|  |  |  |  |  |  |  |  |
|  |  |  |  |  |  |  |  |
| ***B. subtilis - L. monocytogenes pairing*** | ***B. subtilis*** | | |  | ***L. monocytogenes*** | | |
| **dist1 vs. dist2** | **dist1 mean (green)** | **Wilcoxon's  2-sided** | **dist2 mean (red)** |  | **dist1 mean (green)** | **Wilcoxon's  2-sided** | **dist2 mean (red)** |
| **FD-MSCM-SH vs. FD-SSCM** | 0.43 ± 5.96 | **7.22E-18** | 7.03 ± 18.81 |  | 1.65 ± 5.96 | **3.46E-07** | 0.46 ± 4.73 |
| **FD-MSCM-SH vs. QUBIC** | 0.43 ± 5.96 | **6.64E-06** | 1.41 ± 3.94 |  | 1.65 ± 5.96 | **0.352** | 9.57 ± 13.54 |
| **FD-MSCM-SH vs. FD-COAL** | 0.43 ± 5.96 | **0.001** | 2.72 ± 7.30 |  | 1.65 ± 5.96 | **0.325** | 3.66 ± 7.60 |
| **FD-MSCM-EL vs. FD-SSCM** | 2.12 ± 7.86 | **3.86E-08** | 7.03 ± 18.81 |  | 3.32 ± 9.98 | **1.17E-10** | 0.46 ± 4.73 |
| **FD-MSCM-EL vs. QUBIC** | 2.12 ± 7.86 | **0.127** | 1.41 ± 3.94 |  | 3.32 ± 9.98 | **0.573** | 9.57 ± 13.54 |
| **FD-MSCM-EL vs. FD-COAL** | 2.12 ± 7.86 | **0.370** | 2.72 ± 7.30 |  | 3.32 ± 9.98 | **0.917** | 3.66 ± 7.60 |
| **FD-MSCM-SH vs. MSISA-P** | 0.43 ± 5.96 | **1.84E-07** | -2.63 ± 1.00 |  | 1.65 ± 5.96 | **1.29E-06** | -1.56 ± 1.40 |
| **FD-MSCM-SH vs. MSISA-R** | 0.43 ± 5.96 | **5.52E-13** | 10.37 ± 8.84 |  | 1.65 ± 5.96 | **1.12E-07** | 9.06 ± 7.74 |
| **FD-MSCM-SH vs. MSKM-SH** | 0.43 ± 5.96 | **5.11E-07** | -1.91 ± 1.56 |  | 1.65 ± 5.96 | **4.50E-07** | -0.83 ± 1.64 |
| **FD-MSCM-SH vs. MSKM-EL** | 0.43 ± 5.96 | **0.985** | 0.52 ± 5.33 |  | 1.65 ± 5.96 | **0.169** | 0.36 ± 2.68 |
| **FD-MSCM-SH vs. BMSKM-SH** | 0.43 ± 5.96 | **5.07E-08** | -1.97 ± 1.58 |  | 1.65 ± 5.96 | **5.68E-08** | -0.89 ± 1.69 |
| **FD-MSCM-SH vs. BMSKM-EL** | 0.43 ± 5.96 | **0.992** | 0.02 ± 3.81 |  | 1.65 ± 5.96 | **0.177** | 0.43 ± 3.17 |
| **FD-MSCM-EL vs. MSISA-P** | 2.12 ± 7.86 | **7.84E-10** | -2.63 ± 1.00 |  | 3.32 ± 9.98 | **1.56E-08** | -1.56 ± 1.40 |
| **FD-MSCM-EL vs. MSISA-R** | 2.12 ± 7.86 | **6.40E-10** | 10.37 ± 8.84 |  | 3.32 ± 9.98 | **2.25E-06** | 9.06 ± 7.74 |
| **FD-MSCM-EL vs. MSKM-SH** | 2.12 ± 7.86 | **2.41E-13** | -1.91 ± 1.56 |  | 3.32 ± 9.98 | **1.69E-11** | -0.83 ± 1.64 |
| **FD-MSCM-EL vs. MSKM-EL** | 2.12 ± 7.86 | **0.013** | 0.52 ± 5.33 |  | 3.32 ± 9.98 | **0.002** | 0.36 ± 2.68 |
| **FD-MSCM-EL vs. BMSKM-SH** | 2.12 ± 7.86 | **2.88E-14** | -1.97 ± 1.58 |  | 3.32 ± 9.98 | **2.44E-12** | -0.89 ± 1.69 |
| **FD-MSCM-EL vs. BMSKM-EL** | 2.12 ± 7.86 | **0.007** | 0.02 ± 3.81 |  | 3.32 ± 9.98 | **0.002** | 0.43 ± 3.17 |
|  |  |  |  |  |  |  |  |
|  |  |  |  |  |  |  |  |
|  |  |  |  |  |  |  |  |
| ***B. anthracis - L. monocytogenes pairing*** | ***B. anthracis*** | | |  | ***L. monocytogenes*** | | |
| **dist1 vs. dist2** | **dist1 mean (green)** | **Wilcoxon's  2-sided** | **dist2 mean (red)** |  | **dist1 mean (green)** | **Wilcoxon's  2-sided** | **dist2 mean (red)** |
| **FD-MSCM-SH vs. FD-SSCM** | 0.02 ± 3.66 | **3.12E-08** | 5.21 ± 10.80 |  | 1.69 ± 5.21 | **1.86E-06** | 0.46 ± 4.73 |
| **FD-MSCM-SH vs. QUBIC** | 0.02 ± 3.66 | **5.33E-10** | 13.72 ± 14.49 |  | 1.69 ± 5.21 | **0.326** | 9.57 ± 13.54 |
| **FD-MSCM-SH vs. FD-COAL** | 0.02 ± 3.66 | **0.004** | 3.85 ± 8.83 |  | 1.69 ± 5.21 | **0.337** | 3.66 ± 7.60 |
| **FD-MSCM-EL vs. FD-SSCM** | 1.68 ± 6.38 | **0.001** | 5.21 ± 10.80 |  | 3.17 ± 8.21 | **3.92E-10** | 0.46 ± 4.73 |
| **FD-MSCM-EL vs. QUBIC** | 1.68 ± 6.38 | **4.06E-08** | 13.72 ± 14.49 |  | 3.17 ± 8.21 | **0.686** | 9.57 ± 13.54 |
| **FD-MSCM-EL vs. FD-COAL** | 1.68 ± 6.38 | **0.213** | 3.85 ± 8.83 |  | 3.17 ± 8.21 | **0.999** | 3.66 ± 7.60 |
| **FD-MSCM-SH vs. MSKM-SH** | 0.02 ± 3.66 | **3.71E-05** | -1.58 ± 1.60 |  | 1.69 ± 5.21 | **5.77E-06** | -0.67 ± 1.69 |
| **FD-MSCM-SH vs. MSKM-EL** | 0.02 ± 3.66 | **1.47E-04** | 2.52 ± 6.60 |  | 1.69 ± 5.21 | **0.118** | 0.44 ± 3.00 |
| **FD-MSCM-EL vs. MSKM-SH** | 1.68 ± 6.38 | **2.02E-09** | -1.58 ± 1.60 |  | 3.17 ± 8.21 | **3.65E-09** | -0.67 ± 1.69 |
| **FD-MSCM-EL vs. MSKM-EL** | 1.68 ± 6.38 | **0.099** | 2.52 ± 6.60 |  | 3.17 ± 8.21 | **0.004** | 0.44 ± 3.00 |

#### Sequence p-values

Table S8: Comparison of bicluster sequence p-values (-log10) from the full data methods considered by this study for all pairings of B. subtilis, B. anthracis and L. monocytogenes. A comparison of the sequence p-values (-log10) from MSCM (full data) with all other relevant methods for all 3 pairings of the three organisms examined. In the comparisons, we compare both MSCM steps to the other methods. Displayed are the means for each method and/or step compared, as well as the Wilcoxon’s non-parametric rank test (2-sided) comparing their distributions. We direct the reader to 4 for instructions on how to interpret the table. As the table indicates, in 72 of the 92 of the comparisons (78.3%) MSCM does as well or better than its competitors.

| ***B. subtilis - B. anthracis pairing*** | ***B. subtilis*** | | |  | ***B. anthracis*** | | |
| --- | --- | --- | --- | --- | --- | --- | --- |
| **dist1 vs. dist2** | **dist1 mean (green)** | **Wilcoxon's  2-sided** | **dist2 mean (red)** |  | **dist1 mean (green)** | **Wilcoxon's 2-sided** | **dist2 mean (red)** |
| **FD-MSCM-SH vs. FD-SSCM** | 3.86 ± 1.39 | **2.84E-22** | 6.73 ± 3.35 |  | 3.49 ± 1.31 | **0.303** | 3.90 ± 2.62 |
| **FD-MSCM-SH vs. QUBIC** | 3.86 ± 1.39 | **4.41E-33** | 2.06 ± 0.50 |  | 3.49 ± 1.31 | **1.78E-32** | 1.77 ± 0.26 |
| **FD-MSCM-SH vs. FD-COAL** | 3.86 ± 1.39 | **7.40E-25** | 2.47 ± 1.12 |  | 3.49 ± 1.31 | **1.71E-18** | 2.32 ± 1.57 |
| **FD-MSCM-EL vs. FD-SSCM** | 3.47 ± 1.31 | **3.39E-29** | 6.73 ± 3.35 |  | 3.24 ± 1.22 | **0.721** | 3.90 ± 2.62 |
| **FD-MSCM-EL vs. QUBIC** | 3.47 ± 1.31 | **1.83E-25** | 2.06 ± 0.50 |  | 3.24 ± 1.22 | **5.27E-31** | 1.77 ± 0.26 |
| **FD-MSCM-EL vs. FD-COAL** | 3.47 ± 1.31 | **1.64E-16** | 2.47 ± 1.12 |  | 3.24 ± 1.22 | **1.08E-15** | 2.32 ± 1.57 |
| **FD-MSCM-SH vs. MSISA-P** | 3.86 ± 1.39 | **0.164** | 3.65 ± 1.74 |  | 3.49 ± 1.31 | **0.345** | 3.34 ± 1.33 |
| **FD-MSCM-SH vs. MSISA-R** | 3.86 ± 1.39 | **5.63E-15** | 2.02 ± 0.52 |  | 3.49 ± 1.31 | **5.99E-07** | 1.79 ± 0.27 |
| **FD-MSCM-SH vs. MSKM-SH** | 3.86 ± 1.39 | **0.879** | 3.97 ± 1.81 |  | 3.49 ± 1.31 | **0.985** | 3.59 ± 1.53 |
| **FD-MSCM-SH vs. MSKM-EL** | 3.86 ± 1.39 | **8.16E-06** | 3.24 ± 1.58 |  | 3.49 ± 1.31 | **1.04E-09** | 2.66 ± 1.03 |
| **FD-MSCM-SH vs. BMSKM-SH** | 3.86 ± 1.39 | **0.806** | 4.05 ± 1.86 |  | 3.49 ± 1.31 | **0.577** | 3.42 ± 1.33 |
| **FD-MSCM-SH vs. BMSKM-EL** | 3.86 ± 1.39 | **1.02E-07** | 3.06 ± 1.30 |  | 3.49 ± 1.31 | **2.97E-11** | 2.57 ± 0.88 |
| **FD-MSCM-EL vs. MSISA-P** | 3.47 ± 1.31 | **0.915** | 3.65 ± 1.74 |  | 3.24 ± 1.22 | **0.890** | 3.34 ± 1.33 |
| **FD-MSCM-EL vs. MSISA-R** | 3.47 ± 1.31 | **1.63E-12** | 2.02 ± 0.52 |  | 3.24 ± 1.22 | **1.06E-06** | 1.79 ± 0.27 |
| **FD-MSCM-EL vs. MSKM-SH** | 3.47 ± 1.31 | **0.029** | 3.97 ± 1.81 |  | 3.24 ± 1.22 | **0.077** | 3.59 ± 1.53 |
| **FD-MSCM-EL vs. MSKM-EL** | 3.47 ± 1.31 | **0.046** | 3.24 ± 1.58 |  | 3.24 ± 1.22 | **2.57E-06** | 2.66 ± 1.03 |
| **FD-MSCM-EL vs. BMSKM-SH** | 3.47 ± 1.31 | **0.008** | 4.05 ± 1.86 |  | 3.24 ± 1.22 | **0.238** | 3.42 ± 1.33 |
| **FD-MSCM-EL vs. BMSKM-EL** | 3.47 ± 1.31 | **0.004** | 3.06 ± 1.30 |  | 3.24 ± 1.22 | **9.75E-08** | 2.57 ± 0.88 |
|  |  |  |  |  |  |  |  |
|  |  |  |  |  |  |  |  |
| ***B. subtilis - L. monocytogenes pairing*** | ***B. subtilis*** | | |  | ***L. monocytogenes*** | | |
| **dist1 vs. dist2** | **dist1 mean (green)** | **Wilcoxon's  2-sided** | **dist2 mean (red)** |  | **dist1 mean (green)** | **Wilcoxon's 2-sided** | **dist2 mean (red)** |
| **FD-MSCM-SH vs. FD-SSCM** | 4.31 ± 1.94 | 6.74E-15 | 6.73 ± 3.35 |  | 4.82 ± 1.63 | **0.245** | 5.24 ± 2.35 |
| **FD-MSCM-SH vs. QUBIC** | 4.31 ± 1.94 | **4.06E-33** | 2.06 ± 0.50 |  | 4.82 ± 1.63 | **2.19E-30** | 2.36 ± 0.37 |
| **FD-MSCM-SH vs. FD-COAL** | 4.31 ± 1.94 | **9.99E-27** | 2.47 ± 1.12 |  | 4.82 ± 1.63 | **1.35E-08** | 3.51 ± 1.51 |
| **FD-MSCM-EL vs. FD-SSCM** | 3.85 ± 1.82 | **1.47E-22** | 6.73 ± 3.35 |  | 4.55 ± 1.60 | **0.015** | 5.24 ± 2.35 |
| **FD-MSCM-EL vs. QUBIC** | 3.85 ± 1.82 | **8.34E-29** | 2.06 ± 0.50 |  | 4.55 ± 1.60 | **1.38E-26** | 2.36 ± 0.37 |
| **FD-MSCM-EL vs. FD-COAL** | 3.85 ± 1.82 | **3.61E-20** | 2.47 ± 1.12 |  | 4.55 ± 1.60 | **1.80E-06** | 3.51 ± 1.51 |
| **FD-MSCM-SH vs. MSISA-P** | 4.31 ± 1.94 | **0.054** | 5.06 ± 2.38 |  | 4.82 ± 1.63 | **0.007** | 5.77 ± 1.91 |
| **FD-MSCM-SH vs. MSISA-R** | 4.31 ± 1.94 | **4.83E-15** | 1.99 ± 0.50 |  | 4.82 ± 1.63 | **9.28E-16** | 2.42 ± 0.56 |
| **FD-MSCM-SH vs. MSKM-SH** | 4.31 ± 1.94 | **0.007** | 4.79 ± 1.75 |  | 4.82 ± 1.63 | **4.44E-04** | 5.49 ± 1.73 |
| **FD-MSCM-SH vs. MSKM-EL** | 4.31 ± 1.94 | **4.42E-06** | 3.45 ± 1.88 |  | 4.82 ± 1.63 | **0.004** | 4.35 ± 1.67 |
| **FD-MSCM-SH vs. BMSKM-SH** | 4.31 ± 1.94 | **0.161** | 4.61 ± 2.13 |  | 4.82 ± 1.63 | **0.320** | 5.02 ± 1.71 |
| **FD-MSCM-SH vs. BMSKM-EL** | 4.31 ± 1.94 | **3.12E-08** | 3.19 ± 1.39 |  | 4.82 ± 1.63 | **0.006** | 4.43 ± 1.62 |
| **FD-MSCM-EL vs. MSISA-P** | 3.85 ± 1.82 | **0.002** | 5.06 ± 2.38 |  | 4.55 ± 1.60 | **0.001** | 5.77 ± 1.91 |
| **FD-MSCM-EL vs. MSISA-R** | 3.85 ± 1.82 | **4.04E-13** | 1.99 ± 0.50 |  | 4.55 ± 1.60 | **1.78E-13** | 2.42 ± 0.56 |
| **FD-MSCM-EL vs. MSKM-SH** | 3.85 ± 1.82 | **3.82E-07** | 4.79 ± 1.75 |  | 4.55 ± 1.60 | **5.17E-06** | 5.49 ± 1.73 |
| **FD-MSCM-EL vs. MSKM-EL** | 3.85 ± 1.82 | **0.007** | 3.45 ± 1.88 |  | 4.55 ± 1.60 | **0.145** | 4.35 ± 1.67 |
| **FD-MSCM-EL vs. BMSKM-SH** | 3.85 ± 1.82 | **2.38E-04** | 4.61 ± 2.13 |  | 4.55 ± 1.60 | **0.026** | 5.02 ± 1.71 |
| **FD-MSCM-EL vs. BMSKM-EL** | 3.85 ± 1.82 | **4.73E-04** | 3.19 ± 1.39 |  | 4.55 ± 1.60 | **0.244** | 4.43 ± 1.62 |
|  |  |  |  |  |  |  |  |
|  |  |  |  |  |  |  |  |
| ***B. anthracis - L. monocytogenes pairing*** | ***B. anthracis*** | | |  | ***L. monocytogenes*** | | |
| **dist1 vs. dist2** | **dist1 mean (green)** | **Wilcoxon's  2-sided** | **dist2 mean (red)** |  | **dist1 mean (green)** | **Wilcoxon's 2-sided** | **dist2 mean (red)** |
| **FD-MSCM-SH vs. FD-SSCM** | 3.80 ± 1.49 | **0.007** | 3.83 ± 2.57 |  | 4.98 ± 1.73 | **0.673** | 5.24 ± 2.35 |
| **FD-MSCM-SH vs. QUBIC** | 3.80 ± 1.49 | **1.97E-33** | 1.77 ± 0.26 |  | 4.98 ± 1.73 | **1.03E-33** | 2.36 ± 0.37 |
| **FD-MSCM-SH vs. FD-COAL** | 3.80 ± 1.49 | **1.47E-20** | 2.32 ± 1.57 |  | 4.98 ± 1.73 | **6.20E-10** | 3.51 ± 1.51 |
| **FD-MSCM-EL vs. FD-SSCM** | 3.38 ± 1.31 | **0.662** | 3.83 ± 2.57 |  | 4.66 ± 1.73 | **0.044** | 5.24 ± 2.35 |
| **FD-MSCM-EL vs. QUBIC** | 3.38 ± 1.31 | **2.49E-29** | 1.77 ± 0.26 |  | 4.66 ± 1.73 | **1.04E-30** | 2.36 ± 0.37 |
| **FD-MSCM-EL vs. FD-COAL** | 3.38 ± 1.31 | **6.13E-16** | 2.32 ± 1.57 |  | 4.66 ± 1.73 | **3.57E-07** | 3.51 ± 1.51 |
| **FD-MSCM-SH vs. MSKM-SH** | 3.80 ± 1.49 | **0.303** | 3.94 ± 1.46 |  | 4.98 ± 1.73 | **0.001** | 5.61 ± 1.74 |
| **FD-MSCM-SH vs. MSKM-EL** | 3.80 ± 1.49 | **8.75E-14** | 2.61 ± 1.04 |  | 4.98 ± 1.73 | **0.001** | 4.37 ± 1.51 |
| **FD-MSCM-EL vs. MSKM-SH** | 3.38 ± 1.31 | **4.908E-04** | 3.94 ± 1.46 |  | 4.66 ± 1.73 | **3.80E-07** | 5.61 ± 1.74 |
| **FD-MSCM-EL vs. MSKM-EL** | 3.38 ± 1.31 | **3.44E-08** | 2.61 ± 1.04 |  | 4.66 ± 1.73 | **0.191** | 4.37 ± 1.51 |

### Comparisons with EO-MSCM

#### Residuals

Table S9: Comparison of bicluster residuals from the expression only methods considered by this study for all pairings of B. subtilis, B. anthracis and L. monocytogenes. A comparison of the residuals of the results from MSCM (expression only) with all other relevant methods for all 3 pairings of the three organisms examined. In the comparisons, we compare both MSCM steps to the other methods. Displayed are the means for each method and/or step compared, as well as the Wilcoxon’s non-parametric rank test (2-sided) comparing their distributions. We direct the reader to 4 for instructions on how to interpret the table. In this case, these results illustrate that in 61 of the 116 comparisons (52.6%) MSCM step did as well or better than its competitors.

| ***B. subtilis - B. anthracis pairing*** |  | ***B. subtilis*** |  |  |  | ***B. anthracis*** |  |
| --- | --- | --- | --- | --- | --- | --- | --- |
| **dist1 vs. dist2** | **dist1 mean (green)** | **Wilcoxon's  2-sided** | **dist2 mean (red)** |  | **dist1 mean (green)** | **Wilcoxon's  2-sided** | **dist2 mean (red)** |
| **EO-MSCM-SH vs. EO-SSCM** | 0.52 ± 0.09 | **6.21E-20** | 0.44 ± 0.20 |  | 0.50 ± 0.20 | **1.89E-38** | 0.23 ± 0.06 |
| **EO-MSCM-SH vs. FD-SSCM** | 0.52 ± 0.09 | **2.38E-05** | 0.49 ± 0.13 |  | 0.50 ± 0.20 | **1.79E-23** | 0.31 ± 0.12 |
| **EO-MSCM-SH vs. QUBIC** | 0.52 ± 0.09 | **9.34E-36** | 0.87 ± 0.21 |  | 0.50 ± 0.20 | **3.12E-50** | 1.51 ± 0.29 |
| **EO-MSCM-SH vs. EO-COAL** | 0.52 ± 0.09 | **7.01E-40** | 0.78 ± 0.23 |  | 0.50 ± 0.20 | **1.98E-04** | 0.58 ± 0.17 |
| **EO-MSCM-SH vs. FD-COAL** | 0.52 ± 0.09 | **3.60E-40** | 0.80 ± 0.25 |  | 0.50 ± 0.20 | **3.53E-04** | 0.58 ± 0.17 |
| **EO-MSCM-EL vs. EO-SSCM** | 0.52 ± 0.10 | **2.72E-18** | 0.44 ± 0.20 |  | 0.49 ± 0.20 | **3.63E-35** | 0.23 ± 0.06 |
| **EO-MSCM-EL vs. FD-SSCM** | 0.52 ± 0.10 | **0.003** | 0.49 ± 0.13 |  | 0.49 ± 0.20 | **2.76E-20** | 0.31 ± 0.12 |
| **EO-MSCM-EL vs. QUBIC** | 0.52 ± 0.10 | **1.35E-35** | 0.87 ± 0.21 |  | 0.49 ± 0.20 | **2.88E-50** | 1.51 ± 0.29 |
| **EO-MSCM-EL vs. EO-COAL** | 0.52 ± 0.10 | **2.61E-42** | 0.78 ± 0.23 |  | 0.49 ± 0.20 | **3.81E-05** | 0.58 ± 0.17 |
| **EO-MSCM-EL vs. FD-COAL** | 0.52 ± 0.10 | **1.63E-42** | 0.80 ± 0.25 |  | 0.49 ± 0.20 | **6.99E-05** | 0.58 ± 0.17 |
| **EO-MSCM-SH vs. MSISA-P** | 0.52 ± 0.09 | **9.13E-17** | 0.98 ± 0.39 |  | 0.50 ± 0.20 | **1.71E-22** | 1.97 ± 0.94 |
| **EO-MSCM-SH vs. MSISA-R** | 0.52 ± 0.09 | **2.34E-19** | 1.11 ± 0.41 |  | 0.50 ± 0.20 | **5.65E-22** | 1.58 ± 0.38 |
| **EO-MSCM-SH vs. MSKM-SH** | 0.52 ± 0.09 | **4.07E-30** | 0.41 ± 0.07 |  | 0.50 ± 0.20 | **0.107** | 0.53 ± 0.12 |
| **EO-MSCM-SH vs. MSKM-EL** | 0.52 ± 0.09 | **2.86E-27** | 0.42 ± 0.06 |  | 0.50 ± 0.20 | **0.455** | 0.48 ± 0.11 |
| **EO-MSCM-SH vs. BMSKM-SH** | 0.52 ± 0.09 | **4.07E-14** | 0.45 ± 0.07 |  | 0.50 ± 0.20 | **8.32E-08** | 0.38 ± 0.07 |
| **EO-MSCM-SH vs. BMSKM-EL** | 0.52 ± 0.09 | **1.08E-14** | 0.45 ± 0.06 |  | 0.50 ± 0.20 | **1.06E-06** | 0.39 ± 0.07 |
| **EO-MSCM-EL vs. MSISA-P** | 0.52 ± 0.10 | **6.41E-17** | 0.98 ± 0.39 |  | 0.49 ± 0.20 | **1.51E-22** | 1.97 ± 0.94 |
| **EO-MSCM-EL vs. MSISA-R** | 0.52 ± 0.10 | **2.20E-19** | 1.11 ± 0.41 |  | 0.49 ± 0.20 | **5.31E-22** | 1.58 ± 0.38 |
| **EO-MSCM-EL vs. MSKM-SH** | 0.52 ± 0.10 | **5.39E-27** | 0.41 ± 0.07 |  | 0.49 ± 0.20 | **0.047** | 0.53 ± 0.12 |
| **EO-MSCM-EL vs. MSKM-EL** | 0.52 ± 0.10 | **5.92E-24** | 0.42 ± 0.06 |  | 0.49 ± 0.20 | **0.703** | 0.48 ± 0.11 |
| **EO-MSCM-EL vs. BMSKM-SH** | 0.52 ± 0.10 | **1.11E-09** | 0.45 ± 0.07 |  | 0.49 ± 0.20 | **1.04E-06** | 0.38 ± 0.07 |
| **EO-MSCM-EL vs. BMSKM-EL** | 0.52 ± 0.10 | **1.24E-09** | 0.45 ± 0.06 |  | 0.49 ± 0.20 | **8.70E-06** | 0.39 ± 0.07 |
|  |  |  |  |  |  |  |  |
|  |  |  |  |  |  |  |  |
| ***B. subtilis - L. monocytogenes pairing*** |  | ***B. subtilis*** |  |  | ***L. monocytogenes*** | | |
| **dist1 vs. dist2** | **dist1 mean (green)** | **Wilcoxon's  2-sided** | **dist2 mean (red)** |  | **dist1 mean (green)** | **Wilcoxon's  2-sided** | **dist2 mean (red)** |
| **EO-MSCM-SH vs. EO-SSCM** | 0.52 ± 0.08 | **6.03E-20** | 0.44 ± 0.20 |  | 0.49 ± 0.17 | **1.42E-17** | 0.29 ± 0.10 |
| **EO-MSCM-SH vs. FD-SSCM** | 0.52 ± 0.08 | **1.30E-04** | 0.49 ± 0.13 |  | 0.49 ± 0.17 | **5.74E-08** | 0.40 ± 0.18 |
| **EO-MSCM-SH vs. QUBIC** | 0.52 ± 0.08 | **6.59E-37** | 0.87 ± 0.21 |  | 0.49 ± 0.17 | **1.06E-23** | 1.81 ± 0.85 |
| **EO-MSCM-SH vs. EO-COAL** | 0.52 ± 0.08 | **1.33E-43** | 0.78 ± 0.23 |  | 0.49 ± 0.17 | **0.657** | 1.63 ± 3.07 |
| **EO-MSCM-SH vs. FD-COAL** | 0.52 ± 0.08 | **2.21E-43** | 0.80 ± 0.25 |  | 0.49 ± 0.17 | **0.617** | 1.70 ± 3.24 |
| **EO-MSCM-EL vs. EO-SSCM** | 0.50 ± 0.09 | **7.36E-17** | 0.44 ± 0.20 |  | 0.48 ± 0.17 | **1.85E-17** | 0.29 ± 0.10 |
| **EO-MSCM-EL vs. FD-SSCM** | 0.50 ± 0.09 | **0.055** | 0.49 ± 0.13 |  | 0.48 ± 0.17 | **1.75E-07** | 0.40 ± 0.18 |
| **EO-MSCM-EL vs. QUBIC** | 0.50 ± 0.09 | **1.10E-37** | 0.87 ± 0.21 |  | 0.48 ± 0.17 | **5.53E-24** | 1.81 ± 0.85 |
| **EO-MSCM-EL vs. EO-COAL** | 0.50 ± 0.09 | **6.71E-47** | 0.78 ± 0.23 |  | 0.48 ± 0.17 | **0.755** | 1.63 ± 3.07 |
| **EO-MSCM-EL vs. FD-COAL** | 0.50 ± 0.09 | **1.26E-46** | 0.80 ± 0.25 |  | 0.48 ± 0.17 | **0.734** | 1.70 ± 3.24 |
| **EO-MSCM-SH vs. MSISA-P** | 0.52 ± 0.08 | **8.65E-10** | 0.87 ± 0.34 |  | 0.49 ± 0.17 | **4.97E-19** | 1.59 ± 0.52 |
| **EO-MSCM-SH vs. MSISA-R** | 0.52 ± 0.08 | **1.64E-18** | 1.11 ± 0.42 |  | 0.49 ± 0.17 | **3.60E-21** | 1.31 ± 0.34 |
| **EO-MSCM-SH vs. MSKM-SH** | 0.52 ± 0.08 | **1.11E-34** | 0.40 ± 0.07 |  | 0.49 ± 0.17 | **0.358** | 0.50 ± 0.12 |
| **EO-MSCM-SH vs. MSKM-EL** | 0.52 ± 0.08 | **3.97E-28** | 0.42 ± 0.06 |  | 0.49 ± 0.17 | **0.683** | 0.48 ± 0.11 |
| **EO-MSCM-SH vs. BMSKM-SH** | 0.52 ± 0.08 | **1.14E-20** | 0.43 ± 0.07 |  | 0.49 ± 0.17 | **3.32E-04** | 0.42 ± 0.09 |
| **EO-MSCM-SH vs. BMSKM-EL** | 0.52 ± 0.08 | **1.16E-18** | 0.44 ± 0.06 |  | 0.49 ± 0.17 | **7.46E-04** | 0.42 ± 0.09 |
| **EO-MSCM-EL vs. MSISA-P** | 0.50 ± 0.09 | **4.44E-10** | 0.87 ± 0.34 |  | 0.48 ± 0.17 | **4.97E-19** | 1.59 ± 0.52 |
| **EO-MSCM-EL vs. MSISA-R** | 0.50 ± 0.09 | **1.38E-18** | 1.11 ± 0.42 |  | 0.48 ± 0.17 | **3.60E-21** | 1.31 ± 0.34 |
| **EO-MSCM-EL vs. MSKM-SH** | 0.50 ± 0.09 | **4.41E-29** | 0.40 ± 0.07 |  | 0.48 ± 0.17 | **0.190** | 0.50 ± 0.12 |
| **EO-MSCM-EL vs. MSKM-EL** | 0.50 ± 0.09 | **5.98E-22** | 0.42 ± 0.06 |  | 0.48 ± 0.17 | **0.972** | 0.48 ± 0.11 |
| **EO-MSCM-EL vs. BMSKM-SH** | 0.50 ± 0.09 | **2.09E-14** | 0.43 ± 0.07 |  | 0.48 ± 0.17 | **0.001** | 0.42 ± 0.09 |
| **EO-MSCM-EL vs. BMSKM-EL** | 0.50 ± 0.09 | **4.23E-12** | 0.44 ± 0.06 |  | 0.48 ± 0.17 | **0.002** | 0.42 ± 0.09 |
|  |  |  |  |  |  |  |  |
|  |  |  |  |  |  |  |  |
| ***B. anthracis - L. monocytogenes pairing*** | ***B. anthracis*** | | |  | ***L. monocytogenes*** | | |
| **dist1 vs. dist2** | **dist1 mean (green)** | **Wilcoxon's  2-sided** | **dist2 mean (red)** |  | **dist1 mean (green)** | **Wilcoxon's  2-sided** | **dist2 mean (red)** |
| **EO-MSCM-SH vs. EO-SSCM** | 0.52 ± 0.17 | **1.52E-48** | 0.23 ± 0.06 |  | 0.50 ± 0.18 | **2.48E-17** | 0.29 ± 0.10 |
| **EO-MSCM-SH vs. FD-SSCM** | 0.52 ± 0.17 | **9.86E-34** | 0.31 ± 0.12 |  | 0.50 ± 0.18 | **1.67E-08** | 0.40 ± 0.18 |
| **EO-MSCM-SH vs. QUBIC** | 0.52 ± 0.17 | **4.65E-49** | 1.51 ± 0.29 |  | 0.50 ± 0.18 | **1.06E-22** | 1.81 ± 0.85 |
| **EO-MSCM-SH vs. EO-COAL** | 0.52 ± 0.17 | **5.58E-04** | 0.58 ± 0.17 |  | 0.50 ± 0.18 | **0.568** | 1.63 ± 3.07 |
| **EO-MSCM-SH vs. FD-COAL** | 0.52 ± 0.17 | **8.27E-04** | 0.58 ± 0.17 |  | 0.50 ± 0.18 | **0.532** | 1.70 ± 3.24 |
| **EO-MSCM-EL vs. EO-SSCM** | 0.50 ± 0.17 | **2.27E-45** | 0.23 ± 0.06 |  | 0.50 ± 0.19 | **1.46E-16** | 0.29 ± 0.10 |
| **EO-MSCM-EL vs. FD-SSCM** | 0.50 ± 0.17 | **1.11E-29** | 0.31 ± 0.12 |  | 0.50 ± 0.19 | **1.20E-07** | 0.40 ± 0.18 |
| **EO-MSCM-EL vs. QUBIC** | 0.50 ± 0.17 | **4.46E-49** | 1.51 ± 0.29 |  | 0.50 ± 0.19 | **2.80E-23** | 1.81 ± 0.85 |
| **EO-MSCM-EL vs. EO-COAL** | 0.50 ± 0.17 | **5.24E-05** | 0.58 ± 0.17 |  | 0.50 ± 0.19 | **0.810** | 1.63 ± 3.07 |
| **EO-MSCM-EL vs. FD-COAL** | 0.50 ± 0.17 | **6.24E-05** | 0.58 ± 0.17 |  | 0.50 ± 0.19 | **0.753** | 1.70 ± 3.24 |
| **EO-MSCM-SH vs. MSKM-SH** | 0.52 ± 0.17 | **1.15E-10** | 0.40 ± 0.08 |  | 0.50 ± 0.18 | **0.002** | 0.43 ± 0.08 |
| **EO-MSCM-SH vs. MSKM-EL** | 0.52 ± 0.17 | **1.26E-11** | 0.39 ± 0.07 |  | 0.50 ± 0.18 | **4.66E-04** | 0.43 ± 0.08 |
| **EO-MSCM-EL vs. MSKM-SH** | 0.50 ± 0.17 | **6.56E-09** | 0.40 ± 0.08 |  | 0.50 ± 0.19 | **0.010** | 0.43 ± 0.08 |
| **EO-MSCM-EL vs. MSKM-EL** | 0.50 ± 0.17 | **1.83E-09** | 0.39 ± 0.07 |  | 0.50 ± 0.19 | **0.003** | 0.43 ± 0.08 |

#### Mean correlations

Table S10: Comparison of bicluster mean correlations from the expression only methods considered by this study for all pairings of B. subtilis, B. anthracis and L. monocytogenes. A comparison of the mean correlations of the results from MSCM (expression only) with all other relevant methods for all 3 pairings of the three organisms examined. In the comparisons, we compare both MSCM steps to the other methods. Displayed are the means for each method and/or step compared, as well as the Wilcoxon’s non-parametric rank test (2-sided) comparing their distributions. We direct the reader to 4 for instructions on how to interpret the table. In this case, these results illustrate that in 65 of the 116 comparisons (56%) MSCM step did as well or better than its competitors.

| ***B. subtilis - B. anthracis pairing*** |  | ***B. subtilis*** |  |  | ***B. anthracis*** | | |
| --- | --- | --- | --- | --- | --- | --- | --- |
| **dist1 vs. dist2** | **dist1 mean (green)** | **Wilcoxon's  2-sided** | **dist2 mean (red)** |  | **dist1 mean (green)** | **Wilcoxon's  2-sided** | **dist2 mean (red)** |
| **EO-MSCM-SH vs. EO-SSCM** | 0.52 ± 0.12 | **2.25E-29** | 0.70 ± 0.11 |  | 0.69 ± 0.17 | **1.02E-34** | 0.91 ± 0.05 |
| **EO-MSCM-SH vs. FD-SSCM** | 0.52 ± 0.12 | **0.007** | 0.56 ± 0.14 |  | 0.69 ± 0.17 | **7.13E-15** | 0.82 ± 0.15 |
| **EO-MSCM-SH vs. QUBIC** | 0.52 ± 0.12 | **1.23E-21** | 0.36 ± 0.19 |  | 0.69 ± 0.17 | **4.14E-23** | 0.49 ± 0.05 |
| **EO-MSCM-SH vs. EO-COAL** | 0.52 ± 0.12 | **5.25E-05** | 0.58 ± 0.14 |  | 0.69 ± 0.17 | **0.002** | 0.64 ± 0.13 |
| **EO-MSCM-SH vs. FD-COAL** | 0.52 ± 0.12 | **2.49E-05** | 0.59 ± 0.15 |  | 0.69 ± 0.17 | **1.05E-04** | 0.62 ± 0.13 |
| **EO-MSCM-EL vs. EO-SSCM** | 0.54 ± 0.12 | **2.67E-26** | 0.70 ± 0.11 |  | 0.69 ± 0.19 | **2.90E-31** | 0.91 ± 0.05 |
| **EO-MSCM-EL vs. FD-SSCM** | 0.54 ± 0.12 | **0.098** | 0.56 ± 0.14 |  | 0.69 ± 0.19 | **4.09E-13** | 0.82 ± 0.15 |
| **EO-MSCM-EL vs. QUBIC** | 0.54 ± 0.12 | **3.57E-23** | 0.36 ± 0.19 |  | 0.69 ± 0.19 | **3.11E-19** | 0.49 ± 0.05 |
| **EO-MSCM-EL vs. EO-COAL** | 0.54 ± 0.12 | **0.003** | 0.58 ± 0.14 |  | 0.69 ± 0.19 | **0.004** | 0.64 ± 0.13 |
| **EO-MSCM-EL vs. FD-COAL** | 0.54 ± 0.12 | **0.001** | 0.59 ± 0.15 |  | 0.69 ± 0.19 | **2.44E-04** | 0.62 ± 0.13 |
| **EO-MSCM-SH vs. MSISA-P** | 0.52 ± 0.12 | **0.003** | 0.60 ± 0.14 |  | 0.69 ± 0.17 | **5.35E-06** | 0.56 ± 0.07 |
| **EO-MSCM-SH vs. MSISA-R** | 0.52 ± 0.12 | **0.304** | 0.55 ± 0.13 |  | 0.69 ± 0.17 | **7.54E-10** | 0.51 ± 0.03 |
| **EO-MSCM-SH vs. MSKM-SH** | 0.52 ± 0.12 | **2.38E-05** | 0.58 ± 0.11 |  | 0.69 ± 0.17 | **1.58E-14** | 0.52 ± 0.14 |
| **EO-MSCM-SH vs. MSKM-EL** | 0.52 ± 0.12 | **0.003** | 0.56 ± 0.11 |  | 0.69 ± 0.17 | **4.87E-07** | 0.58 ± 0.15 |
| **EO-MSCM-SH vs. BMSKM-SH** | 0.52 ± 0.12 | **0.028** | 0.49 ± 0.13 |  | 0.69 ± 0.17 | **0.265** | 0.72 ± 0.10 |
| **EO-MSCM-SH vs. BMSKM-EL** | 0.52 ± 0.12 | **0.107** | 0.50 ± 0.12 |  | 0.69 ± 0.17 | **0.341** | 0.71 ± 0.10 |
| **EO-MSCM-EL vs. MSISA-P** | 0.54 ± 0.12 | **0.020** | 0.60 ± 0.14 |  | 0.69 ± 0.19 | **2.01E-05** | 0.56 ± 0.07 |
| **EO-MSCM-EL vs. MSISA-R** | 0.54 ± 0.12 | **0.629** | 0.55 ± 0.13 |  | 0.69 ± 0.19 | **4.92E-08** | 0.51 ± 0.03 |
| **EO-MSCM-EL vs. MSKM-SH** | 0.54 ± 0.12 | **0.002** | 0.58 ± 0.11 |  | 0.69 ± 0.19 | **2.01E-13** | 0.52 ± 0.14 |
| **EO-MSCM-EL vs. MSKM-EL** | 0.54 ± 0.12 | **0.057** | 0.56 ± 0.11 |  | 0.69 ± 0.19 | **1.23E-06** | 0.58 ± 0.15 |
| **EO-MSCM-EL vs. BMSKM-SH** | 0.54 ± 0.12 | **0.002** | 0.49 ± 0.13 |  | 0.69 ± 0.19 | **0.346** | 0.72 ± 0.10 |
| **EO-MSCM-EL vs. BMSKM-EL** | 0.54 ± 0.12 | **0.012** | 0.50 ± 0.12 |  | 0.69 ± 0.19 | **0.413** | 0.71 ± 0.10 |
|  |  |  |  |  |  |  |  |
|  |  |  |  |  |  |  |  |
| ***B. subtilis - L. monocytogenes pairing*** |  | ***B. subtilis*** |  |  | ***L. monocytogenes*** | | |
| **dist1 vs. dist2** | **dist1 mean (green)** | **Wilcoxon's  2-sided** | **dist2 mean (red)** |  | **dist1 mean (green)** | **Wilcoxon's  2-sided** | **dist2 mean (red)** |
| **EO-MSCM-SH vs. EO-SSCM** | 0.52 ± 0.13 | **3.09E-28** | 0.70 ± 0.11 |  | 0.64 ± 0.18 | **1.43E-18** | 0.86 ± 0.08 |
| **EO-MSCM-SH vs. FD-SSCM** | 0.52 ± 0.13 | **0.004** | 0.56 ± 0.14 |  | 0.64 ± 0.18 | **1.67E-04** | 0.71 ± 0.20 |
| **EO-MSCM-SH vs. QUBIC** | 0.52 ± 0.13 | **3.89E-21** | 0.36 ± 0.19 |  | 0.64 ± 0.18 | **1.06E-13** | 0.45 ± 0.27 |
| **EO-MSCM-SH vs. EO-COAL** | 0.52 ± 0.13 | **2.15E-05** | 0.58 ± 0.14 |  | 0.64 ± 0.18 | **1.37E-09** | 0.81 ± 0.13 |
| **EO-MSCM-SH vs. FD-COAL** | 0.52 ± 0.13 | **9.27E-06** | 0.59 ± 0.15 |  | 0.64 ± 0.18 | **5.02E-09** | 0.80 ± 0.12 |
| **EO-MSCM-EL vs. EO-SSCM** | 0.54 ± 0.12 | **1.67E-24** | 0.70 ± 0.11 |  | 0.64 ± 0.18 | **2.87E-19** | 0.86 ± 0.08 |
| **EO-MSCM-EL vs. FD-SSCM** | 0.54 ± 0.12 | **0.187** | 0.56 ± 0.14 |  | 0.64 ± 0.18 | **8.67E-05** | 0.71 ± 0.20 |
| **EO-MSCM-EL vs. QUBIC** | 0.54 ± 0.12 | **1.86E-23** | 0.36 ± 0.19 |  | 0.64 ± 0.18 | **2.98E-13** | 0.45 ± 0.27 |
| **EO-MSCM-EL vs. EO-COAL** | 0.54 ± 0.12 | **0.007** | 0.58 ± 0.14 |  | 0.64 ± 0.18 | **8.12E-10** | 0.81 ± 0.13 |
| **EO-MSCM-EL vs. FD-COAL** | 0.54 ± 0.12 | **0.003** | 0.59 ± 0.15 |  | 0.64 ± 0.18 | **1.67E-09** | 0.80 ± 0.12 |
| **EO-MSCM-SH vs. MSISA-P** | 0.52 ± 0.13 | **0.099** | 0.60 ± 0.20 |  | 0.64 ± 0.18 | **8.39E-05** | 0.47 ± 0.23 |
| **EO-MSCM-SH vs. MSISA-R** | 0.52 ± 0.13 | **0.268** | 0.55 ± 0.12 |  | 0.64 ± 0.18 | **0.001** | 0.50 ± 0.27 |
| **EO-MSCM-SH vs. MSKM-SH** | 0.52 ± 0.13 | **1.40E-07** | 0.59 ± 0.11 |  | 0.64 ± 0.18 | **7.37E-10** | 0.51 ± 0.17 |
| **EO-MSCM-SH vs. MSKM-EL** | 0.52 ± 0.13 | **0.002** | 0.56 ± 0.11 |  | 0.64 ± 0.18 | **1.87E-05** | 0.55 ± 0.16 |
| **EO-MSCM-SH vs. BMSKM-SH** | 0.52 ± 0.13 | **0.737** | 0.52 ± 0.14 |  | 0.64 ± 0.18 | **0.403** | 0.63 ± 0.15 |
| **EO-MSCM-SH vs. BMSKM-EL** | 0.52 ± 0.13 | **0.311** | 0.53 ± 0.12 |  | 0.64 ± 0.18 | **0.883** | 0.64 ± 0.14 |
| **EO-MSCM-EL vs. MSISA-P** | 0.54 ± 0.12 | **0.324** | 0.60 ± 0.20 |  | 0.64 ± 0.18 | **6.38E-05** | 0.47 ± 0.23 |
| **EO-MSCM-EL vs. MSISA-R** | 0.54 ± 0.12 | **0.874** | 0.55 ± 0.12 |  | 0.64 ± 0.18 | **0.002** | 0.50 ± 0.27 |
| **EO-MSCM-EL vs. MSKM-SH** | 0.54 ± 0.12 | **8.84E-05** | 0.59 ± 0.11 |  | 0.64 ± 0.18 | **4.30E-10** | 0.51 ± 0.17 |
| **EO-MSCM-EL vs. MSKM-EL** | 0.54 ± 0.12 | **0.121** | 0.56 ± 0.11 |  | 0.64 ± 0.18 | **1.46E-05** | 0.55 ± 0.16 |
| **EO-MSCM-EL vs. BMSKM-SH** | 0.54 ± 0.12 | **0.298** | 0.52 ± 0.14 |  | 0.64 ± 0.18 | **0.406** | 0.63 ± 0.15 |
| **EO-MSCM-EL vs. BMSKM-EL** | 0.54 ± 0.12 | **0.578** | 0.53 ± 0.12 |  | 0.64 ± 0.18 | **0.852** | 0.64 ± 0.14 |
|  |  |  |  |  |  |  |  |
|  |  |  |  |  |  |  |  |
|  |  |  |  |  |  |  |  |
|  |  |  |  |  |  |  |  |
| ***B. anthracis - L. monocytogenes pairing*** | ***B. anthracis*** | | |  | ***L. monocytogenes*** | | |
| **dist1 vs. dist2** | **dist1 mean (green)** | **Wilcoxon's  2-sided** | **dist2 mean (red)** |  | **dist1 mean (green)** | **Wilcoxon's  2-sided** | **dist2 mean (red)** |
| **EO-MSCM-SH vs. EO-SSCM** | 0.63 ± 0.16 | **2.22E-48** | 0.91 ± 0.05 |  | 0.63 ± 0.19 | **1.01E-18** | 0.86 ± 0.08 |
| **EO-MSCM-SH vs. FD-SSCM** | 0.63 ± 0.16 | **1.47E-28** | 0.82 ± 0.15 |  | 0.63 ± 0.19 | **2.84E-05** | 0.71 ± 0.20 |
| **EO-MSCM-SH vs. QUBIC** | 0.63 ± 0.16 | **3.47E-14** | 0.49 ± 0.05 |  | 0.63 ± 0.19 | **3.42E-12** | 0.45 ± 0.27 |
| **EO-MSCM-SH vs. EO-COAL** | 0.63 ± 0.16 | **0.824** | 0.64 ± 0.13 |  | 0.63 ± 0.19 | **5.52E-10** | 0.81 ± 0.13 |
| **EO-MSCM-SH vs. FD-COAL** | 0.63 ± 0.16 | **0.341** | 0.62 ± 0.13 |  | 0.63 ± 0.19 | **1.30E-09** | 0.80 ± 0.12 |
| **EO-MSCM-EL vs. EO-SSCM** | 0.63 ± 0.17 | **6.29E-45** | 0.91 ± 0.05 |  | 0.63 ± 0.19 | **1.54E-18** | 0.86 ± 0.08 |
| **EO-MSCM-EL vs. FD-SSCM** | 0.63 ± 0.17 | **1.56E-25** | 0.82 ± 0.15 |  | 0.63 ± 0.19 | **3.54E-05** | 0.71 ± 0.20 |
| **EO-MSCM-EL vs. QUBIC** | 0.63 ± 0.17 | **3.37E-15** | 0.49 ± 0.05 |  | 0.63 ± 0.19 | **1.22E-11** | 0.45 ± 0.27 |
| **EO-MSCM-EL vs. EO-COAL** | 0.63 ± 0.17 | **0.910** | 0.64 ± 0.13 |  | 0.63 ± 0.19 | **5.61E-10** | 0.81 ± 0.13 |
| **EO-MSCM-EL vs. FD-COAL** | 0.63 ± 0.17 | **0.280** | 0.62 ± 0.13 |  | 0.63 ± 0.19 | **1.98E-09** | 0.80 ± 0.12 |
| **EO-MSCM-SH vs. MSKM-SH** | 0.63 ± 0.16 | **0.002** | 0.69 ± 0.12 |  | 0.63 ± 0.19 | **0.250** | 0.60 ± 0.14 |
| **EO-MSCM-SH vs. MSKM-EL** | 0.63 ± 0.16 | **4.39E-05** | 0.70 ± 0.10 |  | 0.63 ± 0.19 | **0.892** | 0.63 ± 0.13 |
| **EO-MSCM-EL vs. MSKM-SH** | 0.63 ± 0.17 | **0.003** | 0.69 ± 0.12 |  | 0.63 ± 0.19 | **0.224** | 0.60 ± 0.14 |
| **EO-MSCM-EL vs. MSKM-EL** | 0.63 ± 0.17 | **1.20E-04** | 0.70 ± 0.10 |  | 0.63 ± 0.19 | **0.817** | 0.63 ± 0.13 |

#### Network Association p-values

Table S11: Comparison of bicluster network association p-values from the expression only methods considered by this study for all pairings of B. subtilis, B. anthracis and L. monocytogenes. A comparison of the residuals of the results from MSCM (expression only) with all other relevant methods for all 3 pairings of the three organisms examined. In the comparisons, we compare both MSCM steps to the other methods. Displayed are the means for each method and/or step compared, as well as the Wilcoxon’s non-parametric rank test (2-sided) comparing their distributions. We direct the reader to 4 for instructions on how to interpret the table. In this case, these results illustrate that in 103 of the 116 comparisons (88.8%) MSCM step did as well or better than its competitors.

| ***B. subtilis - B. anthracis pairing*** |  | ***B. subtilis*** |  |  | ***B. anthracis*** | | |
| --- | --- | --- | --- | --- | --- | --- | --- |
| **dist1 vs. dist2** | **dist1 mean (green)** | **Wilcoxon's  2-sided** | **dist2 mean (red)** |  | **dist1 mean (green)** | **Wilcoxon's  2-sided** | **dist2 mean (red)** |
| **EO-MSCM-SH vs. EO-SSCM** | 6.24 ± 7.63 | **0.435** | 7.63 ± 9.05 |  | 6.45 ± 7.29 | **0.416** | 5.39 ± 6.03 |
| **EO-MSCM-SH vs. FD-SSCM** | 6.24 ± 7.63 | **2.07E-05** | 9.78 ± 9.76 |  | 6.45 ± 7.29 | **0.721** | 5.44 ± 5.38 |
| **EO-MSCM-SH vs. QUBIC** | 6.24 ± 7.63 | **6.42E-16** | 2.52 ± 4.78 |  | 6.45 ± 7.29 | **0.938** | 6.73 ± 7.52 |
| **EO-MSCM-SH vs. EO-COAL** | 6.24 ± 7.63 | **0.568** | 7.75 ± 9.50 |  | 6.45 ± 7.29 | **0.198** | 6.18 ± 7.98 |
| **EO-MSCM-SH vs. FD-COAL** | 6.24 ± 7.63 | **0.451** | 7.57 ± 9.16 |  | 6.45 ± 7.29 | **0.144** | 6.50 ± 8.74 |
| **EO-MSCM-EL vs. EO-SSCM** | 6.32 ± 7.80 | **0.328** | 7.63 ± 9.05 |  | 6.24 ± 7.31 | **0.716** | 5.39 ± 6.03 |
| **EO-MSCM-EL vs. FD-SSCM** | 6.32 ± 7.80 | **3.91E-06** | 9.78 ± 9.76 |  | 6.24 ± 7.31 | **0.950** | 5.44 ± 5.38 |
| **EO-MSCM-EL vs. QUBIC** | 6.32 ± 7.80 | **6.13E-16** | 2.52 ± 4.78 |  | 6.24 ± 7.31 | **0.702** | 6.73 ± 7.52 |
| **EO-MSCM-EL vs. EO-COAL** | 6.32 ± 7.80 | **0.402** | 7.75 ± 9.50 |  | 6.24 ± 7.31 | **0.316** | 6.18 ± 7.98 |
| **EO-MSCM-EL vs. FD-COAL** | 6.32 ± 7.80 | **0.330** | 7.57 ± 9.16 |  | 6.24 ± 7.31 | **0.226** | 6.50 ± 8.74 |
| **EO-MSCM-SH vs. MSISA-P** | 6.24 ± 7.63 | **0.490** | 5.56 ± 5.86 |  | 6.45 ± 7.29 | **0.814** | 5.61 ± 7.17 |
| **EO-MSCM-SH vs. MSISA-R** | 6.24 ± 7.63 | **4.21E-04** | 9.69 ± 9.37 |  | 6.45 ± 7.29 | **0.464** | 9.66 ± 9.95 |
| **EO-MSCM-SH vs. MSKM-SH** | 6.24 ± 7.63 | **0.190** | 7.87 ± 9.35 |  | 6.45 ± 7.29 | **0.060** | 4.38 ± 5.10 |
| **EO-MSCM-SH vs. MSKM-EL** | 6.24 ± 7.63 | **0.137** | 8.15 ± 9.65 |  | 6.45 ± 7.29 | **0.005** | 4.06 ± 5.32 |
| **EO-MSCM-SH vs. BMSKM-SH** | 6.24 ± 7.63 | **0.110** | 7.27 ± 8.25 |  | 6.45 ± 7.29 | **0.489** | 5.54 ± 6.48 |
| **EO-MSCM-SH vs. BMSKM-EL** | 6.24 ± 7.63 | **0.346** | 6.93 ± 8.19 |  | 6.45 ± 7.29 | **0.024** | 4.56 ± 5.86 |
| **EO-MSCM-EL vs. MSISA-P** | 6.32 ± 7.80 | **0.429** | 5.56 ± 5.86 |  | 6.24 ± 7.31 | **0.935** | 5.61 ± 7.17 |
| **EO-MSCM-EL vs. MSISA-R** | 6.32 ± 7.80 | **0.000** | 9.69 ± 9.37 |  | 6.24 ± 7.31 | **0.282** | 9.66 ± 9.95 |
| **EO-MSCM-EL vs. MSKM-SH** | 6.32 ± 7.80 | **0.130** | 7.87 ± 9.35 |  | 6.24 ± 7.31 | **0.113** | 4.38 ± 5.10 |
| **EO-MSCM-EL vs. MSKM-EL** | 6.32 ± 7.80 | **0.090** | 8.15 ± 9.65 |  | 6.24 ± 7.31 | **0.012** | 4.06 ± 5.32 |
| **EO-MSCM-EL vs. BMSKM-SH** | 6.32 ± 7.80 | **0.063** | 7.27 ± 8.25 |  | 6.24 ± 7.31 | **0.676** | 5.54 ± 6.48 |
| **EO-MSCM-EL vs. BMSKM-EL** | 6.32 ± 7.80 | **0.247** | 6.93 ± 8.19 |  | 6.24 ± 7.31 | **0.054** | 4.56 ± 5.86 |
|  |  |  |  |  |  |  |  |
|  |  |  |  |  |  |  |  |
| ***B. subtilis - L. monocytogenes pairing*** |  | ***B. subtilis*** |  |  | ***L. monocytogenes*** | | |
| **dist1 vs. dist2** | **dist1 mean (green)** | **Wilcoxon's  2-sided** | **dist2 mean (red)** |  | **dist1 mean (green)** | **Wilcoxon's  2-sided** | **dist2 mean (red)** |
| **EO-MSCM-SH vs. EO-SSCM** | 9.11 ± 10.61 | **0.419** | 7.63 ± 9.05 |  | 6.31 ± 8.71 | **0.594** | 4.42 ± 6.01 |
| **EO-MSCM-SH vs. FD-SSCM** | 9.11 ± 10.61 | **0.058** | 9.78 ± 9.76 |  | 6.31 ± 8.71 | **0.003** | 6.90 ± 7.75 |
| **EO-MSCM-SH vs. QUBIC** | 9.11 ± 10.61 | **8.39E-16** | 2.52 ± 4.78 |  | 6.31 ± 8.71 | **2.23E-05** | 9.95 ± 10.82 |
| **EO-MSCM-SH vs. EO-COAL** | 9.11 ± 10.61 | **0.279** | 7.75 ± 9.50 |  | 6.31 ± 8.71 | **0.422** | 5.24 ± 7.41 |
| **EO-MSCM-SH vs. FD-COAL** | 9.11 ± 10.61 | **0.334** | 7.57 ± 9.16 |  | 6.31 ± 8.71 | **0.744** | 5.93 ± 8.27 |
| **EO-MSCM-EL vs. EO-SSCM** | 8.50 ± 10.57 | **0.852** | 7.63 ± 9.05 |  | 6.00 ± 8.15 | **0.546** | 4.42 ± 6.01 |
| **EO-MSCM-EL vs. FD-SSCM** | 8.50 ± 10.57 | **0.002** | 9.78 ± 9.76 |  | 6.00 ± 8.15 | **0.002** | 6.90 ± 7.75 |
| **EO-MSCM-EL vs. QUBIC** | 8.50 ± 10.57 | **1.01E-13** | 2.52 ± 4.78 |  | 6.00 ± 8.15 | **1.34E-05** | 9.95 ± 10.82 |
| **EO-MSCM-EL vs. EO-COAL** | 8.50 ± 10.57 | **0.999** | 7.75 ± 9.50 |  | 6.00 ± 8.15 | **0.440** | 5.24 ± 7.41 |
| **EO-MSCM-EL vs. FD-COAL** | 8.50 ± 10.57 | **0.894** | 7.57 ± 9.16 |  | 6.00 ± 8.15 | **0.751** | 5.93 ± 8.27 |
| **EO-MSCM-SH vs. MSISA-P** | 9.11 ± 10.61 | **0.317** | 9.05 ± 8.89 |  | 6.31 ± 8.71 | **0.533** | 3.70 ± 1.79 |
| **EO-MSCM-SH vs. MSISA-R** | 9.11 ± 10.61 | **0.126** | 9.61 ± 9.29 |  | 6.31 ± 8.71 | **0.077** | 6.20 ± 6.65 |
| **EO-MSCM-SH vs. MSKM-SH** | 9.11 ± 10.61 | **0.199** | 9.76 ± 10.54 |  | 6.31 ± 8.71 | **0.052** | 7.88 ± 9.56 |
| **EO-MSCM-SH vs. MSKM-EL** | 9.11 ± 10.61 | **0.499** | 7.68 ± 9.47 |  | 6.31 ± 8.71 | **0.895** | 4.91 ± 6.44 |
| **EO-MSCM-SH vs. BMSKM-SH** | 9.11 ± 10.61 | **0.504** | 9.23 ± 10.39 |  | 6.31 ± 8.71 | **0.327** | 7.10 ± 9.45 |
| **EO-MSCM-SH vs. BMSKM-EL** | 9.11 ± 10.61 | **0.112** | 6.79 ± 8.75 |  | 6.31 ± 8.71 | **0.960** | 4.86 ± 6.39 |
| **EO-MSCM-EL vs. MSISA-P** | 8.50 ± 10.57 | **0.139** | 9.05 ± 8.89 |  | 6.00 ± 8.15 | **0.603** | 3.70 ± 1.79 |
| **EO-MSCM-EL vs. MSISA-R** | 8.50 ± 10.57 | **0.015** | 9.61 ± 9.29 |  | 6.00 ± 8.15 | **0.061** | 6.20 ± 6.65 |
| **EO-MSCM-EL vs. MSKM-SH** | 8.50 ± 10.57 | **0.022** | 9.76 ± 10.54 |  | 6.00 ± 8.15 | **0.038** | 7.88 ± 9.56 |
| **EO-MSCM-EL vs. MSKM-EL** | 8.50 ± 10.57 | **0.712** | 7.68 ± 9.47 |  | 6.00 ± 8.15 | **0.873** | 4.91 ± 6.44 |
| **EO-MSCM-EL vs. BMSKM-SH** | 8.50 ± 10.57 | **0.110** | 9.23 ± 10.39 |  | 6.00 ± 8.15 | **0.304** | 7.10 ± 9.45 |
| **EO-MSCM-EL vs. BMSKM-EL** | 8.50 ± 10.57 | **0.548** | 6.79 ± 8.75 |  | 6.00 ± 8.15 | **0.932** | 4.86 ± 6.39 |
|  |  |  |  |  |  |  |  |
|  |  |  |  |  |  |  |  |
| ***B. anthracis - L. monocytogenes pairing*** | ***B. anthracis*** | | |  | ***L. monocytogenes*** | | |
| **dist1 vs. dist2** | **dist1 mean (green)** | **Wilcoxon's  2-sided** | **dist2 mean (red)** |  | **dist1 mean (green)** | **Wilcoxon's  2-sided** | **dist2 mean (red)** |
| **EO-MSCM-SH vs. EO-SSCM** | 6.75 ± 9.09 | **0.621** | 5.39 ± 6.03 |  | 5.63 ± 8.48 | **0.982** | 4.42 ± 6.01 |
| **EO-MSCM-SH vs. FD-SSCM** | 6.75 ± 9.09 | **0.440** | 5.47 ± 5.39 |  | 5.63 ± 8.48 | **6.20E-05** | 6.90 ± 7.75 |
| **EO-MSCM-SH vs. QUBIC** | 6.75 ± 9.09 | **0.392** | 6.73 ± 7.52 |  | 5.63 ± 8.48 | **1.87E-07** | 9.95 ± 10.82 |
| **EO-MSCM-SH vs. EO-COAL** | 6.75 ± 9.09 | **0.794** | 6.18 ± 7.98 |  | 5.63 ± 8.48 | **0.687** | 5.24 ± 7.41 |
| **EO-MSCM-SH vs. FD-COAL** | 6.75 ± 9.09 | **0.623** | 6.50 ± 8.74 |  | 5.63 ± 8.48 | **0.929** | 5.93 ± 8.27 |
| **EO-MSCM-EL vs. EO-SSCM** | 6.82 ± 8.86 | **0.872** | 5.39 ± 6.03 |  | 5.93 ± 8.70 | **0.859** | 4.42 ± 6.01 |
| **EO-MSCM-EL vs. FD-SSCM** | 6.82 ± 8.86 | **0.653** | 5.47 ± 5.39 |  | 5.93 ± 8.70 | **1.29E-04** | 6.90 ± 7.75 |
| **EO-MSCM-EL vs. QUBIC** | 6.82 ± 8.86 | **0.514** | 6.73 ± 7.52 |  | 5.93 ± 8.70 | **3.52E-07** | 9.95 ± 10.82 |
| **EO-MSCM-EL vs. EO-COAL** | 6.82 ± 8.86 | **0.555** | 6.18 ± 7.98 |  | 5.93 ± 8.70 | **0.533** | 5.24 ± 7.41 |
| **EO-MSCM-EL vs. FD-COAL** | 6.82 ± 8.86 | **0.451** | 6.50 ± 8.74 |  | 5.93 ± 8.70 | **0.959** | 5.93 ± 8.27 |
| **EO-MSCM-SH vs. MSKM-SH** | 6.75 ± 9.09 | **0.623** | 5.67 ± 7.00 |  | 5.63 ± 8.48 | **0.087** | 6.83 ± 8.86 |
| **EO-MSCM-SH vs. MSKM-EL** | 6.75 ± 9.09 | **0.401** | 3.86 ± 4.13 |  | 5.63 ± 8.48 | **0.693** | 4.94 ± 6.73 |
| **EO-MSCM-EL vs. MSKM-SH** | 6.82 ± 8.86 | **0.749** | 5.67 ± 7.00 |  | 5.93 ± 8.70 | **0.118** | 6.83 ± 8.86 |
| **EO-MSCM-EL vs. MSKM-EL** | 6.82 ± 8.86 | **0.190** | 3.86 ± 4.13 |  | 5.93 ± 8.70 | **0.847** | 4.94 ± 6.73 |

#### Motif E-values

Table S12: Comparison of bicluster motif E-values (-log10) from the expression only methods considered by this study for all pairings of B. subtilis, B. anthracis and L. monocytogenes. A comparison of the motif E-values (-log10) from MSCM (expression only) with all other relevant methods for all 3 pairings of the three organisms examined. In the comparisons, we compare both MSCM steps to the other methods. Displayed are the means for each method and/or step compared, as well as the Wilcoxon’s non-parametric rank test (2-sided) comparing their distributions. We direct the reader to 4 for instructions on how to interpret the table. As the table indicates, in 39 of the 116 of the comparisons (33.7%) MSCM does as well or better than its competitors. This is by far the metric that EO-MSCM does on.

| ***B. subtilis - B. anthracis pairing*** | ***B. subtilis*** | | |  | ***B. anthracis*** | | |
| --- | --- | --- | --- | --- | --- | --- | --- |
| **dist1 vs. dist2** | **dist1 mean (green)** | **Wilcoxon's  2-sided** | **dist2 mean (red)** |  | **dist1 mean (green)** | **Wilcoxon's  2-sided** | **dist2 mean (red)** |
| **EO-MSCM-SH vs. EO-SSCM** | -0.90 ± 3.44 | **0.026** | 0.71 ± 5.69 |  | 0.09 ± 4.65 | **6.24E-07** | 2.17 ± 5.38 |
| **EO-MSCM-SH vs. FD-SSCM** | -0.90 ± 3.44 | **2.01E-28** | 7.03 ± 18.81 |  | 0.09 ± 4.65 | **3.33E-09** | 4.98 ± 10.63 |
| **EO-MSCM-SH vs. QUBIC** | -0.90 ± 3.44 | **9.92E-13** | 1.41 ± 3.94 |  | 0.09 ± 4.65 | **1.28E-09** | 13.72 ± 14.49 |
| **EO-MSCM-SH vs. EO-COAL** | -0.90 ± 3.44 | **4.24E-08** | 2.40 ± 7.33 |  | 0.09 ± 4.65 | **1.56E-04** | 3.94 ± 8.70 |
| **EO-MSCM-SH vs. FD-COAL** | -0.90 ± 3.44 | **2.22E-08** | 2.72 ± 7.30 |  | 0.09 ± 4.65 | **0.001** | 3.85 ± 8.83 |
| **EO-MSCM-EL vs. EO-SSCM** | -0.20 ± 4.56 | **0.157** | 0.71 ± 5.69 |  | 1.02 ± 6.11 | **3.26E-04** | 2.17 ± 5.38 |
| **EO-MSCM-EL vs. FD-SSCM** | -0.20 ± 4.56 | **4.99E-22** | 7.03 ± 18.81 |  | 1.02 ± 6.11 | **2.59E-06** | 4.98 ± 10.63 |
| **EO-MSCM-EL vs. QUBIC** | -0.20 ± 4.56 | **4.60E-09** | 1.41 ± 3.94 |  | 1.02 ± 6.11 | **9.04E-09** | 13.72 ± 14.49 |
| **EO-MSCM-EL vs. EO-COAL** | -0.20 ± 4.56 | **1.25E-05** | 2.40 ± 7.33 |  | 1.02 ± 6.11 | **0.005** | 3.94 ± 8.70 |
| **EO-MSCM-EL vs. FD-COAL** | -0.20 ± 4.56 | **5.86E-06** | 2.72 ± 7.30 |  | 1.02 ± 6.11 | **0.022** | 3.85 ± 8.83 |
| **EO-MSCM-SH vs. MSISA-P** | -0.90 ± 3.44 | **0.295** | -1.12 ± 2.03 |  | 0.09 ± 4.65 | **0.120** | 0.46 ± 3.43 |
| **EO-MSCM-SH vs. MSISA-R** | -0.90 ± 3.44 | **3.54E-13** | 9.40 ± 9.19 |  | 0.09 ± 4.65 | **2.25E-05** | 2.34 ± 11.56 |
| **EO-MSCM-SH vs. MSKM-SH** | -0.90 ± 3.44 | **0.915** | -1.18 ± 2.62 |  | 0.09 ± 4.65 | **0.294** | -0.22 ± 2.96 |
| **EO-MSCM-SH vs. MSKM-EL** | -0.90 ± 3.44 | **0.002** | 0.19 ± 4.26 |  | 0.09 ± 4.65 | **3.74E-09** | 2.74 ± 5.66 |
| **EO-MSCM-SH vs. BMSKM-SH** | -0.90 ± 3.44 | **0.620** | -1.09 ± 2.68 |  | 0.09 ± 4.65 | **0.639** | -0.39 ± 2.87 |
| **EO-MSCM-SH vs. BMSKM-EL** | -0.90 ± 3.44 | **8.72E-05** | 0.44 ± 4.06 |  | 0.09 ± 4.65 | **1.18E-11** | 3.07 ± 5.44 |
| **EO-MSCM-EL vs. MSISA-P** | -0.20 ± 4.56 | **0.701** | -1.12 ± 2.03 |  | 1.02 ± 6.11 | **0.434** | 0.46 ± 3.43 |
| **EO-MSCM-EL vs. MSISA-R** | -0.20 ± 4.56 | **1.40E-11** | 9.40 ± 9.19 |  | 1.02 ± 6.11 | **1.57E-05** | 2.34 ± 11.56 |
| **EO-MSCM-EL vs. MSKM-SH** | -0.20 ± 4.56 | **0.353** | -1.18 ± 2.62 |  | 1.02 ± 6.11 | **0.957** | -0.22 ± 2.96 |
| **EO-MSCM-EL vs. MSKM-EL** | -0.20 ± 4.56 | **0.036** | 0.19 ± 4.26 |  | 1.02 ± 6.11 | **4.10E-06** | 2.74 ± 5.66 |
| **EO-MSCM-EL vs. BMSKM-SH** | -0.20 ± 4.56 | **0.663** | -1.09 ± 2.68 |  | 1.02 ± 6.11 | **0.518** | -0.39 ± 2.87 |
| **EO-MSCM-EL vs. BMSKM-EL** | -0.20 ± 4.56 | **0.006** | 0.44 ± 4.06 |  | 1.02 ± 6.11 | **6.89E-08** | 3.07 ± 5.44 |
|  |  |  |  |  |  |  |  |
|  |  |  |  |  |  |  |  |
| ***B. subtilis - L. monocytogenes pairing*** | ***B. subtilis*** | | |  | ***L. monocytogenes*** | | |
| **dist1 vs. dist2** | **dist1 mean (green)** | **Wilcoxon's  2-sided** | **dist2 mean (red)** |  | **dist1 mean (green)** | **Wilcoxon's  2-sided** | **dist2 mean (red)** |
| **EO-MSCM-SH vs. EO-SSCM** | -1.56 ± 3.27 | **1.06E-05** | 0.71 ± 5.69 |  | 0.29 ± 5.09 | **4.46E-05** | 2.04 ± 4.51 |
| **EO-MSCM-SH vs. FD-SSCM** | -1.56 ± 3.27 | **3.94E-37** | 7.03 ± 18.81 |  | 0.29 ± 5.09 | **0.162** | 0.46 ± 4.73 |
| **EO-MSCM-SH vs. QUBIC** | -1.56 ± 3.27 | **4.26E-19** | 1.41 ± 3.94 |  | 0.29 ± 5.09 | **0.302** | 9.57 ± 13.54 |
| **EO-MSCM-SH vs. EO-COAL** | -1.56 ± 3.27 | **3.88E-13** | 2.40 ± 7.33 |  | 0.29 ± 5.09 | **0.001** | 2.92 ± 6.80 |
| **EO-MSCM-SH vs. FD-COAL** | -1.56 ± 3.27 | **3.68E-13** | 2.72 ± 7.30 |  | 0.29 ± 5.09 | **4.89E-04** | 3.66 ± 7.60 |
| **EO-MSCM-EL vs. EO-SSCM** | -0.95 ± 4.01 | **0.003** | 0.71 ± 5.69 |  | 0.34 ± 5.08 | **8.48E-05** | 2.04 ± 4.51 |
| **EO-MSCM-EL vs. FD-SSCM** | -0.95 ± 4.01 | **1.28E-30** | 7.03 ± 18.81 |  | 0.34 ± 5.08 | **0.121** | 0.46 ± 4.73 |
| **EO-MSCM-EL vs. QUBIC** | -0.95 ± 4.01 | **2.38E-14** | 1.41 ± 3.94 |  | 0.34 ± 5.08 | **0.269** | 9.57 ± 13.54 |
| **EO-MSCM-EL vs. EO-COAL** | -0.95 ± 4.01 | **2.27E-09** | 2.40 ± 7.33 |  | 0.34 ± 5.08 | **0.001** | 2.92 ± 6.80 |
| **EO-MSCM-EL vs. FD-COAL** | -0.95 ± 4.01 | **1.14E-09** | 2.72 ± 7.30 |  | 0.34 ± 5.08 | **0.001** | 3.66 ± 7.60 |
| **EO-MSCM-SH vs. MSISA-P** | -1.56 ± 3.27 | **0.023** | -2.63 ± 1.00 |  | 0.29 ± 5.09 | **0.011** | -1.56 ± 1.40 |
| **EO-MSCM-SH vs. MSISA-R** | -1.56 ± 3.27 | **6.06E-17** | 10.37 ± 8.84 |  | 0.29 ± 5.09 | **1.01E-09** | 9.06 ± 7.74 |
| **EO-MSCM-SH vs. MSKM-SH** | -1.56 ± 3.27 | **0.592** | -1.91 ± 1.56 |  | 0.29 ± 5.09 | **0.617** | -0.83 ± 1.64 |
| **EO-MSCM-SH vs. MSKM-EL** | -1.56 ± 3.27 | **5.02E-06** | 0.52 ± 5.33 |  | 0.29 ± 5.09 | **0.002** | 0.36 ± 2.68 |
| **EO-MSCM-SH vs. BMSKM-SH** | -1.56 ± 3.27 | **0.913** | -1.97 ± 1.58 |  | 0.29 ± 5.09 | **0.284** | -0.89 ± 1.69 |
| **EO-MSCM-SH vs. BMSKM-EL** | -1.56 ± 3.27 | **2.33E-06** | 0.02 ± 3.81 |  | 0.29 ± 5.09 | **0.002** | 0.43 ± 3.17 |
| **EO-MSCM-EL vs. MSISA-P** | -0.95 ± 4.01 | **0.002** | -2.63 ± 1.00 |  | 0.34 ± 5.08 | **0.008** | -1.56 ± 1.40 |
| **EO-MSCM-EL vs. MSISA-R** | -0.95 ± 4.01 | **1.89E-15** | 10.37 ± 8.84 |  | 0.34 ± 5.08 | **1.08E-09** | 9.06 ± 7.74 |
| **EO-MSCM-EL vs. MSKM-SH** | -0.95 ± 4.01 | **0.292** | -1.91 ± 1.56 |  | 0.34 ± 5.08 | **0.459** | -0.83 ± 1.64 |
| **EO-MSCM-EL vs. MSKM-EL** | -0.95 ± 4.01 | **0.001** | 0.52 ± 5.33 |  | 0.34 ± 5.08 | **0.004** | 0.36 ± 2.68 |
| **EO-MSCM-EL vs. BMSKM-SH** | -0.95 ± 4.01 | **0.143** | -1.97 ± 1.58 |  | 0.34 ± 5.08 | **0.196** | -0.89 ± 1.69 |
| **EO-MSCM-EL vs. BMSKM-EL** | -0.95 ± 4.01 | **0.001** | 0.02 ± 3.81 |  | 0.34 ± 5.08 | **0.004** | 0.43 ± 3.17 |
|  |  |  |  |  |  |  |  |
|  |  |  |  |  |  |  |  |
| ***B. anthracis - L. monocytogenes pairing*** | ***B. anthracis*** | | |  | ***L. monocytogenes*** | | |
| **dist1 vs. dist2** | **dist1 mean (green)** | **Wilcoxon's  2-sided** | **dist2 mean (red)** |  | **dist1 mean (green)** | **Wilcoxon's  2-sided** | **dist2 mean (red)** |
| **EO-MSCM-SH vs. EO-SSCM** | -0.81 ± 3.42 | **8.48E-12** | 2.17 ± 5.38 |  | 0.50 ± 5.37 | **1.24E-04** | 2.04 ± 4.51 |
| **EO-MSCM-SH vs. FD-SSCM** | -0.81 ± 3.42 | **1.14E-15** | 5.21 ± 10.80 |  | 0.50 ± 5.37 | **0.161** | 0.46 ± 4.73 |
| **EO-MSCM-SH vs. QUBIC** | -0.81 ± 3.42 | **3.82E-10** | 13.72 ± 14.49 |  | 0.50 ± 5.37 | **0.333** | 9.57 ± 13.54 |
| **EO-MSCM-SH vs. EO-COAL** | -0.81 ± 3.42 | **4.78E-07** | 3.94 ± 8.70 |  | 0.50 ± 5.37 | **0.002** | 2.92 ± 6.80 |
| **EO-MSCM-SH vs. FD-COAL** | -0.81 ± 3.42 | **8.93E-06** | 3.85 ± 8.83 |  | 0.50 ± 5.37 | **0.001** | 3.66 ± 7.60 |
| **EO-MSCM-EL vs. EO-SSCM** | -0.42 ± 3.90 | **1.18E-09** | 2.17 ± 5.38 |  | 0.99 ± 6.39 | **0.001** | 2.04 ± 4.51 |
| **EO-MSCM-EL vs. FD-SSCM** | -0.42 ± 3.90 | **2.52E-13** | 5.21 ± 10.80 |  | 0.99 ± 6.39 | **0.020** | 0.46 ± 4.73 |
| **EO-MSCM-EL vs. QUBIC** | -0.42 ± 3.90 | **1.15E-09** | 13.72 ± 14.49 |  | 0.99 ± 6.39 | **0.398** | 9.57 ± 13.54 |
| **EO-MSCM-EL vs. EO-COAL** | -0.42 ± 3.90 | **6.80E-06** | 3.94 ± 8.70 |  | 0.99 ± 6.39 | **0.012** | 2.92 ± 6.80 |
| **EO-MSCM-EL vs. FD-COAL** | -0.42 ± 3.90 | **8.02E-05** | 3.85 ± 8.83 |  | 0.99 ± 6.39 | **0.008** | 3.66 ± 7.60 |
| **EO-MSCM-SH vs. MSKM-SH** | -0.81 ± 3.42 | **0.595** | -1.58 ± 1.60 |  | 0.50 ± 5.37 | **0.940** | -0.67 ± 1.69 |
| **EO-MSCM-SH vs. MSKM-EL** | -0.81 ± 3.42 | **1.22E-09** | 2.52 ± 6.60 |  | 0.50 ± 5.37 | **0.009** | 0.44 ± 3.00 |
| **EO-MSCM-EL vs. MSKM-SH** | -0.42 ± 3.90 | **0.201** | -1.58 ± 1.60 |  | 0.99 ± 6.39 | **0.271** | -0.67 ± 1.69 |
| **EO-MSCM-EL vs. MSKM-EL** | -0.42 ± 3.90 | **5.61E-08** | 2.52 ± 6.60 |  | 0.99 ± 6.39 | **0.108** | 0.44 ± 3.00 |

#### Sequence p-values

Table S13: Comparison of bicluster sequence p-values (-log10) from the expression only methods considered by this study for all pairings of B. subtilis, B. anthracis and L. monocytogenes. A comparison of the sequence p-values (-log10) from MSCM (expression only) with all other relevant methods for all 3 pairings of the three organisms examined. In the comparisons, we compare both MSCM steps to the other methods. Displayed are the means for each method and/or step compared, as well as the Wilcoxon’s non-parametric rank test (2-sided) comparing their distributions. We direct the reader to 4 for instructions on how to interpret the table. As the table indicates, in 72 of the 92 of the comparisons (78.3%) MSCM does as well or better than its competitors.

| ***B. subtilis - B. anthracis pairing*** | ***B. subtilis*** | | |  | ***B. anthracis*** | | |
| --- | --- | --- | --- | --- | --- | --- | --- |
| **dist1 vs. dist2** | **dist1 mean (green)** | **Wilcoxon's  2-sided** | **dist2 mean (red)** |  | **dist1 mean (green)** | **Wilcoxon's  2-sided** | **dist2 mean (red)** |
| **EO-MSCM-SH vs. EO-SSCM** | 4.11 ± 2.04 | **0.026** | 3.68 ± 1.88 |  | 3.77 ± 1.69 | **4.87E-07** | 2.92 ± 1.17 |
| **EO-MSCM-SH vs. FD-SSCM** | 4.11 ± 2.04 | **7.33E-18** | 6.73 ± 3.35 |  | 3.77 ± 1.69 | **0.097** | 3.90 ± 2.62 |
| **EO-MSCM-SH vs. QUBIC** | 4.11 ± 2.04 | **7.49E-27** | 2.06 ± 0.50 |  | 3.77 ± 1.69 | **6.20E-31** | 1.77 ± 0.26 |
| **EO-MSCM-SH vs. EO-COAL** | 4.11 ± 2.04 | **4.30E-21** | 2.50 ± 1.30 |  | 3.77 ± 1.69 | **1.25E-16** | 2.36 ± 1.16 |
| **EO-MSCM-SH vs. FD-COAL** | 4.11 ± 2.04 | **1.07E-20** | 2.47 ± 1.12 |  | 3.77 ± 1.69 | **2.74E-18** | 2.32 ± 1.57 |
| **EO-MSCM-EL vs. EO-SSCM** | 3.86 ± 1.82 | **0.235** | 3.68 ± 1.88 |  | 3.55 ± 1.73 | **7.07E-04** | 2.92 ± 1.17 |
| **EO-MSCM-EL vs. FD-SSCM** | 3.86 ± 1.82 | **4.50E-21** | 6.73 ± 3.35 |  | 3.55 ± 1.73 | **0.831** | 3.90 ± 2.62 |
| **EO-MSCM-EL vs. QUBIC** | 3.86 ± 1.82 | **1.00E-24** | 2.06 ± 0.50 |  | 3.55 ± 1.73 | **1.51E-24** | 1.77 ± 0.26 |
| **EO-MSCM-EL vs. EO-COAL** | 3.86 ± 1.82 | **4.31E-18** | 2.50 ± 1.30 |  | 3.55 ± 1.73 | **5.34E-12** | 2.36 ± 1.16 |
| **EO-MSCM-EL vs. FD-COAL** | 3.86 ± 1.82 | **1.79E-17** | 2.47 ± 1.12 |  | 3.55 ± 1.73 | **9.21E-14** | 2.32 ± 1.57 |
| **EO-MSCM-SH vs. MSISA-P** | 4.11 ± 2.04 | **0.166** | 3.65 ± 1.74 |  | 3.77 ± 1.69 | **0.152** | 3.34 ± 1.33 |
| **EO-MSCM-SH vs. MSISA-R** | 4.11 ± 2.04 | **1.18E-12** | 2.02 ± 0.52 |  | 3.77 ± 1.69 | **1.67E-06** | 1.79 ± 0.27 |
| **EO-MSCM-SH vs. MSKM-SH** | 4.11 ± 2.04 | **0.742** | 3.97 ± 1.81 |  | 3.77 ± 1.69 | **0.442** | 3.59 ± 1.53 |
| **EO-MSCM-SH vs. MSKM-EL** | 4.11 ± 2.04 | **4.81E-05** | 3.24 ± 1.58 |  | 3.77 ± 1.69 | **3.42E-10** | 2.66 ± 1.03 |
| **EO-MSCM-SH vs. BMSKM-SH** | 4.11 ± 2.04 | **0.944** | 4.05 ± 1.86 |  | 3.77 ± 1.69 | **0.175** | 3.42 ± 1.33 |
| **EO-MSCM-SH vs. BMSKM-EL** | 4.11 ± 2.04 | **9.38E-07** | 3.06 ± 1.30 |  | 3.77 ± 1.69 | **1.05E-11** | 2.57 ± 0.88 |
| **EO-MSCM-EL vs. MSISA-P** | 3.86 ± 1.82 | **0.480** | 3.65 ± 1.74 |  | 3.55 ± 1.73 | **0.663** | 3.34 ± 1.33 |
| **EO-MSCM-EL vs. MSISA-R** | 3.86 ± 1.82 | **1.66E-11** | 2.02 ± 0.52 |  | 3.55 ± 1.73 | **2.55E-05** | 1.79 ± 0.27 |
| **EO-MSCM-EL vs. MSKM-SH** | 3.86 ± 1.82 | **0.421** | 3.97 ± 1.81 |  | 3.55 ± 1.73 | **0.502** | 3.59 ± 1.53 |
| **EO-MSCM-EL vs. MSKM-EL** | 3.86 ± 1.82 | **0.002** | 3.24 ± 1.58 |  | 3.55 ± 1.73 | **2.00E-06** | 2.66 ± 1.03 |
| **EO-MSCM-EL vs. BMSKM-SH** | 3.86 ± 1.82 | **0.240** | 4.05 ± 1.86 |  | 3.55 ± 1.73 | **0.891** | 3.42 ± 1.33 |
| **EO-MSCM-EL vs. BMSKM-EL** | 3.86 ± 1.82 | **9.51E-05** | 3.06 ± 1.30 |  | 3.55 ± 1.73 | **1.27E-07** | 2.57 ± 0.88 |
|  |  |  |  |  |  |  |  |
|  |  |  |  |  |  |  |  |
| ***B. subtilis - L. monocytogenes pairing*** | ***B. subtilis*** | | |  | ***L. monocytogenes*** | | |
| **dist1 vs. dist2** | **dist1 mean (green)** | **Wilcoxon's  2-sided** | **dist2 mean (red)** |  | **dist1 mean (green)** | **Wilcoxon's  2-sided** | **dist2 mean (red)** |
| **EO-MSCM-SH vs. EO-SSCM** | 4.31 ± 1.90 | **2.75E-04** | 3.68 ± 1.88 |  | 5.07 ± 2.00 | **2.69E-09** | 3.62 ± 1.26 |
| **EO-MSCM-SH vs. FD-SSCM** | 4.31 ± 1.90 | **1.51E-14** | 6.73 ± 3.35 |  | 5.07 ± 2.00 | **0.692** | 5.24 ± 2.35 |
| **EO-MSCM-SH vs. QUBIC** | 4.31 ± 1.90 | **5.23E-29** | 2.06 ± 0.50 |  | 5.07 ± 2.00 | **5.51E-28** | 2.36 ± 0.37 |
| **EO-MSCM-SH vs. EO-COAL** | 4.31 ± 1.90 | **1.80E-24** | 2.50 ± 1.30 |  | 5.07 ± 2.00 | **6.16E-07** | 3.74 ± 1.68 |
| **EO-MSCM-SH vs. FD-COAL** | 4.31 ± 1.90 | **1.28E-24** | 2.47 ± 1.12 |  | 5.07 ± 2.00 | **5.19E-09** | 3.51 ± 1.51 |
| **EO-MSCM-EL vs. EO-SSCM** | 3.99 ± 1.82 | **0.050** | 3.68 ± 1.88 |  | 5.08 ± 2.02 | **3.38E-09** | 3.62 ± 1.26 |
| **EO-MSCM-EL vs. FD-SSCM** | 3.99 ± 1.82 | **5.33E-19** | 6.73 ± 3.35 |  | 5.08 ± 2.02 | **0.722** | 5.24 ± 2.35 |
| **EO-MSCM-EL vs. QUBIC** | 3.99 ± 1.82 | **9.61E-25** | 2.06 ± 0.50 |  | 5.08 ± 2.02 | **1.73E-27** | 2.36 ± 0.37 |
| **EO-MSCM-EL vs. EO-COAL** | 3.99 ± 1.82 | **9.45E-20** | 2.50 ± 1.30 |  | 5.08 ± 2.02 | **7.06E-07** | 3.74 ± 1.68 |
| **EO-MSCM-EL vs. FD-COAL** | 3.99 ± 1.82 | **2.81E-19** | 2.47 ± 1.12 |  | 5.08 ± 2.02 | **6.43E-09** | 3.51 ± 1.51 |
| **EO-MSCM-SH vs. MSISA-P** | 4.31 ± 1.90 | **0.069** | 5.06 ± 2.38 |  | 5.07 ± 2.00 | **0.045** | 5.77 ± 1.91 |
| **EO-MSCM-SH vs. MSISA-R** | 4.31 ± 1.90 | **6.55E-13** | 1.99 ± 0.50 |  | 5.07 ± 2.00 | **9.08E-15** | 2.42 ± 0.56 |
| **EO-MSCM-SH vs. MSKM-SH** | 4.31 ± 1.90 | **0.021** | 4.79 ± 1.75 |  | 5.07 ± 2.00 | **0.015** | 5.49 ± 1.73 |
| **EO-MSCM-SH vs. MSKM-EL** | 4.31 ± 1.90 | **3.99E-06** | 3.45 ± 1.88 |  | 5.07 ± 2.00 | **0.001** | 4.35 ± 1.67 |
| **EO-MSCM-SH vs. BMSKM-SH** | 4.31 ± 1.90 | **0.263** | 4.61 ± 2.13 |  | 5.07 ± 2.00 | **0.965** | 5.02 ± 1.71 |
| **EO-MSCM-SH vs. BMSKM-EL** | 4.31 ± 1.90 | **1.77E-08** | 3.19 ± 1.39 |  | 5.07 ± 2.00 | **0.001** | 4.43 ± 1.62 |
| **EO-MSCM-EL vs. MSISA-P** | 3.99 ± 1.82 | **0.010** | 5.06 ± 2.38 |  | 5.08 ± 2.02 | **0.057** | 5.77 ± 1.91 |
| **EO-MSCM-EL vs. MSISA-R** | 3.99 ± 1.82 | **9.88E-12** | 1.99 ± 0.50 |  | 5.08 ± 2.02 | **2.42E-14** | 2.42 ± 0.56 |
| **EO-MSCM-EL vs. MSKM-SH** | 3.99 ± 1.82 | **1.04E-04** | 4.79 ± 1.75 |  | 5.08 ± 2.02 | **0.022** | 5.49 ± 1.73 |
| **EO-MSCM-EL vs. MSKM-EL** | 3.99 ± 1.82 | **0.002** | 3.45 ± 1.88 |  | 5.08 ± 2.02 | **0.001** | 4.35 ± 1.67 |
| **EO-MSCM-EL vs. BMSKM-SH** | 3.99 ± 1.82 | **0.009** | 4.61 ± 2.13 |  | 5.08 ± 2.02 | **0.979** | 5.02 ± 1.71 |
| **EO-MSCM-EL vs. BMSKM-EL** | 3.99 ± 1.82 | **7.87E-05** | 3.19 ± 1.39 |  | 5.08 ± 2.02 | **0.001** | 4.43 ± 1.62 |
|  |  |  |  |  |  |  |  |
|  |  |  |  |  |  |  |  |
| ***B. anthracis - L. monocytogenes pairing*** | ***B. anthracis*** | | |  | ***L. monocytogenes*** | | |
| **dist1 vs. dist2** | **dist1 mean (green)** | **Wilcoxon's  2-sided** | **dist2 mean (red)** |  | **dist1 mean (green)** | **Wilcoxon's  2-sided** | **dist2 mean (red)** |
| **EO-MSCM-SH vs. EO-SSCM** | 3.88 ± 1.80 | **2.58E-07** | 2.92 ± 1.17 |  | 5.02 ± 2.21 | **5.62E-07** | 3.62 ± 1.26 |
| **EO-MSCM-SH vs. FD-SSCM** | 3.88 ± 1.80 | **0.028** | 3.83 ± 2.57 |  | 5.02 ± 2.21 | **0.414** | 5.24 ± 2.35 |
| **EO-MSCM-SH vs. QUBIC** | 3.88 ± 1.80 | **1.13E-27** | 1.77 ± 0.26 |  | 5.02 ± 2.21 | **2.09E-27** | 2.36 ± 0.37 |
| **EO-MSCM-SH vs. EO-COAL** | 3.88 ± 1.80 | **6.71E-16** | 2.36 ± 1.16 |  | 5.02 ± 2.21 | **7.56E-06** | 3.74 ± 1.68 |
| **EO-MSCM-SH vs. FD-COAL** | 3.88 ± 1.80 | **6.01E-17** | 2.32 ± 1.57 |  | 5.02 ± 2.21 | **2.04E-07** | 3.51 ± 1.51 |
| **EO-MSCM-EL vs. EO-SSCM** | 3.80 ± 1.80 | **9.81E-06** | 2.92 ± 1.17 |  | 4.90 ± 2.13 | **1.37E-06** | 3.62 ± 1.26 |
| **EO-MSCM-EL vs. FD-SSCM** | 3.80 ± 1.80 | **0.118** | 3.83 ± 2.57 |  | 4.90 ± 2.13 | **0.203** | 5.24 ± 2.35 |
| **EO-MSCM-EL vs. QUBIC** | 3.80 ± 1.80 | **9.30E-27** | 1.77 ± 0.26 |  | 4.90 ± 2.13 | **2.78E-25** | 2.36 ± 0.37 |
| **EO-MSCM-EL vs. EO-COAL** | 3.80 ± 1.80 | **2.42E-14** | 2.36 ± 1.16 |  | 4.90 ± 2.13 | **2.77E-05** | 3.74 ± 1.68 |
| **EO-MSCM-EL vs. FD-COAL** | 3.80 ± 1.80 | **9.99E-16** | 2.32 ± 1.57 |  | 4.90 ± 2.13 | **9.20E-07** | 3.51 ± 1.51 |
| **EO-MSCM-SH vs. MSKM-SH** | 3.88 ± 1.80 | **0.333** | 3.94 ± 1.46 |  | 5.02 ± 2.21 | **6.34E-04** | 5.61 ± 1.74 |
| **EO-MSCM-SH vs. MSKM-EL** | 3.88 ± 1.80 | **7.27E-11** | 2.61 ± 1.04 |  | 5.02 ± 2.21 | **0.035** | 4.37 ± 1.51 |
| **EO-MSCM-EL vs. MSKM-SH** | 3.80 ± 1.80 | **0.149** | 3.94 ± 1.46 |  | 4.90 ± 2.13 | **1.80E-04** | 5.61 ± 1.74 |
| **EO-MSCM-EL vs. MSKM-EL** | 3.80 ± 1.80 | **3.18E-09** | 2.61 ± 1.04 |  | 4.90 ± 2.13 | **0.067** | 4.37 ± 1.51 |

### Comparisons with Randomized tests

#### Residuals

Table S14: Comparison of bicluster residuals with randomized tests for all methods considered by this study for all pairings of B. subtilis, B. anthracis and L. monocytogenes. A comparison of the residuals of the results from all methods considered for all 3 pairings of the three organisms examined, where each method is compared with its equivalent randomized test. Displayed are the means for each method and/or step compared, as well as the Wilcoxon’s non-parametric rank test (2-sided) comparing their distributions. We direct the reader to 4 for instructions on how to interpret the table. In this case, MSISA and Qubic always reported results worse than random, most likely due their identification of inversely correlated biclusters. Coalesce was worse than random for B. subtilis and L. monocytogenes, but better for B. anthracis.

| ***B. subtilis - B. anthracis pairing*** |  | ***B. subtilis*** |  |  |  | ***B. anthracis*** |  |
| --- | --- | --- | --- | --- | --- | --- | --- |
| **Method** | **derived  mean (green)** | **Wilcoxon's  2-sided** | **shuffle mean (red)** |  | **derived  mean (green)** | **Wilcoxon's  2-sided** | **shuffle mean (red)** |
| **FD-MSCM-SH** | 0.51 ± 0.08 | **3.61E-51** | 0.59 ± 0.04 |  | 0.30 ± 0.09 | **1.15E-99** | 0.78 ± 0.06 |
| **FD-MSCM-EL** | 0.49 ± 0.09 | **7.10E-59** | 0.59 ± 0.03 |  | 0.32 ± 0.09 | **1.19E-99** | 0.78 ± 0.05 |
| **FD-SSCM** | 0.49 ± 0.13 | **6.97E-81** | 0.58 ± 0.03 |  | 0.31 ± 0.12 | **3.06E-202** | 0.79 ± 0.05 |
| **MSKM-SH** | 0.41 ± 0.07 | **7.34E-97** | 0.56 ± 0.03 |  | 0.53 ± 0.12 | **3.71E-86** | 0.76 ± 0.05 |
| **MSKM-EL** | 0.42 ± 0.06 | **1.51E-97** | 0.57 ± 0.02 |  | 0.48 ± 0.11 | **7.31E-98** | 0.80 ± 0.03 |
| **MSISA-P** | 0.98 ± 0.39 | **1.47E-10** | 0.69 ± 0.15 |  | 1.97 ± 0.94 | **3.74E-28** | 0.76 ± 0.08 |
| **MSISA-R** | 1.11 ± 0.41 | **3.92E-19** | 0.66 ± 0.10 |  | 1.58 ± 0.38 | **5.50E-26** | 0.79 ± 0.03 |
| **EO-MSCM-SH** | 0.52 ± 0.09 | **8.04E-35** | 0.58 ± 0.04 |  | 0.50 ± 0.20 | **1.12E-57** | 0.76 ± 0.07 |
| **EO-MSCM-EL** | 0.52 ± 0.10 | **1.25E-47** | 0.58 ± 0.04 |  | 0.49 ± 0.20 | **1.01E-66** | 0.77 ± 0.06 |
| **EO-SSCM** | 0.44 ± 0.20 | **9.78E-61** | 0.58 ± 0.03 |  | 0.23 ± 0.06 | **6.94E-139** | 0.80 ± 0.04 |
| **QUBIC** | 0.87 ± 0.21 | **7.37E-54** | 0.63 ± 0.06 |  | 1.51 ± 0.29 | **2.93E-97** | 0.81 ± 0.02 |
| **EO-COAL** | 0.78 ± 0.23 | **3.90E-27** | 0.65 ± 0.11 |  | 0.58 ± 0.17 | **3.14E-69** | 0.80 ± 0.05 |
| **FD-COAL** | 0.80 ± 0.25 | **2.63E-31** | 0.65 ± 0.10 |  | 0.58 ± 0.17 | **5.66E-66** | 0.80 ± 0.04 |
| **BMSKM-SH** | 0.45 ± 0.07 | **5.36E-88** | 0.56 ± 0.03 |  | 0.38 ± 0.07 | **2.41E-98** | 0.77 ± 0.05 |
| **BMSKM-EL** | 0.45 ± 0.06 | **5.82E-96** | 0.57 ± 0.02 |  | 0.39 ± 0.07 | **1.83E-98** | 0.80 ± 0.02 |
|  |  |  |  |  |  |  |  |
|  |  |  |  |  |  |  |  |
|  |  |  |  |  |  |  |  |
|  |  |  |  |  |  |  |  |
| ***B. subtilis - L. monocytogenes pairing*** |  | ***B. subtilis*** |  |  | ***L. monocytogenes*** | | |
| **Method** | **derived  mean (green)** | **Wilcoxon's  2-sided** | **shuffle mean (red)** |  | **derived  mean (green)** | **Wilcoxon's  2-sided** | **shuffle mean (red)** |
| **FD-MSCM-SH** | 0.52 ± 0.08 | **1.56E-43** | 0.58 ± 0.04 |  | 0.34 ± 0.12 | **7.38E-93** | 0.71 ± 0.08 |
| **FD-MSCM-EL** | 0.50 ± 0.10 | **7.54E-64** | 0.59 ± 0.03 |  | 0.34 ± 0.12 | **2.00E-93** | 0.73 ± 0.08 |
| **FD-SSCM** | 0.49 ± 0.13 | **6.97E-81** | 0.58 ± 0.03 |  | 0.40 ± 0.18 | **6.90E-158** | 0.76 ± 0.08 |
| **MSKM-SH** | 0.40 ± 0.07 | **3.81E-94** | 0.55 ± 0.03 |  | 0.50 ± 0.12 | **4.09E-62** | 0.68 ± 0.09 |
| **MSKM-EL** | 0.42 ± 0.06 | **2.01E-95** | 0.57 ± 0.02 |  | 0.48 ± 0.11 | **9.44E-88** | 0.74 ± 0.07 |
| **MSISA-P** | 0.87 ± 0.34 | **5.97E-05** | 0.68 ± 0.20 |  | 1.59 ± 0.52 | **4.72E-20** | 0.71 ± 0.32 |
| **MSISA-R** | 1.11 ± 0.42 | **2.40E-17** | 0.66 ± 0.10 |  | 1.31 ± 0.34 | **2.48E-21** | 0.79 ± 0.09 |
| **EO-MSCM-SH** | 0.52 ± 0.08 | **4.19E-33** | 0.57 ± 0.04 |  | 0.49 ± 0.17 | **5.28E-50** | 0.70 ± 0.10 |
| **EO-MSCM-EL** | 0.50 ± 0.09 | **4.34E-52** | 0.58 ± 0.04 |  | 0.48 ± 0.17 | **6.06E-54** | 0.70 ± 0.10 |
| **EO-SSCM** | 0.44 ± 0.20 | **9.78E-61** | 0.58 ± 0.03 |  | 0.29 ± 0.10 | **7.26E-56** | 0.79 ± 0.05 |
| **QUBIC** | 0.87 ± 0.21 | **7.37E-54** | 0.63 ± 0.06 |  | 1.81 ± 0.85 | **2.51E-24** | 0.82 ± 0.03 |
| **EO-COAL** | 0.78 ± 0.23 | **3.90E-27** | 0.65 ± 0.11 |  | 1.63 ± 3.07 | **7.52E-18** | 0.79 ± 0.08 |
| **FD-COAL** | 0.80 ± 0.25 | **2.63E-31** | 0.65 ± 0.10 |  | 1.70 ± 3.24 | **2.90E-19** | 0.80 ± 0.07 |
| **BMSKM-SH** | 0.43 ± 0.07 | **3.02E-85** | 0.55 ± 0.03 |  | 0.42 ± 0.09 | **1.73E-87** | 0.68 ± 0.09 |
| **BMSKM-EL** | 0.44 ± 0.06 | **9.03E-96** | 0.57 ± 0.02 |  | 0.42 ± 0.09 | **2.28E-95** | 0.74 ± 0.06 |
|  |  |  |  |  |  |  |  |
|  |  |  |  |  |  |  |  |
| ***B. anthracis - L. monocytogenes pairing*** |  | ***B. anthracis*** |  |  | ***L. monocytogenes*** | | |
| **Method** | **derived  mean (green)** | **Wilcoxon's  2-sided** | **shuffle mean (red)** |  | **derived  mean (green)** | **Wilcoxon's  2-sided** | **shuffle mean (red)** |
| **FD-MSCM-SH** | 0.33 ± 0.10 | **2.75E-97** | 0.73 ± 0.07 |  | 0.36 ± 0.14 | **4.59E-88** | 0.73 ± 0.08 |
| **FD-MSCM-EL** | 0.36 ± 0.11 | **7.87E-97** | 0.75 ± 0.07 |  | 0.36 ± 0.13 | **9.49E-91** | 0.75 ± 0.08 |
| **FD-SSCM** | 0.31 ± 0.12 | **1.02E-192** | 0.79 ± 0.05 |  | 0.40 ± 0.18 | **6.90E-158** | 0.76 ± 0.08 |
| **MSKM-SH** | 0.40 ± 0.08 | **2.42E-94** | 0.70 ± 0.07 |  | 0.43 ± 0.08 | **3.22E-89** | 0.70 ± 0.08 |
| **MSKM-EL** | 0.39 ± 0.07 | **1.68E-96** | 0.78 ± 0.04 |  | 0.43 ± 0.08 | **5.25E-95** | 0.75 ± 0.06 |
| **EO-MSCM-SH** | 0.52 ± 0.17 | **1.08E-42** | 0.71 ± 0.09 |  | 0.50 ± 0.18 | **1.35E-41** | 0.71 ± 0.10 |
| **EO-MSCM-EL** | 0.50 ± 0.17 | **8.12E-50** | 0.72 ± 0.09 |  | 0.50 ± 0.19 | **7.83E-45** | 0.72 ± 0.10 |
| **EO-SSCM** | 0.23 ± 0.06 | **6.94E-139** | 0.80 ± 0.04 |  | 0.29 ± 0.10 | **7.26E-56** | 0.79 ± 0.05 |
| **QUBIC** | 1.51 ± 0.29 | **2.93E-97** | 0.81 ± 0.02 |  | 1.81 ± 0.85 | **2.51E-24** | 0.82 ± 0.03 |
| **EO-COAL** | 0.58 ± 0.17 | **3.14E-69** | 0.80 ± 0.05 |  | 1.63 ± 3.07 | **7.52E-18** | 0.79 ± 0.08 |
| **FD-COAL** | 0.58 ± 0.17 | **5.66E-66** | 0.80 ± 0.04 |  | 1.70 ± 3.24 | **2.90E-19** | 0.80 ± 0.07 |

#### Mean correlations

Table S15: Comparison of bicluster mean correlations with randomized tests for all methods considered by this study for all pairings of B. subtilis, B. anthracis and L. monocytogenes. A comparison of the mean correlations of the results from all methods considered for all 3 pairings of the three organisms examined, where each method is compared with its equivalent randomized test. Displayed are the means for each method and/or step compared, as well as the Wilcoxon’s non-parametric rank test (2-sided) comparing their distributions. We direct the reader to 4 for instructions on how to interpret the table. In nearly all comparisons, the method was significantly better than random. The sole exception were the biclusters produces by MSISA-R for L. monocytogenes from the pairing of B. subtilis and L. monocytogenes.

| ***B. subtilis - B. anthracis pairing*** |  | ***B. subtilis*** |  |  | ***B. anthracis*** | | |
| --- | --- | --- | --- | --- | --- | --- | --- |
| **Method** | **derived  mean (green)** | **Wilcoxon's  2-sided** | **shuffle mean (red)** |  | **derived  mean (green)** | **Wilcoxon's  2-sided** | **shuffle mean (red)** |
| **FD-MSCM-SH** | 0.59 ± 0.11 | **3.99E-99** | 0.27 ± 0.04 |  | 0.85 ± 0.09 | **1.28E-99** | 0.40 ± 0.06 |
| **FD-MSCM-EL** | 0.61 ± 0.11 | **1.36E-99** | 0.26 ± 0.04 |  | 0.84 ± 0.09 | **1.10E-99** | 0.39 ± 0.05 |
| **FD-SSCM** | 0.56 ± 0.14 | **1.49E-190** | 0.25 ± 0.04 |  | 0.82 ± 0.15 | **1.36E-191** | 0.37 ± 0.06 |
| **MSKM-SH** | 0.58 ± 0.11 | **4.49E-98** | 0.25 ± 0.04 |  | 0.52 ± 0.14 | **1.18E-39** | 0.39 ± 0.05 |
| **MSKM-EL** | 0.56 ± 0.11 | **3.37E-98** | 0.25 ± 0.03 |  | 0.58 ± 0.15 | **9.14E-65** | 0.37 ± 0.03 |
| **MSISA-P** | 0.60 ± 0.14 | **2.43E-12** | 0.44 ± 0.10 |  | 0.56 ± 0.07 | **1.12E-07** | 0.49 ± 0.08 |
| **MSISA-R** | 0.55 ± 0.13 | **2.61E-10** | 0.42 ± 0.08 |  | 0.51 ± 0.03 | **6.32E-09** | 0.47 ± 0.04 |
| **EO-MSCM-SH** | 0.52 ± 0.12 | **2.44E-90** | 0.27 ± 0.05 |  | 0.69 ± 0.17 | **1.29E-73** | 0.40 ± 0.07 |
| **EO-MSCM-EL** | 0.54 ± 0.12 | **1.41E-93** | 0.26 ± 0.05 |  | 0.69 ± 0.19 | **1.80E-69** | 0.40 ± 0.06 |
| **EO-SSCM** | 0.70 ± 0.11 | **1.05E-106** | 0.25 ± 0.04 |  | 0.91 ± 0.05 | **6.91E-139** | 0.37 ± 0.04 |
| **QUBIC** | 0.36 ± 0.19 | **2.58E-10** | 0.32 ± 0.04 |  | 0.49 ± 0.05 | **8.79E-67** | 0.41 ± 0.03 |
| **EO-COAL** | 0.58 ± 0.14 | **1.28E-115** | 0.37 ± 0.10 |  | 0.64 ± 0.13 | **6.88E-84** | 0.42 ± 0.06 |
| **FD-COAL** | 0.59 ± 0.15 | **1.73E-99** | 0.38 ± 0.10 |  | 0.62 ± 0.13 | **3.19E-74** | 0.41 ± 0.05 |
| **BMSKM-SH** | 0.49 ± 0.13 | **3.89E-92** | 0.25 ± 0.04 |  | 0.72 ± 0.10 | **1.12E-97** | 0.39 ± 0.05 |
| **BMSKM-EL** | 0.50 ± 0.12 | **1.89E-97** | 0.25 ± 0.03 |  | 0.71 ± 0.10 | **2.07E-98** | 0.37 ± 0.03 |
|  |  |  |  |  |  |  |  |
|  |  |  |  |  |  |  |  |
| ***B. subtilis - L. monocytogenes pairing*** |  | ***B. subtilis*** |  |  | ***L. monocytogenes*** | | |
| **Method** | **derived  mean (green)** | **Wilcoxon's  2-sided** | **shuffle mean (red)** |  | **derived  mean (green)** | **Wilcoxon's  2-sided** | **shuffle mean (red)** |
| **FD-MSCM-SH** | 0.59 ± 0.11 | **1.07E-95** | 0.27 ± 0.05 |  | 0.80 ± 0.13 | **1.06E-89** | 0.44 ± 0.09 |
| **FD-MSCM-EL** | 0.61 ± 0.10 | **2.81E-97** | 0.26 ± 0.04 |  | 0.81 ± 0.11 | **1.05E-93** | 0.43 ± 0.08 |
| **FD-SSCM** | 0.56 ± 0.14 | **1.24E-190** | 0.25 ± 0.04 |  | 0.71 ± 0.20 | **2.05E-106** | 0.42 ± 0.10 |
| **MSKM-SH** | 0.59 ± 0.11 | **5.31E-95** | 0.26 ± 0.05 |  | 0.51 ± 0.17 | **4.57E-11** | 0.42 ± 0.10 |
| **MSKM-EL** | 0.56 ± 0.11 | **3.20E-96** | 0.25 ± 0.03 |  | 0.55 ± 0.16 | **4.76E-27** | 0.42 ± 0.07 |
| **MSISA-P** | 0.60 ± 0.20 | **4.42E-06** | 0.44 ± 0.14 |  | 0.47 ± 0.23 | **0.010** | 0.55 ± 0.23 |
| **MSISA-R** | 0.55 ± 0.12 | **3.47E-10** | 0.42 ± 0.08 |  | 0.50 ± 0.27 | **0.009** | 0.51 ± 0.17 |
| **EO-MSCM-SH** | 0.52 ± 0.13 | **8.75E-85** | 0.27 ± 0.06 |  | 0.64 ± 0.18 | **1.24E-41** | 0.44 ± 0.11 |
| **EO-MSCM-EL** | 0.54 ± 0.12 | **2.58E-91** | 0.27 ± 0.06 |  | 0.64 ± 0.18 | **1.52E-43** | 0.43 ± 0.10 |
| **EO-SSCM** | 0.70 ± 0.11 | **1.04E-106** | 0.25 ± 0.04 |  | 0.86 ± 0.08 | **6.49E-56** | 0.43 ± 0.07 |
| **QUBIC** | 0.36 ± 0.19 | **2.58E-10** | 0.32 ± 0.04 |  | 0.45 ± 0.27 | **4.59E-23** | 0.45 ± 0.03 |
| **EO-COAL** | 0.58 ± 0.14 | **1.28E-115** | 0.37 ± 0.10 |  | 0.81 ± 0.13 | **2.05E-39** | 0.51 ± 0.10 |
| **FD-COAL** | 0.59 ± 0.15 | **1.73E-99** | 0.38 ± 0.10 |  | 0.80 ± 0.12 | **3.70E-41** | 0.50 ± 0.08 |
| **BMSKM-SH** | 0.52 ± 0.14 | **2.18E-85** | 0.26 ± 0.05 |  | 0.63 ± 0.15 | **4.15E-54** | 0.42 ± 0.10 |
| **BMSKM-EL** | 0.53 ± 0.12 | **9.89E-96** | 0.25 ± 0.03 |  | 0.64 ± 0.14 | **3.92E-66** | 0.42 ± 0.07 |
|  |  |  |  |  |  |  |  |
|  |  |  |  |  |  |  |  |
| ***B. anthracis - L. monocytogenes pairing*** | ***B. anthracis*** | | |  | ***L. monocytogenes*** | | |
| **Method** | **derived  mean (green)** | **Wilcoxon's  2-sided** | **shuffle mean (red)** |  | **derived  mean (green)** | **Wilcoxon's  2-sided** | **shuffle mean (red)** |
| **FD-MSCM-SH** | 0.82 ± 0.11 | **1.43E-97** | 0.40 ± 0.07 |  | 0.77 ± 0.14 | **2.14E-84** | 0.44 ± 0.09 |
| **FD-MSCM-EL** | 0.80 ± 0.11 | **9.72E-98** | 0.39 ± 0.06 |  | 0.78 ± 0.13 | **5.76E-91** | 0.43 ± 0.08 |
| **FD-SSCM** | 0.82 ± 0.15 | **4.67E-182** | 0.37 ± 0.05 |  | 0.71 ± 0.20 | **2.23E-106** | 0.42 ± 0.10 |
| **MSKM-SH** | 0.69 ± 0.12 | **2.60E-88** | 0.39 ± 0.07 |  | 0.60 ± 0.14 | **2.13E-51** | 0.42 ± 0.10 |
| **MSKM-EL** | 0.70 ± 0.10 | **2.16E-95** | 0.37 ± 0.03 |  | 0.63 ± 0.13 | **2.53E-65** | 0.42 ± 0.07 |
| **EO-MSCM-SH** | 0.63 ± 0.16 | **3.74E-56** | 0.40 ± 0.08 |  | 0.63 ± 0.19 | **7.13E-36** | 0.43 ± 0.11 |
| **EO-MSCM-EL** | 0.63 ± 0.17 | **9.25E-57** | 0.40 ± 0.08 |  | 0.63 ± 0.19 | **7.82E-36** | 0.43 ± 0.10 |
| **EO-SSCM** | 0.91 ± 0.05 | **6.93E-139** | 0.37 ± 0.04 |  | 0.86 ± 0.08 | **6.28E-56** | 0.43 ± 0.07 |
| **QUBIC** | 0.49 ± 0.05 | **8.79E-67** | 0.41 ± 0.03 |  | 0.45 ± 0.27 | **4.59E-23** | 0.45 ± 0.03 |
| **EO-COAL** | 0.64 ± 0.13 | **6.88E-84** | 0.42 ± 0.06 |  | 0.81 ± 0.13 | **2.05E-39** | 0.51 ± 0.10 |
| **FD-COAL** | 0.62 ± 0.13 | **3.19E-74** | 0.41 ± 0.05 |  | 0.80 ± 0.12 | **3.70E-41** | 0.50 ± 0.08 |

## Additional GO term and KEGG pathway enrichment figures

GO term enrichments were initially introduced by Draghici et al [92] as a measure of the functional coherence of a set of genes. Effectively, GO term enrichments represent the probability, by chance, that a set of genes share the same functional annotation, which is approximated using the hypergeomentric distribution:

$$P\left(b_{k}|G,T\right)=\frac{\left(\begin{matrix} \left|T\right| \\ \left|b_{k}\cap T\right| \end{matrix}\right)\left(\begin{matrix} \left|G\right|-\left|T\right| \\ \left|b_{k}\right|-\left|b_{k}\cap T\right| \end{matrix}\right)}{\left(\begin{matrix} \left|G\right| \\ \left|b_{k}\right| \end{matrix}\right)}$$

where *b_k_* is the set of genes in bicluster *k*; *G* is the set of genes in the genome ; and *T* is the set of genes having a particular GO term annotation. Similarly, KEGG pathway enrichments were approximated with a hypergeometric distribution, where *T* was instead the set of genes associated with a given KEGG pathway.

For all the pairings between *B. subtilis, B. anthracis* and *L. monocytogenes*, there is a consistent increase from the shared to elaboration steps of all the multi-species methods, with the percentage of FD-MSCM-EL biclusters with significant GO term enrichments consistently greater than the SSCM results. Similar behavior is observed with the KEGG pathway enrichments. The higher percentages reported for the MSISA and Qubic methods are a reflection of the high redundancy of the biclusters identified by them.

Figure S34: Comparison of the fraction of biclusters with significant GO and KEGG annotation enrichments from all methods considered by this study for the B. subtilis – B. anthracis pairing. (A) GO Terms. Percentage of biclusters with enriched GO terms. (B) KEGG Pathways. Percentage of biclusters with enriched KEGG pathways. Explanations of the method name abbreviations can be found in Table S1.


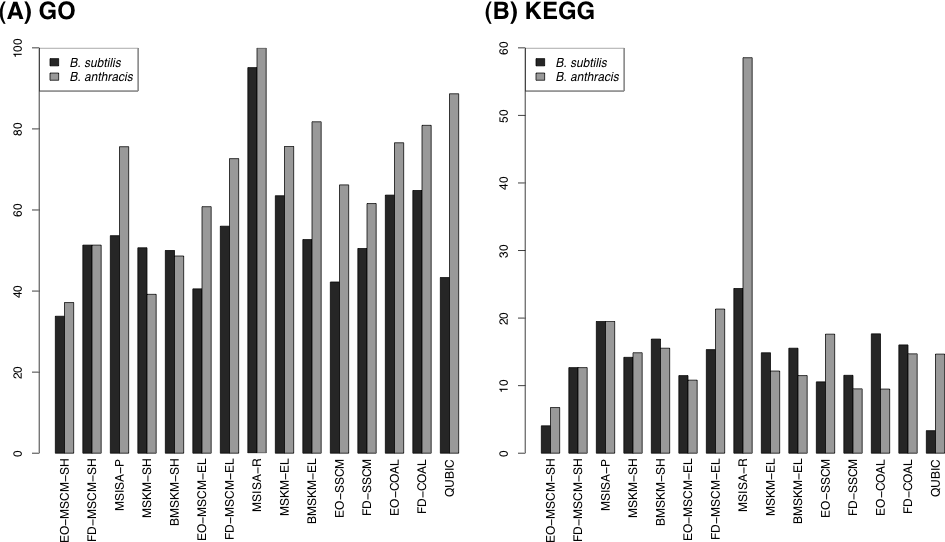


Figure S35: Comparison of the fraction of biclusters with significant GO and KEGG annotation enrichments for the multi-species cMonkey, multi-species k-means and single-species cMonkey methods for the B. subtilis – L. monocytogenes pairing. (A) GO Terms. Percentage of biclusters with enriched GO terms. (B) KEGG Pathways. Percentage of biclusters with enriched KEGG pathways. Explanations of the method name abbreviations can be found in Table S1.


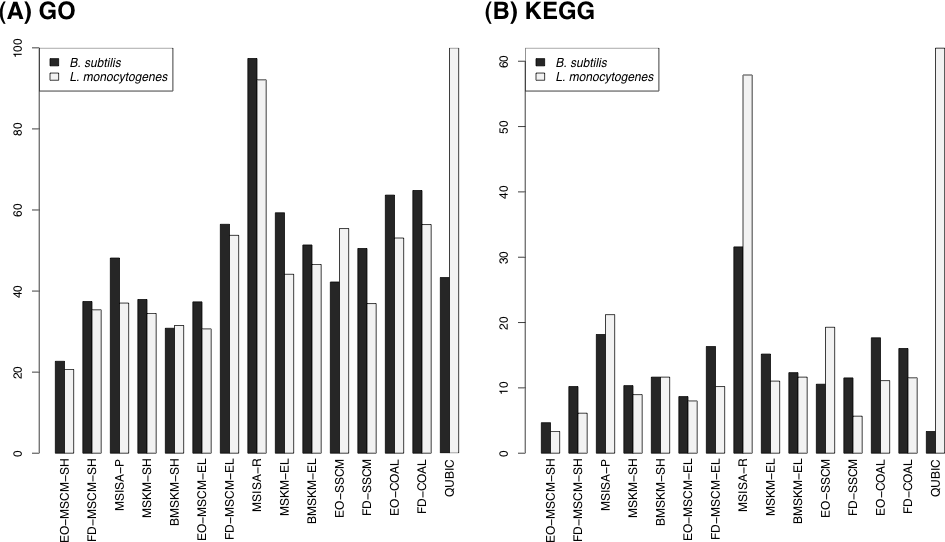


Figure S36: Comparison of the fraction of biclusters with significant GO and KEGG annotation enrichments for the multi-species cMonkey, multi-species k-means and single-species cMonkey methods for the B. anthracis – L. monocytogenes pairing. (A) GO Terms. Percentage of biclusters with enriched GO terms. (B) KEGG Pathways. Percentage of biclusters with enriched KEGG pathways. Explanations of the method name abbreviations can be found in Table S1.


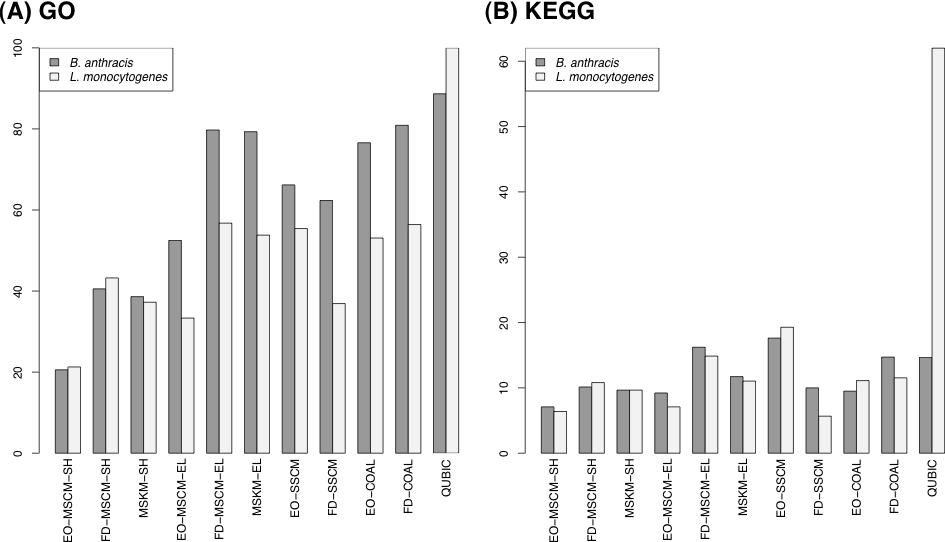


## Putative σ^E^ binding site in the ctaC operon upstream sequence

Figure S37: Putative σ^E^ binding site in the regulatory upstream sequence of the ctaC operon. Four genes from Bicluster 84, ctaC ctaD ctaE ctaF, encode the subunits for cytochrome C oxidase. These genes have not been shown to be regulated by σ^E^, however, the region upstream of ctaC contains a possible σ^E^ binding site. (A) Consensus binding sequence for σ^E^ (Sierro, et al., 2008, Eichenberger et al. 2004). (B) Portion of the intercistronic region from B. subtilis between ctaB and ctaC showing the respective -35 and -10 regions of the potential σ^E^ binging site (blue, bold, uppercase letters). Also indicated are the consensus sequences for the two regions, the putative ribosomal binding site (underlined, uppercase letters), and the translation start site (green, uppercase letters).


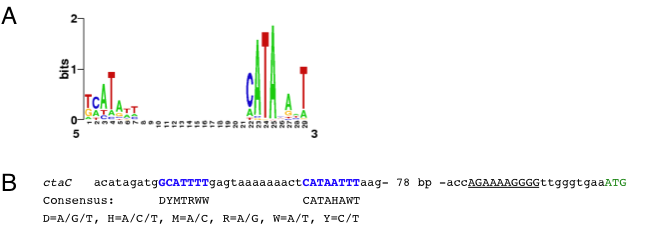


## Flagellar motility regulators and missing genes

Table S16: B. subtilis flagellar assembly genes that are missing in B. anthracis, and their associated function. The genes in the table are present in the B. subtilis flagellar assembly pathway as indicated by KEGG, but missing in B. anthracis.

| **Gene** | **Function** |
| --- | --- |
| *flgM* | anti-σ^D^ factor |
| *fliJ* | Part of the type III secretion chaperone-usher complex |
| *fliK* | hook length regulator |
| *fliO* | Part of the Type III secretion apparatus |
| *fliT* | Chaperone |
| *sigD* | Sigma factor responsible for the expression of motility and chemotaxis genes |

The major regulators of motility in both *B. subtilis* and *L. monocytogenes* were also examined, and their functions are shown in Table S17.

Table S17: Major Regulators of Motility in B. subtilis and L. monocytogenes

| **Regulator** | **Organism** | **Function** | **Reference** |
| --- | --- | --- | --- |
| *sigD* | *B. subtilis* | Sigma factor responsible for the expression of motility and chemotaxis genes | Marques-Magana and Chamberlin, 1994 [93] |
| *mogR* | *L. monocytogenes* | Transcriptional repressor of flagellar genes when at temperatures > 37°C | Grundling *et al*., 2004 [52] |
| *degU* | *L. monocytogenes* | Response regulator which controls temperature-responsive expression of *gmaR* | Shen *et al*., 2006 [94] |
| *gmaR* | *L. monocytogenes* | Antirepressor of MogR | Shen *et al*., 2006 [94] |

A BLAST search was performed to see if these regulators are present in *B. cereus* and *B. anthracis* (strains Ames and Sterne)*.* These searches found that *B. subtilis* had no similar match to MogR but did have a fairly strong match for GmaR and does have DegU. *B. cereus* and *B. anthracis* str. Ames had similarly strong matches to *L. monocytogenes* DegU, MogR and GmaR. *B. anthracis* str. Sterne had matches to DegU and MogR that were similar to *B. cereus* and *B. anthracis* Ames but had two very strong matches for GmaR, one to the N-terminus and the other to the C-terminus. In contrast, *L. monocytogenes, B. cereus,* and *B. anthracis* (Ames and Sterne strains) do not have *sigD*.

These findings indicate that the presence or absence of the essential *B.* *subtilis* transcriptional regulators of motility genes does not preclude motility in *B.* *anthracis*, as portions of *B. anthracis* motility are likely to differ from *B. subtilis,* and are likely to resemble *L. monocytogenes and B. cereus*.

Table S18: As shown in the table, the genetic composition of the B. anthracis strains Sterne, A2012 and CDC 684 are almost identical to the motile species B. cereus, B. thuringiensis, B. weihenstephanensis and L. monocytogenes. In contrast, the B. anthracis strains Ames, Ames 0581 and A0248 are lacking multiple genes present in the other motile organisms.

|  | *B.subtilis* | *B. amyloliquefaciens* | *B. clausii* | *B. anthracis* (Sterne, A2012, CDC 684) | *B. anthracis* (Ames, Ames 0581, A0248) |
| --- | --- | --- | --- | --- | --- |
|  | *B. halodurans* | *B. pumilus* |  | *B. cereus* (all) |  |
|  | *B. licheniformis* (all) |  |  | *B. thuringiensis* |  |
|  |  |  |  | *B. weihenstephanensis* |  |
| **Genes** |  |  |  | *L. monocytogenes* |  |
| *flgL* | X | X | X | X |  |
| *flgM* | X | X | X |  |  |
| *fliF* | X | X | X | X |  |
| *fliJ* | X | X | X |  |  |
| *fliK* | X | * |  |  |  |
| *fliM* | X | X | X | X |  |
| *fliO* | X | X | X |  |  |
| *FliT* | X | X |  |  |  |
| *cheC* | X | X | X |  |  |
| *cheD* | X | X | X |  |  |
| *cheV* | X | X | X | X’ |  |
| *Chew* | X | X | X | X’’ |  |

X gene is present in the KEGG flagellar assembly pathway

X’ gene is not present in B. anthracis Sterne or A2012

X’’ gene is present in B. anthracis Sterne and A2012 but not the other organisms in the column

* gene is not present in KEGG but is recognized by NCBI

Figure S38: B. anthracis Sterne frameshift mutations. B. anthracis Sterne contains frameshift mutations in five motility genes (motB, flgL, fliF, fliM, and cheV). The frameshift mutation in motB was discovered in B. anthracis Sterne by first performing protein BLAST searches using B. cereus ATCC 14579 MotB as the query sequence. Investigation of the B. anthracis Sterne gene sequence revealed a single base deletion (red delta, Δ) that causes a frameshift mutation resulting in a stop codon (red, uppercase letters) thus truncating the MotB protein. The other B. anthracis frameshift mutations (flgL, fliF, fliM, cheV) were discovered using the same method.


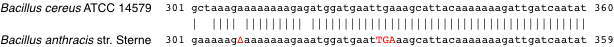


# Full descriptions of highlighted biclusters

## Gene lists for B. subtilis, B. anthracis Sterne sporulation clusters 32, 82, and 84.

### B. subtilis - B. anthracis cluster 32

Figure S39: B. subtilis cluster 32 image (post-elaboration)

**
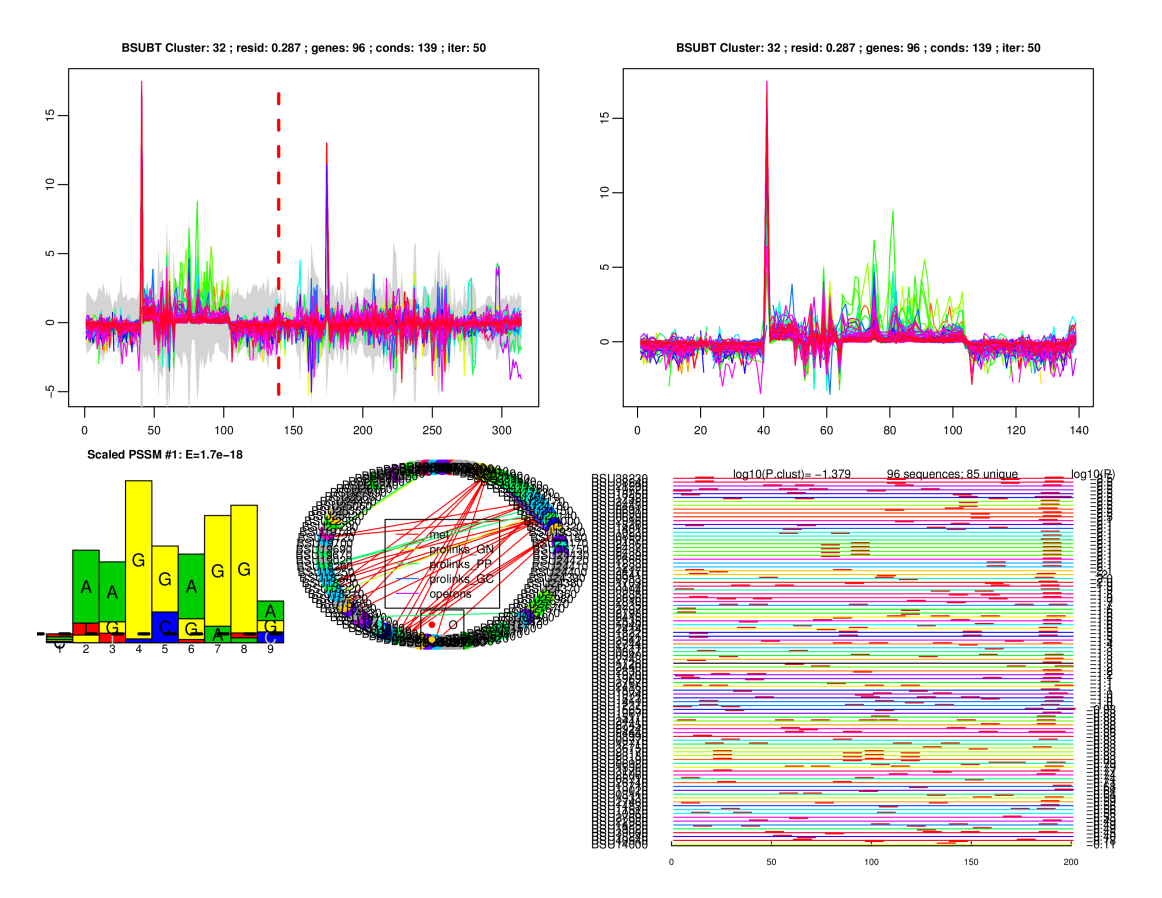
**

| ***B. subtilis* cluster 32 core genes** |  |  |
| --- | --- | --- |
| **Locus** | **Name** | **Function** |
| BSU06890 | *cotJA* | polypeptide composition of the spore coat; required for the assembly of CotJC |
| BSU06900 | *cotJB* | polypeptide composition of the spore coat |
| BSU06910 | *cotJC* | polypeptide composition of the spore coat |
| BSU23190 | *dacB* | D-alanyl-D-alanine carboxypeptidase (penicillin-binding protein 5*) |
| BSU28380 | *gerM* | germination (cortex hydrolysis) and sporulation (stage II, multiple polar septa) |
| BSU27440 | *glnH* | glutamine ABC transporter (glutamine-binding protein) |
| BSU27450 | *glnM* | glutamine ABC transporter (integral membrane protein) |
| BSU27460 | *glnP* | glutamine ABC transporter (integral membrane protein) |
| BSU27430 | *glnQ* | glutamine ABC transporter (ATP-binding protein) |
| BSU15320 | *sigE* | sporulation sigma factor SigE |
| BSU23180 | *spmA* | spore maturation protein |
| BSU23170 | *spmB* | spore maturation protein |
| BSU24430 | *spoIIIAA* | mutants block sporulation after engulfment (stage III sporulation) |
| BSU24420 | *spoIIIAB* | stage III sporulation protein SpoAB |
| BSU24410 | *spoIIIAC* | mutants block sporulation after engulfment (stage III sporulation) |
| BSU24400 | *spoIIIAD* | mutants block sporulation after engulfment (stage III sporulation) |
| BSU24390 | *spoIIIAE* | mutants block sporulation after engulfment (stage III sporulation) |
| BSU24380 | *spoIIIAF* | mutants block sporulation after engulfment (stage III sporulation) |
| BSU24370 | *spoIIIAG* | mutants block sporulation after engulfment (stage III sporulation) |
| BSU24360 | *spoIIIAH* | mutants block sporulation after engulfment (stage III sporulation) |
| BSU27980 | *spoIVFA* | inhibition of SpoIVFB (negative regulation) and hypothesised to stabilize the thermolabile SpoIVFB product (positive regulation) (stage IV sporulation) |
| BSU27970 | *spoIVFB* | membrane metalloprotease |
| BSU27670 | *spoVB* | involved in spore cortex synthesis (stage V sporulation) |
| BSU01570 | *ybaN* | hypothetical protein |
| BSU09940 | *yhaL* | hypothetical protein |
| BSU11510 | *yjbE* | hypothetical protein |
| BSU14110 | *ykuK* | hypothetical protein |
| BSU13710 | *ykvI* | hypothetical protein |
| BSU15030 | *ylbJ* | hypothetical protein |
| BSU15650 | *yloB* | hypothetical protein |
| BSU25350 | *yqfD* | hypothetical protein |
| BSU25060 | *yqfZ* | hypothetical protein |
| BSU24440 | *yqhV* | hypothetical protein |
| BSU27690 | *yrzE* | hypothetical protein |
| BSU28100 | *ysxE* | hypothetical protein |
| BSU29240 | *ytrI* | hypothetical protein |
| BSU28960 | *ytxC* | hypothetical protein |
| BSU32350 | *yunB* | hypothetical protein |
|  |  |  |
| ***B. subtilis* cluster 32 elaboration genes** |  |  |
| **Locus** | **Name** | **Function** |
| BSU17260 | *aprX* | alkaline serine protease |
| BSU17030 | *cotE* | morphogenic protein |
| BSU26740 | *cypA* | cytochrome P450-like enzyme |
| BSU12370 | *exuR* | transcriptional regulator (LacI family) |
| BSU19690 | *kamA* | lysine 2,3-aminomutase |
| BSU36410 | *mbl* | MreB-like protein |
| BSU24170 | *mmgA* | acetyl-CoA acetyltransferase |
| BSU24160 | *mmgB* | 3-hydroxybutyryl-CoA dehydrogenase |
| BSU24150 | *mmgC* | acyl-CoA dehydrogenase |
| BSU24140 | *mmgD* | citrate synthase 3 |
| BSU14000 | *patA* | aminotransferase A |
| BSU38990 | *scoA* | succinyl CoA:3-oxoacid CoA-transferase (subunit A) |
| BSU19330 | *sodF* | superoxide dismutase |
| BSU36750 | *spoIID* | required for complete dissolution of the asymmetric septum (stage II sporulation) |
| BSU15170 | *spoVD* | penicillin-binding protein |
| BSU09400 | *spoVR* | involved in spore cortex synthesis (stage V sporulation) |
| BSU37830 | *spsJ* | spore coat polysaccharide synthesis |
| BSU19320 | *sqhC* | squalene-hopene cyclase |
| BSU12350 | *yjmF* | D-mannonate oxidoreductase |
| BSU14830 | *ylaM* | glutaminase |
| BSU18220 | *yngF* | enoyl-CoA hydratase |
| BSU18230 | *yngG* | hydroxymethylglutaryl-CoA lyase |
| BSU18240 | *yngH* | acetyl-CoA carboxylase biotin carboxylase subunit |
| BSU18250 | *yngI* | acyl-CoA synthetase |
| BSU12710 | *xkdR* | hypothetical protein |
| BSU00160 | *yaaH* | hypothetical protein |
| BSU03110 | *ycgH* | hypothetical protein |
| BSU03670 | *yclF* | hypothetical protein |
| BSU05710 | *ydhD* | hypothetical protein |
| BSU06920 | *yesJ* | hypothetical protein |
| BSU09830 | *yhaX* | hypothetical protein |
| BSU08980 | *yhbH* | hypothetical protein |
| BSU09770 | *yheD* | hypothetical protein |
| BSU10230 | *yhfH* | hypothetical protein |
| BSU10400 | *yhxC* | hypothetical protein |
| BSU10960 | *yitE* | hypothetical protein |
| BSU12110 | *yjfA* | hypothetical protein |
| BSU12320 | *yjmC* | hypothetical protein |
| BSU12330 | *yjmD* | hypothetical protein |
| BSU14250 | *yknT* | hypothetical protein |
| BSU14810 | *ylaK* | hypothetical protein |
| BSU17320 | *ymaF* | hypothetical protein |
| BSU18210 | *yngE* | hypothetical protein |
| BSU18260 | *yngJ* | hypothetical protein |
| BSU19020 | *yobN* | hypothetical protein |
| BSU19670 | *yodN* | hypothetical protein |
| BSU19700 | *yodP* | hypothetical protein |
| BSU19720 | *yodR* | hypothetical protein |
| BSU19740 | *yodT* | hypothetical protein |
| BSU21290 | *yomN* | hypothetical protein |
| BSU22980 | *ypbG* | hypothetical protein |
| BSU25420 | *yqeW* | hypothetical protein |
| BSU26660 | *yrdN* | hypothetical protein |
| BSU29160 | *ytvI* | hypothetical protein |
| BSU31740 | *yuxH* | hypothetical protein |
| BSU31730 | *yuzC* | hypothetical protein |
| BSU38240 | *ywcA* | hypothetical protein |
| BSU38230 | *ywcB* | hypothetical protein |
| BSU39000 | *yxjC* | hypothetical protein |
| BSU40940 | *yyaD* | hypothetical protein |

Figure S40: B. anthracis cluster 32 image (post-elaboration)

**
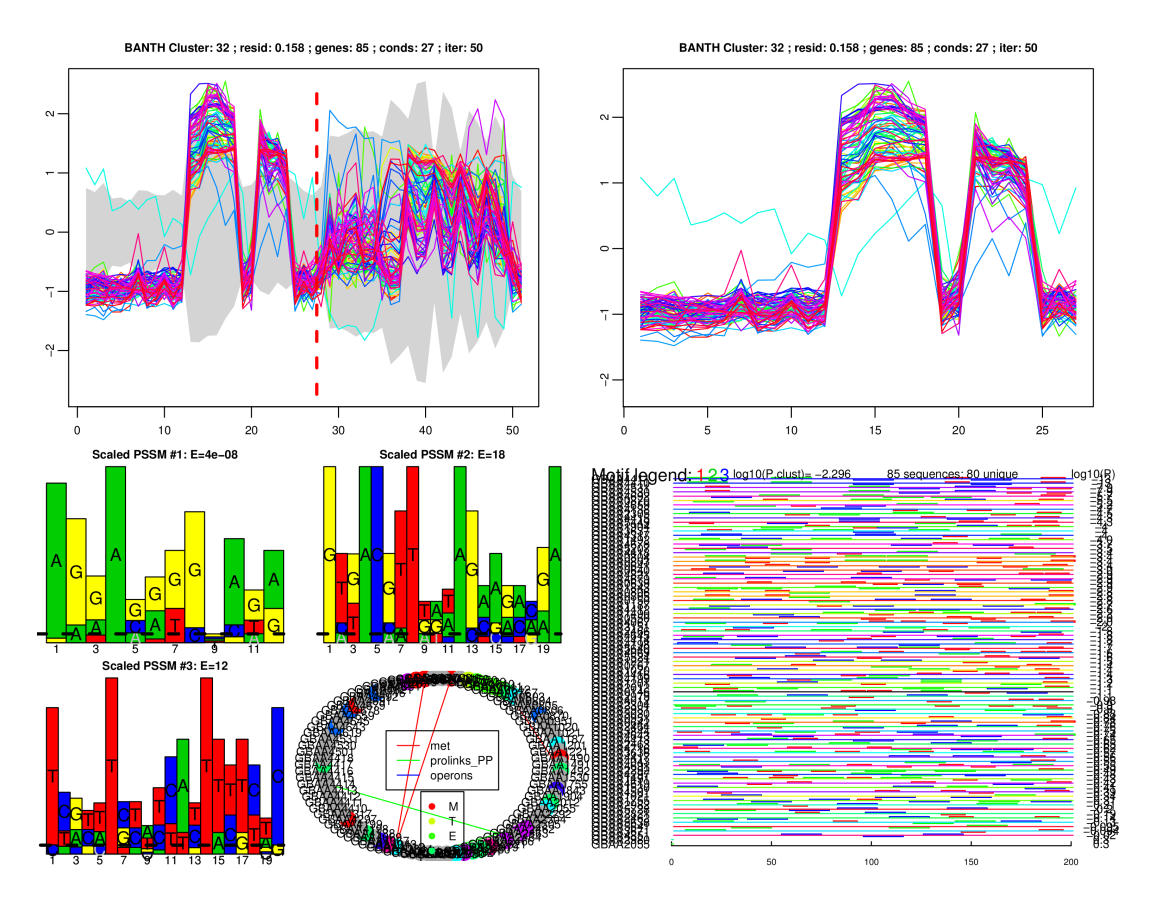
**

| ***B. anthracis* cluster 32 core genes** |  |  |
| --- | --- | --- |
| **Locus** | **Name** | **Function** |
| GBAA0805 | *cotJA* | cotja protein |
| GBAA0804 | *cotJB* | cotjb protein |
| GBAA0803 | *cotJC* | cotjc protein |
| GBAA4716 | *gerM* | germination protein gerM |
| GBAA4043 | *sigE* | sporulation sigma factor SigE |
| GBAA1491 | *spmA* | spore maturation protein a |
| GBAA4417 | *spoIIIAA* | stage iii sporulation protein aa |
| GBAA4416 | *spoIIIAB* | stage III sporulation protein SpoAB |
| GBAA4415 | *spoIIIAC* | stage iii sporulation protein ac |
| GBAA4414 | *spoIIIAD* | stage iii sporulation protein ad |
| GBAA4413 | *spoIIIAE* | stage iii sporulation protein ae |
| GBAA4412 | *spoIIIAF* | stage iii sporulation protein af |
| GBAA4411 | *spoIIIAG* | stage iii sporulation protein ag |
| GBAA4679 | *spoIVFA* | stage iv sporulation protein fa |
| GBAA4678 | *spoIVFB* | stage iv sporulation protein fb |
| GBAA4643 | *spoVB* | stage v sporulation protein b |
| GBAA0640 | *-* | amino acid abc transporter, amino acid-binding protein |
| GBAA0639 | *-* | amino acid abc transporter, atp-binding protein |
| GBAA0641 | *-* | amino acid abc transporter, permease protein |
| GBAA0642 | *-* | amino acid abc transporter, permease protein |
| GBAA4012 | *-* | cation-transporting atpase, e1-e2 family |
| GBAA1490 | *-* | d-alanyl-d-alanine carboxypeptidase family protein |
| GBAA0150 | *-* | polysaccharide deacetylase, putative |
| GBAA1492 | *-* | spore maturation protein |
| GBAA4530 | *-* | sporulation protein |
| GBAA4410 | *-* | stage iii sporulation protein ah |
| GBAA1020 | *-* | hypothetical protein |
| GBAA1201 | *-* | hypothetical protein |
| GBAA2012 | *-* | hypothetical protein |
| GBAA4138 | *-* | hypothetical protein |
| GBAA4198 | *-* | hypothetical protein |
| GBAA4418 | *-* | hypothetical protein |
| GBAA4501 | *-* | hypothetical protein |
| GBAA4645 | *-* | hypothetical protein |
| GBAA4691 | *-* | hypothetical protein |
| GBAA4821 | *-* | hypothetical protein |
| GBAA4851 | *-* | hypothetical protein |
| GBAA5207 | *-* | hypothetical protein |
|  |  |  |
| ***B. anthracis* cluster 32 elaboration genes** |  |  |
| **Locus** | **Name** | **Function** |
| GBAA5449 | *celA-3* | pts system, cellobiose-specific iib component |
| GBAA0146 | *cwlD* | germination-specific n-acetylmuramoyl-l-alanine amidase |
| GBAA5640 | *cwlJ-2* | cell wall hydrolase |
| GBAA4297 | *dacF* | d-alanyl-d-alanine carboxypeptidase |
| GBAA1530 | *spoIVA* | stage iv sporulation protein a |
| GBAA0767 | *spoVR* | stage v sporulation protein r |
| GBAA1221 | *-* | bacteriocin o-metyltransferase, putative |
| GBAA1755 | *-* | bnr repeat domain protein |
| GBAA3030 | *-* | catalase |
| GBAA3668 | *-* | glycosyl hydrolase, family 18 |
| GBAA0870 | *-* | hydrolase, haloacid dehalogenase-like family |
| GBAA4659 | *-* | lysm domain protein |
| GBAA2055 | *-* | magnesium transporter, cora family |
| GBAA2980 | *-* | polyketide synthesis domain protein |
| GBAA2981 | *-* | polyketide synthesis domain protein |
| GBAA4067 | *-* | prophage lambdaba02, ftsk/spoiiie family protein |
| GBAA2462 | *-* | pts system, cellobiose-specific iib component, putative |
| GBAA2463 | *-* | pts system, cellobiose-specific iic component, putative |
| GBAA5524 | *-* | stage ii sporulation protein |
| GBAA4692 | *-* | stage vi sporulation protein d, putative |
| GBAA2979 | *-* | transcriptional regulator, putative |
| GBAA0550 | *-* | hypothetical protein |
| GBAA0806 | *-* | hypothetical protein |
| GBAA0951 | *-* | hypothetical protein |
| GBAA1021 | *-* | hypothetical protein |
| GBAA1187 | *-* | hypothetical protein |
| GBAA1843 | *-* | hypothetical protein |
| GBAA1904 | *-* | hypothetical protein |
| GBAA2292 | *-* | hypothetical protein |
| GBAA2304 | *-* | hypothetical protein |
| GBAA2305 | *-* | hypothetical protein |
| GBAA2464 | *-* | hypothetical protein |
| GBAA2466 | *-* | hypothetical protein |
| GBAA2821 | *-* | hypothetical protein |
| GBAA2982 | *-* | hypothetical protein |
| GBAA3151 | *-* | hypothetical protein |
| GBAA3636 | *-* | hypothetical protein |
| GBAA3637 | *-* | hypothetical protein |
| GBAA3638 | *-* | hypothetical protein |
| GBAA3671 | *-* | hypothetical protein |
| GBAA3844 | *-* | hypothetical protein |
| GBAA4069 | *-* | hypothetical protein |
| GBAA4199 | *-* | hypothetical protein |
| GBAA4317 | *-* | hypothetical protein |
| GBAA4531 | *-* | hypothetical protein |
| GBAA4619 | *-* | hypothetical protein |
| GBAA5641 | *-* | hypothetical protein |
| GBAA5728 | *-* | hypothetical protein |

### B. subtilis - B. anthracis cluster 82

Figure S41: B. subtilis cluster 82 image (post-elaboration)

**
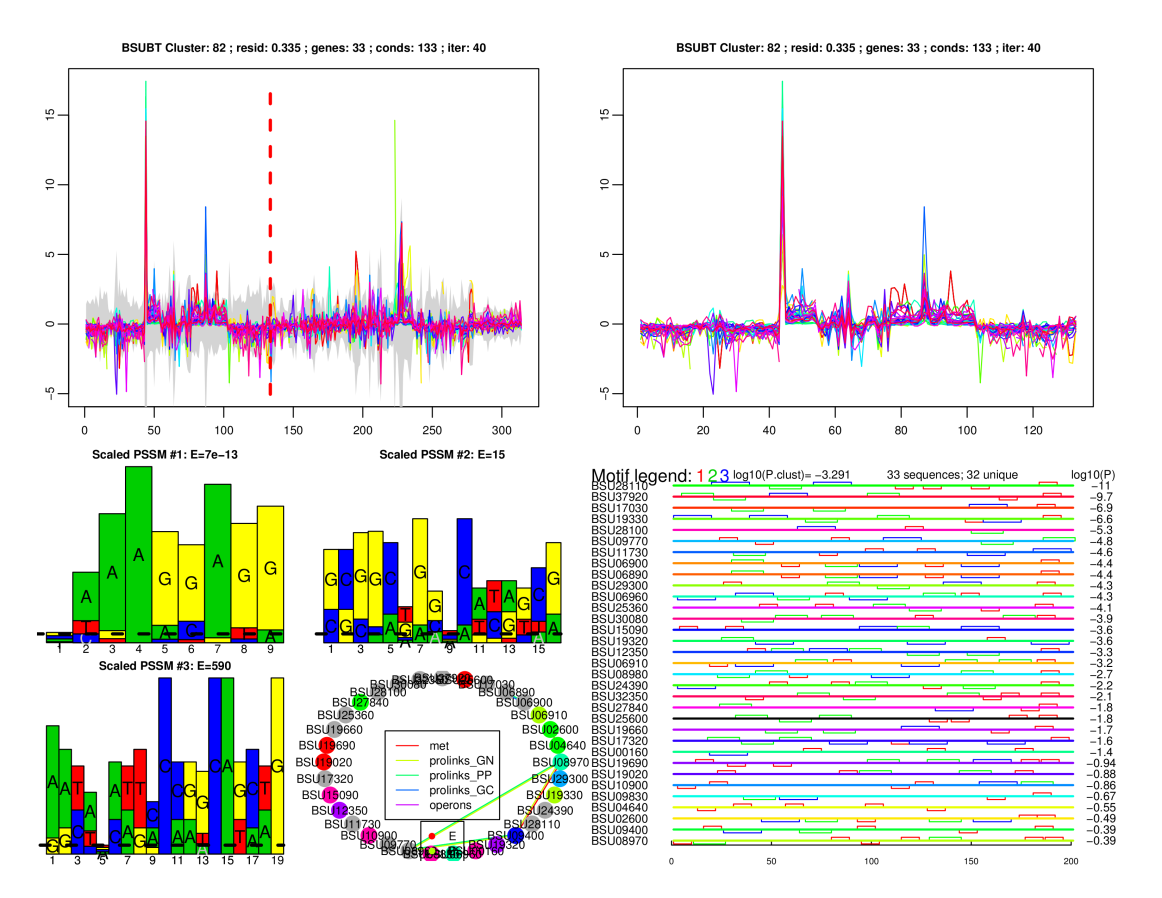
**

| ***B. subtilis* cluster 82 core genes** |  |  |
| --- | --- | --- |
| **Locus** | **Name** | **Function** |
| BSU04640 | *alr* | D-alanine racemase |
| BSU25600 | *comER* | late competence protein ComER |
| BSU17030 | *cotE* | morphogenic protein (spore coat protein) |
| BSU06890 | *cotJA* | polypeptide composition of the spore coat; required for the assembly of CotJC |
| BSU06900 | *cotJB* | polypeptide composition of the spore coat |
| BSU06910 | *cotJC* | polypeptide composition of the spore coat |
| BSU02600 | *cwlJ* | cell wall hydrolase (stored in the spore coat) |
| BSU08970 | *prkA* | serine protein kinase |
| BSU27840 | *safA* | morphogenetic protein associated with SpoVID (spore coat protein) |
| BSU19330 | *sodF* | superoxide dismutase |
| BSU28110 | *spoVID* | required for assembly of the spore coat (stage VI sporulation) |
| BSU09400 | *spoVR* | involved in spore cortex synthesis (stage V sporulation) |
| BSU19320 | *sqhC* | squalene-hopene cyclase |
| BSU00160 | *yaaH* | hypothetical protein (spore coat protein) |
| BSU09830 | *yhaX* | hypothetical protein (spore coat protein) |
| BSU08980 | *yhbH* | hypothetical protein |
| BSU10900 | *yisY* | hypothetical protein (spore coat protein) |
| BSU11730 | *yjbX* | hypothetical protein (spore coat protein, cotO) |
| BSU19020 | *yobN* | hypothetical protein |
| BSU19660 | *yozD* | hypothetical protein |
| BSU25360 | *yqfC* | hypothetical protein |
| BSU28100 | *ysxE* | hypothetical protein (spore coat protein) |
| BSU30080 | *yteV* | hypothetical protein |
| BSU32350 | *yunB* | hypothetical protein |
| BSU37920 | *ywdL* | hypothetical protein (spore coat protein, gerQ) |
|  |  |  |
| ***B. subtilis* cluster 82 elaboration genes** |  |  |
| **Locus** | **Name** | **Function** |
| BSU19690 | *kamA* | lysine 2,3-aminomutase |
| BSU29300 | *ribR* | riboflavin kinase |
| BSU24390 | *spoIIIAE* | mutants block sporulation after engulfment (stage III sporulation) |
| BSU12350 | *yjmF* | D-mannonate oxidoreductase |
| BSU06960 | *yesN* | hypothetical protein |
| BSU09770 | *yheD* | hypothetical protein |
| BSU15090 | *ylbO* | hypothetical protein |
| BSU17320 | *ymaF* | hypothetical protein |

Note: spore coat protein assignments are according to Henriques and Moran (2007), Ann Rev Microbiol

Figure S42: B. anthracis cluster 82 image (post-elaboration)

**
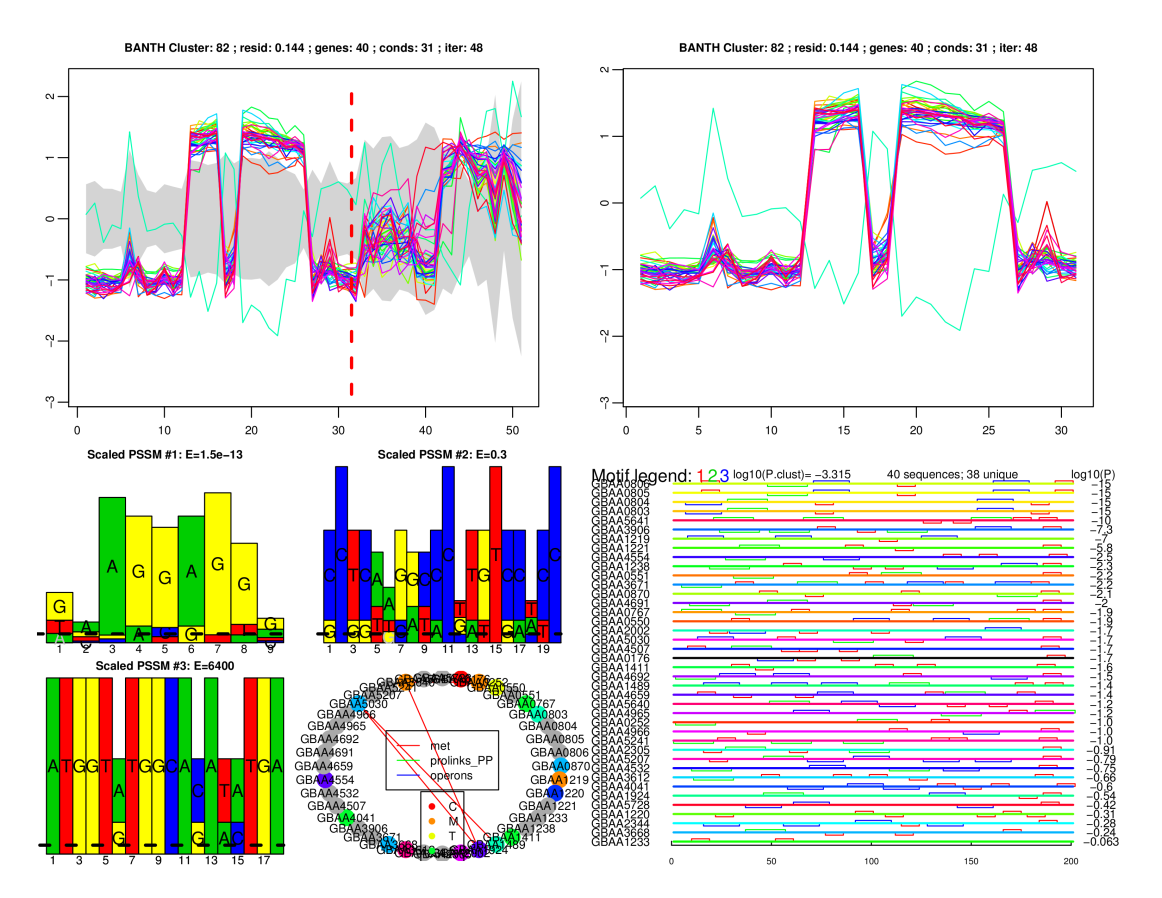
**

| ***B. anthracis* cluster 82 core genes** |  |  |
| --- | --- | --- |
| **Locus** | **Name** | **Function** |
| GBAA3906 | *cotE* | spore coat protein e |
| GBAA0805 | *cotJA* | cotja protein |
| GBAA0804 | *cotJB* | cotjb protein |
| GBAA0803 | *cotJC* | cotjc protein |
| GBAA5640 | *cwlJ-2* | cell wall hydrolase |
| GBAA0252 | *dal-1* | alanine racemase |
| GBAA0767 | *spoVR* | stage v sporulation protein r |
| GBAA1924 | *-* | amine oxidase, flavin-containing |
| GBAA3668 | *-* | glycosyl hydrolase, family 18 |
| GBAA5030 | *-* | hydrolase, alpha/beta fold family |
| GBAA0870 | *-* | hydrolase, haloacid dehalogenase-like family |
| GBAA4554 | *-* | late competence protein ComER |
| GBAA4659 | *-* | lysm domain protein |
| GBAA3612 | *-* | squalene-hopene cyclase |
| GBAA4692 | *-* | stage vi sporulation protein d, putative |
| GBAA1489 | *-* | superoxide dismutase |
| GBAA0550 | *-* | hypothetical protein |
| GBAA0551 | *-* | hypothetical protein |
| GBAA1233 | *-* | hypothetical protein |
| GBAA2305 | *-* | hypothetical protein |
| GBAA4532 | *-* | hypothetical protein |
| GBAA4691 | *-* | hypothetical protein |
| GBAA4965 | *-* | hypothetical protein |
| GBAA5207 | *-* | hypothetical protein |
| GBAA5641 | *-* | hypothetical protein |
|  |  |  |
| ***B. anthracis* cluster 82 elaboration genes** |  |  |
| **Locus** | **Name** | **Function** |
| GBAA1238 | *cotZ-2* | spore coat protein z |
| GBAA0176 | *-* | alcohol dehydrogenase, zinc-containing |
| GBAA1221 | *-* | bacteriocin o-metyltransferase, putative |
| GBAA1219 | *-* | glycosyl transferase, group 2 family protein |
| GBAA5241 | *-* | spore coat protein f-related protein |
| GBAA2002 | *-* | transcriptional regulator, arsr family |
| GBAA0806 | *-* | hypothetical protein |
| GBAA1220 | *-* | hypothetical protein |
| GBAA1411 | *-* | hypothetical protein |
| GBAA2344 | *-* | hypothetical protein |
| GBAA3671 | *-* | hypothetical protein |
| GBAA4041 | *-* | hypothetical protein |
| GBAA4507 | *-* | hypothetical protein |
| GBAA4966 | *-* | hypothetical protein |
| GBAA5728 | *-* | hypothetical protein |
|  |  |  |
| ***B. anthracis* cluster 82 core genes** |  |  |
| **Locus** | **Name** | **Function** |
| GBAA3906 | *cotE* | spore coat protein E |
| GBAA0805 | *cotJA* | cotja protein |
| GBAA0804 | *cotJB* | cotjb protein |
| GBAA0803 | *cotJC* | cotjc protein |
| GBAA5640 | *cwlJ-2* | cell wall hydrolase |
| GBAA0252 | *dal-1* | alanine racemase |
| GBAA0767 | *spoVR* | stage v sporulation protein r |
| GBAA1924 | *-* | amine oxidase, flavin-containing |
| GBAA3668 | *-* | glycosyl hydrolase, family 18 |
| GBAA5030 | *-* | hydrolase, alpha/beta fold family |
| GBAA0870 | *-* | hydrolase, haloacid dehalogenase-like family |
| GBAA4554 | *-* | late competence protein ComER |
| GBAA4659 | *-* | lysm domain protein |
| GBAA3612 | *-* | squalene-hopene cyclase |

### B. subtilis - B. anthracis cluster 84

Figure S43: B. subtilis cluster 84 image (post-elaboration)

**
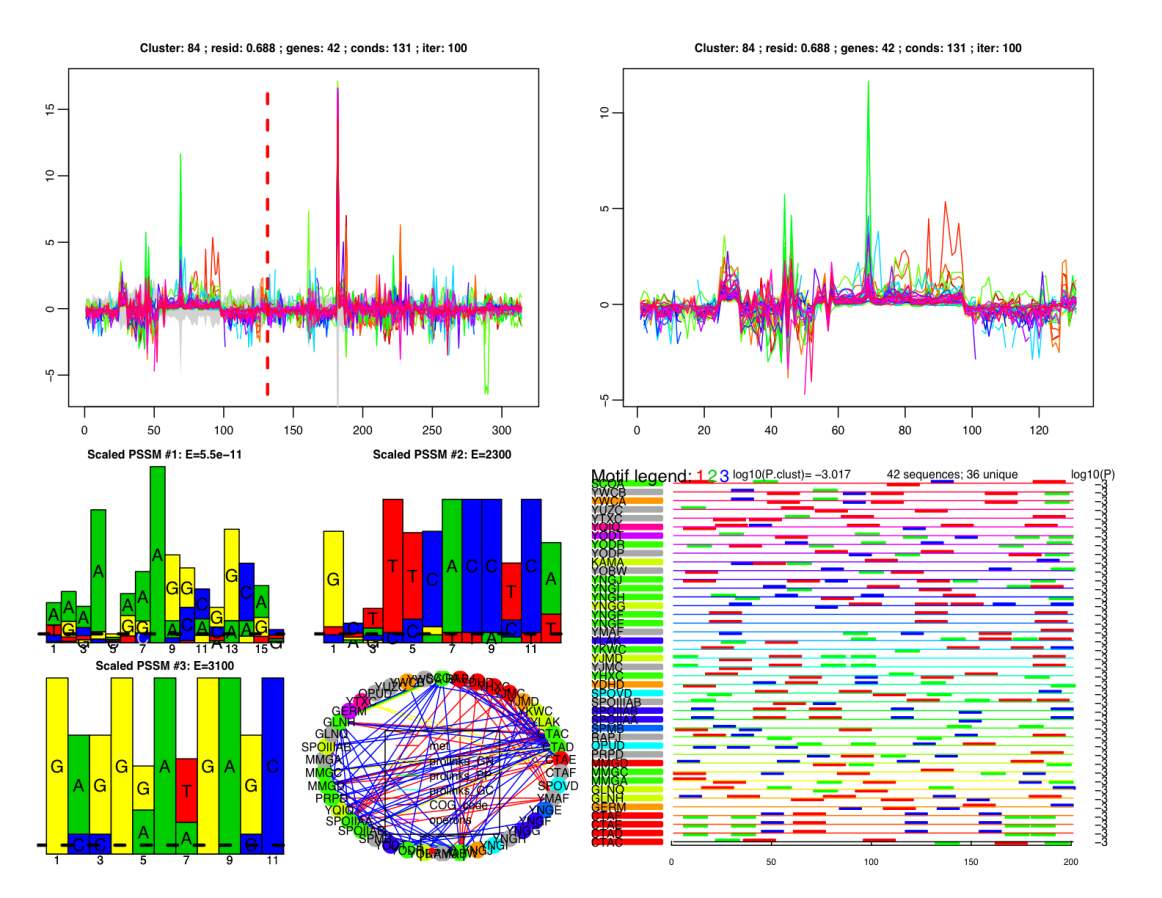
**

| ***B. subtilis* cluster 84 core genes** |  |  |
| --- | --- | --- |
| **Locus** | **Name** | **Function** |
| BSU14890 | *ctaC* | cytochrome caa3 oxidase (subunit II) |
| BSU14900 | *ctaD* | cytochrome caa3 oxidase (subunit I) |
| BSU14910 | *ctaE* | cytochrome caa3 oxidase (subunit III) |
| BSU14920 | *ctaF* | cytochrome caa3 oxidase (subunit IV) |
| BSU19690 | *kamA* | lysine 2,3-aminomutase |
| BSU24170 | *mmgA* | acetyl-CoA acetyltransferase |
| BSU24150 | *mmgC* | acyl-CoA dehydrogenase |
| BSU24140 | *mmgD* | citrate synthase 3 |
| BSU30070 | *opuD* | glycine betaine transporter |
| BSU24130 | *prpD* | 2-methylcitrate dehydratase |
| BSU02820 | *rapJ* | response regulator aspartate phosphatase |
| BSU23470 | *spoIIAA* | anti-anti-sigma factor (antagonist of SpoIIAB) |
| BSU23460 | *spoIIAB* | anti-sigma F factor |
| BSU18220 | *yngF* | enoyl-CoA hydratase |
| BSU18230 | *yngG* | hydroxymethylglutaryl-CoA lyase |
| BSU18240 | *yngH* | acetyl-CoA carboxylase biotin carboxylase subunit |
| BSU13960 | *ykwC* | hypothetical protein |
| BSU14810 | *ylaK* | hypothetical protein |
| BSU18210 | *yngE* | hypothetical protein |
| BSU18260 | *yngJ* | hypothetical protein |
| BSU19700 | *yodP* | hypothetical protein |
| BSU19720 | *yodR* | hypothetical protein |
| BSU19740 | *yodT* | hypothetical protein |
| BSU24120 | *yqiQ* | hypothetical protein |
| BSU38240 | *ywcA* | hypothetical protein |
| BSU38230 | *ywcB* | hypothetical protein |
|  |  |  |
| ***B. subtilis* cluster 84 elaboration genes** |  |  |
| **Locus** | **Name** | **Function** |
| BSU28380 | *gerM* | germination (cortex hydrolysis) and sporulation (stage II, multiple polar septa) |
| BSU27440 | *glnH* | glutamine ABC transporter (glutamine-binding protein) |
| BSU27430 | *glnQ* | glutamine ABC transporter (ATP-binding protein) |
| BSU23170 | *spmB* | spore maturation protein |
| BSU24420 | *spoIIIAB* | stage III sporulation protein SpoAB |
| BSU15170 | *spoVD* | penicillin-binding protein |
| BSU18250 | *yngI* | acyl-CoA synthetase |
| BSU38990 | *scoA* | succinyl CoA:3-oxoacid CoA-transferase (subunit A) |
| BSU05710 | *ydhD* | hypothetical protein |
| BSU10400 | *yhxC* | hypothetical protein |
| BSU12320 | *yjmC* | hypothetical protein |
| BSU12330 | *yjmD* | hypothetical protein |
| BSU17320 | *ymaF* | hypothetical protein |
| BSU19110 | *yobW* | hypothetical protein |
| BSU28960 | *ytxC* | hypothetical protein |
| BSU31730 | *yuzC* | hypothetical protein |

Figure S44: B. anthracis cluster 84 image (post-elaboration)

**
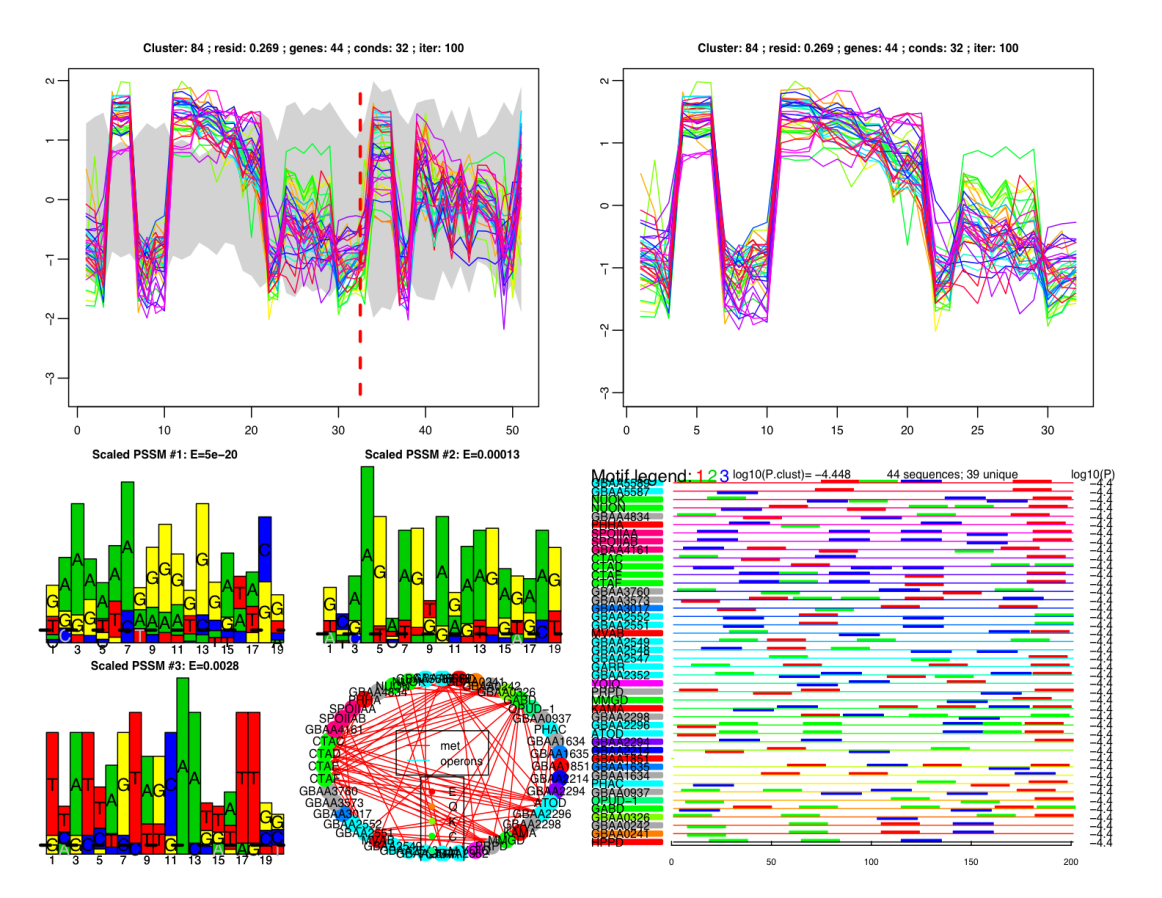
**

| ***B. anthracis* cluster 84 core genes** |  |  |
| --- | --- | --- |
| **Locus** | **Name** | **Function** |
| GBAA4154 | *ctaC* | cytochrome c oxidase, subunit ii |
| GBAA4153 | *ctaD* | cytochrome c oxidase, subunit i |
| GBAA4152 | *ctaE* | cytochrome c oxidase, subunit iii |
| GBAA4151 | *ctaF* | cytochrome c oxidase, subunit ivb |
| GBAA2353 | *garR* | 2-hydroxy-3-oxopropionate reductase |
| GBAA2300 | *kamA* | l-lysine 2,3-aminomutase |
| GBAA2348 | *mmgD* | citrate synthase |
| GBAA2550 | *mvaB* | hydroxymethylglutaryl-CoA lyase |
| GBAA0554 | *opuD-1* | glycine betaine transporter |
| GBAA2349 | *prpD* | 2-methylcitrate dehydratase |
| GBAA4296 | *spoIIAA* | anti-sigma f factor antagonist |
| GBAA4295 | *spoIIAB* | anti-sigma F factor |
| GBAA2350 | *yqiQ* | carboxyvinyl-carboxyphosphonate phosphorylmutase |
| GBAA5589 | *-* | acetyl-CoA acetyltransferase |
| GBAA2548 | *-* | acetyl-CoA carboxylase |
| GBAA2298 | *-* | acetyltransferase, gnat family |
| GBAA2547 | *-* | acyl-coa dehydrogenase |
| GBAA5587 | *-* | acyl-coa dehydrogenase |
| GBAA2552 | *-* | carboxyl transferase domain protein |
| GBAA2296 | *-* | coa-transferase, beta subunit |
| GBAA2551 | *-* | enoyl-CoA hydratase |
| GBAA4161 | *-* | phoh family protein |
| GBAA3760 | *-* | prophage lambdaba01, tpr domain protein, putative |
| GBAA1635 | *-* | sodium/solute symporter family protein |
| GBAA1634 | *-* | hypothetical protein |
| GBAA2294 | *-* | hypothetical protein |
|  |  |  |
| ***B. anthracis* cluster 84 elaboration genes** |  |  |
| **Locus** | **Name** | **Function** |
| GBAA2295 | *atoD* | acetate coa-transferase, subunit a |
| GBAA0327 | *gabD* | succinate-semialdehyde dehydrogenase (nadp+) |
| GBAA0240 | *hppD* | 4-hydroxyphenylpyruvate dioxygenase |
| GBAA1851 | *ilvB-2* | acetolactate synthase III large subunit |
| GBAA5535 | *nuoK* | NADH dehydrogenase kappa subunit |
| GBAA5532 | *nuoN* | NADH dehydrogenase subunit N |
| GBAA1331 | *phaC* | poly(r)-hydroxyalkanoic acid synthase, class iii, phac subunit |
| GBAA2549 | *-* | acetyl-CoA carboxylase |
| GBAA2352 | *-* | acyl-coa dehydrogenase |
| GBAA0241 | *-* | fumarylacetoacetate hydrolase family protein |
| GBAA0242 | *-* | homogentisate 1,2-dioxygenase, putative |
| GBAA4586 | *-* | phenylalanine-4-hydroxylase, putative |
| GBAA0326 | *-* | sensory box sigma-54 dependent dna-binding response regulator |
| GBAA0937 | *-* | hypothetical protein |
| GBAA2214 | *-* | hypothetical protein |
| GBAA3017 | *-* | hypothetical protein |
| GBAA3573 | *-* | hypothetical protein |
| GBAA4834 | *-* | hypothetical protein |

## Gene lists for flagellar clusters

- *B. subtilis* - *B. anthracis* cluster 58
- *B. subtilis* - *L. monocytogenes* cluster 79
- *B. anthracis* - *L. monocytogenes* cluster 102

### B. subtilis - B. anthracis cluster 58:

Figure S45: B. subtilis cluster 58 image (post-elaboration)

**
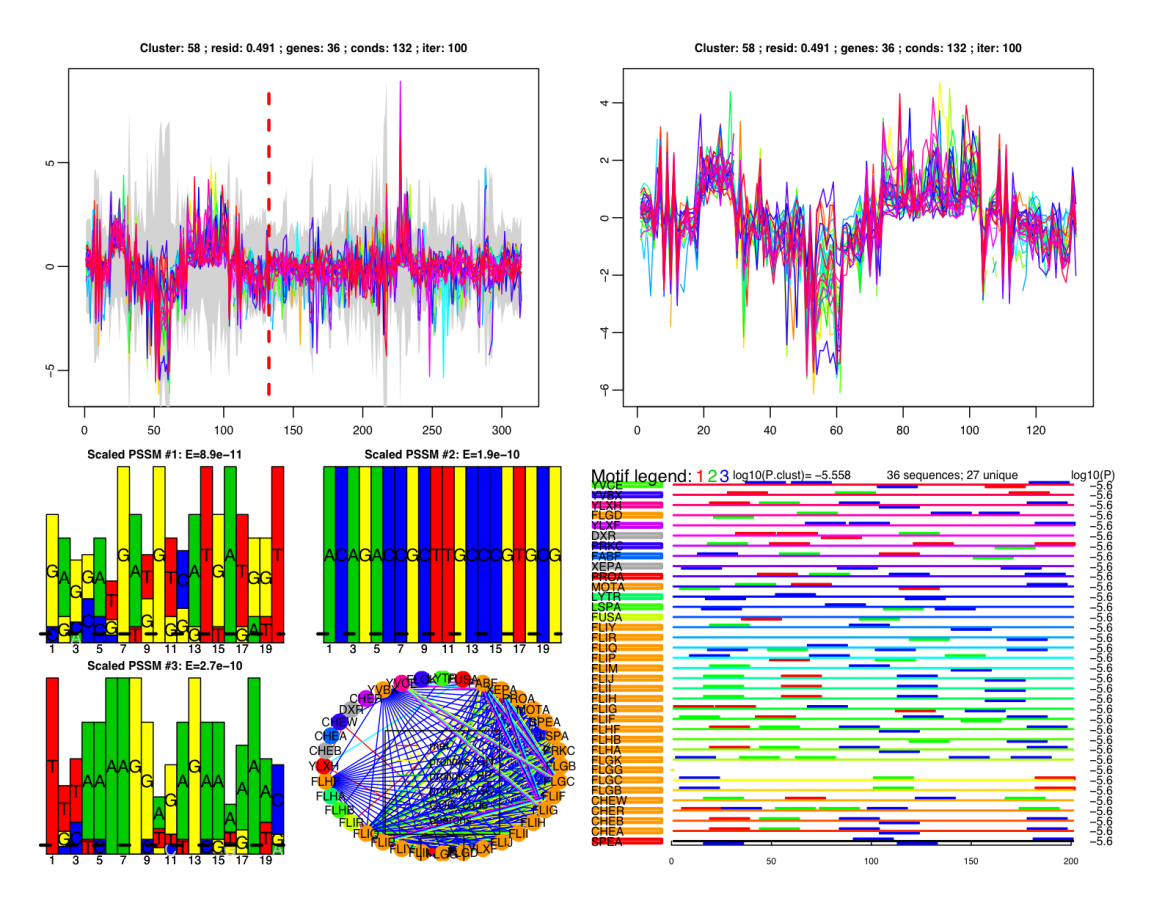
**

| ***B. subtilis* cluster 58 shared** |  |  |
| --- | --- | --- |
| **Locus** | **Name** | **Function** |
| BSU13130 | *proA* | gamma-glutamyl phosphate reductase |
| BSU13690 | *motA* | flagellar motor protein MotA |
| BSU16180 | *flgB* | flagellar basal body rod protein FlgB |
| BSU16190 | *flgC* | flagellar basal body rod protein FlgC |
| BSU16220 | *fliG* | flagellar motor switch protein G |
| BSU16240 | *fliI* | flagellum-specific ATP synthase |
| BSU16290 | *flgG* | flagellar basal body rod protein FlgG |
| BSU16320 | *fliY* | flagellar motor switch protein |
| BSU16350 | *fliP* | flagellar biosynthesis protein FliP |
| BSU16360 | *fliQ* | flagellar biosynthesis protein FliQ |
| BSU16370 | *fliR* | flagellar biosynthesis protein FliR |
| BSU16380 | *flhB* | flagellar biosynthesis protein FlhB |
| BSU16390 | *flhA* | flagellar biosynthesis protein A |
| BSU16400 | *flhF* | flagellar biosynthesis regulator FlhF |
| BSU22720 | *cheR* | methyl-accepting chemotaxis proteins (MCPs) methyltransferase |
| BSU34800 | *yvcE* | hypothetical protein |
| BSU35410 | *flgK* | flagellar hook-associated protein FlgK |
| BSU35650 | *lytR* | membrane-bound transcriptional regulator LytR |
| ***B. subtilis* cluster 58 elaboration genes** |  |  |
| **Locus** | **Name** | **Function** |
| BSU01120 | *fusA* | elongation factor G |
| BSU11340 | *fabF* | 3-oxoacyl-(acyl carrier protein) synthase II |
| BSU12780 | *xepA* | lytic exoenzyme associated with defective prophage PBSX |
| BSU14630 | *speA* | arginine decarboxylase |
| BSU15450 | *lspA* | lipoprotein signal peptidase |
| BSU15770 | *prkC* | protein kinase |
| BSU16210 | *fliF* | flagellar MS-ring protein |
| BSU16230 | *fliH* | flagellar assembly protein H |
| BSU16250 | *fliJ* | flagellar biosynthesis chaperone |
| BSU16260 | *ylxF* | hypothetical protein |
| BSU16280 | *flgD* | flagellar basal body rod modification protein |
| BSU16310 | *fliM* | flagellar motor switch protein FliM |
| BSU16410 | *ylxH* | hypothetical protein |
| BSU16420 | *cheB* | chemotaxis-specific methylesterase |
| BSU16430 | *cheA* | two-component sensor histidine kinase |
| BSU16440 | *cheW* | modulation of CheA activity in response to attractants (chemotaxis) |
| BSU16550 | *dxr* | 1-deoxy-D-xylulose 5-phosphate reductoisomerase |
| BSU34020 | *yvbX* | hypothetical protein |

Figure S46: B. anthracis cluster 58 image (post-elaboration)

**
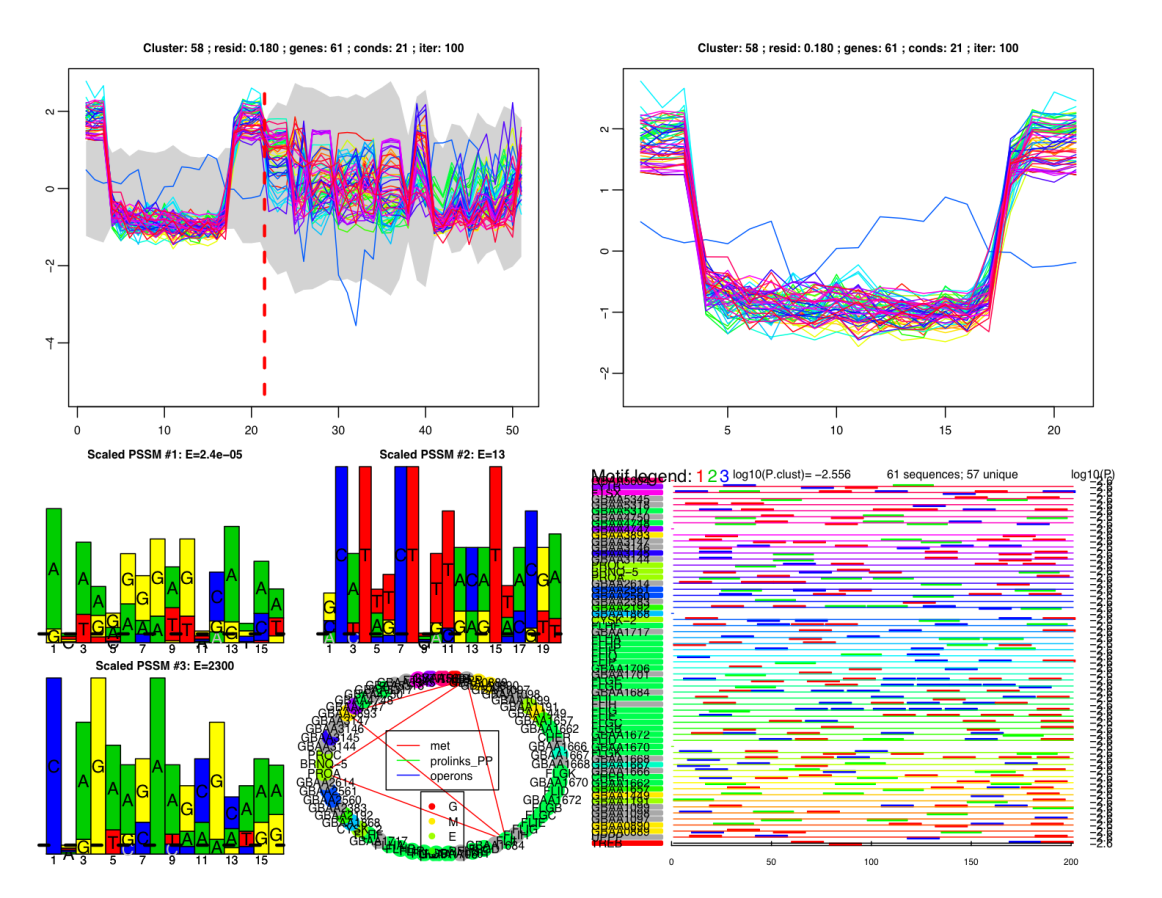
**

| ***B. anthracis* cluster 58 core genes** |  |  |
| --- | --- | --- |
| **Locus** | **Name** | **Function** |
| GBAA1449 | *-* | peptidase, m23/m37 family |
| GBAA1662 | *-* | flagellar motor switch protein |
| GBAA1665 | *cheR* | chemotaxis protein methyltransferase cher |
| GBAA1669 | *-* | flagellar hook-associated protein |
| GBAA1674 | *flgB* | flagellar basal body rod protein |
| GBAA1675 | *flgC* | flagellar basal body rod protein |
| GBAA1679 | *fliG* | flagellar motor protein |
| GBAA1681 | *-* | flagellum-specific ATP synthase |
| GBAA1686 | *-* | flagellar hook protein |
| GBAA1712 | *-* | flagellar biosynthesis protein |
| GBAA1713 | *-* | flagellar biosynthesis protein |
| GBAA1714 | *fliR* | flagellar biosynthesis protein |
| GBAA1715 | *-* | flagellar biosynthesis protein |
| GBAA1716 | *flhA* | flagellar biosynthesis protein |
| GBAA1718 | *-* | flagellar biosynthesis protein |
| GBAA2992 | *proA* | gamma-glutamyl phosphate reductase |
| GBAA4748 | *-* | flagellar motor protein |
| GBAA5506 | *lytR* | membrane-bound transcriptional regulator LytR |
| ***B. anthracis* cluster 58 elaboration genes** |  |  |
| **Locus** | **Name** | **Function** |
| GBAA0631 | *treB* | pts system, trehalose-specific iibc component |
| GBAA0683 | *uppP* | undecaprenyl pyrophosphate phosphatase |
| GBAA0889 | *-* | alginate o-acetyltransferase, putative |
| GBAA0890 | *-* | alginate o-acetyltransferase, putative |
| GBAA1097 | *-* | hypothetical protein |
| GBAA1098 | *-* | wall-associated domain protein |
| GBAA1099 | *-* | hypothetical protein |
| GBAA1191 | *-* | oligopeptide abc transporter, oligopeptide-binding protein |
| GBAA1657 | *-* | hypothetical protein |
| GBAA1666 | *-* | hypothetical protein |
| GBAA1667 | *-* | hypothetical protein |
| GBAA1668 | *-* | hypothetical protein |
| GBAA1670 | *-* | flagellar hook-associated protein |
| GBAA1671 | *-* | flagellar hook-associated protein |
| GBAA1672 | *-* | flagellar protein flis, putative |
| GBAA1676 | *-* | flagellar basal body protein |
| GBAA1680 | *-* | hypothetical protein |
| GBAA1684 | *-* | hypothetical protein |
| GBAA1685 | *-* | flagellar hook assembly protein |
| GBAA1701 | *-* | hypothetical protein |
| GBAA1706 | *-* | flagellin |
| GBAA1717 | *-* | hypothetical protein |
| GBAA1831 | *cysK-2* | cysteine synthase a |
| GBAA1868 | *-* | hydrolase, alpha/beta fold family |
| GBAA2192 | *-* | hypothetical protein |
| GBAA2383 | *-* | hypothetical protein |
| GBAA2560 | *-* | sensor histidine kinase |
| GBAA2561 | *-* | dna-binding response regulator |
| GBAA2614 | *-* | hypothetical protein |
| GBAA3142 | *brnQ-5* | branched-chain amino acid transport system ii carrier protein |
| GBAA3143 | *proC* | pyrroline-5-carboxylate reductase |
| GBAA3144 | *-* | hypothetical protein |
| GBAA3145 | *-* | malate dehydrogenase, putative |
| GBAA3146 | *-* | hypothetical protein |
| GBAA3147 | *-* | hypothetical protein |
| GBAA3893 | *-* | cell wall hydrolase, putative |
| GBAA4747 | *-* | dna-binding protein |
| GBAA4750 | *-* | d-alanyl-d-alanine carboxypeptidase family protein |
| GBAA5317 | *-* | methyl-accepting chemotaxis protein |
| GBAA5318 | *-* | endonuclease/exonuclease/phosphatase family |
| GBAA5345 | *-* | hypothetical protein |
| GBAA5415 | *ftsX* | cell division abc transporter, permease protein ftsx |
| GBAA5604 | *-* | abc transporter, atp-binding protein |

### B. subtilis - L. monocytogenes cluster 79

Figure S47: B. subtilis cluster 79 image (post-elaboration)

**
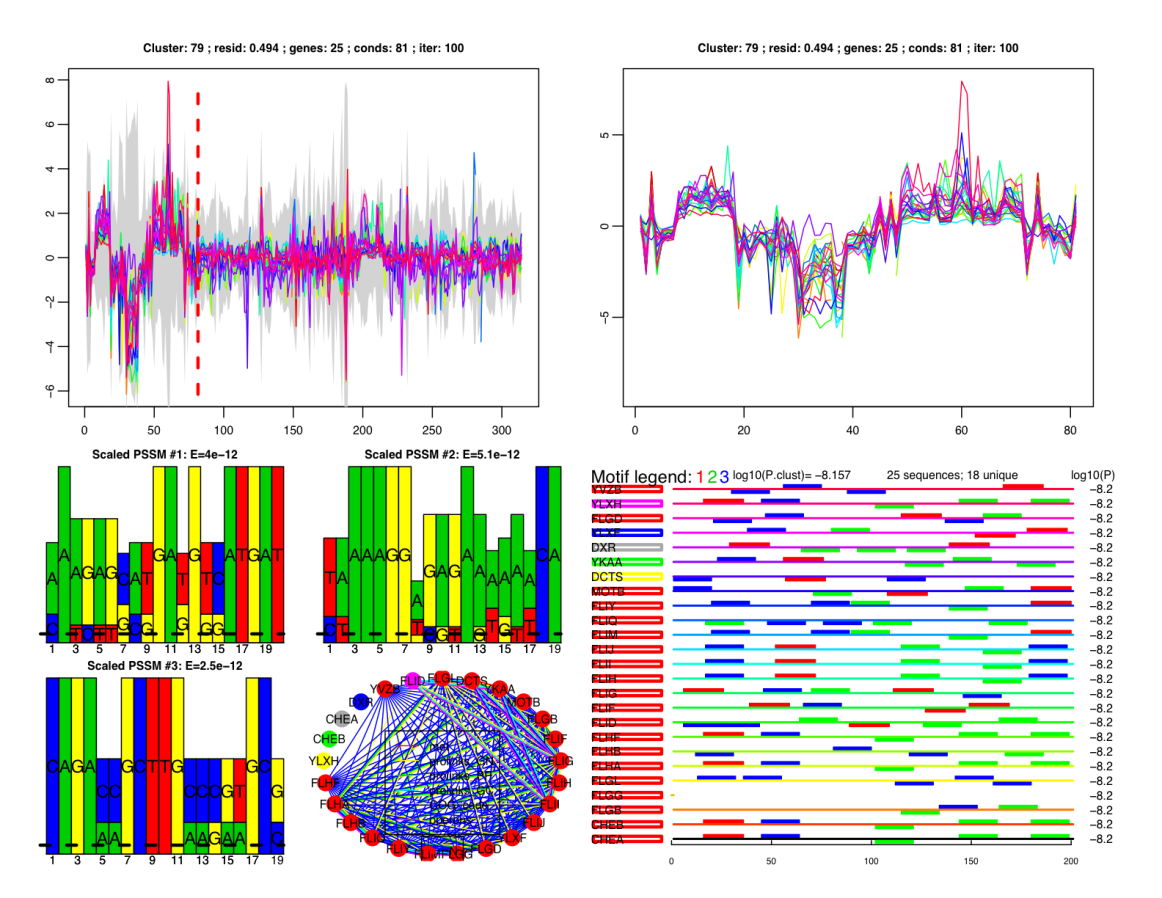
**

| ***B. subtilis* cluster 79 shared** |  |  |
| --- | --- | --- |
| **Locus** | **Name** | **Function** |
| BSU12850 | *ykaA* | hypothetical protein |
| BSU13680 | *motB* | flagellar motor protein MotB |
| BSU16180 | *flgB* | flagellar basal body rod protein FlgB |
| BSU16210 | *fliF* | flagellar MS-ring protein |
| BSU16220 | *fliG* | flagellar motor switch protein G |
| BSU16240 | *fliI* | flagellum-specific ATP synthase |
| BSU16280 | *flgD* | flagellar basal body rod modification protein |
| BSU16290 | *flgG* | flagellar basal body rod protein FlgG |
| BSU16310 | *fliM* | flagellar motor switch protein FliM |
| BSU16320 | *fliY* | flagellar motor switch protein |
| BSU16380 | *flhB* | flagellar biosynthesis protein FlhB |
| BSU16390 | *flhA* | flagellar biosynthesis protein A |
| BSU16400 | *flhF* | flagellar biosynthesis regulator FlhF |
| BSU16430 | *cheA* | two-component sensor histidine kinase |
| BSU16550 | *dxr* | 1-deoxy-D-xylulose 5-phosphate reductoisomerase |
| BSU35150 | *yvzB* | hypothetical protein |
| BSU35340 | *fliD* | flagellar capping protein |
| BSU35400 | *flgL* | flagellar hook-associated protein FlgL |
| ***B. subtilis* cluster 79 elaboration genes** |  |  |
| **Locus** | **Name** | **Function** |
| BSU04450 | *dctS* | two-component sensor histidine kinase |
| BSU16230 | *fliH* | flagellar assembly protein H |
| BSU16250 | *fliJ* | flagellar biosynthesis chaperone |
| BSU16260 | *ylxF* | hypothetical protein |
| BSU16360 | *fliQ* | flagellar biosynthesis protein FliQ |
| BSU16410 | *ylxH* | hypothetical protein |
| BSU16420 | *cheB* | chemotaxis-specific methylesterase |

Figure S48: L. monocytogenes cluster 79 image (post-elaboration)

**
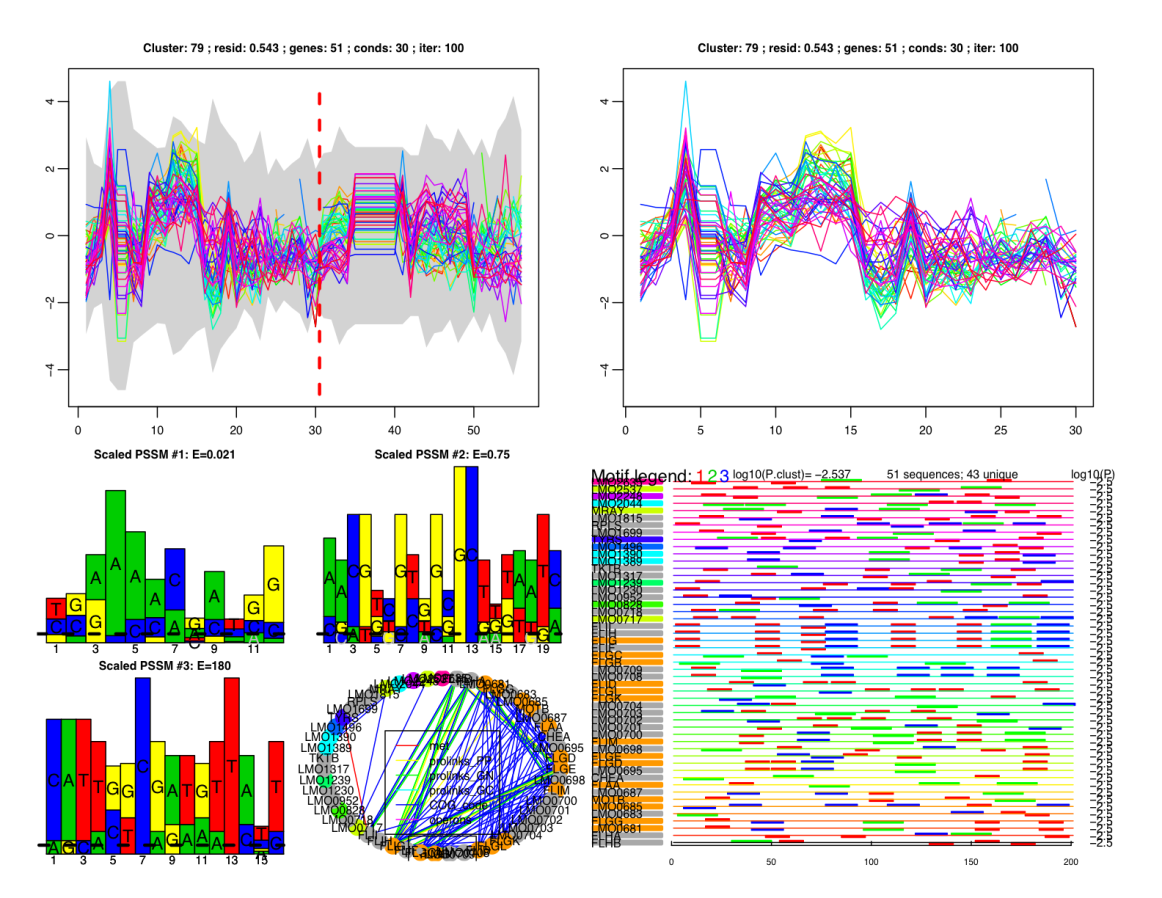
**

| ***L. monocytogenes* cluster 79 shared** |  |  |
| --- | --- | --- |
| **Locus** | **Name** | **Function** |
| LMO0679 | *flhB* | flagellar biosynthesis protein FlhB |
| LMO0680 | *flhA* | flagellar biosynthesis protein A |
| LMO0681 | *-* | flagellar biosynthesis regulator FlhF |
| LMO0686 | *motB* | hypothetical protein |
| LMO0690 | *flaA* | flagellin |
| LMO0692 | *cheA* | two-component sensor histidine kinase CheA |
| LMO0696 | *flgD* | flagellar basal body rod modification protein |
| LMO0697 | *flgE* | flagellar hook protein FlgE |
| LMO0699 | *fliM* | flagellar motor switch protein FliM |
| LMO0700 | *-* | flagellar motor switch protein |
| LMO0706 | *flgL* | flagellar hook-associated protein FlgL |
| LMO0707 | *fliD* | flagellar capping protein |
| LMO0710 | *flgB* | flagellar basal body rod protein FlgB |
| LMO0713 | *fliF* | flagellar MS-ring protein |
| LMO0714 | *fliG* | flagellar motor switch protein G |
| LMO0716 | *fliI* | flagellum-specific ATP synthase |
| LMO1317 | *-* | 1-deoxy-D-xylulose 5-phosphate reductoisomerase |
| LMO2248 | *-* | hypothetical protein |
| ***L. monocytogenes* cluster 79 elaboration genes** |  |  |
| **Locus** | **Name** | **Function** |
| LMO0682 | *flgG* | flagellar basal body rod protein FlgG |
| LMO0683 | *-* | hypothetical protein |
| LMO0685 | *-* | flagellar motor protein MotA |
| LMO0687 | *-* | hypothetical protein |
| LMO0695 | *-* | hypothetical protein |
| LMO0698 | *-* | flagellar motor switch protein |
| LMO0701 | *-* | hypothetical protein |
| LMO0702 | *-* | hypothetical protein |
| LMO0703 | *-* | hypothetical protein |
| LMO0704 | *-* | hypothetical protein |
| LMO0705 | *flgK* | flagellar hook-associated protein FlgK |
| LMO0708 | *-* | hypothetical protein |
| LMO0709 | *-* | hypothetical protein |
| LMO0711 | *flgC* | flagellar basal body rod protein FlgC |
| LMO0715 | *fliH* | flagellar assembly protein H |
| LMO0717 | *-* | hypothetical protein |
| LMO0718 | *-* | hypothetical protein |
| LMO0828 | *-* | hypothetical protein |
| LMO0952 | *-* | hypothetical protein |
| LMO1230 | *-* | hypothetical protein |
| LMO1239 | *-* | hypothetical protein |
| LMO1365 | *tktB* | 1-deoxy-D-xylulose-5-phosphate synthase |
| LMO1389 | *-* | hypothetical protein |
| LMO1390 | *-* | hypothetical protein |
| LMO1496 | *-* | hypothetical protein |
| LMO1598 | *tyrS* | tyrosyl-tRNA synthetase |
| LMO1699 | *-* | hypothetical protein |
| LMO1787 | *rplS* | 50S ribosomal protein L19 |
| LMO1815 | *-* | hypothetical protein |
| LMO2037 | *mraY* | hypothetical protein |
| LMO2044 | *-* | hypothetical protein |
| LMO2537 | *-* | hypothetical protein |
| LMO2635 | *-* | 1,4-dihydroxy-2-naphthoate octaprenyltransferase |

### B. anthracis - L. monocytogenes cluster 102

Figure S49: B. anthracis cluster 102 image (post-elaboration)

**
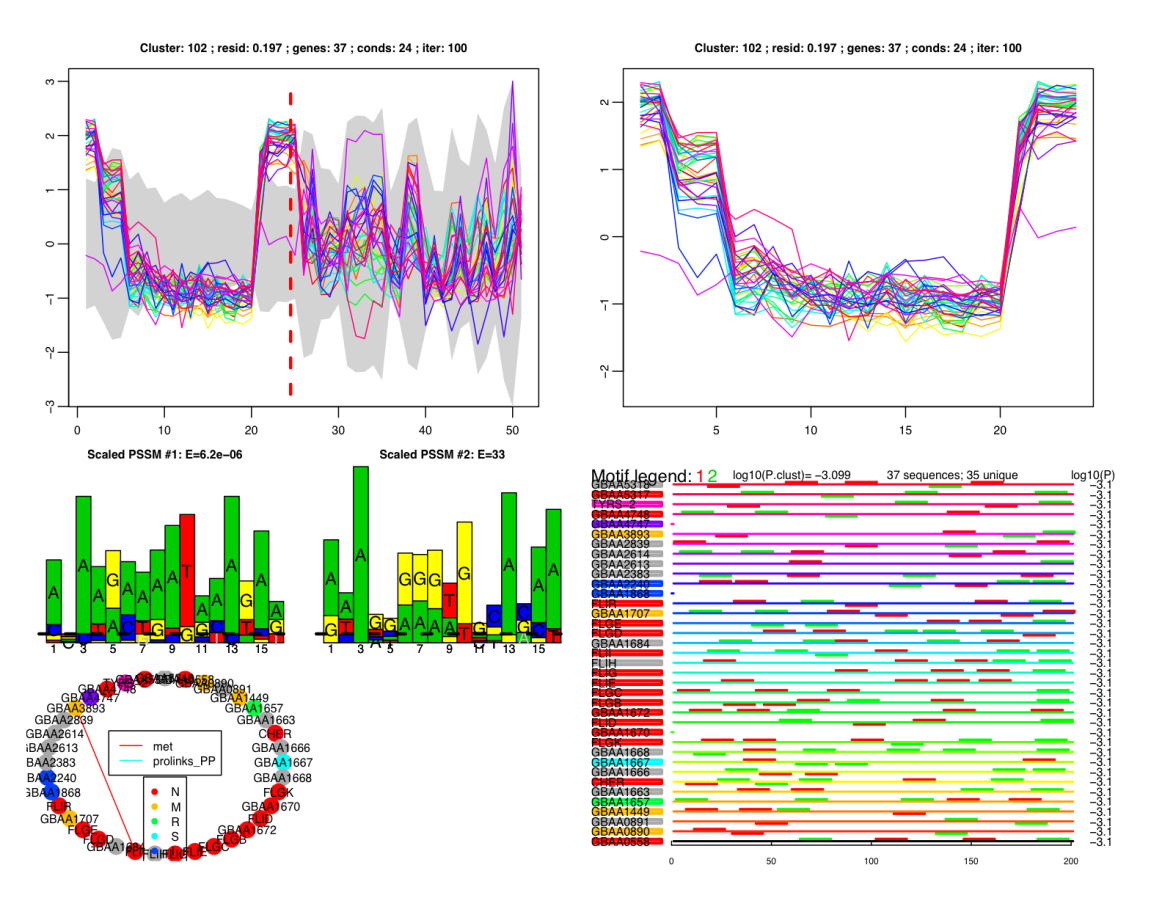
**

| ***B. anthracis* cluster 102 core genes** |  |  |
| --- | --- | --- |
| **Locus** | **Name** | **Function** |
| GBAA1667 | *-* | hypothetical protein |
| GBAA1669 | *-* | flagellar hook-associated protein |
| GBAA1670 | *-* | flagellar hook-associated protein |
| GBAA1672 | *-* | flagellar protein flis, putative |
| GBAA1674 | *flgB* | flagellar basal body rod protein |
| GBAA1675 | *flgC* | flagellar basal body rod protein |
| GBAA1676 | *-* | flagellar basal body protein |
| GBAA1679 | *fliG* | flagellar motor protein |
| GBAA1680 | *-* | hypothetical protein |
| GBAA1681 | *-* | flagellum-specific ATP synthase |
| GBAA1685 | *-* | flagellar hook assembly protein |
| GBAA1686 | *-* | flagellar hook protein |
| GBAA1707 | *-* | transglycosylase, slt family |
| GBAA1714 | *fliR* | flagellar biosynthesis protein |
| GBAA5314 | *tyrS-2* | tyrosyl-tRNA synthetase |
|  |  |  |
| ***B. anthracis* cluster 102 elaboration genes** |  |  |
| **Locus** | **Name** | **Function** |
| GBAA0558 | *-* | methyl-accepting chemotaxis protein |
| GBAA0890 | *-* | alginate o-acetyltransferase, putative |
| GBAA0891 | *-* | hypothetical protein |
| GBAA1449 | *-* | peptidase, m23/m37 family |
| GBAA1657 | *-* | hypothetical protein |
| GBAA1663 | *-* | hypothetical protein |
| GBAA1665 | *cheR* | chemotaxis protein methyltransferase cher |
| GBAA1666 | *-* | hypothetical protein |
| GBAA1668 | *-* | hypothetical protein |
| GBAA1671 | *-* | flagellar hook-associated protein |
| GBAA1684 | *-* | hypothetical protein |
| GBAA1868 | *-* | hydrolase, alpha/beta fold family |
| GBAA2240 | *-* | 1-acyl-sn-glycerol-3-phosphate acyltransferase, putative |
| GBAA2383 | *-* | hypothetical protein |
| GBAA2613 | *-* | hypothetical protein |
| GBAA2614 | *-* | hypothetical protein |
| GBAA2839 | *-* | hypothetical protein |
| GBAA3893 | *-* | cell wall hydrolase, putative |
| GBAA4747 | *-* | dna-binding protein |
| GBAA4748 | *-* | flagellar motor protein |
| GBAA5317 | *-* | methyl-accepting chemotaxis protein |
| GBAA5318 | *-* | endonuclease/exonuclease/phosphatase family |

Figure S50: L. monocytogenes cluster 102 image (post-elaboration)

**
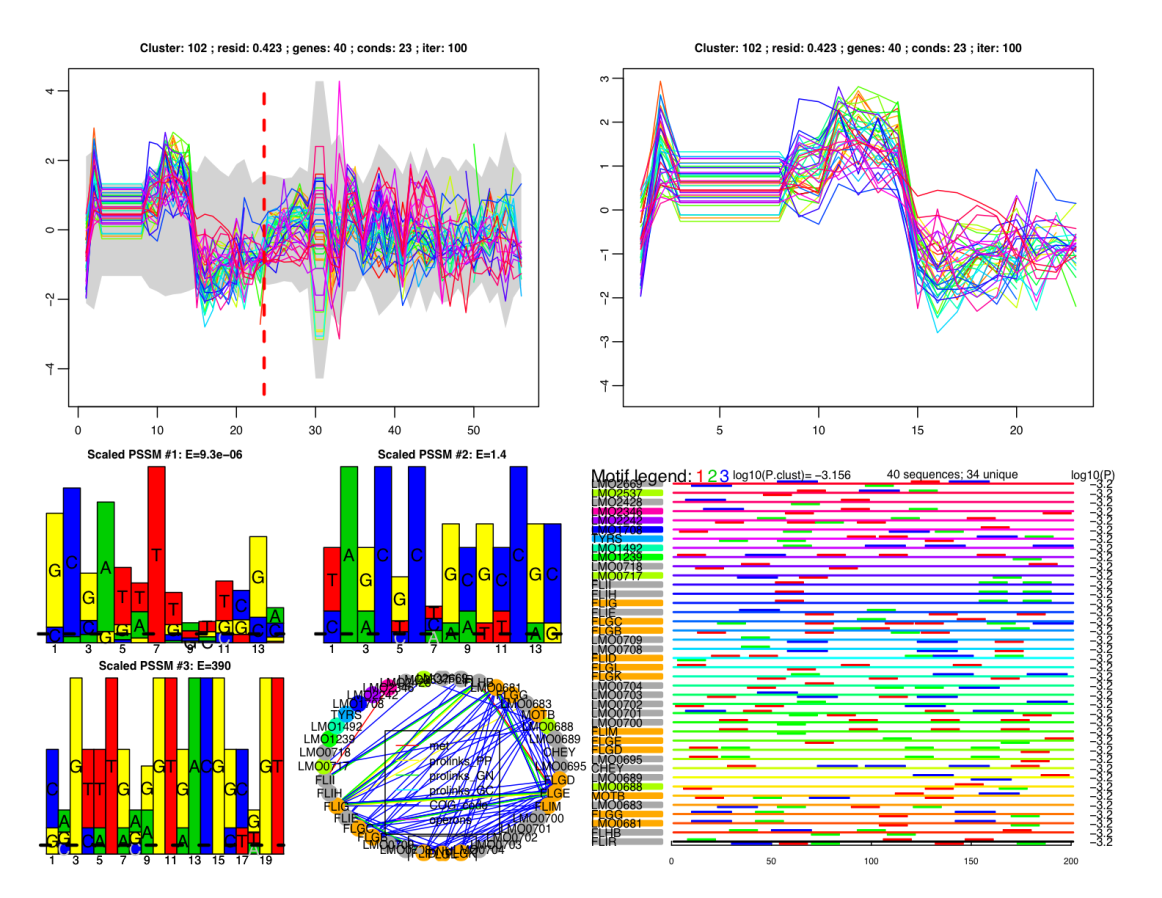
**

| ***L. monocytogenes* cluster 102 core genes** |  |  |
| --- | --- | --- |
| **Locus** | **Name** | **Function** |
| LMO0678 | *fliR* | flagellar biosynthesis protein FliR |
| LMO0696 | *flgD* | flagellar basal body rod modification protein |
| LMO0697 | *flgE* | flagellar hook protein FlgE |
| LMO0703 | *-* | hypothetical protein |
| LMO0705 | *flgK* | flagellar hook-associated protein FlgK |
| LMO0707 | *fliD* | flagellar capping protein |
| LMO0708 | *-* | hypothetical protein |
| LMO0710 | *flgB* | flagellar basal body rod protein FlgB |
| LMO0711 | *flgC* | flagellar basal body rod protein FlgC |
| LMO0712 | *fliE* | flagellar hook-basal body protein FliE |
| LMO0714 | *fliG* | flagellar motor switch protein G |
| LMO0715 | *fliH* | flagellar assembly protein H |
| LMO0716 | *fliI* | flagellum-specific ATP synthase |
| LMO0717 | *-* | hypothetical protein |
| LMO1598 | *tyrS* | tyrosyl-tRNA synthetase |
| ***L. monocytogenes* cluster 102 elaboration genes** |  |  |
| **Locus** | **Name** | **Function** |
| LMO0679 | *flhB* | flagellar biosynthesis protein FlhB |
| LMO0681 | *-* | flagellar biosynthesis regulator FlhF |
| LMO0682 | *flgG* | flagellar basal body rod protein FlgG |
| LMO0683 | *-* | hypothetical protein |
| LMO0686 | *motB* | hypothetical protein |
| LMO0688 | *-* | hypothetical protein |
| LMO0689 | *-* | hypothetical protein |
| LMO0691 | *cheY* | Chemotaxis response regulator CheY |
| LMO0695 | *-* | hypothetical protein |
| LMO0699 | *fliM* | flagellar motor switch protein FliM |
| LMO0700 | *-* | flagellar motor switch protein |
| LMO0701 | *-* | hypothetical protein |
| LMO0702 | *-* | hypothetical protein |
| LMO0704 | *-* | hypothetical protein |
| LMO0706 | *flgL* | flagellar hook-associated protein FlgL |
| LMO0709 | *-* | hypothetical protein |
| LMO0718 | *-* | hypothetical protein |
| LMO1239 | *-* | hypothetical protein |
| LMO1492 | *-* | hypothetical protein |
| LMO1708 | *-* | hypothetical protein |
| LMO2242 | *-* | hypothetical protein |
| LMO2346 | *-* | hypothetical protein |
| LMO2428 | *-* | hypothetical protein |
| LMO2537 | *-* | hypothetical protein |
| LMO2669 | *-* | hypothetical protein |

# Materials

## Data set analyzed

For *B. subtilis*, we compiled an expression data matrix that consisted of 314 conditions from 15 studies that examine the regulons of over 40 known transcriptional regulators and sigma factors [54, 95-111]. For the two pathogens, the *L. monocytogenes* expression matrix contained 56 conditions that were compiled from 8 studies covering early stationary phase, salt, alkali, and cold shocks [112-118]; while the *B. anthracis* matrix contained 51 conditions from a single study by Bergman et al [64] covering the full life-cycle of the *B. anthracis* Sterne strain. Most data was collected from the GEO omnibus database [119, 120], though additional *B. subtilis* data also came from the KEGG Expression Database [121]. In addition to these expression data sets, we also included upstream sequence data (200 bases upstream of the start codon), retrieved from RSA Tools [89] as well as network associations from KEGG [23, 122-124], Prolinks [24] and Predictome [25].

We used InParanoid to identify putative sets of orthologs between these three species. Using InParanoid with the default settings (BLOSSUM45 substitution matrix), we identified 2225 orthologous groups between *B. subtilis* and *B. anthracis*, 1439 between *B. subtilis* and *L. monocytogenes*, and 1494 between *B. anthracis* and *L. monocytogenes*. Note, that while these are the total number of groups, the total number of genes and orthologous pairs is larger as we also include non-best-matching orthologs in our analysis. Tables S1 and S2 provide full listings of the number of genes, conditions and edges (by network association) in our database for each organism, as well as the total number of genes, orthologs and ortholog families for each organism pairing.

Table S19: Size of the data sets used for the multi-species analysis, by organism.

| **Number of:** |  | ***Bacillus subtilis*** | ***Bacillus anthracis*** | ***Listeria monocytogenes*** |
| --- | --- | --- | --- | --- |
|  | **genes** | 3928 | 5861 | 2795 |
|  | **conditions** | 314 | 51 | 56 |
| **association edges:** |  |  |  |  |
|  | **operon** | 839 | 997 | 494 |
|  | **metabolic (KEGG)** | 49630 | 73981 | 36825 |
|  | **GN (Prolinks)*** | 6105 | 7338 | 1982 |
|  | **PP (Prolinks) *** | 6036 | 7703 | 1970 |
|  | **GC (Prolinks) *** | 839 | 997 | 494 |
|  | **COG-code** | 227096 | 370354 | 110489 |
|  |  |  |  |  |
| *Prolinks codes: GN = Gene Neighbor, PP = Phylogenetic Profile, GC = Gene Cluster | | | | |

Table S20: Total number of orthologs, orthologous families, and ortholog pairs generated by InParanoid, by organism pairings.

| **Number of:** | ***B. subtilis- B. anthracis*** | ***B. subtilis- L. monocytogenes*** | ***B. anthracis- L. monocytogenes*** |
| --- | --- | --- | --- |
| **orthologous groups** | 2225 | 1439 | 1494 |
| **orthologous pairs** | 2443 | 1564 | 1690 |
| **multi-member groups** | 118 | 95 | 129 |
| **Remaining unique genes (per organism)** | *B. subtilis*: 2279 | *B. subtilis*: 1519 | *B. anthracis*: 1634 |
|  | *B. anthracis*: 2339 | *L. monocytogenes*: 1478 | *L. monocytogenes*: 1537 |

## External tools used

Ortholog analysis and identification was performed using InParanoid version 2.0 on protein sequences in fasta format that were retrieved from NCBI Bacterial Genomes (<ftp://ftp.ncbi.nih.gov/genomes/Bacteria/>), and using BLAST version 2.2.10. During the cMonkey optimizations, MEME & MAST version 3.5.7 was used as part of the iterative search for new motifs. Upstream sequences were retrieved from Regulatory Sequence Analysis Tools (RSAT) {Thomas-Chollier, 2008 #252;van Helden, 2003 #87. All GO term enrichments were calculated using the GO-TermFinder library {Sherlock, 2009 #239, using a Bonferonni false discovery correction. All KEGG pathway enrichments were calculated using a utility built in-house for this purpose; also Bonferonni corrected.

# Methods

## Additional multi-species cMonkey biclustering steps:

Below, we describe in greater detail the seeding procedure used to generate the “shared” seeds for multi-species biclustering optimization, as well as the “Extend” optimization step, following the two multi-species optimization steps, which identifies species-specific modules.

### Seeding the initial biclusters:

For any two genomes, ***G_U_*** and ***G_V_****,* we use***OC_U_***and***OC_V_*** to refer to the portions of these genomes with one or more orthologs in the other genome, which we term the ‘*Orthologous Cores*’ (OC) of these genomes. Furthermore, we will use ***OC_UV_***(U: genome 1; V: genome 2) to refer to the list of *all possible* pairings of orthologs between the species, which for convenience we will refer to as ‘*orthologous pairs*’. To begin the multi-species cMonkey algorithm we must seed a new bicluster from the set of all ortholog pairs, ***OC_UV_***. Using a strategy similar to the one employed by the original *cMonkey* algorithm, an ortholog pair from ***OC_UV_*** is randomly selected, with the most differentially expressed conditions for each organism’s respective ortholog selected. Thus, if we let ***X_U_*** and ***X_V_*** represent the expression datasets for the two genomes, we randomly select a pair from ***OC_UV_***, and then select as conditions the most differentially expressed conditions in ***X_U_*** and ***X_V_*** that correspond to the genes for that pair, ***X_U-Diff_*** and ***X_V-Diff_***.

To complete the seeding process we expand this cluster by adding a set number of pairs with the best ‘pair’-wise correlation to this seed pair over the conditions where the genes of the seed pair are most differentially expressed in each organism. To accomplish this, we first calculate the within-species correlations to the genes of the seed pair for all genes in the orthologous core for the respective organism, considering only those conditions in ***X_U-Diff_*** and ***X_V-Diff_***. To convert these ‘gene’-wise correlations to ‘ortholog-pair-wise’ correlations, for each pair we simply multiply the correlations of its two gene members together. To avoid multiplicative inversion, we set all negative correlations to 0 prior to this multiplication. As a last step we must choose the conditions for each seed, within each organism’s single species data space, we calculate the average expression of the genes in the seed bicluster across ***X_U-Diff_*** and ***X_V-Diff_***, and select the most differentially expressed conditions for the seed bicluster.

Given this seed, iterations are performed using a coupled scoring function, described below, to decide if pairs of genes in ***OC_UV_*** should be either added or dropped from this bicluster. After convergence we seed another bicluster and repeat the process until we reach the user specified number of biclusters.

### Extend to include species-specific modules:

As a final step, we can search for biclusters that have no conserved component and no overlap with genes included in multi-species biclusters. This step is not the main focus of this work, it is described for completeness. If complete coverage of a single species data-space/genome is a requirement (for example if the biclusters are to serve as the basis for downstream analysis such as network inference) we can generate additional biclusters that are seeded and optimized exactly in the same way as the original *cMonkey* but restricted to genes not found in shared (conserved) and elaborated (partially conserved) biclusters.

## Multi-Species Iterative Signature Algorithm

We re-implemented a multi-species version of the Iterative Signature Algorithm (ISA) described by Bergmann et al {Bergmann, 2003 #10}, using the isa2 package for R [13, 81], available from CRAN. Our results show that this multi-species version of ISA produced far fewer conserved multi-species biclusters that are far less conserved than either the multi-species k-means or cMonkey methods.

The previously described multi-species ISA method can be described in five main steps:

1. A well-characterized organism is used as a ‘reference’ organism, with a less characterized organism as the ‘target’ organism (note, we use the terminology of a later paper from the same group [35] which employs a similar strategy for multi-species comparisons).
2. Using a pre-generated set of biclusters from the reference organism, biclusters containing genes that have putative orthologs in the target organism are selected and used to generate ‘homologous’ biclusters for the target organism that contain these putative orthologs such that there is a direct one-to-one mapping between the biclusters for both organisms.
3. ISA is performed on the target organism, using only these homologous biclusters as seeds.
4. The intersection of the input to and results from step 3 are selected to generate a set of 'purified’ biclusters in order to select only the conserved genes in the reference organism.
5. In the final step, ISA is run a second time, but using the purified biclusters to generate a set of 'refined’ biclusters. As such, this step is similar to the elaboration step of the multi-species cMonkey method as it is allows species-specific modifications to be added to the purified bicluster.

Our analysis used *B. subtilis* as the reference organism as it is the best characterized of the three organisms in our study, as well as having the largest expression data set. To generate the original set of biclusters, we employed a strategy similar to that described in [125] where we varied the gene threshold (t_g_) over the range of 2.0 to 4.0 in steps of 0.2, and for each gene threshold used, we varied the condition threshold (t_c_) over the range of 2 to 4.0 in steps of 0.5. For each combination of gene and condition threshold, we used nearly 24000 starting points, with these consisting of 2 types, the first being a starting point for each individual gene (~4000) and the second consisting of 20000 random pairings of genes. Following the strategy described by the authors of the tutorial for the isa2 R package (<http://www2.unil.ch/cbg/homepage/ISA_tutorial.html>), these were merged and filtered for ‘robustness’ (a metric of bicluster quality provided in the isa2 package; we direct the reader to the tutorial for more information) using built-in functions from the isa2 package. This strategy produced over 150 biclusters, though subsequent analysis still showed a high degree of overlap among these. For this reason, we merged these further using a two-step process. First, biclusters with more than 85% element-wise overlap (overlap of matrix entries, i.e. the expression of a single gene, for a single condition) were merged, and in the second step, biclusters with greater than 90% gene-wise overlap were merged, producing a final set of 41 biclusters.

Using this set of 41 *B. subtilis* biclusters as the reference, we replicated the multi-species biclustering process described above for both *B. anthracis* and *L. monocytogenes*, with the one following difference. A feature of the isa2 package allows the user to specify the ‘direction’ of the biclusters that are found, whether “updown” (the default), “up”, or “down” (we direct the reader to the package documentation for explanation of this argument for the isa.iterate function). For completeness, during each step that required the application of ISA, we ran ISA three times for each reference bicluster, once for each direction. As this can produce three possible biclusters in the target organism (one for each direction), we selected the bicluster with the greatest robustness score as the representative bicluster in the target organism for the bicluster from the reference organism. Note, in some cases, no bicluster was produced in the target organism regardless of the direction used in the analysis. For the pairing of *B. subtilis* and *B. anthracis*, this resulted in 41 multi-species biclusters, while for the pairing of *B. subtilis* and *L. monocytogenes*, this produced 38 multi-species biclusters (3 biclusters were filtered as they contained no genes).

## Explanation of the (bi)cluster coherence metrics

### Residuals

Cheng and Church [6] originally introduced residuals as a measure of bicluster coherence. For our purposes, we use a modified version of the residual measure used that takes into account gene-wise expression variance. Thus, if we let *x_ij_* be the expression value for gene *g* in condition *c*, these are defined for any bicluster containing a set of *G* genes over *C* conditions as:

$$resid\left(G,C\right)=\left(\frac{\frac{1}{\left|G\right|\left|C\right|}\sum_{g\in G, c\in C} abs\left(x_{gc}-x_{gC}-x_{Gc}+x_{GC}\right)}{\frac{1}{\left|G\right|}\sum_{g\in G} var_{C}\left(x_{g}\right)}\right)$$

where

$$x_{gC}=\frac{1}{\left|C\right|}\sum_{c\in C} x_{gc}, x_{Gc}=\frac{1}{\left|G\right|}\sum_{g\in G} x_{gc}, x_{GC}\frac{1}{\left|G\right|\left| C\right|}\sum_{g\in G,c\in C} x_{gc}$$

and

$$\mathrm{var}_{C}\left(x_{g}\right)=\frac{1}{\left|C\right|}\sum_{c\in C} \left(x_{gc}-x_{gC}\right)^{2}$$

As such, they can be understood to be a measure of the average deviation from the signal present within the bicluster, normalized by the average variance of the genes in *G* for the conditions in *C*. As a simple comparison, the residuals from the (bi)clusters produced by each method were pooled and compared with each other using two-sided Wilcoxon’s non-parametric rank tests. We direct the reader to Figure S1Figure S3), as well as Table S4 (FD-MSCM Table S9 (EO-MSCM) and Table S14 (randomized tests) for the results of these comparisons, for each pairing of organisms.

### Mean correlations

We also evaluated (bi)cluster expression coherence using the average pairwise correlation between genes in a (bi)cluster, over the conditions in the (bi)cluster. Because some of the methods we evaluated in this study could identify biclusters with inversely correlated patterns of expression, we took the absolute values of these correlations. Thus, if we let *x_ij_* be the expression value for gene *g* in condition *c*, the mean correlations are defined for any bicluster containing a set of *G* genes over *C* conditions as:

$$mean.cor\left(G,C\right)=\left(\frac{\left|G\right|\left(\left|G\right|-1\right)}{2}\right)^{-1}\left(\sum_{x,y \in G; x \neq y} abs\left(\frac{\sum_{c \in C} \left(x_{c}-\bar{x}\right)\left(y_{c}-\bar{y}\right)}{\sqrt{\sum_{c \in C} \left(x_{c}-\bar{x}\right)^{2}\sum_{c \in C} \left(y_{c}-\bar{y}\right)^{2}}}\right)\right)$$

As a simple comparison, the average pairwise correlations from the (bi)clusters produced by each method were pooled and compared with each other using two-sided Wilcoxon’s non-parametric rank tests. We direct the reader to Figure S4Figure S6, as well as Table S5 (FD-MSCM), Table S10 (EO-MSCM) and Table S15 (randomized tests) for the results of these comparisons.

### Network Association p-values

Briefly, the association p-values for a bicluster are modeled using a hypergeometric distribution, where for a given bicluster *b_k_* for genome *G*, the association p-value for an individual network, *N*, is calculated as:

$$pvalue\left( b_{k},N\right)=\frac{\left(\begin{matrix} \left|N\right| \\ n_{b_{k}\to b_{k}} \end{matrix}\right)\left(\begin{matrix} poss\left(G\right)-\left|N\right| \\ poss\left(b_{k}\right)-n_{b_{k}\to b_{k}} \end{matrix}\right)}{\left(\begin{matrix} poss\left(G\right) \\ poss\left(b_{k}\right) \end{matrix}\right)}$$

Whereis the number of edges in *N* shared between the genes in *b_k_*; and for any given set of vertices, *X*, *poss(X)* is the number of edges if *X* were completely connected, i.e.

$$poss\left(X\right)=\frac{\left|X\right|\left(\left|X\right|-1\right)}{2}$$

As a simple comparison, the association p-values for all network types were pooled together (Figures 5, S5 and S6) and compared using two-sided Wilcoxon’s non-parametric rank tests. We direct the reader to Figure S7Figure S9, as well as Table S6 (FD-MSCM) and Table S11 (EO-MSCM) for the results of these comparisons.

### Motif E-values

Motif E-values were generated by MEME, the motif discovery tool used by cMonkey [49]. MEME uses a metric, called an E-value, which was first described by Hertz and Stormo [126] with the aim to assess the statistical significance of the information content (or relative entropy) of a sequence motif, defined as [127]. Thus, for a given motif, an E-value is an estimate of the expected number of motifs of the same length that have the same or greater information content as the motif being considered. The E-value can be interpreted as the score for a one-sided p-value for the null distribution of information content for motifs of a given length. Therefore, the larger the E-value of a motif, the less significant it is; the smaller the E-value, the more significant it is. As a simple comparison, the E-values from the (bi)clusters produced by each method were pooled and compared with each other using two-sided Wilcoxon’s non-parametric rank tests. In this case, we selected the first motif identified by MEME for the (bi)clusters (as these are generally the most reliable). We direct the reader to Figure S10Figure S12, as well as Table S7 (FD-MSCM) and Table S12 (EO-MSCM) for the results of these comparisons.

### Sequence p-values

In addition to the motif E-values, we also compared the distributions of the sequence p-values that were returned by MAST, the motif search utility used by cMonkey [128]. Briefly, sequence p-values are an estimate of the significance of a sequence’s match to one or more motifs, and can be understood to be a measure of the likelihood of a random sequence having as good or better match or matches. For a given sequence and motif, the motif’s PSSM is used to score the degree of the match (likelihood of a match) to a sliding window across the length of the sequence, with the maximal match selected as sequence’s score for that motif. The p-value reported by MAST, then, is simply this score if working with a single motif, and in the case of multiple motifs, it is the multiplication (or addition if using log-likelihoods) of the individual motifs match score to the sequence. To compare each optimization, then, we calculated the average p-value for the genes in each bi(cluster) with respect to the bi(cluster’s) associated motifs, and compared the distributions of these. We direct the reader to Figure S13Figure S15, as well as Table S8 (FD-MSCM) and Table S13 (EO-MSCM) for these comparisons.
